# Bisulfite probing reveals DNA structural intricacies

**DOI:** 10.1093/nar/gkad115

**Published:** 2023-03-07

**Authors:** Andrew T M Bagshaw, Neil J Gemmell

**Affiliations:** Department of Anatomy, School of Biomedical Sciences, University of Otago, PO Box 56, Dunedin 9054, New Zealand; Department of Anatomy, School of Biomedical Sciences, University of Otago, PO Box 56, Dunedin 9054, New Zealand

## Abstract

In recent decades, study of DNA structure has largely been focused on the interrelationships between nucleotides at the level of nearest neighbours. A little-utilized approach to probing structure on a larger scale is non-denaturing bisulfite modification of genomic DNA in conjunction with high-throughput sequencing. This technique revealed a marked gradient in reactivity increasing towards the 5′ end of poly-dC:dG mononucleotide repeats as short as two base pairs, suggesting that access of the anion may be greater at these points due to positive-roll bending not predicted by existing models. Consistent with this, the 5′ ends of these repeats are strikingly enriched at positions relative to the nucleosome dyad that bend towards the major groove, while their 3′ ends tend to sit outside these areas. Mutation rates are also higher at the 5′ ends of poly-dC:dG when CpG dinucleotides are excluded. These findings shed light on the mechanisms underlying bending/flexibility of the DNA double helix as well as the sequences that facilitate DNA packaging.

## INTRODUCTION

Although the structure of the DNA double-helix at the level of the relative angular positions of adjacent base pairs (dinucleotide steps) has been explored in great detail, larger-scale influences are less well understood. Sequence features such as mononucleotide runs have been known for decades to influence helical flexibility (1), but applications that involve prediction of DNA bending, such as the modelling of nucleosome binding, currently rely on dinucleotide models (2).

A well-established approach to examining fine structural aspects of native DNA at a broader scale using strand cleavage by the hydroxyl radical (3) is limited by incomplete understanding of its mechanism. An alternative method uses non-denaturing bisulfite treatment followed by PCR amplification and high-throughput sequencing. This is similar to commonly used methods for mapping cytosine methylation using bisulfite, with the exception that double-stranded DNA is treated, so in theory only unmethylated cytosines at regions that are single-stranded for structural reasons should convert to uracil and ultimately be detectable as thymine by sequencing.

The main previous application of bisulfite as a probe of DNA structure has been in helping to characterize discrete non B-DNA structural features with single-stranded components including intramolecular triplexes, quadruplexes and cruciforms (4–6), and more recently to map R-loops genome wide (7). These studies were confined to examining relatively large structural entities, and bisulfite has generally been overlooked for finer probing because it reacts only very weakly with double-stranded DNA. An exception to this was an investigation of the Bcl-2 translocation breakpoint implicated in Burkitt's Lymphoma, which showed that non-denaturing bisulfite sequencing is sensitive enough to detect subtle conformational change at the translocation-associated sequence GGGCCC, noting that this might be explained by its B-A-form transition characteristics (8).

Specifically, it was proposed that the positive roll-bend angle and decreased base-pair lifetimes seen at the motif using crystallographic and spectroscopic methods could allow easier access for the bisulfite anion. Positive-roll/major-groove-directed bending at the motif GGGCCC has been indicated by multiple other methods (8–11), and base-pair stability is lowest at the central GC dinucleotide of this motif (10). A previous observation that bisulfite reactivity is significantly elevated in supercoiled plasmid DNA (4) is also relevant, since supercoiling has been associated with both bending and decreased base-pair stability (12). However, while it has been proposed that increased presence of divalent cations in the major groove of GGGCCC motifs could promote their observed bending, and that the same could occur at G:C-rich sequences in general (1), the idea that the increasing structural perturbation towards the central point of GGGCCC considered as the convergence of the 5′ ends of two adjacent poly-C runs might also apply to the 5′ ends of poly-C in other contexts has not been studied.

In addition to the motif GGGCCC, known influences on DNA bending that are not well modelled at the tetranucleotide or lower levels include A-tracts, defined as four or more consecutive A:T base pairs containing no TA dinucleotides (9,13,14). A-tracts are relatively inflexible, but bending of DNA flanking A-tracts has been shown, and this can be directed oppositely to GGGCCC, i.e. narrowing the minor groove, at junction points with non-A-tract DNA (1,9,14,15). If bisulfite reactivity reflects DNA bending, the effect of neighbouring A-tracts might therefore be suppressive.

Data on mutational patterns suggest an additional indirect test of differential bending properties between the ends of poly-dC:dG runs (hereafter referred to as poly-C), e.g. a correlation between DNA bending based on roll and tilt parameters and the mismatched base pairs that antecede inherited mutations has been shown (16). Evidence of major-groove-directed bending by mismatched duplexes (31) and elevated structural perturbations by transversions including increased roll bending at A:C mismatches (32) are also relevant to the hypothesis that polar structure at poly-C influences mutability, though it is also notable that helical flexibility is expected to facilitate mismatch repair (16). Apparently contrary to the hypothesis of mutation-promoting structural abnormality at the 5′ ends of poly-C runs, elevated polymorphism near the 3′ ends of poly-C, thought to be due to an electrostatic hole created by loss of an electron due to oxidation, has been reported (17). However, that analysis included the motif CpG, the hypermutability of which is driven by the vulnerability of 5-methylcytosine to deamination, to which DNA bending/flexibility is not necessarily related (18). Analysis excluding CpG dinucleotides has not, to our knowledge, been published.

We sought to test these hypotheses using deep sequencing of bisulfite-modified protein-free native DNA, in conjunction with available datasets on de novo mutations of the human genome.

## MATERIALS AND METHODS

### Reagents

Sodium bisulfite (cat. # 243973) and hydroquinone (cat. # 74347) were from Sigma Aldrich. The Wizard DNA clean-up kit (cat. # A7231) and Proteinase K (cat. # V3021) were from Promega. DNA extraction was done with a Norgen Biotek Blood DNA Isolation Mini Kit (Cat# 46300). Degenerate PCR primers were custom made by Integrated DNA Technologies.

### DNA extraction

DNA was extracted from buffy coat cells of an individual human male according to the kit manufacturer's instructions. Briefly, Proteinase K was added to a microcentrifuge tube, followed by 200 μl of buffy coat. Next, 300 μl of lysis solution was added, and samples were vortexed and incubated at 55 °C for 10 min. Following ethanol precipitation, samples were given an additional incubation at 37°C for 16–24 h with Proteinase-K, followed by re-precipitation with ethanol and suspension in 30 μl dd H_2_O.

### Bisulfite treatment

We carried out bisulfite modification as described (4). For each reaction, 0.3 g of sodium bisulfite was first dissolved in 1050 μl dd H_2_O/g sodium bisulfite. To this was added 525.2 μl of 2 M NaOH/g sodium bisulfite. Once dissolved, the pH of the solution was confirmed to be 5.2–5.3. In a 1.5 mL microcentrifuge tube, 457.5 μl of the sodium bisulfite solution was then mixed with 12 μl of 20 mM hydroquinone. To this was added 1 μg of genomic DNA in 30 μl of dd H_2_O with pipette mixing. The solution was then incubated for 16 h at 37°C in the dark. The DNA was purified using a Wizard DNA clean-up kit according to the manufacturer's protocol, and then desulfonated with 0.3 M NaOH for 15 min at 37°C. Following this, ethanol precipitation was carried out for 1 h at –20°C, followed by three washes with 70% ethanol, and suspension in 30 μl Tris–EDTA (pH 8.0).

### PCR amplification

We PCR-amplified twelve bisulfite-modified promoter, coding and intergenic regions of 1.7–2 kb from human Chromosome 21. This size range was selected for convenient sequencing library preparation for Illumina Miseq. Degenerate primers were used to reduce PCR bias towards less reacted molecules. These had 50% A residues at each G, and 50% T at each C (see Supplementary Table S1 for primer sequences). Thermal cycling was as follows: initial denaturation at 95°C for 3 min, followed by 10 cycles of 98°C for 20 s, 60°C for 2 min and 72°C for 1:30, and then 25 cycles of 98°C for 15 s, 45°C for 45 s and 72°C for 1:30, with a 5-min final extension at 72°C. This protocol was intended to maximize yield while minimizing non-specific amplification due to the degenerate primers. It produced mostly clean products, as visualized under UV light following ethidium bromide staining. Amplicons were cleaned using magnetic beads. Preliminary experiments showed these PCR methods yielded bisulfite conversion levels comparable to published studies (4,8).

### Sequencing

The amplicon library was sequenced with one Illumina MiSeq 2 × 300 base PE run, version 3 chemistry, at 5% volume (New Zealand Genomics Ltd; Massey University). Quality control included trimming both ends of all reads up to the first base with a sequencing quality score of 32 (*p* = 0.00063). All remaining reads shorter than 500 bp and all reads containing any base with a sequencing quality score lower than 22 (*p* = 0.0063) were also removed.

### Alignment and variant mapping

Following quality control, a total of 21 kb was aligned to the reference sequence using Bowtie-2 with ‘sensitive’ settings. All bases covered by less than 500 reads were excluded from further analysis, as were those with mapping quality scores lower than 20 (*p* = 0.01). Each base was scored for numbers of each unexpected nucleotide using BAM-readcount (19). Both strands were combined in the results, i. e. a G → A transition was interpreted as C → T on the other strand, and polarity was accounted for in the analysis of poly-C runs, e.g. DCC*C*D was combined with H*G*GGH.

### Statistical analyses

Statistical analysis was done in R (Version 4.1). Significant differences involving bending and mutation rate statistics vs nucleosome position were determined by two-tailed T- or chi-square tests. The effects of A-tracts and local A:T content on bisulfite reactivity were evaluated using a generalized linear model (quasibinomial family in R). For the analysis of cyclization data from Basu et al. (20), the mean of the three experiments (‘c26’, ‘c29’ and ‘c31’) was taken and used as the independent variable in linear regression against the frequencies of sequence motifs per 50mer, or bisulfite reactivities based on 4- or 7-bp sliding windows as specified. The plots of bisulfite reactivity vs nucleosome position (Supplementary Figure S3) were based on 7-bp sliding windows, with values determined by averaging reactivity from Dumelie *et al.* (7) at the central points of each heptameric context in the data. Mutation rate was defined as the number of de novo mutations of a motif, based on data from Kessler et al. divided by the number of instances of the motif within the region concerned.

Operations involving large datasets employed bespoke Bash or Python scripts, which available at https://doi.org/10.5281/zenodo.7613454. Genome-wide data on nucleosome dyad positions were from Gaffney *et al.* (2012). The level of roll-bending at each position of predicted 146 bp nucleosome-bound regions was based on data from x-ray crystal structures (2). For the purposes of this work, the positions of these regions were practically identical to those determined by other studies (21).

## RESULTS

### A gradient in bisulfite reactivity across poly-C

**Figure 1.**
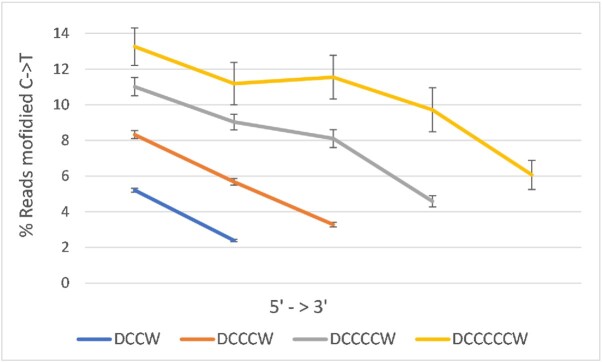
The polar gradient shown by bisulfite reactivity. The *y*-axis shows mean percentages of reads showing C → T transition relative to the reference. CpG dinucleotides, which are relatively unreactive (Supplementary Figure S2), but accentuate the gradient only mildly, were excluded. Mean read depths were 6592–7381, and the minimum depth was 500 reads. Strands were combined, i.e. G→A was considered C→T. Error bars are ± 1 SEM. ‘D’ represents any base other than C, and ‘W’ represents A or T. Isolated C (DCD) showed only 2% reactivity on average. Total numbers of poly-Cs analysed were 2104 (DCCW), 882 (DCCCW), 324 (DCCCCW) and 80 (DCCCCCW).

We examined just over 19 kb of protein-free genomic DNA from human Chromosome 21 with bisulfite treatment followed by PCR and deep sequencing. Out of a total of 7249 cytosines (Cs) covered by >500 reads, 99% appeared as thymine (T) in at least one read (Supplementary Table S2). poly-C showed substantially elevated reactivity, which increased with their length (Figure 1), while isolated C (DCD, where D = A, T or G) was converted in only just over 2% of reads.

Upon visual examination of read alignments, the most obvious pattern was sharply increasing reactivity from right-to-left at poly-C and left-to-right at poly-G, i.e. a 3′→5′ (polar) gradient, even in the absence of 3′ CpG dinucleotides, i.e. D(C)_*n*_W (Figure 1). This was evident for Poly-C runs as short as two base-pairs. The pattern was also present in a separate dataset of bisulfite reactivity in human genomic DNA (7) (Supplementary Figure S1). The larger scale of that dataset allowed investigation of repeats longer than 5 bp, but these much rarer sequences showed only shallow gradients. Nonetheless, the gradient was evident at the vast majority of C/G runs, including 94% of GG/CC in the data generated for this study. There were no differences in sequencing/PCR error (percentage of non-reference reads not attributable to bisulfite modification, which averaged less than 0.5%) or read depth between the 3' and 5' ends of poly-C considered as all types combined, or separated by repeat length (*p* > 0.19 by *T*-test).

### Exceptionally high reactivity of G_n_C_n_ motifs

We confirmed the exceptionally high reactivity of the motif GGGCCC, previously found to react strongly with bisulfite (8). It also showed a high 5′:3′ reactivity ratio (2.9 on average), i.e. modification peaked at the central GC dinucleotide. In our data, eight of the ten most reactive motifs were of the form G_*n*_C_*n*_ (Table 1; see Supplementary Table S3 for the equivalent data from Dumelie and Jaffrey) (7). Consistent with Tsai *et al.* (8), A-forming potential measured at a trinucleotide-step level (22,23) was not a significant predictor of bisulfite reactivity.

**Table 1.** Contexts of the most bisulfite-reactive cytosines. Bases converted in more than 40% of reads were excluded due to the possibility of heterozygosity. The equivalent results from Dumelie and Jaffrey (7) can be found in Supplementary Table S2

% Reactivity	Context	% Reactivity	Context
32.5	GGG**C**CCC	30.3	CTC**C**ATC
29.1	GGG**C**CGG	27.4	GGG**C**CCG
26.9	GGG**C**CCA	26.6	GGG**C**CCC
26.2	GGG**C**CTC	26.1	GGG**C**CTC
25.4	AGG**C**ATA	24.6	GGA**C**CCT
23.7	GCC**C**CCA	23.7	GGG**C**CTG
23.4	GGC**C**CCA	23.3	GGG**C**CCC
22.9	GGC**C**CCC	22.2	GAA**C**ATA
22.2	GGG**C**TTG	22.4	AGG**C**CCC

### Absence of clear associations with established base-pair step parameters

We regressed the mean modification frequencies of each overlapping di- and tetranucleotide in the tested sequence against the canonical base-step parameters twist, roll, tilt, slide, shift and rise derived from molecular dynamics simulation (24). At the dinucleotide step level, slide (*p* = 0.005) and rise (*p* = 0.007) were weakly positively associated with reactivity, but at the tetranucleotide level the former was in a negative direction (*p* = 7 × 10^−8^), while the latter was weaker (*p* = 0.024). Salient factors influencing the lack of associations with the bending parameters tilt and roll were (i) that the highly reactive GC dinucleotide is relatively rigid according to the step data, having the second-lowest roll value among dinucleotides and (ii) CA and AC, positioned at the 3′ and 5′ ends of poly-C respectively, are considered to be very high- and very low-roll respectively. These attributes are well established in dinucleotide step parameter datasets based on molecular dynamics simulation (25–27), though context-dependence of the bending characteristics of CA and GC has been shown (21).

### Effects of flanking bases

**Figure 2.**
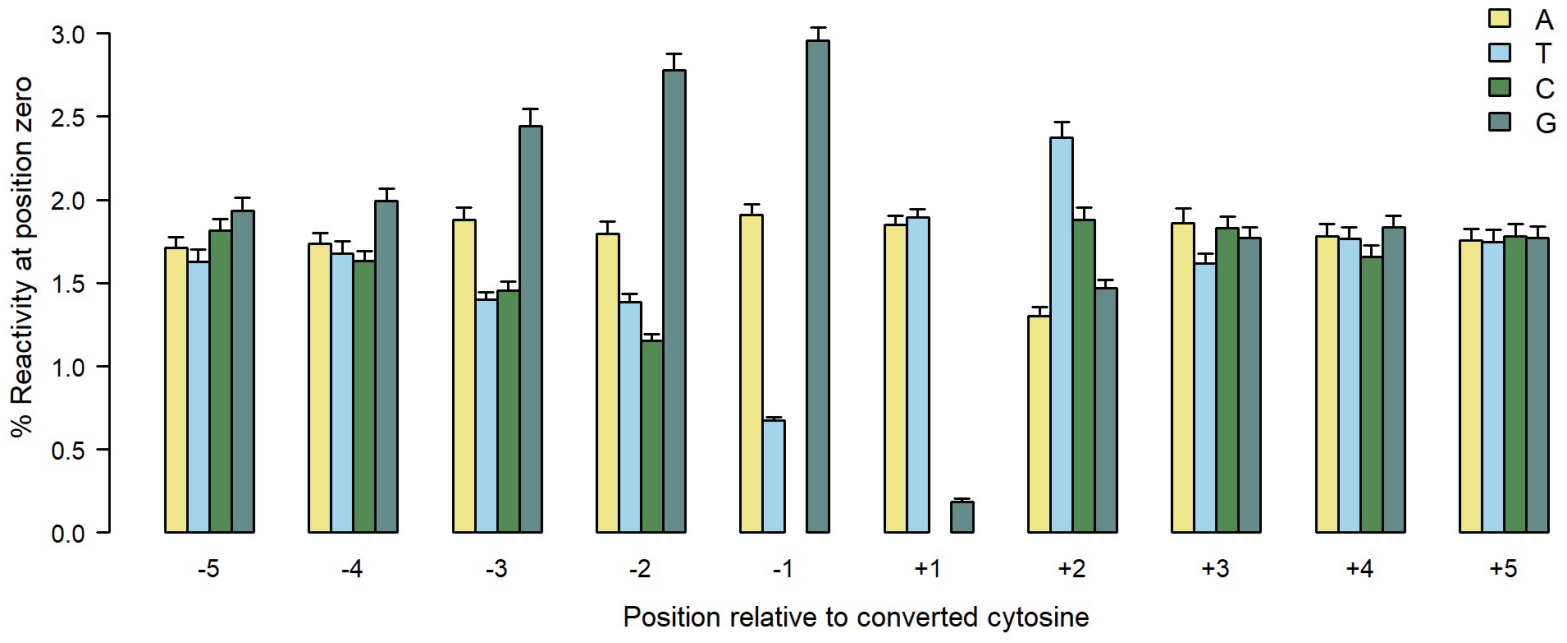
The effect of flanking sequence on bisulfite reactivity at isolated C (DCD). The bars represent mean reactivity levels at position zero associated with each particular flanking base, e. g. the mean reactivity of the dinucleotide GC was just over 3%. Similar patterns were seen at poly-C runs. The total number of Cs included was 3912. Error bars are 1 SEM.

Analysis of sequence to either side of each bisulfite-modified cytosine revealed clear contextual effects at flanking bases as distant as 3 bp (*p* < 10^−6^ by Wilcoxon signed-rank test; Figure 2). Most notably, 5′ G neighbours increased reactivity while 5′ T suppressed it. G as 3′ neighbour decreased reactivity even when CpG dinucleotides with little or no modification (<0.4%; a threshold chosen because it corresponded to a step in the plot of reactivity at these dinucleotides; Supplementary Figure S2) were excluded in order to indicate the effect of CpG methylation.

Given that reactivity of the motif GGGCCC peaks at its central GC dinucleotide, it is notable that GC dinucleotides in general were 46% more reactive than other dinucleotides, excluding CC. As noted in the previous section, this does not appear to be due to known intrinsic properties of the GC dinucleotide step. Although it is distinguished by high twist, this property is also shared with TC, which suppressed reactivity.

### Nucleosome-binding data indicate polar bending at poly-C

Examination of the distribution of poly-C sequences relative to bound nucleosomes provides an indirect test of the hypothesis that the 5′ ends of poly-C in general are bent towards the major groove. It has been known for several decades that frequencies of short GC-rich sequences, notably SS dinucleotides (two consecutive residues without an A or T) and the highly bisulfite-reactive GCC/GGC trinucleotide, show periodic maxima at positions where the major groove faces inward towards the protein and is narrowed, while AT-rich di- and trinucleotides are more often found with the opposite periodicity, i.e. in regions bent towards the minor groove (28,29) (Supplementary Figure S3 B).

**Figure 3.**
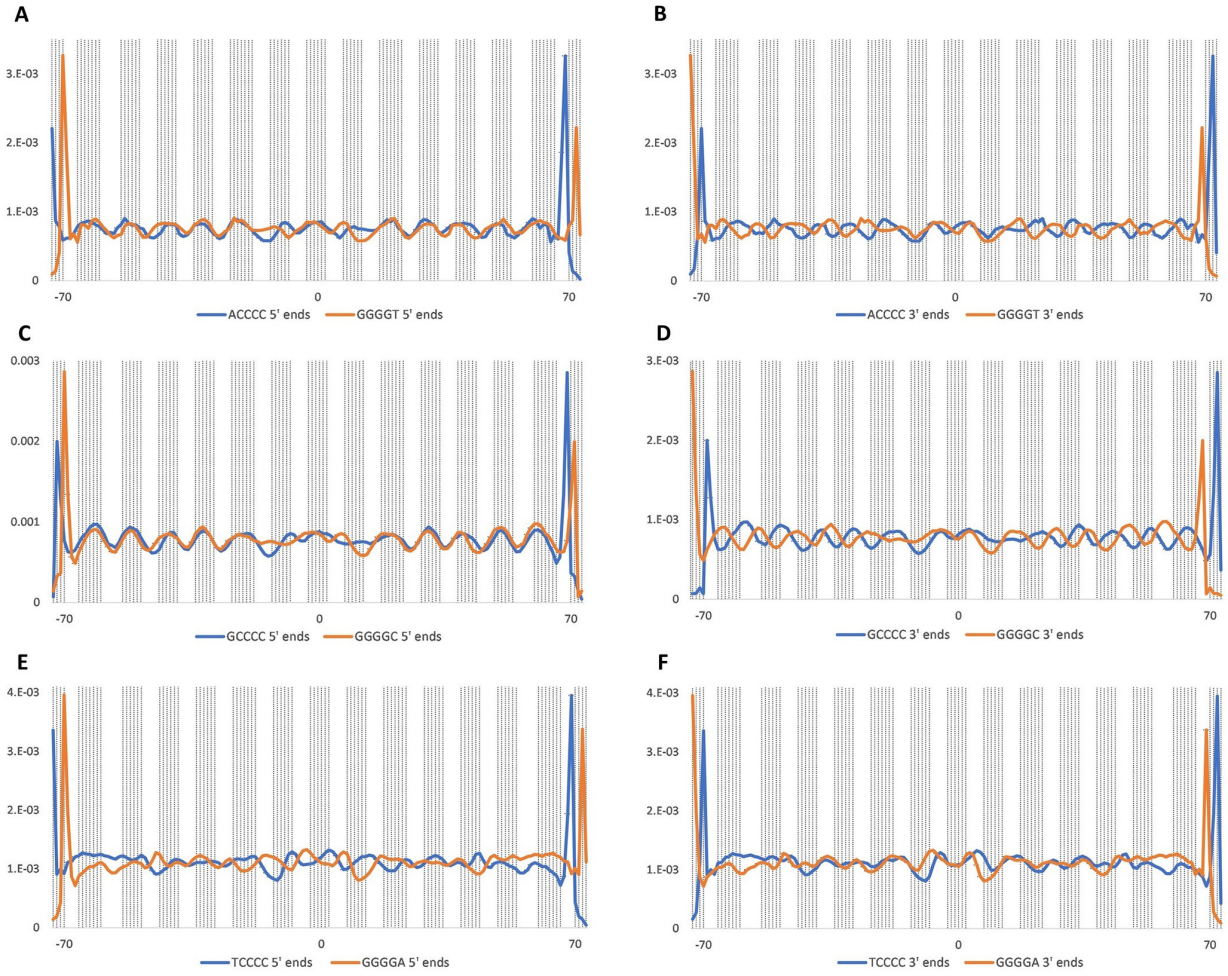
Comparison of the locations of the 5′ ends of poly-C_4_ (**A**, **C**, **E**) with their 3′ ends (**B**, **D**, **F**) relative to nucleosome positioning. Vertical dotted lines indicate areas with positive-roll bending when bound to nucleosomes, and the intervening white space corresponds to negative-roll bending (2). Point zero on the x-axes represents the dyad position, i.e. the point of internal symmetry of the nucleosome. Plots for other poly-C lengths up to eight bp, which show similar trends, can be found in Supplementary Figures S4–S7.

In our data, bisulfite modification per cytosine is correlated with sites that undergo positive roll bending to a clear, though undramatic, extent, and the correlation increases with increasing strength of nucleosome positioning (Supplementary Figure S3C, D) and increasing reactivity of the motif analyzed (Supplementary Figure S3E, F). More interesting patterns emerge on closer examination. Most notably, the 5′ ends of poly-C sequences are strikingly enriched in the central, most positively bent parts of these areas, while their 3′ ends tend to sit away from the peaks (Figures 3 and Supplementary Figure S4–S7). This trend begins at *n* = 2 for G(C_*n*_) and *n* = 3 for A(C_*n*_). The phenomenon is still visible at *n* = 8 (Supplementary Figure S7), though longer repeats are rare. The combination of a significant positive association with roll for 5′ ends (*P* < 0.05) and no such association for 3′ ends is evident for *n* = 3 and 4 for A(C_*n*_) and *n* = 2, 3 and 4 for G(C_*n*_). Consistent with the suppressive effect on bisulfite reactivity of 5′ T, T(C_*n*_) sequences do not show the pattern.

Interestingly, previous studies of GC-rich motifs associated with positively roll-bent regions of the nucleosome have shown that the patterns tend to be reversed near the nucleosome dyad (28) (Supplementary Figure S3 B). It is therefore notable that the 5′ ends of A(C_*n*_) repeats (Figure 3A, Supplementary Figures S4A, S5A, S6A and S7A), and the motifs most highly reactive with bisulfite (Supplementary Figure S3 F) are enriched in the three central peaks of positive-roll bending, though this is less clear for G(C_*n*_), and bisulfite reactivity overall.

### Suppressive effect of neighbouring A-tracts

Consistent with the hypothesis of major groove compression promoting bisulfite reactivity, and contrary to the expectation of higher reactivity due to greater DNA breathing in AT-rich regions, we observed decreasing reactivity with increasing flanking AT-content (Supplementary Table S3). Total reactivity was lower in the presence of a neighbouring A-tract by 42%, 44% and 41% for DCD, DCCD and DCCCD respectively. The effect was greatest for 5' A-tracts on the 5' C’s of poly-C. A-tracts had a weaker influence when located to the 3' side, where they were associated with 24–26% reductions. Taking into account local AT-content and the suppressive effect of 5' T residues, the influence of A-tracts was much reduced but remained significant (*p* < 0.02). Presence of a TA dinucleotide adjacent to the 5′ end of poly-C had a positive effect on bisulfite reactivity (*P* < 2.6 × 10^−5^ in the model presented in Supplementary Table S3). This is also consistent with a bending/flexibility-based structural explanation for the reactivity gradient at poly-C, because this highly flexible dinucleotide is known to be prone to positive roll bending (24,30).

### Correlation with bending measured by cyclization

An additional test of the link between bisulfite reactivity and DNA flexibility/bending is enabled by a dataset of ‘cyclizability’ scores measured for 82367 50-bp DNA fragments (20). Mean bisulfite reactivity per cytosine obtained for each 50mer using a 4 bp sliding window was correlated with mean cyclizability (*r* = 0.23; *p* < 10^−16^). Much weaker associations were evident for G/C-content and number of A-tracts per sequence (*r* = 0.03 and –0.04 respectively). Poly-C runs of 4 bp or more were enriched by 36% in the half of sequences with the most positive cyclizability scores (*p* < 10^−16^), and 3.6-fold in the top 1000 compared with the bottom 1000. While these correlations are quite weak, it is notable that the cyclizability data are noisy, with standard deviations from the three experiments exceeding the positive or negative magnitude of their mean scores in nearly half the tested sequences.

Also of interest in view of currently used dinucleotide step models, numbers of GC and CG dinucleotides per sequence were weak positive and negative predictors of cyclizability (*r* = 0.08 and –0.06, respectively), which is contrary to expectation based on their roll and tilt values. Further, the two strongest dinucleotide predictors were TA and TC (*r* = 0.21 and –0.16, respectively), and this is also consistent with the hypothesis that bisulfite reactivity reflects DNA bending in view of the strong negative and positive influences on reactivity of 5′-T and 5′-TA neighbours respectively.

### Polar mutation biases at poly-C

**Figure 4.**
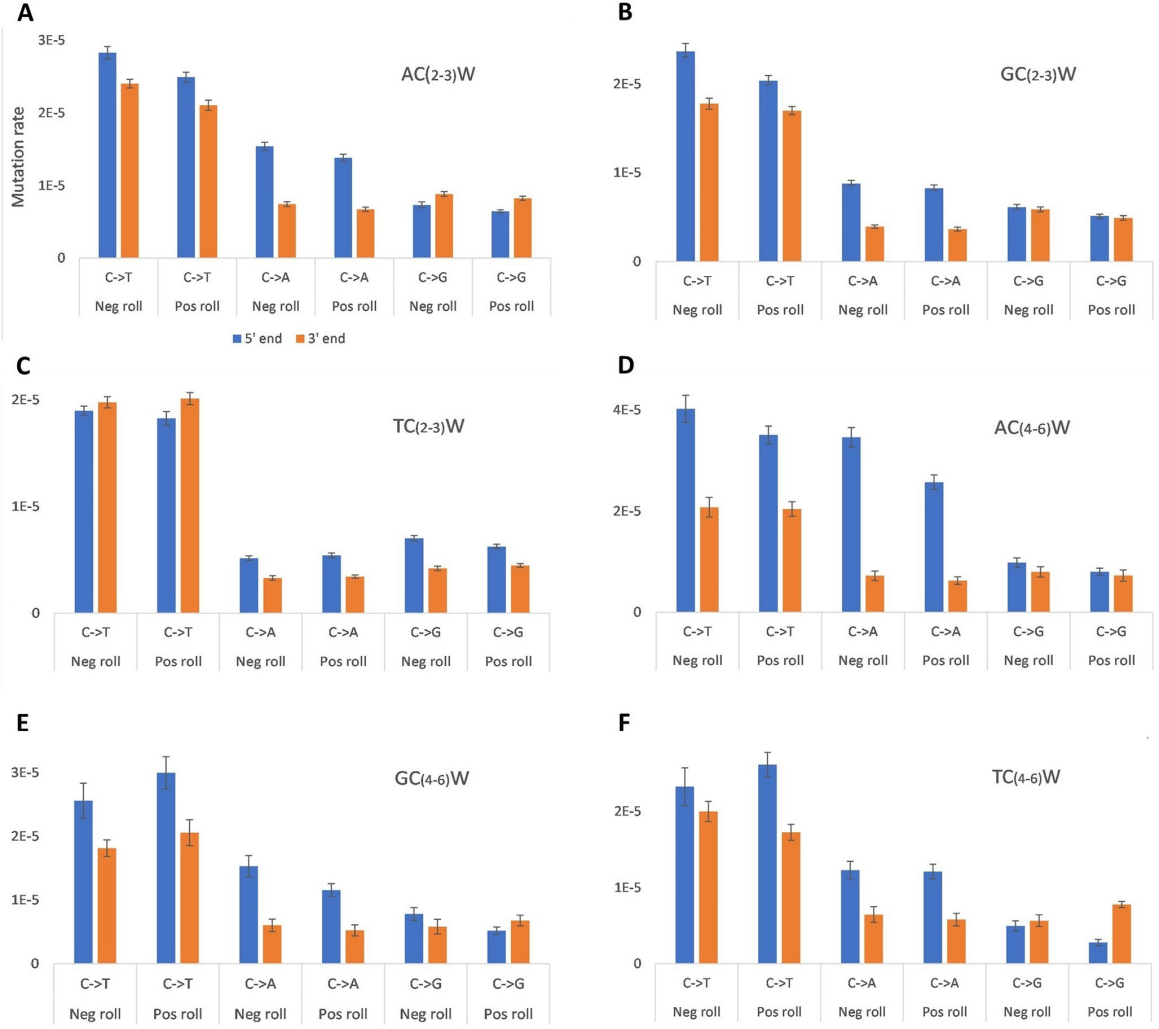
Mutation rates of Cs within various poly-C contexts. These were the 5′- and 3′-end C residues of poly-C_*n*_ where *n* = 2–3 (**A**–**C**) or 4–6 (**D**–**F**), 5′ flanking nucleotides A (A and D), G (B and E) or T (C and F), and position with respect to nucleosome bending. The latter was divided into positive and negative roll (2). Strands were combined, i.e. ‘C→T’ includes G→A mutations. Error bars are ± 1 SEM. Total numbers of mutations analysed were 2728, 1620, 2372, 269, 123 and 224 for (A)–(F), respectively.

Using a recent database of 93048 germline de novo mutations from human trios (31), we found that poly-C is more mutable at its 5′ than its 3′ end when adjacent 3′ G residues (i.e. all CpG dinucleotides) are excluded, for all combinations of flanking bases, by an average of 28% for runs of 2–5 bp (Supplementary Table S4). Consistent with bisulfite reactivity patterns, the gradient is much less marked in the presence of a 5′ T (Figure 4). It is much stronger for C → A transversions than for C → T transitions, but is oppositely directed for C → G transversions, which are enriched at poly-C 3′ ends, though they are comparatively rare (Figure 4).

Location with respect to nucleosome positioning has previously been shown to affect patterns of mutation (32), and the enrichment of WW and SS dinucleotides at sites with negative- and positive-roll bending respectively can be explained in part by elevated C→T mutability in the former (33). However, mutability of C_*n*_ runs divided between *n* = 2–3 and n = 4–6, as well as between 5′ A, G and T adjacent residues, is similarly distributed between positive- and negative-roll-bending regions of the nucleosome (Figure 4).

## DISCUSSION

Increased flexibility and/or base-pair opening at one end of poly-C repeats would be consistent with a hypothesis known as the ‘broken stack’ model of DNA bending (34). This predicts bending at runs of G:C base pairs due to a tendency for consecutive G residues to align/stack squarely (in parallel), necessitating periodic disruption in either stacking or Watson-Crick hydrogen bonding to accommodate the curved shape of the B-form double-helix. However, polarity in bending at poly-C was apparently not specifically invoked by the authors of this model.

Other well-known models of DNA bending have focussed on the role of A-tracts or cation binding (1). Our observation that poly-C is highly bisulfite-reactive is clearly consistent with the latter, given that its observed electropositive characteristic should attract the bisulfite anion (8). Our hypothesis of DNA bending predicts that the significance of the suppressive role of A-tracts is that they primarily direct bending oppositely to GGGCCC, i.e. narrowing the minor groove, which has been observed previously (9). The significance of the potentially related observation that neighbouring 5′ T residues are especially suppressive of reactivity is less clear. However, it is notable in this context that repeats of TCCA/TGGA have been found to be the most significant feature in random sequence with outstandingly low nucleosome occupancy (40). The authors of that study showed that the nucleosome-resisting property of TGGA-rich fragments was not due to quadruplex formation, so resistance to bending is a plausible explanation. In this context it is also notable that, in our data, sequences with quadruplex-forming potential also show the gradient (data not shown), which is unexpected if they actually form the structures in the absence of DNA-binding proteins or polymerase-induced supercoiling.

Transient single-strandedness due DNA breathing appears unlikely to contribute heavily to the patterns we observed, given that flanking A/T-content is negatively correlated with reactivity. Therefore, it is also noteworthy that steric inhibition should preclude the attachment of bisulfite to anti-form C, in which bases in the native Watson-Crick double-helix are locked (11). It may be reasonable to speculate that a permissive syn-form conformation, which has been demonstrated in the context of the double helix in Z-DNA (12), could play a role, at least in the context of existing structural perturbation. In the pH range we used for bisulfite treatment (5.2–5.3), some Cs are expected to be protonated in double-stranded DNA, as a result of which they can form a weakened Hoogsteen-type base pair with G, which adopts a syn configuration (35). The steric implications of the rotations about the sugar-base bond that these transformations would entail could presumably increase sensitivity to structural fluctuations.

Another possibility to be considered is that artefacts of bisulfite conversion contributed to the patterns we observed, for example if the conversion process stimulated reactivity at the 5′ end of poly-C. However, this seems less likely in view of observations that, in RNA, the G:U mismatch has been shown to create a region of strong electronegative potential (36). Further experimental work to confirm our hypotheses will require high technical sensitivity, as major groove compression at GGGCCC was not seen in solution NMR (11), and 5′ ends of poly-C aren’t necessarily bent in crystal structures. It has been hypothesized that detectable bending at GGGCCC motifs may require the presence of multivalent cations (1), and it is notable that Mg^2+^ concentration correlates positively with both nucleosome formation (37) and major-groove-directed bending (38). However, it appears that bisulfite probing may be sensitive enough to obviate the requirement for multivalent cations, as the principal cation in our reaction mixture was Na^+^, and there is some evidence that DNA binding of monovalent cations may be weak, at least for GC-rich tracts (39).

The phenomenon of increased electrostatic positive charge at poly-C is also reflected in enhanced reactivity of the hydroxyl radical at these sequences (3), though neither this nor other established methods indicate a polar structural gradient at poly-C (40,41). Bisulfite probing may therefore provide unique insight into the structural intricacies of GC-rich sequences.

## DATA AVAILABILITY

Data are deposited in the Genbank short read archive under accession PRJNA910828. Scripts are available at Zenodo, https://doi.org/10.5281/zenodo.7613454, and are also available upon request to the corresponding author.

## Supplementary Material

gkad115_Supplemental_Files

## References

[1] Hud N.V. , Plavec J. A unified model for the origin of DNA sequence-directed curvature. Biopolymers. 2003; 69:144–158. 12717729 10.1002/bip.10364

[2] Liu G. , Zhao H., Meng H., Xing Y., Cai L. A deformation energy model reveals sequence-dependent property of nucleosome positioning. Chromosoma. 2021; 130:27–40. 33452566 10.1007/s00412-020-00750-9 PMC7889546

[3] Greenbaum J.A. , Pang B., Tullius T.D. Construction of a genome-scale structural map at single-nucleotide resolution. Genome Res. 2007; 17:947–953. 17568010 10.1101/gr.6073107 PMC1891353

[4] Raghavan S.C. , Chastain P., Lee J.S., Hegde B.G., Houston S., Langen R., Hsieh C.-L., Haworth I.S., Lieber M.R. Evidence for a triplex DNA conformation at the bcl-2 major breakpoint region of the t(14;18) translocation. J. Biol. Chem. 2005; 280:22749–22760. 15840562 10.1074/jbc.M502952200

[5] Gough G.W. , Sullivan K.M., Lilley D.M. The structure of cruciforms in supercoiled DNA: probing the single-stranded character of nucleotide bases with bisulphite. EMBO J. 1986; 5:191–196. 3007115 10.1002/j.1460-2075.1986.tb04195.x PMC1166713

[6] Nambiar M. , Goldsmith G., Moorthy B.T., Lieber M.R., Joshi M.V., Choudhary B., Hosur R.V., Raghavan S.C. Formation of a G-quadruplex at the BCL2 major breakpoint region of the t(14;18) translocation in follicular lymphoma. Nucleic Acids Res. 2011; 39:936–948. 20880994 10.1093/nar/gkq824 PMC3035451

[7] Dumelie J.G. , Jaffrey S.R. Defining the location of promoter-associated R-loops at near-nucleotide resolution using bisDRIP-seq. Elife. 2017; 6:e28306. 29072160 10.7554/eLife.28306 PMC5705216

[8] Tsai A.G. , Engelhart A.E., Hatmal M.M., Houston S.I., Hud N.V., Haworth I.S., Lieber M.R. Conformational variants of duplex DNA correlated with cytosine-rich chromosomal fragile sites. J. Biol. Chem. 2009; 284:7157–7164. 19106104 10.1074/jbc.M806866200 PMC2652318

[9] Brukner I. , Dlakic M., Savic A., Susic S., Pongor S., Suck D. Evidence for opposite groove-directed curvature of GGGCCC and AAAAA sequence elements. Nucleic Acids Res. 1993; 21:1025–1029. 8451169 10.1093/nar/21.4.1025 PMC309239

[10] Dornberger U. , Leijon M., Fritzsche H. High base pair opening rates in tracts of GC base pairs. J. Biol. Chem. 1999; 274:6957–6962. 10066749 10.1074/jbc.274.11.6957

[11] Dornberger U. , Spackovj N., Walter A., Gollmick F.A., Sponer J., Fritzsche H. Solution structure of the dodecamer d-(CATGGGCC-CATG)2 is B-DNA. Experimental and molecular dynamics study. J. Biomol. Struct. Dyn. 2001; 19:159–174. 11565847 10.1080/07391102.2001.10506728

[12] Fogg J.M. , Judge A.K., Stricker E., Chan H.L., Zechiedrich L. Supercoiling and looping promote DNA base accessibility and coordination among distant sites. Nat. Commun. 2021; 12:5683. 34584096 10.1038/s41467-021-25936-2 PMC8478907

[13] Geggier S. , Vologodskii A. Sequence dependence of DNA bending rigidity. Proc. Natl. Acad. Sci. U.S.A. 2010; 107:15421–15426. 20702767 10.1073/pnas.1004809107 PMC2932579

[14] Stefl R. , Wu H., Ravindranathan S., Sklenár V., Feigon J. DNA A-tract bending in three dimensions: solving the dA4T4 vs. dT4A4 conundrum. Proc. Natl. Acad. Sci. U.S.A. 2004; 101:1177–1182. 14739342 10.1073/pnas.0308143100 PMC337026

[15] Hizver J. , Rozenberg H., Frolow F., Rabinovich D., Shakked Z. DNA bending by an adenine–thymine tract and its role in gene regulation. Proc. Natl. Acad. Sci. U.S.A. 2001; 98:8490–8495. 11438706 10.1073/pnas.151247298 PMC37463

[16] Růžička M. , Souček P., Kulhánek P., Radová L., Fajkusová L., Réblová K. Bending of DNA duplexes with mutation motifs. DNA Res. 2019; 26:341–352. 31230075 10.1093/dnares/dsz013 PMC6704406

[17] Bacolla A. , Zhu X., Chen H., Howells K., Cooper D.N., Vasquez K.M. Local DNA dynamics shape mutational patterns of mononucleotide repeats in human genomes. Nucleic Acids Res. 2015; 43:5065–5080. 25897114 10.1093/nar/gkv364 PMC4446427

[18] Shen J.-C. , Rideout William M. I.I.I., Jones P.A. The rate of hydrolytic deamination of 5-methylcytosine in double-stranded DNA. Nucleic Acids Res. 1994; 22:972–976. 8152929 10.1093/nar/22.6.972 PMC307917

[19] Khanna A. , Larson D.E., Srivatsan S.N., Mosior M., Abbott T.E., Kiwala S., Ley T.J., Duncavage E.J., Walter M.J., Walker J.R. et al. Bam-readcount – rapid generation of basepair-resolution sequence metrics. JOSS. 2022; 7:3722. 10.21105/joss.03722 PMC13367885 42453900

[20] Basu A. , Bobrovnikov D.G., Qureshi Z., Kayikcioglu T., Ngo T.T.M., Ranjan A., Eustermann S., Cieza B., Morgan M.T., Hejna M. et al. Measuring DNA mechanics on the genome scale. Nature. 2021; 589:462–467. 33328628 10.1038/s41586-020-03052-3 PMC7855230

[21] Cui F. , Zhurkin V.B. Structure-based analysis of DNA sequence patterns guiding nucleosome positioning in vitro. J. Biomol. Struct. Dyn. 2010; 27:821–841. 20232936 10.1080/073911010010524947 PMC2993692

[22] Basham B. , Schroth G.P., Ho P.S. An A-DNA triplet code: thermodynamic rules for predicting A- and B-DNA. Proc. Natl. Acad. Sci. U.mS.A. 1995; 92:6464–6468. 10.1073/pnas.92.14.6464 PMC41538 7604014

[23] Tolstorukov M.Y. , Ivanov V.I., Malenkov G.G., Jernigan R.L., Zhurkin V.B. Sequence-dependent B↔A transition in DNA evaluated with dimeric and trimeric scales. Biophys. J. 2001; 81:3409–3421. 11721003 10.1016/S0006-3495(01)75973-5 PMC1301797

[24] Fujii S. , Kono H., Takenaka S., Go N., Sarai A. Sequence-dependent DNA deformability studied using molecular dynamics simulations. Nucleic Acids Res. 2007; 35:6063–6074. 17766249 10.1093/nar/gkm627 PMC2094071

[25] Dixit S.B. , Beveridge D.L., Case D.A., Cheatham T.E., Giudice E., Lankas F., Lavery R., Maddocks J.H., Osman R., Sklenar H. et al. Molecular dynamics simulations of the 136 unique tetranucleotide sequences of DNA oligonucleotides. II: sequence context effects on the dynamical structures of the 10 unique dinucleotide steps. Biophys. J. 2005; 89:3721–3740. 16169978 10.1529/biophysj.105.067397 PMC1366942

[26] Lavery R. , Zakrzewska K., Beveridge D., Bishop T.C., Case D.A., Cheatham T. 3rd, Dixit S., Jayaram B., Lankas F., Laughton C. et al. A systematic molecular dynamics study of nearest-neighbor effects on base pair and base pair step conformations and fluctuations in B-DNA. Nucleic Acids Res. 2010; 38:299–313. 19850719 10.1093/nar/gkp834 PMC2800215

[27] Beveridge D.L. , Cheatham T.E. 3rd, Mezei M. The ABCs of molecular dynamics simulations on B-DNA, circa 2012. J. Biosci. 2012; 37:379–397. 22750978 10.1007/s12038-012-9222-6 PMC4029509

[28] Satchwell S.C. , Drew H.R., Travers A.A. Sequence periodicities in chicken nucleosome core DNA. J. Mol. Biol. 1986; 191:659–675. 3806678 10.1016/0022-2836(86)90452-3

[29] Barbier J. , Vaillant C., Volff J.-N., Brunet F.G., Audit B. Coupling between sequence-mediated nucleosome organization and genome evolution. Genes (Basel). 2021; 12:851. 34205881 10.3390/genes12060851 PMC8228248

[30] Goodsell D.S. , Kaczor-Grzeskowiak M., Dickerson R.E. The crystal structure of C-C-A-T-T-A-A-T-G-G. Implications for bending of B-DNA at T-A steps. J. Mol. Biol. 1994; 239:79–96. 8196049 10.1006/jmbi.1994.1352

[31] Kessler M.D. , Loesch D.P., Perry J.A., Heard-Costa N.L., Taliun D., Cade B.E., Wang H., Daya M., Ziniti J., Datta S. et al. De novo mutations across 1,465 diverse genomes reveal mutational insights and reductions in the Amish founder population. Proc. Natl. Acad. Sci. U.S.A. 2020; 117:2560–2569. 31964835 10.1073/pnas.1902766117 PMC7007577

[32] Li C. , Luscombe N.M. Nucleosome positioning stability is a modulator of germline mutation rate variation across the human genome. Nat. Commun. 2020; 11:1363. 32170069 10.1038/s41467-020-15185-0 PMC7070026

[33] Pich O. , Muiños F., Sabarinathan R., Reyes-Salazar I., Gonzalez-Perez A., Lopez-Bigas N. Somatic and germline mutation periodicity follow the orientation of the DNA minor groove around nucleosomes. Cell. 2018; 175:1074–1087. 30388444 10.1016/j.cell.2018.10.004

[34] Dickerson R.E. , Goodsell D., Kopka M.L. MPD and DNA bending in crystals and in solution. J. Mol. Biol. 1996; 256:108–125. 8609604 10.1006/jmbi.1996.0071

[35] Pulleyblank D.E. , Haniford D.B., Morgan A.R. A structural basis for S1 nuclease sensitivity of double-stranded DNA. Cell. 1985; 42:271–280. 2990727 10.1016/s0092-8674(85)80122-7

[36] Varani G. , McClain W.H. The G x U wobble base pair. A fundamental building block of RNA structure crucial to RNA function in diverse biological systems. EMBO Rep. 2000; 1:18–23. 11256617 10.1093/embo-reports/kvd001 PMC1083677

[37] Ohyama T. New aspects of magnesium function: a key regulator in nucleosome self-assembly, chromatin folding and phase separation. Int. J. Mol. Sci. 2019; 20:4232. 31470631 10.3390/ijms20174232 PMC6747271

[38] Guéroult M. , Boittin O., Mauffret O., Etchebest C., Hartmann B. Mg2+ in the major groove modulates B-DNA structure and dynamics. PLoS One. 2012; 7:e41704. 22844516 10.1371/journal.pone.0041704 PMC3402463

[39] Denisov V.P. , Halle B. Sequence-specific binding of counterions to B-DNA. Proc. Natl. Acad. Sci. U.S.A. 2000; 97:629–633. 10639130 10.1073/pnas.97.2.629 PMC15381

[40] Zhou T. , Yang L., Lu Y., Dror I., Dantas Machado A.C., Ghane T., Di Felice R., Rohs R. DNAshape: a method for the high-throughput prediction of DNA structural features on a genomic scale. Nucleic Acids Res. 2013; 41:W56–W62. 23703209 10.1093/nar/gkt437 PMC3692085

[41] Chiu T.-P. , Yang L., Zhou T., Main B.J., Parker S.C.J., Nuzhdin S.V., Tullius T.D., Rohs R. GBshape: a genome browser database for DNA shape annotations. Nucleic Acids Res. 2015; 43:D103–D109. 25326329 10.1093/nar/gku977 PMC4384032

