# Supplementary material for: Bisulfite probing reveals DNA structural intricacies
Source: Nucleic Acids Res. 2023 Mar 7;51(7):3261–9. doi: 10.1093/nar/gkad115 (PMC10123088; doi:10.1093/nar/gkad115)
Supplement: gkad115_Supplemental_Files [file gkad115_supplemental_files.zip › Supplementary_figures.pdf]

Supplementary figures

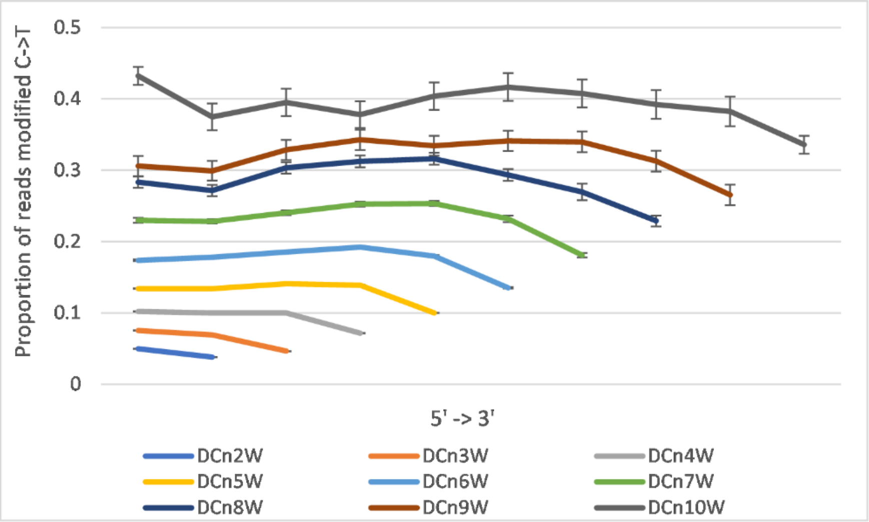

Figure S1: The polar reactivity gradient at poly-C based on the data from Dumelie and Jaffrey (1). Error bars are ± 1 SEM.

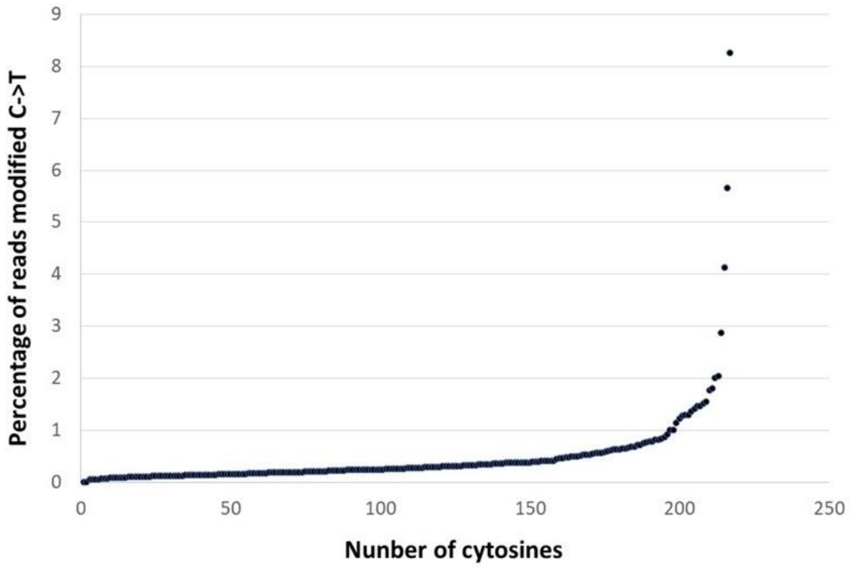

Figure S2: Bisulfite reactivity of all CpG dinucleotides in the data generated for this study.

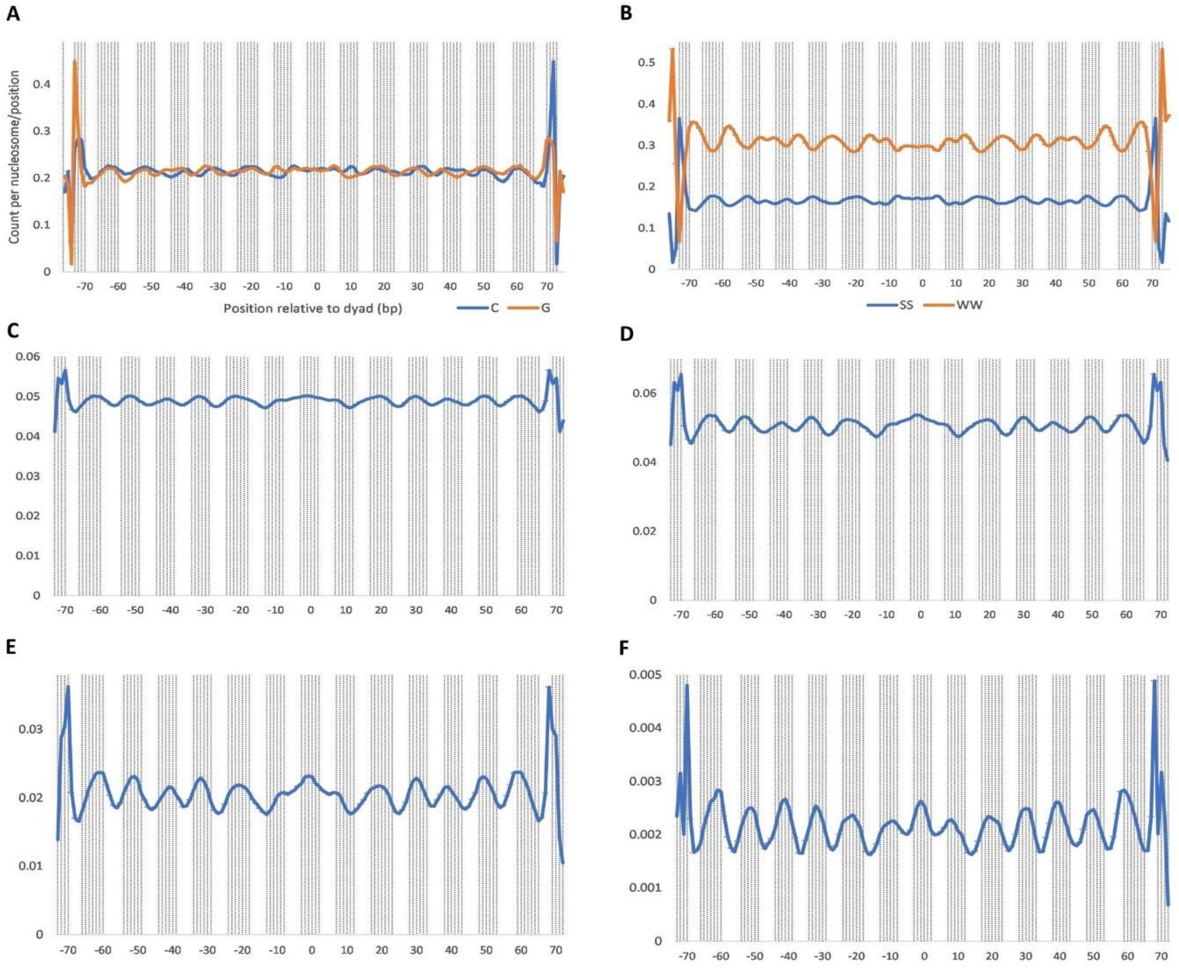

Figure S3: Other associations with nucleosome phasing. Differences in base composition (A) and dinucleotide content (B) between areas of positive-roll bending (vertical dotted lines, based on data from x-ray crystal structures (2)) and negative-roll bending (white space) in the nucleosome have been shown previously (reviewed in (3)). Point zero on the x-axes represents the dyad position, i. e. the point of internal symmetry of the nucleosome. Mean bisulfite modification per cytosine analyzed in the study by Dumelie and Jaffrey(1) correlates weakly with bending overall, based on a 7-bp sliding window (C), but this increases with increasing strength of nucleosome positioning, e. g. dyad positions indicated by more than five reads at a single position are more strongly correlated with reactivity (D). This increases further for the most bisulfite-reactive heptamers, e. g. the top 1000 (E), and the top 50 (F).

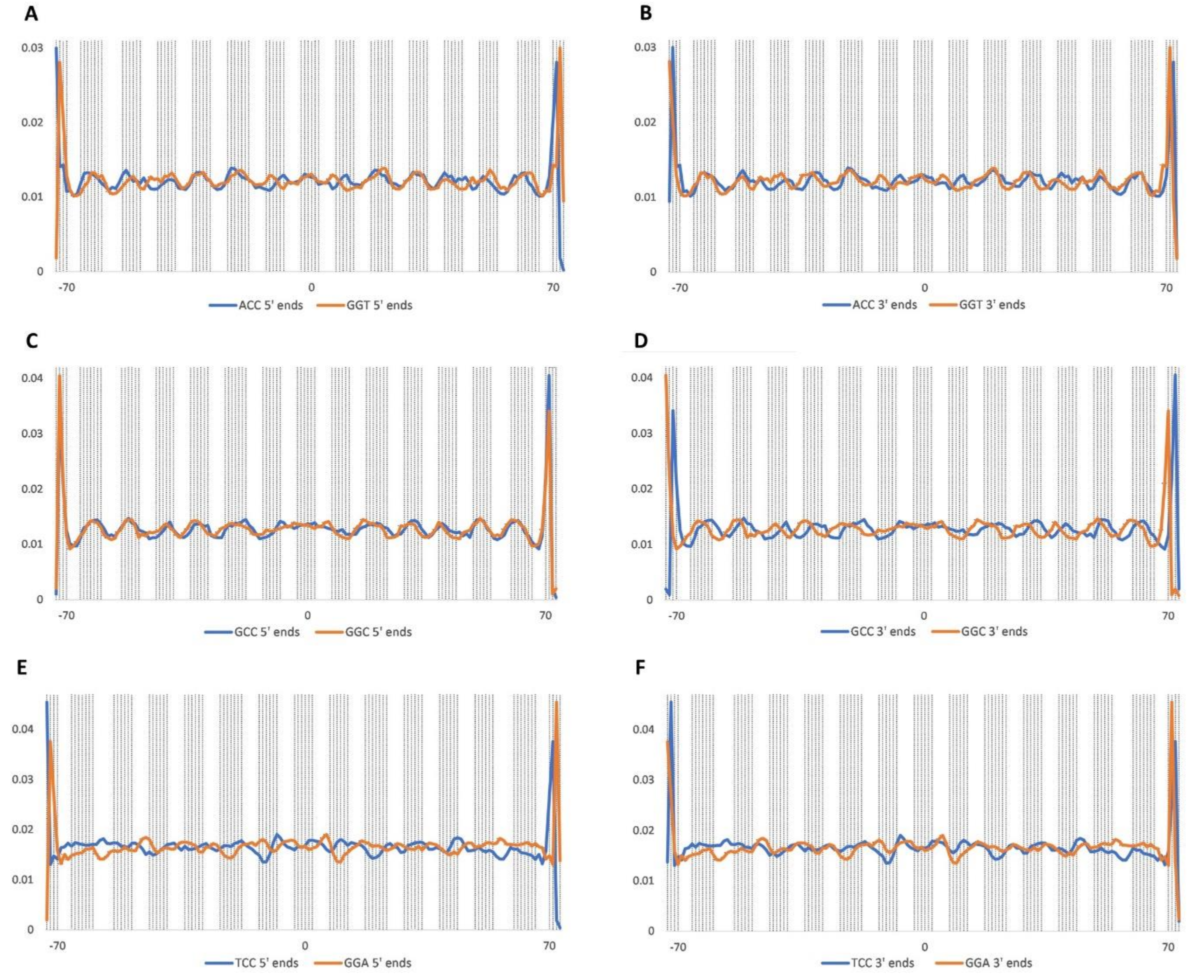

Figure S4: Comparison of the locations of the 5’ ends of poly-C of length 2 bp (A, C, E) with their 3’ ends (B, D, F) relative to nucleosome positioning. Vertical dotted lines indicate areas with positive-roll bending when bound to nucleosomes, and the intervening white space corresponds to negative-roll bending (2). Point zero on the x-axes represents the dyad position, i. e. the point of internal symmetry of the nucleosome.

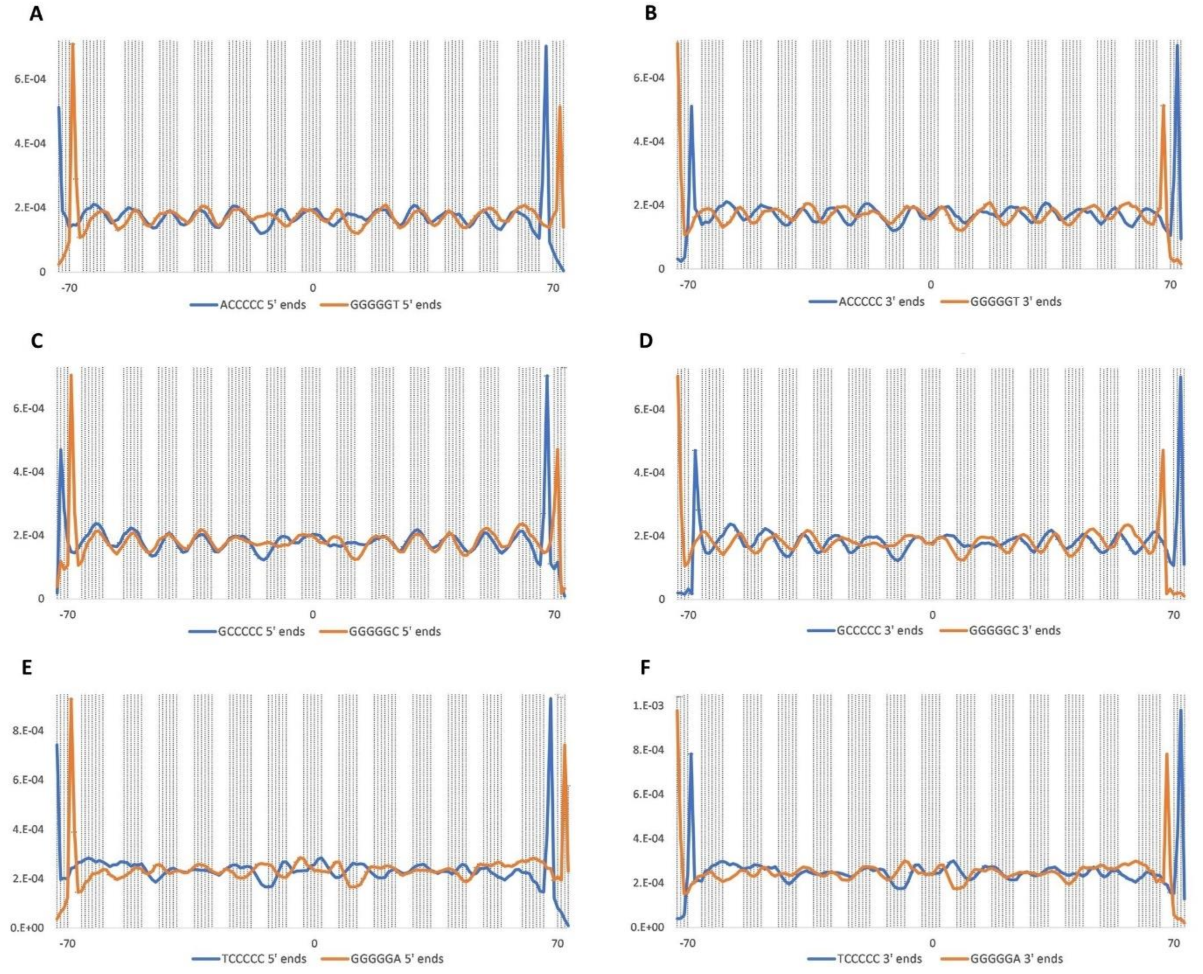

Figure S5: Comparison of the locations of the 5’ ends of poly-C of length 5 bp (A, C, E) with their 3’ ends (B, D, F) relative to nucleosome positioning. Vertical dotted lines indicate areas with positive-roll bending when bound to nucleosomes, and the intervening white space corresponds to negative-roll bending (2). Point zero on the x-axes represents the dyad position, i. e. the point of internal symmetry of the nucleosome.

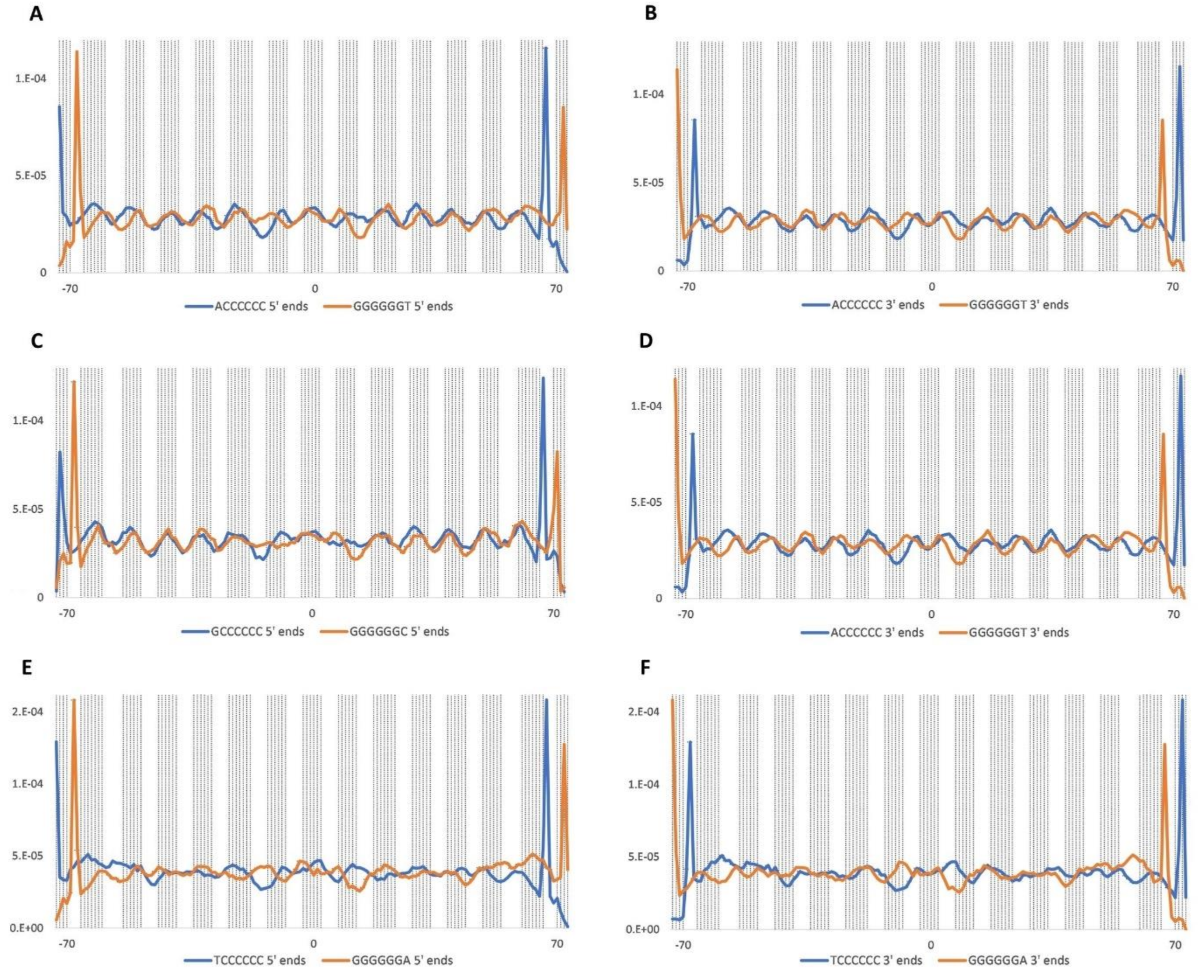

Figure S6: Comparison of the locations of the 5’ ends of poly-C of length 6 bp (A, C, E) with their 3’ ends (B, D, F) relative to nucleosome positioning. Vertical dotted lines indicate areas with positive-roll bending when bound to nucleosomes, and the intervening white space corresponds to negative-roll bending (2). Point zero on the x-axes represents the dyad position, i. e. the point of internal symmetry of the nucleosome.

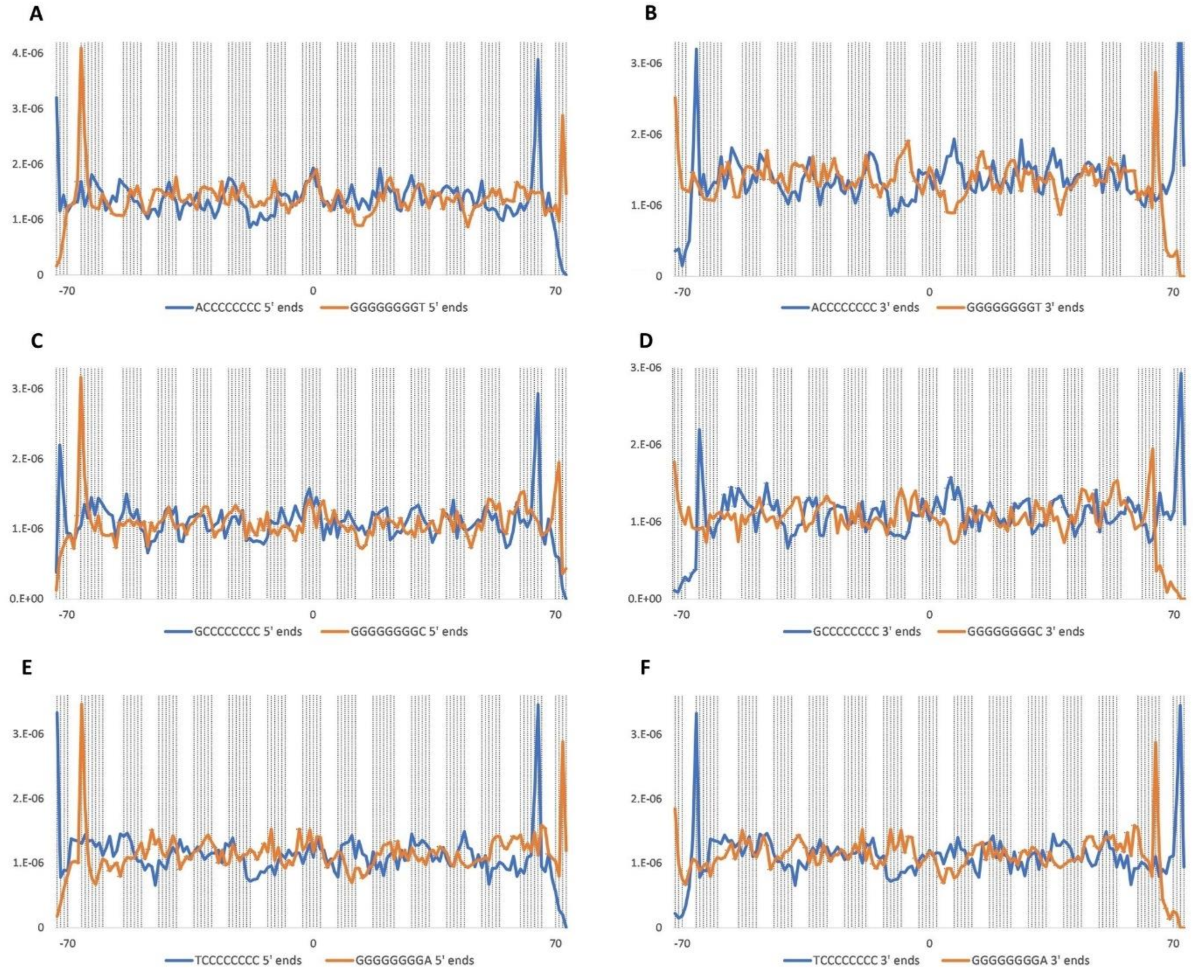

Figure S7: Comparison of the locations of the 5’ ends of poly-C of length 8 bp (A, C, E) with their 3’ ends (B, D, F) relative to nucleosome positioning. Vertical dotted lines indicate areas with positive-roll bending when bound to nucleosomes, and the intervening white space corresponds to negative-roll bending (2). Point zero on the x-axes represents the dyad position, i. e. the point of internal symmetry of the nucleosome.

1. Dumelie,J.G. and Jaffrey,S.R. (2017) Defining the location of promoter-associated R-loops at near-nucleotide resolution using bisDRIP-seq. *Elife*, **6**, e28306.

2. Liu,G., Zhao,H., Meng,H., Xing,Y. and Cai,L. (2021) A deformation energy model reveals sequence-dependent property of nucleosome positioning. *Chromosoma*, **130**, 27–40.

3. Barbier,J., Vaillant,C., Volff,J.-N., Brunet,F.G. and Audit,B. (2021) Coupling between Sequence-Mediated Nucleosome Organization and Genome Evolution. *Genes (Basel).*, **12**, 851.
