# Supplementary material for: Bisulfite probing reveals DNA structural intricacies
Source: Nucleic Acids Res. 2023 Mar 7;51(7):3261–9. doi: 10.1093/nar/gkad115 (PMC10123088; doi:10.1093/nar/gkad115)
Supplement: gkad115_Supplemental_Files [file gkad115_supplemental_files.zip › Supplementary_table_legends.docx]

**Table S1:** Sequences of degenerate oligonucleotides and corresponding reference sequences for each of the primer pairs used for PCR amplification of bisulfite-modified genomic DNA.

**Table S2:** Levels of bisulfite reactivity (percentage of reads modified C->T) and error (other modifications) for each base of the ~19 kb from human Chromosome 21 probed by bisulfite and high-throughput sequencing for this study.

**Table S3:** Bisfulite reactivity (percentage of reads modified C->T) for the central (fourth) nucleotides of all possible heptamers with a G or C at their fourth position, based on the data from Dumelie and Jaffrey1. Heptamers modified in more than 40% of reads were excluded in view of the possibility of heterozygosity.

**Table S4:** Statistical models (generalized linear; quasibinomial family in R) showing the effects of 5'-neighbouring A-tracts on reactivity at DCD, DCCD and DCCCD. These were significant when controlling for the possible confounding effects of local AT-content (number of A:T base pairs within 8 bp) and 5' T residues. Only 5’ C’s were considered for DCCD and DCCCD. "Minus" indicates position to the 5' side of the 5' C.

**Supplementary references**

1. Kessler,M.D., Loesch,D.P., Perry,J.A., Heard-Costa,N.L., Taliun,D., Cade,B.E., Wang,H., Daya,M., Ziniti,J., Datta,S., *et al.* (2020) De novo mutations across 1,465 diverse genomes reveal mutational insights and reductions in the Amish founder population. *Proc. Natl. Acad. Sci. U. S. A.*, **117**, 2560–2569.
