# Supplementary material for: Bisulfite probing reveals DNA structural intricacies
Source: Nucleic Acids Res. 2023 Mar 7;51(7):3261–9. doi: 10.1093/nar/gkad115 (PMC10123088; doi:10.1093/nar/gkad115)
Supplement: gkad115_Supplemental_Files [file gkad115_supplemental_files.zip › Table S2.docx]

**Table S2:** Levels of bisulfite reactivity (percentage of reads modified C->T) and error (other modifications) for each base of the ~19 kb from human Chromosome 21 probed by bisulfite and high-throughput sequencing for this study.

**Ref base %Reads modified C>T %Non C>T changes**

G 4.815864023 0.424929178

A 0 0

C 5.650459921 0.262812089

G 0.258064516 7.483870968

C 0.626566416 8.897243108

T 0 0

G 1.231802912 1.791713326

G 3.474484256 0.868621064

T 0 0

T 0 0

T 0 0

G 2.577319588 6.786941581

G 9.960159363 0.3187251

T 0 0

A 0 0

G 16.1849711 0.361271676

C 8.480325645 4.47761194

C 6.380120887 2.41773002

C 13.60366252 0.196206671

C 4.528061224 1.147959184

T 0 0

G 3.897685749 0.182704019

T 0 0

G 3.405017921 1.314217443

G 5.49211528 0.108754758

T 0 0

G 2.423603793 0.105374078

T 0 0

T 0 0

A 0 0

C 3.341176471 0.235294118

A 0 0

G 3.885291397 0.046253469

G 7.123161765 0.091911765

A 0 0

G 6.370569762 0.179452669

T 0 0

A 0 0

C 3.090988246 1.001306051

T 0 0

T 0 0

G 1.924721985 0.213857998

G 3.664252237 0.042607584

C 2.677433064 0.042498938

A 0 0

A 0 0

G 1.374427322 0.624739692

A 0 0

C 0.369458128 0.862068966

A 0 0

G 1.13682501 0.852618758

C 0.725806452 1.411290323

A 0 0

G 1.048543689 0

T 0 0

T 0 0

T 0 0

C 4.611379695 0.520639643

C 5.241786637 0.812107789

C 4.231052244 0.367917586

C 0.109729334 0.585223116

G 0.250357654 0.143061516

G 1.374207188 0.070472163

G 10.29923452 0.243562978

T 0 0

A 0 0

C 6.010186757 0.13582343

A 0 0

C 7.520053476 2.072192513

C 1.53078203 0.798668885

C 0.557194363 1.081612586

G 0.096899225 0.452196382

C 0.70535428 1.025969862

T 0 0

A 0 0

G 1.516108654 1.010739103

G 5.481158518 0.467144192

T 0 0

G 0.552147239 0

T 0 0

T 0 0

T 0 0

T 0 0

G 1.544739143 0

T 0 0

A 0 0

G 1.622247972 1.245654693

C 1.216333623 1.274254272

A 0 0

G 0.769011678 0.056963828

A 0 0

G 1.864218141 0.111296605

T 0 0

G 1.995012469 0.221668052

C 2.599557522 0.442477876

A 0 0

T 0 0

C 0.349368449 1.182477829

T 0 0

G 1.428949458 0.026462027

T 0 0

G 0.790722193 0.184501845

A 0 0

G 1.203873332 0.104684638

A 0 0

C 0.907205806 0.311041991

A 0 0

A 0 0

C 0.756429652 0.353000504

T 0 0

G 1.127819549 0.125313283

G 4.423894026 0.074981255

T 0 0

G 0.498753117 0.174563591

A 0 0

A 0 0

C 5.156172533 0.347050074

T 0 0

G 1.223391241 0.04893565

C 1.05006105 0.244200244

T 0 0

T 0 0

T 0 0

C 3.358925144 0.143953935

C 1.602870813 0.071770335

T 0 0

T 0 0

G 0.757575758 0.426136364

C 1.109799292 0.141676505

A 0 0

A 0 0

C 4.464703132 0.140252454

C 0.789779326 0.209059233

T 0 0

A 0 0

G 1.589495508 0.046072334

A 0 0

G 3.060989643 0.069044879

G 6.586826347 0

G 7.509790371 0.207325501

C 3.798342541 0.046040516

A 0 0

G 0.299056821 0.046008742

A 0 0

T 0 0

T 0 0

T 0 0

C 0.159416989 0.523798679

T 0 0

G 0.34121929 0.045495905

G 1.930063579 0.227066303

C 2.065365411 0.06808897

A 0 0

A 0 0

G 1.022676745 0.222321032

T 0 0

T 0 0

C 1.496808276 0.088047546

C 0.635686103 0.02192021

A 0 0

G 1.17929679 0.043677659

A 0 0

G 2.664337192 0.152871806

G 4.806641905 0.436967446

G 10.00218866 0.043773255

T 0 0

G 0.983606557 0.06557377

G 1.198779425 0.414123801

A 0 0

T 0 0

T 0 0

C 0.924134967 0.236406619

C 0.622986037 0.236305048

A 0 0

A 0 0

A 0 0

C 3.955763505 0.042535091

A 0 0

T 0 0

G 4.151738672 0.18967334

T 0 0

T 0 0

C 4.534161491 0.082815735

C 1.571546733 0.124069479

T 0 0

C 0.764620789 0.103327134

T 0 0

A 0 0

A 0 0

T 0 0

G 2.282541641 0.041126876

T 0 0

G 0.948258091 0.061842919

G 1.437076576 0.57483063

A 0 0

T 0 0

C 0.676229508 0.409836066

T 0 0

G 0.75172694 0.101584722

C 1.19772635 0.223304913

A 0 0

G 0.95007075 0.262785527

T 0 0

G 0.772043885 0.284437221

A 0 0

T 0 0

G 1.540020263 0

T 0 0

C 1.151515152 0.565656566

T 0 0

C 1.062124248 0.300601202

T 0 0

G 1.375872383 0.239282154

C 2.892479553 0.538599641

C 1.597763132 0.319552626

A 0 0

T 0 0

T 0 0

C 0.455716267 0.138696255

A 0 0

G 1.272513704 0.274079875

T 0 0

G 2.602447077 0.194212468

G 4.256559767 0.252672498

G 6.818181818 0.097125097

C 8.841818888 0.077730276

A 0 0

T 0 0

A 0 0

G 3.084384093 0.407371484

C 2.994943602 0.661221315

T 0 0

G 1.902017291 0.172910663

T 0 0

G 4.9922899 0.134926754

C 7.159353349 0.153964588

C 7.678916828 0.193423598

C 3.641618497 0.096339114

T 0 0

T 0 0

T 0 0

T 0 0

A 0 0

G 1.555209953 0.563763608

T 0 0

G 1.773187841 1.013250195

G 3.473170732 1.151219512

G 4.673807205 1.304771178

G 7.87796872 0.926819849

T 0 0

C 2.119868954 0.770861438

T 0 0

A 0 0

G 0.582524272 1.242718447

A 0 0

T 0 0

C 0.545915383 0.428933515

T 0 0

C 0.233781414 0.331190337

A 0 0

G 6.179992275 0.096562379

C 7.399536321 0.231839258

C 4.125508425 0.116211505

C 2.806270563 0.290303851

T 0 0

G 1.259689922 0.23255814

G 0 0

G 2.452793459 0.214132762

A 0 0

T 0 0

T 0 0

G 2.660313679 0.258090133

G 4.436927975 0.238758456

G 7.508464449 1.473809998

G 8.303393214 0.379241517

G 7.706164932 0.260208167

C 9.65738329 1.1420557

A 0 0

T 0 0

T 0 0

T 0 0

T 0 0

C 0.445524504 0.141757797

T 0 0

C 0.202101859 0.242522231

A 0 0

G 0.607410407 0.425187285

T 0 0

T 0 0

G 1.704891415 0.202963264

C 1.441039172 0.182666937

T 0 0

C 0.613496933 0.36809816

A 0 0

A 0 0

T 0 0

C 0.746419205 0.766592697

T 0 0

C 0.559888022 0.539892022

A 0 0

G 6.906729634 0.157418339

C 8.944999021 0.606772362

C 5.834464043 0.290754022

C 4.163498099 0.266159696

T 0 0

A 0 0

G 3.989411987 0.605029306

G 8.369709622 0.265705067

G 7.523809524 0.152380952

G 10.02115791 0.403923832

C 7.065845206 0.3658067

A 0 0

G 1.239349342 0.271107668

T 0 0

G 1.07100357 0.694168981

A 0 0

C 3.602161297 0.680408245

C 4.09556314 0.642441277

A 0 0

T 0 0

T 0 0

C 3.035714286 0.674603175

C 2.063714686 0.360649169

T 0 0

T 0 0

A 0 0

T 0 0

A 0 0

T 0 0

A 0 0

T 0 0

G 13.94605395 0.41958042

G 6.815910454 0.279832101

T 0 0

T 0 0

G 0.684793555 0.422960725

T 0 0

T 0 0

T 0 0

G 1.929788544 0.349004311

T 0 0

A 0 0

T 0 0

A 0 0

T 0 0

T 0 0

C 0.340749649 0.180396873

T 0 0

T 0 0

T 0 0

G 0.483189048 0.342258909

G 1.110213969 0.282599919

A 0 0

A 0 0

C 1.009285426 0.363342753

T 0 0

G 0.621740874 0.120336943

T 0 0

C 0.84354288 0.100421771

T 0 0

T 0 0

G 0 0

A 0 0

T 0 0

T 0 0

T 0 0

T 0 0

A 0 0

T 0 0

T 0 0

A 0 0

C 1.415752742 0.219341974

T 0 0

G 0.756369427 0.179140127

T 0 0

T 0 0

A 0 0

G 1.467380527 0.1189768

T 0 0

C 1.153081511 0.238568588

T 0 0

C 0.808040993 0.137958218

T 0 0

C 1.039623382 0.196155355

A 0 0

T 0 0

T 0 0

A 0 0

C 4.72921434 0.419527079

T 0 0

C 2.631578947 0.381388253

C 5.518298525 0.766430351

A 0 0

C 1.991276313 0.531007017

T 0 0

C 6.142963515 0.18615041

C 7.305255311 0.223630265

C 2.942271881 0.130353818

T 0 0

T 0 0

A 0 0

T 0 0

T 0 0

A 0 0

T 0 0

A 0 0

G 1.345716452 0.227445034

T 0 0

A 0 0

A 0 0

A 0 0

T 0 0

G 0.72769054 0.095748755

A 0 0

T 0 0

T 0 0

C 0.513210416 0.228093518

T 0 0

T 0 0

T 0 0

G 0.639819345 0.112909296

T 0 0

A 0 0

T 0 0

T 0 0

T 0 0

G 0.465029762 0.093005952

A 0 0

C 0.553812073 0.276906037

T 0 0

T 0 0

T 0 0

C 3.840553331 0.236621769

C 4.332787184 0.473329692

C 1.542089985 0.526124819

T 0 0

G 1.064981949 0.306859206

G 3.050727208 0.141894289

T 0 0

C 0.564573042 0.211714891

A 0 0

A 0 0

G 1.684801685 0.105300105

G 8.787193318 0.087001914

T 0 0

A 0 0

C 3.723681921 0.156603445

T 0 0

A 0 0

A 0 0

G 1.050420168 0.175070028

T 0 0

G 0.655679603 0.230373915

G 2.19042572 0.3003003

T 0 0

T 0 0

T 0 0

T 0 0

T 0 0

C 0.67861493 0.278406125

T 0 0

C 0.919819507 0.06942034

T 0 0

C 3.075859686 0.069120442

C 1.415501467 0.51786639

T 0 0

G 0.582191781 0.119863014

T 0 0

T 0 0

T 0 0

G 1.045594789 0.18854988

G 1.107891597 0.630645986

A 0 0

T 0 0

C 0.46217049 0.119821979

T 0 0

A 0 0

G 0.357812234 0.187425456

A 0 0

T 0 0

T 0 0

G 0.446505238 0.343465568

A 0 0

T 0 0

A 0 0

C 1.215337213 0.239643958

A 0 0

A 0 0

T 0 0

A 0 0

A 0 0

T 0 0

T 0 0

T 0 0

T 0 0

T 0 0

T 0 0

G 0.679463224 0.101919484

C 0.643086817 0.067693349

A 0 0

A 0 0

T 0 0

T 0 0

A 0 0

T 0 0

G 1.57258732 0.281410363

A 0 0

G 1.901769472 0

C 1.622418879 0.098328417

A 0 0

A 0 0

T 0 0

G 5.922220429 0.38728417

C 2.609536082 0.209407216

T 0 0

G 1.274494185 0.159311773

C 1.464268661 0.12732771

T 0 0

G 1.126626468 0.031735957

T 0 0

G 0.995664044 0.06423639

A 0 0

A 0 0

C 3.713826367 0.160771704

A 0 0

T 0 0

T 0 0

C 0.350485901 0.127449419

T 0 0

T 0 0

G 0.429799427 0.429799427

A 0 0

T 0 0

C 0.624 0.096

A 0 0

T 0 0

G 2.13844448 0.269285601

T 0 0

C 1.262227832 0.268223414

T 0 0

T 0 0

A 0 0

G 1.107298815 0.140361822

T 0 0

G 1.678694697 0.502039536

G 2.644836272 0.267632242

A 0 0

C 11.52574398 0.409384349

A 0 0

T 0 0

A 0 0

A 0 0

G 1.964512041 0.031685678

C 2.011403231 0.459296801

A 0 0

A 0 0

T 0 0

A 0 0

A 0 0

T 0 0

T 0 0

G 1.371158392 0.48857368

C 1.052300927 0.251295744

T 0 0

G 1.249421564 0.354774024

C 1.248266297 0.231160425

T 0 0

G 1.606487148 0.183598531

G 2.808040509 0.721190732

G 6.560121766 0.060882801

T 0 0

C 1.348893604 0.303122158

T 0 0

G 0.892990028 0.059532669

T 0 0

G 1.729234407 0.266036063

T 0 0

G 1.451851852 0.118518519

C 1.317347543 0.044404973

A 0 0

G 1.139053254 0.355029586

G 2.964719834 0.088941595

A 0 0

A 0 0

T 0 0

G 0.486988282 0.380459595

A 0 0

T 0 0

T 0 0

T 0 0

G 1.092398726 0.364132909

C 1.151166313 0.46955468

T 0 0

A 0 0

G 0.576368876 0.546033672

A 0 0

T 0 0

C 0.952524947 0.347747203

A 0 0

T 0 0

A 0 0

G 2.041124887 0.120955549

C 1.8999848 0.167198662

A 0 0

C 4.248815528 0.275103164

A 0 0

T 0 0

A 0 0

C 0.352112676 0.137783221

G 0.398956575 0.199478288

T 0 0

T 0 0

T 0 0

A 0 0

T 0 0

C 0.413666309 0.352382412

T 0 0

G 1.868300153 0.245022971

T 0 0

A 0 0

G 1.458173446 0.414428243

C 1.551936079 0.645359557

A 0 0

G 0.262143408 0.092521203

A 0 0

A 0 0

A 0 0

C 0.578848561 0.688360451

T 0 0

G 1.15157174 0.031123561

C 2.65086543 0.826446281

C 1.061173533 0.093632959

A 0 0

A 0 0

G 1.506788005 0.23869909

G 8.002980626 0.029806259

T 0 0

A 0 0

T 0 0

A 0 0

T 0 0

A 0 0

C 1.787043931 0.059568131

A 0 0

G 3.264417845 0.528524794

C 2.338185197 0.232270053

T 0 0

T 0 0

T 0 0

C 1.54735855 0.140668959

C 0.733458177 0.29650437

A 0 0

A 0 0

A 0 0

G 0.790638836 0.284629981

T 0 0

G 0.779882222 0.732134331

T 0 0

G 0.636031166 0.795038957

T 0 0

T 0 0

A 0 0

A 0 0

T 0 0

T 0 0

T 0 0

A 0 0

C 2.235256817 0.063411541

A 0 0

T 0 0

G 5.022321429 0.047831633

C 6.611570248 0.047679593

C 1.766390834 0.159134309

T 0 0

G 2.330004755 0.174354097

C 2.167378579 0.158202816

T 0 0

A 0 0

G 1.405115251 0.142090306

C 2.365930599 0.835962145

A 0 0

A 0 0

G 1.652762474 0.18888714

G 8.301827347 0.236294896

T 0 0

A 0 0

G 2.285714286 0.063492063

G 4.625376208 0.110882306

A 0 0

G 0.595429675 0.160926939

A 0 0

A 0 0

A 0 0

T 0 0

A 0 0

C 1.090368412 0.198248802

A 0 0

G 1.065601066 0.34965035

T 0 0

T 0 0

G 1.678298438 0.48188767

C 1.489572989 0.033101622

T 0 0

T 0 0

C 4.612090265 0.164717509

C 3.409090909 0.312911726

C 2.304147465 0.181040158

A 0 0

A 0 0

G 1.608139153 0.065638333

T 0 0

A 0 0

G 2.427745665 0.511973576

C 2.951360264 0.461665293

T 0 0

G 0.805656034 0.378165077

G 1.918032787 0.393442623

G 1.689408707 0.552306693

A 0 0

T 0 0

T 0 0

A 0 0

C 1.592977893 0.50390117

A 0 0

G 0.924124514 0.453955901

G 5.169340463 0.113433803

T 0 0

G 1.202041824 0.296393875

T 0 0

G 2.02388852 0.481088255

T 0 0

G 2.588942709 0.668114248

C 3.735059761 0.664010624

C 2.065289807 0.199866755

A 0 0

C 5.133779264 0.117056856

C 2.097315436 0.805369128

A 0 0

C 2.681602172 0.11880516

T 0 0

C 3.379073756 0.120068611

C 1.582931865 0.430144529

T 0 0

G 0.888037611 0.15671252

G 2.628372498 0.487380331

C 2.211387776 0.191537524

T 0 0

A 0 0

A 0 0

T 0 0

T 0 0

T 0 0

T 0 0

T 0 0

T 0 0

G 1.290772719 0.244200244

T 0 0

A 0 0

G 0.365853659 0.12195122

A 0 0

G 0.534574927 0.103466115

A 0 0

T 0 0

A 0 0

G 1.789093411 0.086014106

G 2.340388918 0.275339873

G 5.698592516 0.240302094

T 0 0

T 0 0

T 0 0

T 0 0

G 1.781082483 0.069168252

C 3.504834254 0.379834254

C 2.43902439 0.103788272

A 0 0

T 0 0

G 2.862562511 0.27590964

T 0 0

T 0 0

G 1.484294097 0.293406973

G 3.905304994 0.501123207

C 4.787417905 0.414794331

C 2.126923742 0.449593637

A 0 0

G 1.118953348 0.327078671

G 5.133505599 0.137812231

C 5.088265836 0.501903773

T 0 0

A 0 0

G 1.344223243 0.204185809

T 0 0

C 1.122258119 0.578132971

T 0 0

T 0 0

G 0.551438911 0.413579183

A 0 0

A 0 0

C 1.303602058 0.394511149

T 0 0

G 0.852025735 0.904190576

C 1.079387187 0.313370474

T 0 0

G 2.459726312 0.034644033

G 7.825484765 0.519390582

C 8.34914611 0.396756943

C 3.761214631 0.172532781

T 0 0

C 1.331258645 0.121023513

A 0 0

A 0 0

G 0.562192551 0.193253689

T 0 0

G 0.464037123 0.32125647

A 0 0

T 0 0

C 0.299348477 0.440218348

T 0 0

G 2.262364083 0.263065591

C 5.25210084 0.332633053

C 1.955648682 0.436528724

T 0 0

G 3.546099291 0.086490227

C 8.504753673 0.155574762

C 2.607944732 0.155440415

T 0 0

T 0 0

G 2.470650079 0.543192571

G 3.415659485 0.122613417

G 7.135343619 0.806451613

C 7.787889394 0.227511376

T 0 0

C 4.944375773 0.300194243

C 4.117543551 1.319725497

C 1.62786627 1.19026781

A 0 0

A 0 0

A 0 0

G 0.565570873 0.335807706

T 0 0

G 1.1942959 0.303030303

C 1.466905188 0.697674419

T 0 0

A 0 0

G 1.243339254 0.55062167

G 1.863023421 0.496806246

A 0 0

T 0 0

T 0 0

A 0 0

C 1.531066406 0.249243368

A 0 0

G 1.655098772 0.480512547

G 5.654974946 0.304223336

G 4.81216458 1.180679785

A 0 0

T 0 0

G 1.631994261 0.25107604

A 0 0

G 3.5759666 0.127064803

C 4.348613879 0.326146041

C 2.370611654 0.108577633

A 0 0

C 5.725121907 0.252844501

C 3.280173976 0.616165277

A 0 0

T 0 0

G 1.953556948 0.755621084

G 2.213613724 0.276701716

T 0 0

C 0.33557047 0.57792692

G 0 0

A 0 0

C 0.673400673 0.505050505

T 0 0

T 0 0

C 0.848896435 0.113186191

A 0 0

T 0 0

G 0.356740518 0.05632745

A 0 0

T 0 0

A 0 0

A 0 0

A 0 0

A 0 0

C 0.660626652 0.113250283

T 0 0

T 0 0

C 0.28132033 0.131282821

A 0 0

G 0.803888577 0.05608525

T 0 0

G 1.307433694 0.186776242

G 1.93452381 0.502232143

A 0 0

T 0 0

G 1.318477252 0.148560817

A 0 0

G 2.791643557 0.12941394

G 4.817275748 0.313768918

A 0 0

G 4.640883978 0.368324125

C 4.298892989 0.36900369

T 0 0

G 3.234105108 0.073502389

C 7.655677656 0.128205128

C 1.909090909 0.309090909

T 0 0

C 1.814882033 0.054446461

T 0 0

T 0 0

A 0 0

T 0 0

G 1.174527436 0.77078363

A 0 0

T 0 0

G 0.833951075 0.111193477

A 0 0

A 0 0

C 0.870692849 0.111152279

A 0 0

A 0 0

A 0 0

G 0.770500826 0.055035773

A 0 0

A 0 0

G 2.178327488 0.258445634

G 6.346863469 0.129151292

T 0 0

G 0.797182054 0.333704116

G 1.754710011 0.295530107

T 0 0

T 0 0

T 0 0

C 0.451043037 0.432249577

T 0 0

T 0 0

G 0.130645763 0.410600971

A 0 0

A 0 0

A 0 0

T 0 0

G 2.302265132 0.074266617

G 1.127124908 0.424981523

A 0 0

A 0 0

T 0 0

C 1.804922516 0.437556974

T 0 0

A 0 0

C 2.975653742 0.522993688

T 0 0

C 4.536559331 0.231275574

C 1.99752519 0.335867067

T 0 0

G 0.99720084 0.297410777

G 3.326367119 0.208986416

T 0 0

G 0.667720963 0.105429626

A 0 0

A 0 0

G 0.51200565 0.247175141

A 0 0

T 0 0

G 2.168164992 0.158646219

C 1.566349877 0.299190426

T 0 0

G 0.924633636 0.261688765

T 0 0

G 0.506727241 0.349467063

A 0 0

A 0 0

C 7.43006993 0.06993007

A 0 0

T 0 0

T 0 0

G 1.237364936 1.010805159

T 0 0

T 0 0

G 0.210674157 0.193117978

A 0 0

A 0 0

A 0 0

T 0 0

G 0.423803638 0.247218789

A 0 0

C 1.131341701 1.095987272

A 0 0

A 0 0

G 0.619140279 0.053069167

A 0 0

A 0 0

A 0 0

G 0.440451022 1.391825229

A 0 0

A 0 0

T 0 0

T 0 0

T 0 0

A 0 0

C 0.891608392 0.104895105

A 0 0

G 0.993490922 0.085645769

T 0 0

G 0.927766733 0.21537442

T 0 0

T 0 0

A 0 0

C 6.576561727 0.148343498

A 0 0

T 0 0

A 0 0

G 1.028667791 0.640809444

A 0 0

G 1.110719323 0.881523272

T 0 0

T 0 0

A 0 0

G 0.931065354 0.411817368

T 0 0

T 0 0

G 0.614717049 1.193274272

A 0 0

T 0 0

G 0.781391968 0.072687625

A 0 0

A 0 0

G 2.309090909 0.290909091

C 2.164029824 0.20003637

A 0 0

G 2.242314647 0.072332731

T 0 0

A 0 0

G 2.253316373 0.072687625

C 1.802293829 0.091024941

A 0 0

G 0.948040109 0.364630811

G 2.769117917 0.110031175

A 0 0

T 0 0

T 0 0

C 0.205223881 0.149253731

G 0.037404152 0.280531139

A 0 0

G 0.600262615 0.206340274

A 0 0

G 1.255230126 0.418410042

G 3.010097161 0.247666222

A 0 0

T 0 0

C 0.172149962 0.038255547

G 0 0

A 0 0

T 0 0

T 0 0

C 1.494539184 0.134125311

C 0.651965484 0.172579099

A 0 0

A 0 0

T 0 0

T 0 0

T 0 0

C 0.153994225 0.134744947

A 0 0

A 0 0

A 0 0

A 0 0

T 0 0

A 0 0

A 0 0

G 0.720789074 0.056904401

T 0 0

T 0 0

C 0.845864662 0.037593985

T 0 0

T 0 0

C 0.844753144 0.150178337

T 0 0

G 0.970149254 0.037313433

T 0 0

G 1.013645224 0.233918129

G 2.07444746 0.872431175

G 8.990291262 0.660194175

T 0 0

A 0 0

A 0 0

A 0 0

A 0 0

T 0 0

G 3.382120253 0.514240506

C 2.216505047 0.079160895

T 0 0

A 0 0

T 0 0

C 0.296677215 0.138449367

A 0 0

A 0 0

A 0 0

T 0 0

G 0.990687537 0.455716267

G 1.548540798 0.913242009

C 0.239712345 0.679184978

G 0 0

T 0 0

C 0.10034116 0.180614088

G 0.316957211 0.752773376

C 0.435643564 0.277227723

A 0 0

T 0 0

G 2.937697161 0.098580442

C 2.992034195 0.641150185

T 0 0

A 0 0

C 2.652982184 0.135553834

A 0 0

G 0.688976378 0.236220472

A 0 0

G 0.477421922 0.238710961

A 0 0

A 0 0

A 0 0

T 0 0

C 0.289495451 0.434243176

T 0 0

A 0 0

T 0 0

C 0.436862908 0.166423965

A 0 0

T 0 0

G 0.454263886 0.185835226

A 0 0

A 0 0

A 0 0

G 1.240181893 0.413393964

G 4.244306418 0.144927536

A 0 0

A 0 0

G 0.938086304 0.083385449

A 0 0

G 1.174250367 0.020968757

T 0 0

C 0.75837371 0

A 0 0

A 0 0

T 0 0

T 0 0

G 0.465707028 0.148179509

A 0 0

T 0 0

G 1.281229981 0.106769165

T 0 0

G 1.032702238 0.15060241

G 1.851054671 0.27981059

C 1.358930112 0.064710958

A 0 0

A 0 0

A 0 0

C 0.735453169 0.173047804

T 0 0

T 0 0

C 0.763025943 0.065402224

A 0 0

T 0 0

T 0 0

G 1.656134234 0.283286119

T 0 0

T 0 0

G 0.667408231 0.266963293

T 0 0

C 0.31256977 0.022326412

G 0.089325592 0.11165699

T 0 0

A 0 0

T 0 0

T 0 0

T 0 0

T 0 0

A 0 0

A 0 0

G 0.446129824 0.223064912

A 0 0

A 0 0

A 0 0

T 0 0

T 0 0

G 1.052867384 0.201612903

T 0 0

C 0.898270829 0.359308331

A 0 0

C 0.044812906 0.425722608

G 0 0

A 0 0

C 2.590442162 0.178651184

C 1.603206413 0.222667557

A 0 0

C 15.08725425 0.066269052

C 7.326250553 0.044267375

C 5.935769657 0.310077519

C 4.67745511 0.243848371

A 0 0

A 0 0

C 6.878781089 0.112032265

C 1.282932417 0.137457045

T 0 0

T 0 0

C 0.327332242 0.818330606

A 0 0

A 0 0

C 1.844932275 0.186828585

A 0 0

A 0 0

C 3.054848415 0.763712104

C 1.647714087 0.069621722

A 0 0

T 0 0

G 3.475258918 0.391254315

A 0 0

C 9.524897213 0.365463682

C 4.889193512 0.434087274

C 2.647797307 1.004336909

T 0 0

G 0.730270907 0.565371025

A 0 0

T 0 0

C 0.550502633 0.622307324

A 0 0

G 1.03480715 0.352775165

T 0 0

C 0.635742877 0.235460325

A 0 0

G 2.398871119 0.776105362

G 4.789995281 0.613496933

A 0 0

G 4.127893104 0.548795037

C 4.600715137 1.144219309

C 2.93255132 0.122189638

A 0 0

T 0 0

C 3.393554688 1.611328125

C 2.602504297 0.319175055

A 0 0

C 4.046673287 0.42204568

A 0 0

T 0 0

T 0 0

G 0.436456996 0.513478819

A 0 0

G 0.940193262 0.49621311

G 4.360313316 1.227154047

C 0.131509732 0.026301946

G 0 0

A 0 0

G 0.452007445 0.664716831

A 0 0

A 0 0

C 6.498388829 0.37593985

C 1.618122977 0.916936354

T 0 0

C 3.390742734 0.807319699

C 1.816724552 0.160299225

A 0 0

G 1.072386059 0.053619303

C 1.392608463 0.669523299

A 0 0

G 0.683994528 0.164158687

T 0 0

A 0 0

A 0 0

A 0 0

A 0 0

A 0 0

G 0.194013304 0.249445676

A 0 0

T 0 0

T 0 0

A 0 0

T 0 0

G 1.024646912 0.110772639

A 0 0

T 0 0

T 0 0

C 0.603897886 0.356848751

T 0 0

C 1.055480379 0.216508796

T 0 0

A 0 0

A 0 0

A 0 0

G 1.569264069 0.162337662

G 2.997569538 0.243046179

A 0 0

T 0 0

C 0.439198463 0.274499039

A 0 0

G 0.441135925 0.220567963

A 0 0

T 0 0

G 0.724839699 0.1115138

A 0 0

A 0 0

C 7.065368567 0

A 0 0

T 0 0

T 0 0

A 0 0

G 1.306447032 0.170406135

C 2.582292849 0.056753689

A 0 0

T 0 0

T 0 0

T 0 0

T 0 0

T 0 0

T 0 0

T 0 0

A 0 0

A 0 0

G 0.880431696 0.085203067

C 1.249290176 0.085178876

A 0 0

A 0 0

T 0 0

A 0 0

A 0 0

A 0 0

G 0.885200553 0.027662517

T 0 0

A 0 0

T 0 0

T 0 0

T 0 0

T 0 0

T 0 0

A 0 0

C 0.301122365 0.191623323

G 0 0

T 0 0

A 0 0

A 0 0

G 0.190010858 0.190010858

A 0 0

T 0 0

A 0 0

T 0 0

G 8.567639257 0.079575597

T 0 0

A 0 0

T 0 0

G 7.220312087 0.343824385

T 0 0

T 0 0

A 0 0

T 0 0

T 0 0

T 0 0

T 0 0

T 0 0

T 0 0

A 0 0

G 0.77457265 0.560897436

G 3.751995742 0

C 25.39218293 0.186120713

A 0 0

T 0 0

A 0 0

A 0 0

T 0 0

G 20.1128784 0.487429451

C 0.764136526 0.433010698

T 0 0

A 0 0

T 0 0

T 0 0

A 0 0

T 0 0

G 6.209235586 0.026089225

C 4.535974974 0.026068822

A 0 0

T 0 0

T 0 0

T 0 0

A 0 0

A 0 0

T 0 0

A 0 0

G 0.467775468 0.285862786

A 0 0

C 3.289642765 0.179902339

T 0 0

C 2.771478962 0.453514739

C 3.259779338 0.551654965

A 0 0

G 1.549612597 0.074981255

T 0 0

A 0 0

T 0 0

A 0 0

T 0 0

T 0 0

G 1.79028133 0.127877238

T 0 0

A 0 0

A 0 0

A 0 0

C 14.57800512 0.434782609

A 0 0

T 0 0

A 0 0

A 0 0

C 0.540262413 0.308721379

T 0 0

T 0 0

T 0 0

A 0 0

A 0 0

A 0 0

T 0 0

G 2.974171667 0.234803026

C 1.541677554 0.627123073

A 0 0

C 1.562093205 0.104139547

T 0 0

G 1.5625 0.598958333

G 3.829122167 0.312581401

G 4.050169846 0.418082049

A 0 0

G 0.577731092 0.078781513

A 0 0

T 0 0

A 0 0

A 0 0

A 0 0

A 0 0

G 0.509383378 0

T 0 0

A 0 0

T 0 0

T 0 0

T 0 0

G 1.070090958 0.214018192

C 0.641368252 0.160342063

T 0 0

C 0.746268657 0.026652452

T 0 0

T 0 0

T 0 0

T 0 0

A 0 0

T 0 0

G 0.846784864 0.132310135

A 0 0

T 0 0

A 0 0

T 0 0

T 0 0

T 0 0

G 0.633747029 0.052812252

C 1.424802111 0

T 0 0

T 0 0

T 0 0

A 0 0

T 0 0

T 0 0

G 0.90956341 0.12993763

C 1.04139547 0.104139547

A 0 0

G 1.818181818 0.025974026

T 0 0

A 0 0

G 1.68263008 0.129433083

T 0 0

C 0.909090909 0.337662338

T 0 0

G 1.624548736 0.180505415

T 0 0

A 0 0

A 0 0

T 0 0

G 2.733663109 0.390523301

G 1.306165099 0.313479624

A 0 0

A 0 0

A 0 0

C 1.134564644 0.211081794

T 0 0

A 0 0

C 3.726056153 0.446077145

A 0 0

T 0 0

T 0 0

A 0 0

T 0 0

C 0.691857371 0.47897818

T 0 0

C 0.687830688 0.238095238

T 0 0

T 0 0

G 1.793139293 0.285862786

G 3.461738678 0.416449766

G 9.799322387 0.052124055

T 0 0

A 0 0

C 6.490447527 0.261711594

A 0 0

C 6.543494996 0.256607647

C 1.514762516 0.718870347

T 0 0

G 2.207041513 0.236468734

T 0 0

A 0 0

T 0 0

A 0 0

C 1.348799568 0.161855948

A 0 0

G 0.642996925 0.838691641

A 0 0

A 0 0

A 0 0

G 0.28417164 0.369423132

A 0 0

A 0 0

A 0 0

T 0 0

T 0 0

T 0 0

A 0 0

T 0 0

C 47.91726515 0.229819018

A 0 0

T 0 0

G 1.431434297 0.14314343

A 0 0

G 1.145147438 0.114514744

G 3.011184399 0.143389733

A 0 0

A 0 0

A 0 0

T 0 0

G 3.208249785 0.057290175

C 2.895642202 0.114678899

T 0 0

C 1.372212693 0.171526587

A 0 0

T 0 0

G 1.81149165 0.113218228

C 1.215719536 0.452360758

A 0 0

A 0 0

T 0 0

G 1.252783964 0.222717149

A 0 0

T 0 0

G 6.262176454 0.083495686

G 2.165463631 0.388672959

A 0 0

G 6.581148912 0.027886224

G 4.233983287 0.111420613

C 4.376916643 0.0557569

T 0 0

G 0.679117148 0

G 1.217440544 0.453001133

A 0 0

A 0 0

A 0 0

G 1.422070535 0.255972696

T 0 0

C 2.868503266 0.255609202

C 2.243680773 0.397614314

C 1.534526854 0.255754476

A 0 0

A 0 0

G 0.596760443 0.198920148

A 0 0

T 0 0

C 0.660160735 0.401836969

T 0 0

G 0 0

C 1.288290867 0.14314343

A 0 0

G 1.082621083 0.341880342

T 0 0

C 0.690051754 0.40253019

A 0 0

G 0.717978173 0.114876508

C 1.692484223 0.258175559

A 0 0

A 0 0

G 0.773195876 0.257731959

T 0 0

T 0 0

G 1.695889623 0.718597298

G 2.013808976 0.172612198

A 0 0

G 1.754385965 0.316364682

A 0 0

C 8.306984766 0.431158379

C 2.959094865 0.667246881

C 2.796387999 0.17477425

A 0 0

T 0 0

G 0.873108265 0.407450524

A 0 0

G 0.523407967 0.116312882

A 0 0

G 0.702165009 0.175541252

T 0 0

C 0.147232038 1.266195524

G 0.117096019 0.790398126

A 0 0

T 0 0

G 1.526717557 0.440399295

G 4.183614178 0.319581639

T 0 0

G 2.199710564 0.20260492

T 0 0

G 5.855072464 0.202898551

G 12.57952574 0.2313476

C 15.7635468 0.434656621

C 12.25731672 0.434656621

C 10.45599768 0.522799884

C 5.693950178 0.177935943

A 0 0

G 1.274697259 0.159337157

T 0 0

C 1.019736842 0.394736842

T 0 0

G 0 0

T 0 0

G 0 0

T 0 0

T 0 0

T 0 0

G 0 0

A 0 0

A 0 0

G 4.836415363 2.524893314

G 12.42391694 0.501253133

C 12.59927798 0.180505415

C 3.975424648 0.325262017

T 0 0

G 1.701049584 2.605863192

A 0 0

G 0.901225667 0.468637347

A 0 0

A 0 0

C 3.322499097 0.505597689

C 0.97861544 0.108735049

A 0 0

G 0.835452234 2.833272793

G 2.838427948 0.327510917

A 0 0

G 0.99704579 0.295420975

A 0 0

G 2.113459399 0.259547646

C 2.674591382 2.303120357

C 0.668151448 0.259836674

A 0 0

A 0 0

A 0 0

A 0 0

G 2.336269628 0.153198008

C 1.495972382 0.038358266

T 0 0

G 0.38865138 0.038865138

G 2.022559315 0.350058343

C 2.107728337 0

A 0 0

A 0 0

G 4.78243826 0.078400627

C 5.664830842 0.078678206

T 0 0

C 0.787711698 0.07877117

T 0 0

G 1.145339652 0.276461295

G 2.249408051 0

A 0 0

C 8.359746434 0.118858954

C 2.757502028 0.324412003

C 2.204981625 0

A 0 0

G 0.98806093 0.576368876

G 3.796945935 0.082542303

A 0 0

A 0 0

G 0.381194409 0.635324015

A 0 0

G 3.448275862 0.732758621

C 1.690507152 0

T 0 0

G 0.391815411 0.435350457

A 0 0

T 0 0

G 1.100352113 0.308098592

T 0 0

T 0 0

T 0 0

C 0.355082113 0.177541056

A 0 0

G 0 0

T 0 0

T 0 0

C 0.585321927 0.180099054

A 0 0

A 0 0

G 0.905387053 0.633770937

T 0 0

C 0.636363636 0

C 0.227998176 0

G 0 0

A 0 0

A 0 0

G 1.888530631 0.783049286

G 5.719557196 0.691881919

C 3.098982424 0.138760407

A 0 0

G 0.791802515 1.117838845

G 2.702702703 0.139794967

A 0 0

A 0 0

A 0 0

A 0 0

G 0.967585873 0.290275762

A 0 0

C 1.70233463 0

T 0 0

G 0.296882731 0.544285007

A 0 0

A 0 0

G 6.162324649 0.651302605

G 13.53118712 0.201207243

C 12.74014156 0.151668352

C 8.053007136 0.560652396

C 4.239019408 0.408580184

A 0 0

G 2.725366876 1.153039832

C 4.997369805 0.263019463

T 0 0

C 3.004291845 0.160944206

C 4.256495301 0.11055832

A 0 0

G 1.169916435 0.445682451

G 3.790412486 2.006688963

C 2.578475336 0

A 0 0

G 1.494321578 0.298864316

T 0 0

C 0.968523002 0

A 0 0

G 0.910746812 0.485731633

G 4.32136336 0.243457091

C 2.681291895 0

A 0 0

G 1.362229102 0.619195046

G 0 0

A 0 0

G 0.662690524 0.265076209

A 0 0

A 0 0

C 1.57967033 0.206043956

T 0 0

T 0 0

T 0 0

C 4.133998574 0.071275837

C 2.220630372 0

T 0 0

T 0 0

T 0 0

T 0 0

A 0 0

C 1.793721973 0.074738416

T 0 0

C 0.840978593 0.076452599

A 0 0

C 0 0

G 0.232919255 0

A 0 0

G 0.714853058 0.31771247

A 0 0

G 3.281378179 2.132895816

G 7.8125 0.082236842

G 7.024793388 0.165289256

T 0 0

C 2.533783784 0.084459459

A 0 0

G 1.583113456 0.175901495

C 3.625110522 1.503094607

A 0 0

T 0 0

T 0 0

T 0 0

T 0 0

G 0 0

T 0 0

T 0 0

C 1.088031652 0.59347181

T 0 0

G 0 0

T 0 0

T 0 0

C 0.9375 0.416666667

A 0 0

G 1.948051948 1.406926407

G 3.687635575 2.060737527

C 3.977900552 0.220994475

T 0 0

G 0.374531835 1.373283396

T 0 0

C 2.374670185 0.395778364

A 0 0

A 0 0

C 3.85126162 0

T 0 0

G 0 0

A 0 0

G 1.928783383 2.818991098

T 0 0

G 0.850340136 5.782312925

A 0 0

C 6.034482759 4.310344828

C 2.664298401 6.039076377

C 0.179533214 0.897666068

A PRIMER NA NA

G PRIMER NA NA

A PRIMER NA NA

A PRIMER NA NA

A PRIMER NA NA

A PRIMER NA NA

G PRIMER NA NA

C PRIMER NA NA

T PRIMER NA NA

G PRIMER NA NA

G PRIMER NA NA

C PRIMER NA NA

A PRIMER NA NA

C PRIMER NA NA

A PRIMER NA NA

T PRIMER NA NA

A PRIMER NA NA

A PRIMER NA NA

C PRIMER NA NA

A PRIMER NA NA

T PRIMER NA NA

T PRIMER NA NA

C PRIMER NA NA

A PRIMER NA NA

C PRIMER NA NA

C NA NA

A NA NA

T NA NA

C NA NA

G NA NA

T NA NA

G NA NA

C NA NA

T NA NA

G NA NA

C NA NA

A NA NA

A NA NA

G NA NA

A NA NA

G NA NA

C NA NA

T NA NA

G NA NA

C NA NA

A NA NA

A NA NA

A NA NA

A NA NA

C NA NA

C NA NA

C NA NA

T NA NA

C NA NA

T NA NA

C NA NA

T NA NA

G NA NA

C NA NA

T NA NA

T NA NA

C NA NA

T NA NA

A NA NA

A NA NA

C NA NA

A NA NA

C NA NA

T NA NA

G NA NA

A NA NA

T NA NA

G NA NA

C NA NA

T NA NA

C NA NA

A NA NA

G NA NA

C NA NA

C NA NA

C NA NA

A NA NA

C NA NA

C NA NA

T NA NA

C NA NA

C NA NA

A NA NA

G NA NA

T NA NA

G NA NA

G NA NA

G NA NA

C NA NA

A NA NA

G NA NA

G NA NA

G NA NA

A NA NA

G NA NA

C NA NA

T NA NA

G NA NA

G NA NA

G NA NA

T NA NA

G NA NA

C NA NA

C NA NA

G NA NA

G NA NA

G NA NA

A NA NA

G NA NA

G NA NA

A NA NA

C NA NA

T NA NA

T NA NA

G NA NA

G NA NA

G NA NA

G NA NA

T NA NA

T NA NA

G NA NA

C NA NA

C NA NA

A NA NA

G NA NA

C NA NA

C NA NA

C NA NA

A NA NA

G NA NA

T NA NA

G NA NA

T NA NA

G NA NA

G NA NA

G NA NA

C NA NA

C NA NA

T NA NA

G NA NA

G NA NA

A NA NA

C NA NA

A NA NA

G NA NA

T NA NA

T NA NA

G NA NA

C NA NA

T NA NA

G NA NA

A NA NA

G NA NA

A NA NA

A NA NA

T NA NA

C NA NA

T NA NA

C NA NA

C NA NA

C NA NA

T NA NA

C NA NA

C NA NA

G NA NA

C NA NA

C NA NA

C NA NA

T NA NA

G NA NA

T NA NA

G NA NA

A NA NA

C NA NA

T NA NA

T NA NA

C NA NA

T NA NA

T NA NA

A NA NA

A NA NA

T NA NA

T NA NA

A NA NA

C NA NA

T NA NA

T NA NA

A NA NA

G NA NA

A NA NA

G NA NA

G NA NA

G NA NA

T NA NA

C NA NA

A NA NA

C NA NA

C NA NA

C NA NA

T NA NA

G NA NA

G NA NA

T NA NA

T NA NA

A NA NA

G NA NA

C NA NA

T NA NA

T NA NA

C NA NA

A NA NA

C NA NA

T NA NA

A NA NA

G NA NA

T NA NA

C NA NA

T NA NA

C NA NA

G PRIMER NA NA

T PRIMER NA NA

G PRIMER NA NA

G PRIMER NA NA

A PRIMER NA NA

A PRIMER NA NA

A PRIMER NA NA

A PRIMER NA NA

T PRIMER NA NA

T PRIMER NA NA

T PRIMER NA NA

G PRIMER NA NA

C PRIMER NA NA

C PRIMER NA NA

T PRIMER NA NA

C PRIMER NA NA

C PRIMER NA NA

T PRIMER NA NA

A PRIMER NA NA

A PRIMER NA NA

A PRIMER NA NA

A PRIMER NA NA

T PRIMER NA NA

G PRIMER NA NA

T PRIMER NA NA

C NA NA

T NA NA

T NA NA

T NA NA

C NA NA

C NA NA

T NA NA

C NA NA

T NA NA

G NA NA

A NA NA

G NA NA

A NA NA

A NA NA

A NA NA

G NA NA

C NA NA

C NA NA

C NA NA

A NA NA

G NA NA

G NA NA

C 9.922178988 1.361867704

C 4 3.80952381

T 0 0

C 1.305057096 0.815660685

C 1.286173633 0.803858521

A 0 0

A 0 0

A 0 0

G 8.443271768 0.131926121

G 17.03800786 0.262123198

C 12.15189873 0.379746835

C 7.547169811 0.503144654

C 3.740648379 0.249376559

A 0 0

G 3.191489362 0

C 3.040935673 0.584795322

C 1.50812065 0.348027842

A 0 0

G 3.139013453 0.224215247

G 7.880133185 1.66481687

C 4.299889746 0.771775083

T 0 0

G 1.170212766 1.808510638

G 2.210526316 1.894736842

C 1.995798319 1.470588235

G 0 0

T 0 0

C 0.818833163 0.921187308

A 0 0

G 1.108033241 0.184672207

T 0 0

C 1.005484461 1.55393053

T 0 0

G 3.671328671 0.087412587

G 5.492589364 0

G 5.603448276 1.206896552

G 10.54852321 4.641350211

T 0 0

C 0.831946755 0.083194676

A 0 0

G 2.5 0.161290323

C 3.12 3.52

T 0 0

G 0.228832952 0.305110603

C 2.872260015 0.377928949

G 0.224887556 2.623688156

G 2.629656684 3.360116874

G 9.637681159 0.289855072

G 9.279086367 4.496788009

A 0 0

G 6.349206349 0.27605245

G 8.776595745 0.997340426

C 4.064516129 1.161290323

T 0 0

C 1.501877347 1.001251564

C 1.057871811 0.87118855

A 0 0

G 3.64614465 0.179318589

G 9.512485137 0.237812128

C 5.601907032 3.277711561

C 3.683897802 2.554961378

A 0 0

T 0 0

G 2.070509233 0.335758254

T 0 0

G 3.387007218 0.055524708

T 0 0

G 2.580999451 0.054914882

A 0 0

C 1.45137881 0.822447992

A 0 0

C 0.622605364 0.526819923

G 53.37159254 0

G 2.693761815 0.047258979

G 3.406439571 0.046663556

A 0 0

G 0.75926753 0

T 0 0

T 0 0

T 0 0

A 0 0

C 8.099824869 0.262697023

C 3.847835592 0.218627022

C 3.33477696 0.346470333

C 2.379922112 0.302899178

A 0 0

T 0 0

C 3.363133248 0.297999149

C 2.03562341 0.127226463

C 0.464919696 0.211327134

A 0 0

G 0.752508361 0.2090301

T 0 0

T 0 0

T 0 0

C 1.443894389 0.165016502

C 0.86526576 0.082406263

A 0 0

G 1.055623224 0

T 0 0

G 4.262163249 0.040209087

G 4.131568391 0.040112314

A 0 0

G 4.41944556 0.080353556

A 0 0

A 0 0

G 1.678657074 0

C 1.598082301 0.15980823

A 0 0

T 0 0

C 0.118906064 0.198176774

G 0.235201882 0.235201882

T 0 0

T 0 0

C 0.309477756 0.232108317

T 0 0

C 0.303720577 0.227790433

G 0.075301205 0

T 0 0

C 1.300631735 0.260126347

C 1.479837218 0.332963374

A 0 0

C 11.32556446 0.400582666

C 5.771324864 0.072595281

C 5.333817126 0.181422351

C 2.062228654 0.18089725

C 0.32444124 0.396539293

G 0 0

T 0 0

C 0.322696307 0.143420581

A 0 0

T 0 0

G 2.537526805 0.035739814

C 1.178571429 0.321428571

T 0 0

C 0.670194004 0.211640212

T 0 0

T 0 0

T 0 0

T 0 0

C 1.19372442 0.272851296

C 2.316076294 0.272479564

A 0 0

C 6.268958544 0.202224469

C 1.210490921 0.100874243

T 0 0

T 0 0

C 1.137841352 0.097529259

T 0 0

C 1.561504143 0.191204589

C 1.905972046 0.889453621

A 0 0

G 3.293601004 0.2195734

G 7.894736842 0.187969925

G 8.512628625 0.09354537

G 6.327930175 0.872817955

A 0 0

A 0 0

G 2.466437715 0.249765845

G 5.588326607 0.155231295

G 3.533787973 0.247985121

A 0 0

T 0 0

A 0 0

T 0 0

T 0 0

T 0 0

T 0 0

C 0.3347535 0.517346318

A 0 0

G 1.177536232 0.030193237

T 0 0

C 0.38887227 0.837571044

T 0 0

G 2.590819487 0.028161081

T 0 0

A 0 0

C 2.086270087 0.084578517

A 0 0

A 0 0

C 0.363738109 0.279798545

G 0.224152424 0.112076212

A 0 0

A 0 0

T 0 0

C 0.922818792 0.643176734

C 0.587412587 0.615384615

A 0 0

C 0.499029665 0.249514832

T 0 0

G 0.110283981 0.082712986

A 0 0

A 0 0

A 0 0

T 0 0

G 4.267963263 0.081037277

T 0 0

T 0 0

A 0 0

A 0 0

G 1.437908497 0.392156863

G 5.659397715 0.05192108

T 0 0

G 2.618615504 0.259268862

G 3.700828157 0.155279503

G 7.065497679 0.154718927

C 3.375418707 0.15459933

A 0 0

C 1.484514973 0.230355772

A 0 0

G 0.991357397 0.330452466

T 0 0

G 1.386138614 0.420792079

G 3.176930596 0.244379277

T 0 0

C 1.193666261 0.267965895

T 0 0

G 2.577692122 0.602264515

G 4.257878278 0.45706038

G 6.284613532 0.794606309

G 9.954315941 0.096176966

T 0 0

C 1.35494798 0.435518993

T 0 0

T 0 0

C 0.095762509 0.670337563

G 0.168107589 0.192122959

A 0 0

C 3.107817356 0.669376046

C 1.054902901 0.839127308

T 0 0

T 0 0

G 1.038541426 0.115393492

T 0 0

T 0 0

T 0 0

A 0 0

T 0 0

A 0 0

C 0.205667276 0.52559415

G 0.228832952 0.343249428

T 0 0

G 0.996151234 1.17726964

G 3.427282976 0.67643743

T 0 0

G 3.209384683 0.531208499

C 5.076142132 0.110350916

C 1.29499561 0.395083406

T 0 0

G 1.861069033 0.54100844

T 0 0

T 0 0

A 0 0

C 0.946643718 1.032702238

T 0 0

G 1.026957638 1.112537441

G 2.351432236 0.534416417

T 0 0

C 0.106791969 0.363092695

G 0.148999574 0.425713069

G 1.674937965 0.330851944

G 6.33409232 0.372593666

T 0 0

C 0.637728862 0.658300761

T 0 0

G 4.707278481 0.079113924

T 0 0

A 0 0

C 4.235618597 0.098502758

A 0 0

G 1.789264414 0.357852883

G 5.177220231 0.716845878

C 2.604891629 0.039769338

A 0 0

G 0.735001986 0.437028208

T 0 0

T 0 0

T 0 0

C 1.871227364 0.402414487

C 1.487437186 0.72361809

A 0 0

C 1.043548063 0.060204696

T 0 0

G 1.028640581 0.262202501

G 1.59789644 0.343851133

C 0.323167037 0.646334074

G 0 0

T 0 0

T 0 0

T 0 0

T 0 0

T 0 0

T 0 0

A 0 0

C 2.573899055 0.341845968

C 0.784392599 1.045856798

A 0 0

G 1.950636943 0.218949045

G 6.432281262 0.439472633

C 5.152786099 0.519273018

C 2.457542458 0.37962038

A 0 0

G 4.045118631 0.077790743

C 4.724868754 0.369434182

C 1.771461943 0.272532607

T 0 0

A 0 0

G 0.943577893 0.192566917

A 0 0

G 1.855431001 0.231928875

T 0 0

A 0 0

G 0.586624951 0.508408291

A 0 0

A 0 0

T 0 0

G 1.183898974 0.374901342

A 0 0

C 1.961938395 0.392387679

C 0.452044025 0.412735849

G 0 0

C 1.138370952 0.215897939

A 0 0

T 0 0

G 2.036067481 0.271475664

T 0 0

T 0 0

A 0 0

A 0 0

A 0 0

A 0 0

T 0 0

A 0 0

T 0 0

A 0 0

G 0.523458705 0.620395502

A 0 0

T 0 0

T 0 0

C 2.825070159 0.130963517

C 1.563954571 0.279277602

T 0 0

G 5.882352941 0.203477617

G 11.13349203 0.018311665

G 19.92046276 0.14461316

C 13.12713541 0.161841395

C 12.07920792 0.0360036

C 8.403815008 0.197948533

C 3.257739381 0.557955364

A 0 0

G 1.400764053 0.20010915

A 0 0

C 4.617630955 0.219018069

C 0.683287165 0.424746076

T 0 0

G 1.341172148 0.367444424

C 2.68185158 0.679647318

C 0.86286029 0.146869837

A 0 0

G 0.330699982 0.91861106

A 0 0

A 0 0

T 0 0

C 0.495594714 0.201908957

T 0 0

C 0.201834862 0.183486239

T 0 0

G 0.439479949 0.366233291

T 0 0

G 0.275431509 0.165258906

A 0 0

G 0.547745116 0.200839876

A 0 0

T 0 0

G 2.876511543 0.091608648

G 1.907207042 0.036677058

A 0 0

A 0 0

C 5.440796754 0.165990409

C 0.884792627 0.073732719

T 0 0

T 0 0

T 0 0

C 0.384967919 0.164986251

A 0 0

A 0 0

C 2.926650814 0.128040973

C 0.311697836 0.256692336

T 0 0

G 1.014572957 0.036893562

G 1.677419355 0.571428571

G 3.324200913 0.420091324

T 0 0

T 0 0

T 0 0

T 0 0

A 0 0

A 0 0

A 0 0

C 0.658978583 0.201354567

A 0 0

A 0 0

G 5.888847994 0.1104159

C 5.733650852 0.201502107

C 1.428309833 0.256363303

T 0 0

C 4.069767442 0.163517442

C 1.628369821 0.379952958

C 0.18092998 0.18092998

G 0 0

G 1.591320072 0.34358047

G 6.771682057 0.144848814

T 0 0

G 0.383982447 0.329127811

A 0 0

T 0 0

T 0 0

C 0.397040245 0.162425555

T 0 0

G 0.45232495 0.09046499

A 0 0

C 0.994215474 0.343456255

A 0 0

C 1.213768116 0.434782609

T 0 0

C 0.620891161 0.109569028

A 0 0

C 0.91374269 0.109649123

T 0 0

G 1.077822433 0.109609061

G 0.787257415 0.292932992

A 0 0

T 0 0

T 0 0

T 0 0

G 0.312213039 0.183654729

A 0 0

G 0.387883266 0.258588844

A 0 0

A 0 0

C 1.995606005 0.256316368

C 0.365497076 0.255847953

G 0 0

T 0 0

G 1.62260711 0.455788514

G 2.967953387 0.36416606

G 4.386603568 0.40043684

G 8.242358079 0.545851528

T 0 0

T 0 0

G 0.932699342 0.164594001

T 0 0

T 0 0

C 0.440366972 0.385321101

A 0 0

G 0.53338238 0.239102446

A 0 0

C 0.476626948 0.25664528

A 0 0

G 1.090512541 0.490730643

C 1.78343949 0.327570519

A 0 0

G 2.512288367 0.218459858

G 6.024754277 0.236621769

G 5.84261457 0.273872558

A 0 0

C 0.273872558 0.091290853

G 0.182681768 0.018268177

T 0 0

T 0 0

G 0.185219485 0.185219485

A 0 0

T 0 0

G 1.645101664 0.369685767

T 0 0

T 0 0

G 1.599264706 0.128676471

T 0 0

T 0 0

C 3.183072677 0.128794848

C 1.423290203 0.11090573

T 0 0

T 0 0

C 0.347349177 0.164533821

T 0 0

G 0.439479949 0.677531588

C 0.201871903 0.14681593

G 0.128228613 0.531232827

T 0 0

T 0 0

C 2.91704649 0.200546946

C 1.128914785 0.291332848

T 0 0

G 1.180315962 0.780824405

G 3.413217139 0.381263617

T 0 0

G 0.477502296 0.238751148

A 0 0

T 0 0

G 1.758607923 0.166604961

C 1.221317543 0.222057735

T 0 0

G 0.716648291 0.275633958

T 0 0

T 0 0

C 0.401753104 0.73046019

T 0 0

G 0.473674622 0.382583349

T 0 0

T 0 0

C 0.907276356 1.45164217

T 0 0

C 4.274265361 0.338379341

C 3.841365819 0.426818424

C 1.141837645 0.517395183

A 0 0

A 0 0

G 5.221604163 0.161492912

G 10.05376344 0.107526882

C 10.56590258 0.698424069

C 4.387535817 0.411891117

T 0 0

A 0 0

T 0 0

G 0.743696717 0.399056775

C 0.199673262 1.016518424

G 0.31152648 0.568077698

G 3.572730587 0.729128691

T 0 0

G 1.601455869 0.727934486

G 3.145454545 0.654545455

T 0 0

G 0 0

A 0 0

G 0 0

A 0 0

A 0 0

T 0 0

C 0.366770585 0.550155877

T 0 0

C 1.33985153 0.506970849

C 1.117519827 0.072098053

A 0 0

A 0 0

G 1.626742939 0.053628888

G 3.361792956 0.853788687

A 0 0

T 0 0

A 0 0

C 2.022511432 0.228631727

A 0 0

A 0 0

G 1.041482789 0.052956752

A 0 0

C 0.76173605 0.070859167

A 0 0

G 0.88177074 0.521864315

T 0 0

G 0.339710352 0.661541212

A 0 0

T 0 0

C 0.213675214 0.213675214

T 0 0

G 1.193444959 0.391877449

G 1.972982581 0.195520796

A 0 0

G 1.228632479 0.071225071

C 0.126126126 0.198198198

G 0 0

A 0 0

G 0.95409541 0.270027003

T 0 0

G 1.472172352 0.287253142

T 0 0

C 2.468039773 0.230823864

C 1.31112686 0.407512403

T 0 0

G 0 0

A 0 0

A 0 0

A 0 0

G 1.091234347 0.089445438

C 0.983723842 0.107315328

A 0 0

G 0.987078248 0.430725054

A 0 0

C 1.70969993 0.104675506

T 0 0

C 0.625869263 0.034770515

T 0 0

A 0 0

G 1.196047842 0.10400416

C 2.025974026 0.155844156

A 0 0

C 1.481992073 0.103394796

T 0 0

C 0.531067446 0.053106745

A 0 0

G 1.334282156 0.266856431

G 1.996790872 0.08914245

A 0 0

C 1.92479059 0.03564427

T 0 0

G 0.800996796 0.053399786

C 2.707034728 0.765805877

C 1.173959445 0.088936322

A 0 0

A 0 0

C 1.410934744 0.423280423

A 0 0

C 10.58927001 0.087950748

C 7.09106985 0.17683466

C 4.367043847 0.141442716

T 0 0

C 8.469170801 0.194897236

C 6.568696884 0.035410765

C 1.996466431 0.123674912

C 0.302168503 0.24884465

G 0 0

G 0.837640349 0.267332026

G 3.450115597 0.088920505

T 0 0

T 0 0

T 0 0

C 4.487524681 0.14360079

C 1.417040359 0.107623318

T 0 0

T 0 0

G 0.432822362 0.162308386

G 2.172741964 0.071826181

T 0 0

C 0.522052205 0.108010801

T 0 0

G 0.39497307 0.341113106

G 1.171171171 0.396396396

A 0 0

A 0 0

T 0 0

T 0 0

C 2.355072464 0.326086957

C 2.336110105 0.507062658

C 1.680216802 0.252935863

A 0 0

T 0 0

C 6.320501343 0.214861235

C 8.216004277 0.267332026

C 6.970939561 0.178284899

C 2.563189747 0.266998932

T 0 0

G 0.93639576 0.141342756

G 2.293982707 0.194106229

T 0 0

T 0 0

C 2.550026563 0.247919249

C 2.719033233 0.159943131

A 0 0

C 6.474947071 0.194071983

C 0.930640913 0.368744513

T 0 0

G 1.429832304 0.247131509

T 0 0

T 0 0

A 0 0

C 2.390998594 0.228551336

A 0 0

T 0 0

C 0.739957717 0.475687104

A 0 0

C 0.93015093 0.298350298

A 0 0

C 6.334300837 0.307324569

C 1.765815189 0.565746614

T 0 0

C 7.276151344 0.496490327

C 9.056668379 0.325286766

C 8.898377455 0.666097353

C 3.732106339 0.783912747

T 0 0

T 0 0

C 0.61553818 0.116453169

A 0 0

A 0 0

G 3.236245955 0.408788963

G 4.272634791 0.457782299

A 0 0

C 4.674485807 0.645928948

C 1.20809937 0.544495491

A 0 0

G 1.666953085 0.223406084

T 0 0

G 0.983606557 0.345125108

C 0.913163336 0.155065472

A 0 0

G 0.589356908 0.554688854

A 0 0

T 0 0

G 2.665040934 0.174185682

C 2.594670407 0.315568022

C 1.690736175 0.176118352

A 0 0

C 0.63213345 0.105355575

G 0.385086644 0.420094521

T 0 0

C 2.759343346 0.087320992

C 1.865086282 0.12201499

T 0 0

T 0 0

C 0.500690608 0.120856354

A 0 0

C 0.08617718 0.068941744

G 0.157095479 0.209460639

G 2.749080721 0.315181229

G 4.913702008 0.03522367

G 7.547501759 0.33427164

C 11.96130167 0.035180299

T 0 0

C 0.581292936 0

A 0 0

G 0.229034531 0.229034531

A 0 0

A 0 0

T 0 0

G 2.574720922 0.144040331

C 2.01366415 0.233728875

T 0 0

C 0.933908046 0.269396552

A 0 0

C 4.726235063 0.196183342

C 0.875781948 0.339588919

A 0 0

G 3.363427155 0.407151708

C 4.505300353 0.265017668

T 0 0

T 0 0

C 3.77619955 0.13857613

C 1.953665284 0.345781466

T 0 0

C 4.09215956 0.464236589

T 0 0

C 2.80198189 0.615069195

C 4.309165527 0.153898769

A 0 0

C 2.439434724 0.420592194

C 0.220863065 0.951410126

G 0.235769619 0.202088245

A 0 0

G 4.248145651 0.370869858

G 7.005725834 0.269450994

G 8.368271005 0.553412712

C 14.90921206 0.59970015

C 6.063634849 0.86623355

A 0 0

C 2.526420079 0.495376486

A 0 0

G 7.12654925 0.277234181

C 10.79720734 0.340964442

C 8.126009693 0.56542811

C 9.953368709 0.225116578

C 4.27594977 0.476871722

T 0 0

G 2.247191011 0.506409242

G 3.617122098 0.347496446

A 0 0

G 3.979542912 0.271695701

A 0 0

C 9.822428411 0.335946249

C 6.626314113 0.477859191

C 7.218784706 0.650483897

C 3.541931385 0.127064803

T 0 0

T 0 0

G 0.445859873 0.159235669

A 0 0

G 2.852589641 0.079681275

C 2.722929936 0.159235669

T 0 0

G 0.545834002 0.224755177

A 0 0

G 0 0

T 0 0

G 1.437470053 0.271522121

C 1.723037652 0.366943204

T 0 0

T 0 0

T 0 0

G 0.770198051 0.172901603

T 0 0

C 3.335431089 0.204531152

C 2.074493164 0.282885431

T 0 0

T 0 0

G 1.439073987 0.125136868

C 3.110346983 0.171928728

A 0 0

T 0 0

A 0 0

C 2.047514848 0.46889653

T 0 0

C 0.639226692 0.420954163

T 0 0

T 0 0

T 0 0

C 0.308024026 0.154012013

T 0 0

G 1.427475058 0.076745971

G 5.96512695 0.244723157

C 7.098152954 0.564799267

C 3.245063524 0.336751875

T 0 0

C 1.564398542 0.22782503

A 0 0

T 0 0

A 0 0

G 2.08928303 0.195400571

T 0 0

G 3.300882263 0.167325829

G 4.288321168 0.197688564

G 5.138699409 0.106108837

G 6.242442563 0.196493349

C 9.280532044 0.196493349

T 0 0

T 0 0

G 2.227647238 0.045773573

G 5.235042735 0.305250305

C 4.007313728 0.380923358

C 3.230230078 0.35044949

A 0 0

T 0 0

T 0 0

G 2.412909063 0.286532951

T 0 0

C 5.105421687 0.481927711

C 5.310001509 0.211193242

C 2.908378541 0.120554551

T 0 0

T 0 0

C 0.33067789 0.04509244

A 0 0

C 1.49970006 0.059988002

T 0 0

C 2.10192046 0.257069409

C 1.408024224 0.302800908

A 0 0

G 0.549534422 0.488475042

A 0 0

T 0 0

C 0.610128127 0.228798048

T 0 0

C 1.641607855 0.184105554

T 0 0

C 3.521126761 0.260257195

C 1.893997251 0.595692684

T 0 0

T 0 0

T 0 0

C 0.076034063 0.045620438

A 0 0

G 0.426894344 0.243939625

G 52.02743902 0.182926829

T 0 0

C 1.501592598 0.15167602

C 0.954111767 0.106012419

A 0 0

G 0.939251629 0.196939858

G 2.078592019 0.106205432

A 0 0

A 0 0

G 0.893820311 0.09246417

T 0 0

G 0.66542866 0.077375426

C 1.839258114 0.061823802

A 0 0

T 0 0

C 0.524691358 0.046296296

T 0 0

T 0 0

G 0 0

A 0 0

A 0 0

C 0.496124031 0.07751938

T 0 0

T 0 0

A 0 0

A 0 0

C 0.402913374 0.17046335

T 0 0

T 0 0

T 0 0

C 1.384615385 0.123076923

C 0.924214418 0.154035736

A 0 0

G 1.265041654 0.061709349

A 0 0

C 9.507897934 0.19744836

C 5.104373 0.121895475

C 5.248703082 0.091547147

C 55.79588728 0.076161462

C 8.090909091 0.151515152

C 7.669036454 0.015126305

C 3.131145061 0.090757828

T 0 0

T 0 0

C 0.273431566 0.091143855

A 0 0

G 0.400863398 0.246685168

T 0 0

T 0 0

T 0 0

T 0 0

C 0.585786959 0.169569909

C 0.522916026 0.169178714

A 0 0

G 1.13811135 0.076899416

T 0 0

C 3.048688002 0.030335204

C 1.425754588 0.091005612

T 0 0

T 0 0

A 0 0

G 0.423792947 0.151354624

A 0 0

G 0.411271896 0.380807312

A 0 0

G 1.104542626 0.700062228

G 5.989421282 0.062227754

T 0 0

G 1.674753483 0.125215214

G 1.458823529 0.407843137

A 0 0

C 1.386918834 0.315208826

T 0 0

T 0 0

C 0.235294118 0.282352941

T 0 0

G 0.173913043 0.18972332

A 0 0

T 0 0

T 0 0

C 3.75880971 0.125293657

C 1.593501015 0.249960944

T 0 0

T 0 0

T 0 0

G 0.950896337 0.093530787

T 0 0

C 1.193243453 0.402913374

T 0 0

C 0.491249616 0.353085662

T 0 0

G 1.946176068 0.106431504

T 0 0

G 4.572473708 0.121932632

C 8.442150015 0.622532645

C 7.058107697 0.15211439

C 3.486792453 0.422641509

T 0 0

G 2.590909091 0.348484848

T 0 0

A 0 0

G 4.914232731 0.355431927

C 7.487623762 1.051980198

C 1.503642846 0.573554488

T 0 0

C 0.465044179 0.341032398

A 0 0

G 1.320696085 0.435052828

G 5.181832371 0.202903075

T 0 0

C 0.55083412 0.661000944

A 0 0

G 0.919003115 0.529595016

G 4.173179118 0.422006877

C 5.010927256 0.327817671

T 0 0

T 0 0

A 0 0

A 0 0

G 1.655732584 0.265542018

G 4.274976511 0.125274037

C 2.59965338 0.598708051

A 0 0

A 0 0

G 1.546063118 0.796939751

G 6.040688576 1.142410016

T 0 0

C 1.977139327 0.293481619

T 0 0

C 4.717126561 0.416217049

C 2.797417768 0.39963111

T 0 0

C 0.944402133 0.12185834

A 0 0

C 4.557192769 0.258240924

C 0.998157248 2.057739558

T 0 0

G 2.425172678 0.245587107

G 6.090133983 0.837393423

C 6.066565809 1.316187595

C 2.596618357 0.332125604

T 0 0

G 2.934537246 1.053423627

G 4.988696307 0.663149962

G 7.318545345 0.467783311

G 5.061952251 0.423088546

A 0 0

G 1.424414152 0.490120999

A 0 0

G 2.411109416 0.549366702

T 0 0

C 3.664841849 0.714720195

C 3.586081143 0.82054399

C 2.295181639 0.699194406

A 0 0

G 1.350121359 0.530946602

G 2.971654983 0.243828101

A 0 0

C 0.296042381 0.405110626

G 0 0

C 0.759974668 0.443318556

T 0 0

G 1.417992752 0.346620451

C 0.803529226 0.2520876

A 0 0

C 0.812246173 0.281162137

G 0 0

T 0 0

G 3.940428173 0.232702451

C 5.144544607 0.497357787

C 1.303538175 0.512104283

T 0 0

G 0.837209302 0.093023256

T 0 0

G 0.603434937 0.154726907

C 0.401854714 0.355486862

G 0 0

G 2.22358534 0.322036497

G 11.09406168 0.092066902

T 0 0

A 0 0

G 2.283950617 0.154320988

G 3.490183858 0.233717669

A 0 0

T 0 0

G 1.769218726 0.10959762

C 1.112852665 0.109717868

T 0 0

G 0.252964427 0.537549407

A 0 0

T 0 0

G 3.158730159 0

C 5.057872205 0.142698589

C 2.596991291 0.158353127

C 1.155794807 0.253324889

A 0 0

G 0.285986654 0.190657769

A 0 0

T 0 0

T 0 0

T 0 0

C 1.518299505 0.175803101

C 0.972111554 0.398406375

C 0.270830014 0.127449419

G 0 0

T 0 0

T 0 0

A 0 0

G 0.959692898 0.111964171

A 0 0

G 0.781998085 0.159591446

A 0 0

G 5.434439179 0.142180095

C 5.469977994 0.565859793

C 1.895363409 0.344611529

T 0 0

T 0 0

T 0 0

C 4.859727119 0.475241453

C 5.009191176 0.168504902

C 1.956289164 0.290386673

T 0 0

A 0 0

T 0 0

C 2.126682987 0.107099143

C 1.148193509 0.183710961

T 0 0

G 0.263893201 0.077615647

A 0 0

C 0.231053604 0.292667899

G 0 0

G 2.277273427 0.430835513

C 2.850100139 0.09243568

T 0 0

C 0.696163366 0.232054455

T 0 0

A 0 0

G 2.478829869 0.107775212

C 2.890973397 0.384437952

T 0 0

T 0 0

T 0 0

G 0.619770685 0.077471336

T 0 0

G 0.620347395 0.170595533

T 0 0

G 0.979477612 0.248756219

T 0 0

T 0 0

A 0 0

C 2.033898305 0.077041602

T 0 0

T 0 0

A 0 0

C 1.426356589 0.031007752

T 0 0

T 0 0

G 1.387157107 0.374064838

T 0 0

T 0 0

C 1.483447845 0.202998126

C 0.562939797 0.218921032

A 0 0

C 0.84217093 0.202744853

T 0 0

T 0 0

T 0 0

A 0 0

A 0 0

T 0 0

T 0 0

C 0.297619048 0.140977444

A 0 0

A 0 0

A 0 0

A 0 0

T 0 0

G 5.352684145 0.124843945

T 0 0

A 0 0

C 8.864696734 0.139968896

C 2.71950272 0.248640249

C 0.825031133 0.155666252

A 0 0

G 1.594497421 0.031264655

C 1.032056294 0.125097733

A 0 0

A 0 0

C 2.110034386 0.171928728

C 0.406313486 0.17190186

A 0 0

G 3.523884103 0.187940486

C 3.121585766 0.093647573

T 0 0

T 0 0

G 1.231488698 0.483242401

T 0 0

G 1.284259984 0.078308536

C 1.598495534 0.109700674

A 0 0

C 1.318681319 0.062794349

A 0 0

G 0.61157284 0.297945743

T 0 0

T 0 0

C 0.688899327 0.172224832

T 0 0

C 1.045894474 0.093662192

T 0 0

G 1.433691756 0.233754091

G 4.092471103 0.577944392

G 3.110198926 0.52099779

G 6.699398163 0.095026924

T 0 0

T 0 0

T 0 0

C 0.429048149 0.429048149

A 0 0

G 1.232616941 0.316055626

G 3.528481013 0.189873418

A 0 0

G 3.078403078 0.721500722

G 6.381620932 0.047862157

G 4.099793421 1.001112347

A 0 0

T 0 0

G 2.92867265 0.314911038

T 0 0

A 0 0

A 0 0

G 1.393673661 0.344503602

A 0 0

C 4.865034526 0.219711237

A 0 0

T 0 0

A 0 0

C 10.15501424 0.094906675

C 5.906637028 0.41282947

C 6.819265618 0.07947862

C 3.49023375 0.240153698

T 0 0

T 0 0

G 4.489664083 0.387596899

C 6.465168903 0.258606756

C 5.319493999 0.486539085

C 2.938311688 0.12987013

T 0 0

T 0 0

C 0.727331502 0.210117989

A 0 0

G 0.926527958 0.633940182

G 6.282637076 0.424281984

C 2.324439352 0.147323621

A 0 0

C 1.528952505 1.057254392

T 0 0

A 0 0

T 0 0

G 4.937867888 0.228907783

G 4.584151932 0.68762279

C 4.530531845 0.705843729

C 1.689355421 0.475643759

A 0 0

G 1.075098814 0.743083004

A 0 0

A 0 0

G 7.350157729 0.299684543

G 14.78494624 0.379506641

G 16.79848149 0.616893388

G 14.8177496 0.348652932

G 11.94928685 0.348652932

G 15.1822504 0.824088748

C 7.488424078 0.255468625

A 0 0

G 1.560115313 0.237408852

T 0 0

G 1.310344828 1.206896552

A 0 0

C 3.598971722 0.119965724

C 2.393980848 0.598495212

T 0 0

A 0 0

G 1.980700863 1.828339259

G 7.070876722 0.335908633

C 2.265480785 0.134250713

A 0 0

G 0.968893422 0.560938297

A 0 0

G 2.733640868 0.939689048

G 5.723560567 1.725610798

G 7.148965281 1.693175988

C 0.275103164 0.137551582

G 0 0

G 1.94905112 1.726790904

G 4.515021459 0.25751073

A 0 0

G 3.255253186 0.275576989

C 2.392838699 1.652608022

C 1.193317422 0.153426526

A 0 0

G 0.676475562 0.118383223

C 0.828822733 0.947225981

A 0 0

G 0.650325163 0.20010005

A 0 0

T 0 0

G 2.625893302 0.166195779

G 4.349264098 0.413428146

G 4.502721425 0.593765463

A 0 0

T 0 0

A 0 0

C 1.74571805 0.576416337

A 0 0

C 1.530360375 0.362020734

T 0 0

C 0.508446777 0.14761358

A 0 0

G 0.970235159 0.476895247

A 0 0

G 3.19567355 0.557194363

G 6.64915449 0.197011985

A 0 0

G 7.507408627 0.345735924

C 5.224248398 0.213569903

C 1.42296369 0.457965325

T 0 0

G 0 0

C 1.081517353 0.17756255

A 0 0

G 1.272141707 0.225442834

C 1.476962594 0.369240649

A 0 0

G 1.035526525 0.477935319

G 5.830023828 0.397140588

C 1.842147054 0.269969827

A 0 0

G 1.031418597 0.380831482

A 0 0

G 1.828298887 0.635930048

G 7.121284375 0.317914481

C 2.795869738 0.571882446

A 0 0

G 0.902041462 0.506409242

A 0 0

G 2.996548478 0.454973329

G 4.714016342 0.565681961

A 0 0

G 2.618045816 0.607760636

A 0 0

A 0 0

G 3.642590286 0.077833126

G 7.48796023 0.264098182

G 6.838269499 0.310125601

A 0 0

G 1.584096909 0.310607237

G 5.741995074 0.200123153

T 0 0

C 1.183063512 0.420298879

T 0 0

A 0 0

C 2.64262397 0.093269081

A 0 0

C 0.588326366 0.418021366

G 1.112484549 0.123609394

T 0 0

T 0 0

C 0.508709727 0.369970711

T 0 0

G 1.042305334 0.061312078

C 0.965961362 0.18399264

A 0 0

C 1.143292683 0.335365854

T 0 0

G 1.544050863 0.015137754

T 0 0

A 0 0

T 0 0

T 0 0

T 0 0

A 0 0

T 0 0

C 0.997280145 0.211544273

T 0 0

C 3.246753247 0.216450216

C 1.821831095 0.370541918

T 0 0

T 0 0

C 0.521312481 0.321987121

A 0 0

G 0.368437212 0.245624808

T 0 0

T 0 0

C 1.058444547 0.398834177

C 0.783289817 0.445400092

A 0 0

A 0 0

G 0.735632184 0.275862069

G 4.477383863 0.305623472

T 0 0

T 0 0

C 0.822919842 0.502895459

T 0 0

C 0.693397648 0.361772686

T 0 0

C 2.875446961 0.148986889

C 0.906389302 1.158989599

T 0 0

G 1.324799047 0.133968443

G 2.793047096 0.222849502

C 1.899109792 0.548961424

A 0 0

T 0 0

C 0.68401487 0.237918216

T 0 0

A 0 0

T 0 0

A 0 0

T 0 0

T 0 0

G 2.322424974 0.09048409

C 1.313009357 0.256565047

T 0 0

C 1.091405184 0.136425648

A 0 0

T 0 0

G 0.911854103 0.106382979

A 0 0

G 0.899253163 0.15241579

T 0 0

T 0 0

A 0 0

C 1.468563561 0.214165519

A 0 0

G 0.611807892 0.16824717

A 0 0

G 0.939617991 0.16943931

C 0.755589823 0.262143408

A 0 0

A 0 0

A 0 0

G 5.61468456 0.06169983

C 4.551766703 0.354883506

C 1.298902118 0.046389361

T 0 0

G 0.914445133 0.232486051

G 3.301813672 0.093008836

T 0 0

G 0.640424867 0.156201187

T 0 0

G 0.693896862 0.126163066

A 0 0

T 0 0

G 0.884676145 0.18957346

G 2.654867257 0.268647282

T 0 0

T 0 0

A 0 0

C 0.824742268 0.015860428

T 0 0

T 0 0

T 0 0

T 0 0

A 0 0

G 1.265022138 0.91714105

G 4.553359684 0.600790514

T 0 0

G 0.703212402 0.0159821

T 0 0

C 0.863861782 0.175971845

A 0 0

A 0 0

C 0.607611129 0.687559962

T 0 0

T 0 0

G 0.899598394 0.096385542

G 2.9889121 0.144624779

C 1.916572717 0.016105653

T 0 0

G 0.630965863 0.080893059

G 1.408222726 0.275169958

A 0 0

T 0 0

T 0 0

A 0 0

A 0 0

T 0 0

A 0 0

A 0 0

A 0 0

T 0 0

A 0 0

C 3.714661407 0.03287311

C 0.910746812 0.132472264

T 0 0

A 0 0

G 0.979090607 0.116163292

A 0 0

G 0.383781078 0.283664275

A 0 0

A 0 0

C 0.521973396 0.286243475

T 0 0

G 1.069609508 0.118845501

G 3.153611394 0.0508647

T 0 0

A 0 0

A 0 0

A 0 0

G 1.771418838 0.119230114

C 4.891397298 0.15392509

A 0 0

T 0 0

T 0 0

A 0 0

T 0 0

T 0 0

T 0 0

C 0.451232211 0.156195765

T 0 0

G 0.718919867 0.105207785

G 1.828410689 0.562587904

G 7.45900194 0.405572209

T 0 0

G 0.503144654 0.161725067

T 0 0

G 0.761145343 0.416817688

T 0 0

T 0 0

T 0 0

G 0 0

T 0 0

G 0 0

A 0 0

A 0 0

G 1.187384045 0.890538033

G 7.497674419 0.334883721

T 0 0

G 0.635989525 0.149644594

T 0 0

T 0 0

T 0 0

C 1.537307837 0.318710161

C 0.786369594 0.33701554

A 0 0

G 0.926626324 0.113464448

A 0 0

G 1.699446248 0.91655528

G 4.585571757 0.268610898

A 0 0

G 1.065891473 0.271317829

A 0 0

T 0 0

T 0 0

G 1.03880831 0.156801254

G 2.425557089 0.453559456

C 1.463897132 0.21760633

T 0 0

G 0.617899143 0.119593382

T 0 0

G 0 0

A 0 0

G 1.397326853 0.141757797

T 0 0

C 0.867231055 0.144538509

A 0 0

G 1.0627214 0.166701396

T 0 0

G 1.806722689 0.18907563

G 2.934966216 0.04222973

G 6.772486772 0.253968254

C 5.011679762 0.148651518

T 0 0

G 0.432525952 0.08650519

A 0 0

G 1.71701804 0.19560965

T 0 0

G 3.74749052 0.289984385

G 6.580125336 0.470008953

G 7.799819657 0.428313796

G 8.261656858 0.384789498

A 0 0

G 3.7470726 0.351288056

G 5.065088757 0.473372781

A 0 0

G 3.44384477 1.053713698

C 2.225097025 0.232858991

T 0 0

G 4.02360515 0.18776824

C 7.275666936 0.161681487

C 5.974587726 0.108137334

C 10.55058313 0.352590182

T 0 0

C 3.659202622 0.546149645

C 10.09961262 0.747094632

A 0 0

T 0 0

G 2.421171171 0.929054054

T 0 0

G 3.131716303 0.675468222

G 3.2675709 0.493218249

G 7.80141844 0.77089115

C 3.269412135 0.06287331

A 0 0

G 1.816412585 0.778462536

G 5.178629957 1.081612586

C 3.468780971 0.42946812

A 0 0

C 4.138627187 1.547779273

C 3.046218487 1.680672269

A 0 0

T 0 0

C 2.852049911 1.140819964

C 2.84992785 0.072150072

A 0 0

T 0 0

T 0 0

G 0.415251038 1.359003398

A 0 0

C 1.075268817 0

T 0 0

G 4.269752594 1.835594573

G 7.704654896 0.922953451

G 14.03861625 1.36765889

C 13.71359223 0

C 9.208924949 0

C 6.446280992 0.537190083

A 0 0

G 0.43047783 1.851054671

A 0 0

T 0 0

A 0 0

G 0.394866732 3.15893386

A 0 0

A 0 0

C 1.060070671 0.504795558

A 0 0

A 0 0

G 0.669757857 0.309119011

A 0 0

A 0 0

G 2.548806941 0.162689805

G 5.046120456 0.271296799

C 2.339499456 0.054406964

A 0 0

G 0.774336283 0.221238938

A 0 0

A 0 0

G 0.748416811 0.057570524

A 0 0

A 0 0

A 0 0

T 0 0

G 1.060070671 0.058892815

T 0 0

G 0.178677784 0

A 0 0

A 0 0

T 0 0

T 0 0

C 2.53776435 0

C 1.026570048 0

T 0 0

C 0.242718447 0.060679612

T 0 0

T 0 0

T 0 0

C 0.431034483 0

T 0 0

C 0.941619586 0

T 0 0

G 0.892857143 0.12755102

C 0.702426564 0.06385696

T 0 0

G 1.494476933 0.259909032

G 2.426229508 0.131147541

A 0 0

G 3.306878307 0.066137566

C 2.280348759 0

T 0 0

G 0.2734108 0.2050581

G 2.613480055 0.275103164

G 2.548209366 0.619834711

A 0 0

T 0 0

A 0 0

T 0 0

T 0 0

C 0.650759219 0.28922632

T 0 0

T 0 0

C 0.673148841 0.074794316

T 0 0

T 0 0

C 0.851393189 0.154798762

T 0 0

C 5.663430421 0.404530744

C 3.621399177 0.164609053

T 0 0

G 5.630252101 0.420168067

C 6.86440678 0.084745763

C 7.142857143 0

C 3.720106289 0.177147919

T 0 0

T 0 0

G 0 0

G 2.576335878 3.339694656

A 0 0

C 2.152641879 0

A 0 0

T 0 0

C 0.210304942 0

A 0 0

G 0.431965443 0.323974082

A 0 0

G 5.088495575 0.442477876

C 1.789709172 0.111856823

T 0 0

G 1.50462963 1.157407407

C 1.635514019 1.285046729

A 0 0

G 1.70109356 1.093560146

G 8.531994981 0.501882058

C 6.463878327 0.633713561

T 0 0

C 1.470588235 0.534759358

T 0 0

C 2.103786816 0.561009818

T 0 0

G 3.988183161 0.295420975

G 7.898658718 1.490312966

C 11.09422492 0.607902736

C 3.120124805 0.15600624

T NA NA

T NA NA

T NA NA

G NA NA

G NA NA

A NA NA

C NA NA

C NA NA

C NA NA

G NA NA

A NA NA

G NA NA

G NA NA

A PRIMER NA NA

T PRIMER NA NA

T PRIMER NA NA

T PRIMER NA NA

A PRIMER NA NA

T PRIMER NA NA

A PRIMER NA NA

C PRIMER NA NA

C PRIMER NA NA

A PRIMER NA NA

A PRIMER NA NA

G PRIMER NA NA

C PRIMER NA NA

A PRIMER NA NA

G PRIMER NA NA

G PRIMER NA NA

T PRIMER NA NA

T PRIMER NA NA

T PRIMER NA NA

C PRIMER NA NA

T PRIMER NA NA

G PRIMER NA NA

G PRIMER NA NA

G PRIMER NA NA

T PRIMER NA NA

T PRIMER NA NA

C NA NA

T NA NA

C NA NA

A NA NA

G NA NA

G NA NA

C NA NA

C NA NA

T NA NA

T NA NA

T NA NA

G NA NA

G NA NA

C NA NA

C NA NA

T NA NA

T NA NA

G NA NA

G NA NA

A NA NA

C NA NA

T NA NA

G NA NA

A NA NA

T NA NA

A NA NA

A NA NA

G NA NA

G NA NA

A NA NA

C NA NA

T NA NA

T NA NA

A NA NA

A NA NA

C NA NA

T NA NA

C NA NA

A NA NA

T NA NA

G NA NA

T NA NA

A NA NA

A NA NA

A NA NA

A NA NA

T NA NA

A NA NA

T NA NA

A NA NA

T NA NA

A NA NA

G NA NA

A NA NA

A NA NA

T NA NA

T NA NA

G NA NA

T PRIMER NA NA

G PRIMER NA NA

T PRIMER NA NA

T PRIMER NA NA

T PRIMER NA NA

G PRIMER NA NA

T PRIMER NA NA

T PRIMER NA NA

G PRIMER NA NA

C PRIMER NA NA

A PRIMER NA NA

A PRIMER NA NA

T PRIMER NA NA

A PRIMER NA NA

G PRIMER NA NA

G PRIMER NA NA

A PRIMER NA NA

A PRIMER NA NA

G PRIMER NA NA

A PRIMER NA NA

A PRIMER NA NA

C PRIMER NA NA

T PRIMER NA NA

T PRIMER NA NA

G PRIMER NA NA

A 0 0

T 0 0

A 0 0

C 1.276024177 0.067159167

A 0 0

G 0.984251969 0.196850394

G 5.029392554 0.261267146

T 0 0

G 0.453661698 0

A 0 0

A 0 0

T 0 0

T 0 0

T 0 0

T 0 0

T 0 0

A 0 0

C 0.689223058 0.125313283

T 0 0

A 0 0

A 0 0

G 0.411764706 0

A 0 0

A 0 0

T 0 0

T 0 0

A 0 0

C 2.203389831 0.734463277

T 0 0

A 0 0

T 0 0

C 0.664819945 0.498614958

A 0 0

T 0 0

T 0 0

T 0 0

C 1.980700863 0.30472321

C 1.611278953 0.503524673

A 0 0

G 2.082324455 0.04842615

G 6.57322378 0.241662639

C 4.679208876 0.241196334

A 0 0

C 2.375296912 0.237529691

A 0 0

G 0.649651972 0.092807425

A 0 0

C 1.478743068 0.415896488

A 0 0

A 0 0

A 0 0

G 0.811907984 0.180423996

A 0 0

C 0.938757264 0.178810907

A 0 0

A 0 0

A 0 0

A 0 0

G 0.73402418 0.043177893

T 0 0

A 0 0

T 0 0

C 0.465707028 0.16934801

A 0 0

C 1.449875725 0.372825186

A 0 0

A 0 0

T 0 0

T 0 0

C 0.279776179 0.799360512

A 0 0

T 0 0

T 0 0

C 0.301204819 1.280120482

T 0 0

G 0.964519463 0

T 0 0

A 0 0

A 0 0

T 0 0

A 0 0

T 0 0

A 0 0

T 0 0

A 0 0

G 1.820425795 0.678802839

T 0 0

T 0 0

G 2.02957379 0.666859959

T 0 0

A 0 0

T 0 0

G 2.569960023 0.713877784

A 0 0

G 2.327514547 0.027708507

T 0 0

C 1.22984422 0.409948073

T 0 0

A 0 0

T 0 0

T 0 0

C 0.596473029 0.648340249

T 0 0

C 0.670794634 0.515995872

A 0 0

C 3.096446701 1.091370558

A 0 0

T 0 0

T 0 0

G 3.125 0.87594697

C 4.215732454 0.965614696

T 0 0

A 0 0

T 0 0

A 0 0

G 0.745594216 0.112968821

A 0 0

G 0.426391382 0.201974865

A 0 0

A 0 0

A 0 0

T 0 0

A 0 0

C 7.057119205 0.082781457

C 1.053066281 0.330373735

T 0 0

G 1.519195237 0.123177992

A 0 0

G 0.953346856 0.081135903

A 0 0

C 1.987676406 0.059630292

T 0 0

G 1.566477384 0.176228706

G 2.672649239 0.156067109

G 8.266718659 0

T 0 0

A 0 0

A 0 0

T 0 0

T 0 0

T 0 0

A 0 0

T 0 0

A 0 0

A 0 0

G 1.429115854 0

G 3.075754699 0.05695842

A 0 0

A 0 0

A 0 0

A 0 0

G 0.643573727 0.056785917

A 0 0

G 0.939672994 0.150347679

G 5.125797972 0.037551633

T 0 0

T 0 0

A 0 0

A 0 0

A 0 0

T 0 0

T 0 0

G 1.098901099 0.054945055

G 3.118161926 0.328227571

C 5.142231947 0.091174325

T 0 0

C 1.267656646 0.090546903

A 0 0

T 0 0

G 1.472414405 0.141919461

G 2.385512798 0.261187533

T 0 0

T 0 0

C 1.244247486 0.664734958

T 0 0

G 1.012487344 0.016874789

C 1.281835048 0.624051273

A 0 0

G 1.28440367 0.016680567

G 6.535511837 0.233411137

C 6.931348222 0.496277916

T 0 0

G 2.104327208 0

T 0 0

A 0 0

T 0 0

G 8.916642463 0.217833285

G 8.942155878 0.172240563

G 9.145642202 0.215022936

A 0 0

A 0 0

C 9.275403744 0.157210233

C 1.80102916 0.171526587

T 0 0

T 0 0

C 0.225098481 0.14068655

G 0.084554679 0.43686584

G 0 0

G 4.306693025 0.48009037

G 8.268660928 0.155213772

C 0.395480226 0.197740113

G 0.225956786 0.437791272

G 7.636261735 0.028022979

C 9.602463606 0.349944009

C 4.074844075 0.457380457

T 0 0

C 0.93445101 0.398515872

A 0 0

G 1.535298149 0.287868403

G 3.495065789 0.109649123

A 0 0

A 0 0

A 0 0

C 6.759334969 0.109021532

C 1.119912592 0.245834471

T 0 0

T 0 0

C 0.737201365 0.19112628

A 0 0

A 0 0

T 0 0

C 0.779989242 0.174825175

G 0 0

T 0 0

G 0.988402741 0.158144439

G 2.767892448 0.171345723

C 1.423675191 0.290007909

A 0 0

G 0.733272227 0.052376588

A 0 0

A 0 0

G 1.602311531 0.354609929

G 6.761006289 0.039308176

T 0 0

G 0 0

A 0 0

A 0 0

A 0 0

G 4.361132364 0.229533282

G 10.2284264 0.164974619

G 10.18589254 0.140056022

G 8.801213961 0.139099646

A 0 0

G 4.181841015 0.100464649

C 4.986843754 0.112767824

T 0 0

G 1.355081556 0.288582183

G 4.861546172 0.55130936

T 0 0

G 0.665244132 0.263587298

C 0.488721805 0.125313283

G 0.100300903 0.777331996

T 0 0

C 0.915590117 0.639658849

T 0 0

T 0 0

A 0 0

C 3.852889667 0.06254691

A 0 0

T 0 0

G 4.009020296 0.413430218

G 5.315281434 0.488905604

C 7.771371271 0.388568564

C 2.330535021 0.087708307

A 0 0

G 0.91650973 0.150659134

A 0 0

G 1.172022684 0.189035917

C 2.001258653 0.062932662

A 0 0

A 0 0

A 0 0

A 0 0

G 1.189283926 0.075112669

C 1.993730408 0.137931034

A 0 0

G 2.344827586 0.163009404

G 5.681961031 0.201131364

A 0 0

G 3.787116256 0.176144942

G 6.601405622 0.138052209

G 11.13345062 0.037697914

T 0 0

C 3.763576661 0.113665067

A 0 0

G 2.872914314 0.050181909

G 6.537591149 0.050289163

G 6.880040323 0.201612903

A 0 0

G 3.073434941 0.478649704

G 8.428733174 0.150962385

T 0 0

G 3.155768745 0.315576875

C 4.51156325 0.088462025

C 2.517075639 0.139134834

A 0 0

C 2.832825203 0.101626016

A 0 0

C 2.121982211 0.025412961

A 0 0

C 5.073566717 0.012683917

A 0 0

T 0 0

T 0 0

T 0 0

A 0 0

A 0 0

A 0 0

C 0.113895216 0.075930144

G 0 0

A 0 0

C 3.277651228 0.177170337

C 1.189421739 0.417562951

A 0 0

G 0.63500127 0.114300229

A 0 0

T 0 0

C 0.618999495 0.353713997

T 0 0

C 0.313165477 0.488538144

G 49.9311381 0.513334168

C 0.07523511 0.326018809

G 0 0

A 0 0

G 0.577962056 0.288981028

A 0 0

A 0 0

C 1.555834379 0.35131744

T 0 0

C 0.752351097 0.163009404

A 0 0

C 5.286895515 0.613881233

T 0 0

A 0 0

T 0 0

T 0 0

G 0.401253918 0.313479624

C 0.364596429 0.339451848

G 0.062853551 0.226272784

A 0 0

G 0.616740088 0.264317181

A 0 0

A 0 0

C 0.952261621 0.288184438

A 0 0

G 2.873995984 0.125502008

T 0 0

A 0 0

C 6.543813464 0.22564874

C 2.62760875 0.113150616

A 0 0

A 0 0

G 1.464091884 0.113593336

A 0 0

G 2.87961436 0.114169732

G 6.981781119 0.140145241

A 0 0

A 0 0

G 2.999110433 0.216037616

G 10.27767307 0.314109813

T 0 0

A 0 0

C 4.795647223 0.341642414

T 0 0

A 0 0

A 0 0

A 0 0

C 4.148166942 0.012685526

C 3.397302905 0.18153527

A 0 0

T 0 0

T 0 0

C 0.672790788 0.038814853

A 0 0

T 0 0

G 0.508408291 0.23464998

A 0 0

G 0.366108787 0.366108787

A 0 0

A 0 0

A 0 0

T 0 0

T 0 0

C 0.342330481 0.144832126

A 0 0

C 1.559020045 0.262020176

T 0 0

C 5.36433303 0.064943499

C 4.589780263 0.195033156

C 2.441572007 0.169734952

A 0 0

T 0 0

G 0.652145559 0.182600756

A 0 0

T 0 0

C 1.664715828 0.533229289

C 1.368434771 0.273686954

A 0 0

A 0 0

C 4.69953775 0.333846944

C 2.327076369 0.462843919

A 0 0

C 9.088626663 0.150640221

C 3.530594296 0.226159065

T 0 0

C 8.79258517 0.388276553

C 7.099773813 0.175923599

C 3.947699271 0.125722907

A 0 0

C 5.847733534 0.125219133

C 3.450881612 0.277078086

A 0 0

G 3.894440174 0.422751729

G 8.911145352 0.295326143

C 11.29943503 0.74473549

C 5.823863636 0.232438017

A 0 0

C 3.06724846 0.256673511

A 0 0

C 11.00545201 0.101432737

C 3.199593702 0.31742001

T 0 0

C 3.69050649 0.11453296

C 2.724726254 0.08912656

A 0 0

A 0 0

C 1.749271137 0.2281658

A 0 0

C 2.21571374 0.089137909

T 0 0

G 3.075525564 0.11679211

G 5.181616977 0.663976045

G 5.678562126 0.625162803

G 4.371584699 0.858704137

A 0 0

T 0 0

T 0 0

A 0 0

C 1.989721966 0.092238767

A 0 0

A 0 0

T 0 0

T 0 0

C 0.316413975 0.171390903

A 0 0

A 0 0

C 1.965151317 0.078606053

A 0 0

T 0 0

C 0.065172054 0.286757039

G 0 0

G 0.295402004 0.218340611

A 0 0

T 0 0

T 0 0

T 0 0

G 1.771703366 0.253100481

G 3.408094224 0.639017667

G 9.025769956 0.075424261

T 0 0

G 4.552865922 0.225761947

G 7.903650734 0.23836407

G 9.578207381 0.125533517

G 8.365783193 0.326592137

A 0 0

C 8.288401254 0.200626959

A 0 0

C 1.223203026 0.189155107

A 0 0

G 0.436513031 1.078443959

A 0 0

T 0 0

C 1.223911363 0.46379799

C 1.445197596 0.204629748

A 0 0

A 0 0

A 0 0

C 8.547979798 0.101010101

C 12.12814645 0.279684719

A 0 0

T 0 0

A 0 0

T 0 0

C 0.915184326 0.193348801

A 0 0

A 0 0

T 0 0

A 0 0

G 1.162490313 0.426246448

T 0 0

T 0 0

A 0 0

A 0 0

C 2.496442892 0.685551675

C 0.52807831 0.141679547

A 0 0

A 0 0

A 0 0

A 0 0

A 0 0

C 4.269865841 0.154798762

C 0.771505722 0.205734859

T 0 0

G 0.232108317 0.141843972

A 0 0

G 0.426521908 0.155098876

A 0 0

A 0 0

A 0 0

C 0.890092879 0.064499484

T 0 0

A 0 0

A 0 0

T 0 0

G 2.044989775 0.06390593

G 2.514679602 0.063824355

C 3.148903621 0.344212137

A 0 0

A 0 0

G 0.390035229 0.062908908

A 0 0

A 0 0

T 0 0

A 0 0

A 0 0

C 1.464035646 0.75111394

A 0 0

C 1.943817407 0.664660145

A 0 0

C 2.449804268 0.113650713

A 0 0

C 2.004791325 0.037826251

A 0 0

C 2.442709645 0.012591287

A 0 0

T 0 0

C 2.512247205 0.200979776

T 0 0

A 0 0

C 5.441749403 0.125675506

A 0 0

C 2.223626027 0.10107391

A 0 0

C 1.913380737 0.155138979

A 0 0

A 0 0

T 0 0

G 0.932090546 0.279627164

A 0 0

T 0 0

G 2.312834225 0.374331551

C 2.487694559 0

T 0 0

G 1.13436867 0.243079001

A 0 0

C 15.17325393 0.23010287

A 0 0

T 0 0

A 0 0

T 0 0

T 0 0

A 0 0

T 0 0

T 0 0

T 0 0

A 0 0

T 0 0

A 0 0

A 0 0

T 0 0

A 0 0

G 2.395128552 0.311231394

G 5.168690958 0.175438596

G 4.327183939 0.461204558

A 0 0

T 0 0

G 1.756535948 0.285947712

T 0 0

A 0 0

A 0 0

T 0 0

T 0 0

A 0 0

C 9.958448753 0.069252078

C 2.240354031 0.290416263

C 0.899031812 0.179806362

A 0 0

A 0 0

A 0 0

A 0 0

A 0 0

T 0 0

A 0 0

A 0 0

A 0 0

A 0 0

G 0.852669835 0.013978194

T 0 0

A 0 0

T 0 0

T 0 0

A 0 0

A 0 0

C 0.768478413 0.083834009

A 0 0

A 0 0

A 0 0

C 0.672457271 0.028019053

A 0 0

G 0.479954828 0.042348955

A 0 0

A 0 0

T 0 0

C 1.364275668 0.140646976

C 1.275224215 0.09809417

A 0 0

A 0 0

T 0 0

C 2.300124706 0.193986421

C 1.694681731 0.096445302

A 0 0

C 5.2946781 0.095276984

C 1.891671203 0.176918889

A 0 0

G 2.370249285 0.17708759

G 3.903903904 0.054600055

A 0 0

G 2.009358657 0.178915497

C 3.445424476 0.055126792

A 0 0

A 0 0

G 1.302841303 0.138600139

T 0 0

A 0 0

A 0 0

A 0 0

G 1.101352293 0.278823365

T 0 0

C 0.957611604 0.19715533

T 0 0

A 0 0

T 0 0

C 0.767045455 0.028409091

T 0 0

C 1.341428975 0.070601525

C 0.140924464 0.112739572

G 0.098563785 0.168966488

G 0.818168994 0.07053181

A 0 0

A 0 0

A 0 0

G 1.071630006 0.155104343

C 1.695394179 0.084769709

A 0 0

A 0 0

A 0 0

G 0.711035267 0.071103527

A 0 0

C 0.085482262 0.11397635

G 0.229193525 0.143245953

G 2.750212645 0.127587185

T 0 0

T 0 0

C 0.69267741 0.226180379

A 0 0

A 0 0

C 11.30737135 0.069541029

A 0 0

T 0 0

T 0 0

A 0 0

G 0.664635835 0.235391858

A 0 0

G 0.49833887 0.249169435

A 0 0

A 0 0

T 0 0

C 0.495049505 0.343784378

T 0 0

G 0.701706109 0.371491469

T 0 0

T 0 0

A 0 0

G 3.525248401 0.054443991

T 0 0

A 0 0

C 4.539877301 0.218132243

A 0 0

A 0 0

C 1.853901494 0.62257886

T 0 0

C 0.922865014 0.151515152

A 0 0

C 3.072511266 0.095589239

T 0 0

A 0 0

C 8.171839003 0.108415774

A 0 0

T 0 0

G 1.22118551 0.370472009

A 0 0

A 0 0

G 0.988002823 0.155257586

A 0 0

G 0.980533526 0.302811824

A 0 0

C 2.075170512 0.130605137

T 0 0

A 0 0

A 0 0

A 0 0

A 0 0

A 0 0

A 0 0

G 1.602941176 0.073529412

G 3.755729706 0.044359012

G 2.944658183 0.310742823

A 0 0

A 0 0

A 0 0

C 0.787401575 0.133709701

T 0 0

T 0 0

T 0 0

G 1.468213185 0.704742329

G 2.775737994 0.63151711

G 4.481339994 0.07346459

C 6.59808964 0.044085231

A 0 0

T 0 0

C 0.544277729 0.411885849

T 0 0

C 0.788321168 0.087591241

A 0 0

A 0 0

A 0 0

A 0 0

G 0.441595442 0.085470085

A 0 0

T 0 0

A 0 0

G 9.447194719 0.233773377

G 20.09844135 0.095706864

C 22.03992343 0.068362045

C 19.98085339 0.23249453

C 21.05623204 0.205226433

C 12.63215708 0.164767266

T 0 0

A 0 0

A 0 0

C 2.363219708 0.085935262

A 0 0

A 0 0

G 2.59334479 0.078112795

C 2.576112412 0.608899297

A 0 0

A 0 0

C 1.523719761 0.015708451

T 0 0

G 0.91540404 0.315656566

G 3.943839722 0.126202871

T 0 0

A 0 0

A 0 0

A 0 0

T 0 0

T 0 0

T 0 0

A 0 0

A 0 0

T 0 0

A 0 0

T 0 0

T 0 0

T 0 0

A 0 0

G 1.613160837 0.319437789

T 0 0

C 3.708419545 0.12732771

C 2.095903461 0.111146396

T 0 0

A 0 0

A 0 0

T 0 0

T 0 0

T 0 0

T 0 0

A 0 0

A 0 0

A 0 0

G 2.214993804 0.10842627

C 3.418803419 0.310800311

A 0 0

C 1.945345067 0.216149452

A 0 0

C 1.963190184 0.13803681

A 0 0

C 2.062481043 0.090991811

A 0 0

A 0 0

C 4.753336332 0.179937022

C 1.935483871 0.120030008

A 0 0

C 1.903477218 0.164868106

A 0 0

A 0 0

G 0.742536748 0.045461434

A 0 0

T 0 0

A 0 0

T 0 0

T 0 0

C 0.198110332 0.076196282

A 0 0

G 0.228484387 0.335110434

A 0 0

A 0 0

A 0 0

G 1.920438957 0.182898948

C 3.291177815 0.121895475

T 0 0

A 0 0

A 0 0

A 0 0

A 0 0

A 0 0

T 0 0

A 0 0

G 1.151189563 0.092095165

T 0 0

C 0.740055504 0.107924761

A 0 0

G 0.307834385 0.046175158

A 0 0

A 0 0

A 0 0

C 1.030610675 0.092293493

T 0 0

T 0 0

C 4.80024305 0.106334498

C 2.364352834 0.090936647

T 0 0

T 0 0

A 0 0

A 0 0

C 6.225386247 0.060587701

C 1.005944216 0.350556318

T 0 0

G 1.635297748 0.107991361

T 0 0

A 0 0

A 0 0

C 1.278733445 0.167453189

A 0 0

A 0 0

A 0 0

C 9.933171324 0.22782503

A 0 0

T 0 0

T 0 0

G 1.1895684 0.167759646

T 0 0

A 0 0

T 0 0

T 0 0

T 0 0

A 0 0

A 0 0

T 0 0

G 5.233184547 0.184700631

C 3.563165201 0.123399661

A 0 0

T 0 0

G 0.849158561 0.04631774

A 0 0

G 1.004016064 0.061785604

A 0 0

A 0 0

C 1.065637066 0.061776062

A 0 0

G 0.450870647 0.155472637

A 0 0

T 0 0

A 0 0

T 0 0

T 0 0

A 0 0

G 0.943989931 0.078665828

C 0.865732725 0.047221785

A 0 0

A 0 0

A 0 0

T 0 0

T 0 0

T 0 0

T 0 0

C 3.180322732 0.172332759

C 1.457223441 0.047007208

C 0.203125 0.03125

G 0.062470717 0.078088396

T 0 0

A 0 0

A 0 0

A 0 0

A 0 0

G 3.237466604 0.094295144

C 4.30479183 0.062843676

T 0 0

C 0.689115114 0.046985121

A 0 0

G 0.357475909 0.015542431

A 0 0

T 0 0

G 1.572718779 0.046714419

G 4.349181606 0.077942323

T 0 0

G 0.343053173 0.046779978

A 0 0

A 0 0

T 0 0

A 0 0

T 0 0

T 0 0

C 0.522482584 0.126662445

T 0 0

A 0 0

G 0.794407372 0.317762949

G 2.350698856 0.174714104

T 0 0

T 0 0

T 0 0

T 0 0

T 0 0

C 0.095465394 0.047732697

G 0.22264631 0.190839695

G 1.054987212 0.063938619

G 4.429465575 0.048146365

C 4.121231559 0.048107761

A 0 0

A 0 0

T 0 0

A 0 0

C 3.565258363 0.07852992

C 0.489113285 0.173556327

G 0 0

C 7.520453115 0.251730648

C 1.822466614 0.17282011

T 0 0

C 1.248634306 0.390198221

T 0 0

G 0.929656027 0.247908274

T 0 0

C 1.879173785 0.232955428

T 0 0

G 1.7380509 0.217256363

G 2.468944099 0.031055901

A 0 0

C 2.82083075 0.09299442

T 0 0

A 0 0

T 0 0

T 0 0

T 0 0

A 0 0

A 0 0

C 1.18159204 0.155472637

A 0 0

G 0 0

C 7.056387385 0.111500478

A 0 0

T 0 0

T 0 0

G 1.958307012 1.026531901

C 2.394924663 0.142743854

T 0 0

G 0.962772786 0.320924262

T 0 0

G 0.751020408 0.620408163

G 2.534936627 0.698732532

C 0.294599018 0.196399345

G 0 0

T 0 0

G 0.769482646 0.68762279

A 0 0

C 1.455437449 0.179885527

A 0 0

G 2.213247173 0.129240711

T 0 0

A 0 0

G 3.922209511 0.522961268

C 5.971363488 0.618288318

C 3.127035831 0.179153094

A 0 0

T 0 0

C 0.577176781 0.47823219

A 0 0

G 0.790713324 0.185060565

A 0 0

C 2.332214765 0.083892617

A 0 0

A 0 0

T 0 0

A 0 0

T 0 0

G 7.237936772 0.016638935

T 0 0

A 0 0

G 0.633438906 0.200033339

A 0 0

A 0 0

A 0 0

A 0 0

A 0 0

T 0 0

G 1.696367375 0.188485264

T 0 0

G 1.175268268 0.255493102

T 0 0

G 2.445236882 0.20376974

T 0 0

A 0 0

A 0 0

C 1.624915369 0.118483412

T 0 0

G 0.789699571 0.120171674

T 0 0

G 1.380153739 0.244584207

T 0 0

T 0 0

C 2.958683473 0.245098039

C 1.72201722 0.052714813

A 0 0

A 0 0

T 0 0

A 0 0

A 0 0

A 0 0

A 0 0

C 0.633598841 0.271542361

T 0 0

T 0 0

T 0 0

A 0 0

T 0 0

T 0 0

T 0 0

A 0 0

C 1.724770642 0.128440367

A 0 0

A 0 0

G 0.854856311 0.127319025

A 0 0

A 0 0

G 1.470855275 0.036317414

A 0 0

G 1.927623204 0.036370249

A 0 0

C 5.915441844 0.108873163

C 4.668125456 0.273522976

A 0 0

T 0 0

T 0 0

G 0.752846126 0.110172604

G 2.987276415 0.276599668

C 3.266888151 0.073827981

T 0 0

G 0.922679461 0.036907178

G 1.325344953 0.217864924

A 0 0

T 0 0

T 0 0

T 0 0

G 10.09094089 0.174886324

G 19.74533403 0.139542997

C 19.51815642 0.31424581

C 19.1519187 0.157701069

C 19.32758317 0.299243091

C 18.16881259 0.268240343

C 8.299052048 0.250402432

C 0.126490784 0.325262017

G 0.269154854 0.125605598

G 5.252814008 0.357334286

C 6.956993133 0.397542465

C 3.015529072 0.252798844

A 0 0

C 5.912918832 0.21501523

C 1.37745975 0.590339893

A 0 0

G 0.765260723 0.106780566

T 0 0

T 0 0

T 0 0

G 1.033683835 0.07128854

G 1.713979646 0.249955365

A 0 0

A 0 0

A 0 0

C 12.43367527 0.123806155

C 6.379953794 0.159943131

C 8.982356086 0.07128854

C 4.198541185 0.213485145

T 0 0

G 2.442067736 0.124777184

C 5.194805195 0.035580857

A 0 0

T 0 0

T 0 0

A 0 0

G 0.417271408 0.253991292

A 0 0

A 0 0

T 0 0

C 0.810163874 0.073651261

A 0 0

T 0 0

T 0 0

C 0.292986632 0.054934994

G 0 0

C 1.058587333 0.054754517

A 0 0

T 0 0

T 0 0

A 0 0

G 1.12522686 0.054446461

T 0 0

C 0.5613908 0.090546903

A 0 0

G 0.21691974 0.07230658

A 0 0

A 0 0

A 0 0

C 1.325104375 0.054456344

A 0 0

A 0 0

G 0.895304221 0.054814544

A 0 0

G 0.550256787 0.091709464

A 0 0

A 0 0

A 0 0

G 0.277161863 0.055432373

A 0 0

C 0.092919532 0.055751719

G 0.241995532 0.093075205

C 0.81678114 0.129942454

T 0 0

T 0 0

G 0.95976375 0.258397933

A 0 0

G 3.054691787 0.07316627

T 0 0

C 5.929721816 0

C 3.443877551 0.364431487

T 0 0

G 1.57943067 0.073461892

G 4.641812865 0.182748538

C 6.831050228 0.200913242

C 3.015260158 0.294171723

A 0 0

A 0 0

C 5.247543111 0.278138327

A 0 0

T 0 0

G 1.052236002 0.169109357

A 0 0

T 0 0

A 0 0

A 0 0

T 0 0

A 0 0

T 0 0

A 0 0

C 3.64395521 0.037957867

A 0 0

C 1.209909737 0.057614749

A 0 0

A 0 0

A 0 0

G 1.064241486 0.599845201

T 0 0

A 0 0

T 0 0

A 0 0

A 0 0

A 0 0

T 0 0

A 0 0

T 0 0

T 0 0

A 0 0

G 0.44141252 0.381219904

A 0 0

A 0 0

A 0 0

G 1.705115346 0.320962889

G 5.219835374 0.160610319

C 3.862401931 0.020116677

A 0 0

G 1.228847703 0.060435133

A 0 0

G 1.840987255 0.040461258

A 0 0

C 1.628332994 0.061062487

A 0 0

C 1.701517015 0.04100041

A 0 0

A 0 0

C 2.230863692 0.020466639

T 0 0

A 0 0

T 0 0

A 0 0

A 0 0

T 0 0

T 0 0

A 0 0

T 0 0

T 0 0

T 0 0

G 1.884794579 0.169419737

C 6.662423085 0.084871632

A 0 0

T 0 0

A 0 0

A 0 0

T 0 0

T 0 0

A 0 0

T 0 0

A 0 0

T 0 0

G 2.130274478 0.020483408

A 0 0

C 1.407588739 0.081599347

T 0 0

G 1.041883726 0.062513024

T 0 0

T 0 0

T 0 0

A 0 0

C 8.020261714 0.063317856

C 0.778619529 0.105218855

T 0 0

A 0 0

A 0 0

A 0 0

A 0 0

A 0 0

G 0.538444971 0.064613397

T 0 0

T 0 0

C 0.284215129 0.087450809

A 0 0

A 0 0

A 0 0

A 0 0

T 0 0

A 0 0

A 0 0

T 0 0

G 0.509864775 0.066504101

A 0 0

A 0 0

C 0.04428698 0.132860939

G 0.244390136 0.155520995

G 0.602947745 0.044662796

A 0 0

C 0.801246383 0.378366348

A 0 0

A 0 0

A 0 0

C 1.94361384 0.27765912

T 0 0

A 0 0

T 0 0

T 0 0

A 0 0

C 3.293918919 0.084459459

A 0 0

A 0 0

G 1.456003376 0.084405993

T 0 0

G 0.988609499 0.279389641

A 0 0

C 5.936562366 0.27861123

C 2.988260406 0.256136606

A 0 0

A 0 0

G 1.107165239 0.250678922

T 0 0

G 1.901140684 0.126742712

T 0 0

A 0 0

A 0 0

C 2.781875659 0.33719705

A 0 0

T 0 0

C 1.027837259 0.385438972

A 0 0

A 0 0

T 0 0

A 0 0

T 0 0

A 0 0

C 4.509340777 0.064419154

A 0 0

T 0 0

C 1.008583691 0.407725322

A 0 0

A 0 0

T 0 0

C 0.769724182 0.427624546

A 0 0

A 0 0

C 1.244368161 0.236000858

A 0 0

G 1.308170706 0.171563371

T 0 0

G 0.899550225 0.107089312

T 0 0

T 0 0

T 0 0

A 0 0

C 1.64074153 0.063924995

T 0 0

A 0 0

A 0 0

A 0 0

A 0 0

C 0.406330197 0.342172797

A 0 0

G 1.198886748 0.10704346

C 2.076643117 0.321130379

A 0 0

A 0 0

T 0 0

A 0 0

C 1.680493512 0.297808977

A 0 0

G 0.510529675 0.148904488

A 0 0

A 0 0

T 0 0

T 0 0

A 0 0

C 1.56019397 0.295171832

A 0 0

A 0 0

C 11.06902357 0.589225589

A 0 0

T 0 0

T 0 0

T 0 0

T 0 0

A 0 0

T 0 0

G 7.266789141 0.20412329

G 3.678861789 0.12195122

G 4.194528875 0.162107396

A 0 0

A 0 0

A 0 0

G 1.151219512 0.292682927

T 0 0

G 2.746615087 0.290135397

T 0 0

C 9.234930448 0.386398764

C 8.286652501 0.15452965

C 5.927934909 0.174351027

A 0 0

T 0 0

T 0 0

C 1.219268439 0.13991605

A 0 0

T 0 0

T 0 0

A 0 0

T 0 0

A 0 0

A 0 0

C 0.662916834 0.241060667

A 0 0

A 0 0

A 0 0

A 0 0

A 0 0

C 0.444264943 0.040387722

T 0 0

T 0 0

T 0 0

G 0.488698839 0.325799226

T 0 0

G 0.24434942 0.203624516

G 0.874872838 0.325534079

A 0 0

T 0 0

T 0 0

T 0 0

C 0.562248996 0.060240964

A 0 0

T 0 0

A 0 0

A 0 0

A 0 0

A 0 0

A 0 0

T 0 0

C 0.691562932 0.355660937

C 0.745829244 0.157016683

A 0 0

C 0.858034321 0.0975039

A 0 0

A 0 0

A 0 0

A 0 0

T 0 0

A 0 0

C 1.659270693 0.327995369

T 0 0

C 0.595924644 0.115340254

A 0 0

A 0 0

G 0.72769054 0.153198008

A 0 0

A 0 0

C 0.840817886 0.210204472

A 0 0

A 0 0

A 0 0

C 2.662889518 0.169971671

C 0.67860509 0.094250707

A 0 0

A 0 0

G 0.356405928 0.05627462

A 0 0

A 0 0

A 0 0

T 0 0

G 0.728835732 0.018688096

A 0 0

G 2.068579948 0.074543422

C 2.60707635 0.223463687

A 0 0

C 1.763504734 0.055689623

A 0 0

T 0 0

C 0.148395474 0.074197737

G 0.185219485 0.12965364

G 0.760667904 0.111317254

A 0 0

C 1.8144788 0.03703018

A 0 0

T 0 0

C 0.426083735 0.129677658

A 0 0

A 0 0

A 0 0

G 0.668399554 0.055699963

A 0 0

A 0 0

A 0 0

T 0 0

C 0.222634508 0

T 0 0

T 0 0

A 0 0

A 0 0

A 0 0

A 0 0

A 0 0

T 0 0

T 0 0

T 0 0

T 0 0

G 1.115989755 0.036589828

C 2.448830409 0.036549708

C 0.639035969 0.073032682

A 0 0

A 0 0

A 0 0

G 4.068289139 0.018162005

C 5.818379554 0.036251586

T 0 0

C 0.578452639 0.018076645

T 0 0

A 0 0

A 0 0

A 0 0

T 0 0

T 0 0

C 0.321428571 0.017857143

G 0.266951415 0.249154654

T 0 0

T 0 0

G 0.427046263 0.071174377

A 0 0

G 0.673758865 0.070921986

A 0 0

G 2.810180276 0.194414988

C 3.562610229 0.229276896

T 0 0

G 0.933262898 0.105652404

G 1.738672287 0.017562346

A 0 0

A 0 0

T 0 0

C 1.798307475 0.405500705

C 1.180824815 0.070497004

A 0 0

T 0 0

T 0 0

T 0 0

T 0 0

C 0.69735007 0.244072524

A 0 0

A 0 0

G 2.122477383 0.017397356

T 0 0

A 0 0

G 3.902607494 0.155413573

T 0 0

A 0 0

G 2.711103436 0.103609049

G 4.006216543 0.069072699

A 0 0

C 4.273801251 0.191104934

T 0 0

C 1.227639425 0.157839355

T 0 0

A 0 0

A 0 0

G 1.017857143 0.339285714

T 0 0

C 1.620453727 0.144040331

A 0 0

C 3.934010152 0.108774474

A 0 0

T 0 0

G 0.600436681 0.072780204

A 0 0

A 0 0

T 0 0

G 2.608213097 0.03699593

T 0 0

C 1.001855288 0.352504638

A 0 0

A 0 0

T 0 0

T 0 0

T 0 0

T 0 0

C 1.275415896 0.979667283

C 0.887738117 0.351396338

A 0 0

A 0 0

A 0 0

C 1.551819693 0.166266396

T 0 0

G 3.65497076 0.109649123

T 0 0

C 11.5517555 0.254684373

C 13.45838645 0.309597523

C 10.79162875 0.25477707

C 10.79162875 0.145586897

A 0 0

T 0 0

A 0 0

A 0 0

A 0 0

C 1.2984458 0.295101318

T 0 0

C 1.155148377 0.059749054

C 0.568066545 0.101440454

G 0.22380468 0.305188199

T 0 0

G 2.028272895 0.08195042

C 3.264216793 0.020529665

A 0 0

T 0 0

C 6.750360156 1.070179049

C 7.458393261 0.061639614

C 3.227808814 0.206910821

T 0 0

T 0 0

A 0 0

A 0 0

A 0 0

A 0 0

C 0.820189274 0.168243954

T 0 0

C 5.243288591 0.230704698

C 2.483823836 0.083489877

C 0.605301607 0.5426842

G 0 0

C 1.046243984 0.272023436

A 0 0

G 2.012578616 0.398322851

G 3.641338666 0.126289202

A 0 0

C 3.685659818 0.021181953

T 0 0

G 1.145767027 0.169743263

C 1.756242065 0.380871773

T 0 0

G 1.16451408 0.021172983

T 0 0

G 1.567532746 0.279149667

G 2.615219721 0.685959271

G 8.065208065 0.664950665

T 0 0

C 3.428819444 0.151909722

T 0 0

T 0 0

G 0.638625853 0.682669016

A 0 0

C 2.298342541 0.35359116

A 0 0

C 2.181736392 0.179937022

T 0 0

G 2.210070631 0.729095466

C 2.963300661 0.159562343

T 0 0

T PRIMER NA NA

C PRIMER NA NA

T PRIMER NA NA

A PRIMER NA NA

A PRIMER NA NA

A PRIMER NA NA

C PRIMER NA NA

T PRIMER NA NA

T PRIMER NA NA

G PRIMER NA NA

A PRIMER NA NA

C PRIMER NA NA

A PRIMER NA NA

T PRIMER NA NA

G PRIMER NA NA

G PRIMER NA NA

G PRIMER NA NA

A PRIMER NA NA

G PRIMER NA NA

A PRIMER NA NA

G PRIMER NA NA

G PRIMER NA NA

A PRIMER NA NA

A PRIMER NA NA

A PRIMER NA NA

C 0.432380495 0.360317079

G 0.360490267 0.120163422

T 0 0

G 5.558229066 0.264677575

T 0 0

A 0 0

G 5.412621359 0.194174757

G 7.518248175 0.121654501

A 0 0

C 12.53701876 0.098716683

C 2.807017544 0.125313283

C 0.234070221 0.182054616

G 0.078308536 0.443748369

C 0.996068152 0.157273919

T 0 0

G 0.480128034 0.320085356

A 0 0

G 1.409596097 0.352399024

G 3.955264594 0.027277687

A 0 0

A 0 0

T 0 0

G 4.516486561 0.08312552

C 4.219877846 0

T 0 0

T 0 0

C 0.638711469 0.111080255

T 0 0

G 0.285143998 0.171086399

A 0 0

A 0 0

C 0.257142857 0.314285714

G 0.317827218 0.144466917

G 7.681201271 0.404273751

C 10.6327651 0.346720601

C 4.137731481 0.115740741

T 0 0

C 0.173010381 0.144175317

G 0.057636888 0.028818444

G 2.308802309 0.028860029

G 8.121387283 0

G 7.823819183 0.492610837

A 0 0

G 5.30056263 0.207284572

G 8.363095238 0.089285714

G 6.535362578 0.387943897

A 0 0

G 7.508532423 0.09308098

C 11.80555556 0.378787879

C 3.678822777 0.287907869

T 0 0

G 1.498501499 0.3996004

C 2.199266911 0.16661113

T 0 0

G 1.328337875 0.442779292

T 0 0

A 0 0

A 0 0

A 0 0

G 3.455425017 0.276434001

C 4.070981211 0.243562978

T 0 0

G 0.632911392 0.335070737

T 0 0

C 1.520334474 0.266058533

A 0 0

C 1.864535769 0.342465753

A 0 0

G 1.310717039 0.501156515

T 0 0

T 0 0

A 0 0

A 0 0

T 0 0

G 5.067837191 0.11971269

C 7.016840417 0.040096231

C 2.900886382 0.040290089

A 0 0

G 1.676445935 0.209555742

T 0 0

G 2.242911553 0.338552687

C 2.948717949 0.384615385

T 0 0

A 0 0

C 3.586862576 0.043215212

T 0 0

G 1.369863014 0.091324201

T 0 0

G 1.409774436 0.469924812

C 1.36663525 0.047125353

A 0 0

G 0.380589914 1.141769743

A 0 0

A 0 0

A 0 0

G 0.535019455 1.070038911

A 0 0

G 0.198807157 1.491053678

A 0 0

A 0 0

C 2.066532258 0.100806452

T 0 0

C 1.206082853 0.10487677

A 0 0

G 5.131065254 0.111544897

C 7.467714767 0.056148231

C 1.868629672 0.226500566

C 0.458715596 0.401376147

G 0 0

T 0 0

G 0.233100233 0.291375291

A 0 0

G 1.410934744 0.529100529

T 0 0

C 1.548540798 0

A 0 0

G 1.077844311 0.718562874

G 3.121248499 0.06002401

A 0 0

C 3.04136253 0.243309002

T 0 0

G 0.453661698 0.583279326

A 0 0

G 0 0

A 0 0

C 1.864640884 0.276243094

A 0 0

G 0.436363636 1.090909091

C 1.823486506 0.07293946

A 0 0

G 0.516224189 0.368731563

A 0 0

G 0.531914894 0.455927052

A 0 0

C 1.466049383 0.154320988

A 0 0

G 1.209677419 0.483870968

C 3.027823241 2.700490998

A 0 0

A 0 0

G 1.996672213 0.332778702

T 0 0

A 0 0

T 0 0

T 0 0

T 0 0

A 0 0

C 0 0

G 0 0

G 0 0

G 1.563937443 0.09199632

A 0 0

A 0 0

T 0 0

T 0 0

T 0 0

T 0 0

A 0 0

T 0 0

T 0 0

T 0 0

A 0 0

C 0.192122959 0

G 0 0

A 0 0

T 0 0

G 0 0

A 0 0

T 0 0

G 1.771653543 0.196850394

G 5.830039526 0

T 0 0

A 0 0

G 2.911646586 0.100401606

C 9.746192893 0.406091371

A 0 0

T 0 0

T 0 0

T 0 0

C 1.364764268 0

A 0 0

T 0 0

G 2.525252525 1.136363636

T 0 0

C 1.955671447 0

A 0 0

G 0.820793434 1.504787962

T 0 0

C 0.280504909 0

G 0 0

G 2.73381295 1.294964029

T 0 0

G 2.052785924 0.73313783

G 2.359882006 0.589970501

A 0 0

G 1.664145234 0.15128593

A 0 0

A 0 0

A 0 0

A 0 0

G NA NA

A NA NA

T NA NA

T NA NA

C NA NA

C NA NA

T NA NA

T NA NA

T NA NA

T NA NA

T NA NA

C NA NA

A NA NA

A NA NA

T NA NA

A NA NA

A NA NA

A NA NA

T NA NA

G NA NA

A NA NA

T NA NA

G NA NA

T NA NA

G NA NA

A NA NA

C NA NA

A NA NA

A NA NA

C NA NA

T NA NA

G NA NA

G NA NA

C NA NA

T NA NA

A NA NA

C NA NA

G NA NA

T NA NA

A NA NA

T NA NA

T NA NA

T NA NA

G NA NA

A NA NA

A NA NA

A NA NA

A NA NA

A NA NA

G NA NA

A NA NA

A NA NA

A NA NA

A NA NA

C NA NA

G NA NA

T NA NA

T NA NA

A NA NA

G NA NA

A NA NA

C NA NA

C NA NA

C NA NA

G NA NA

T NA NA

A NA NA

T NA NA

C NA NA

T NA NA

C NA NA

A NA NA

C NA NA

A NA NA

C NA NA

A NA NA

A NA NA

C NA NA

C NA NA

C NA NA

C NA NA

A NA NA

A NA NA

A NA NA

C NA NA

A NA NA

A NA NA

A NA NA

A NA NA

T NA NA

T NA NA

C NA NA

C NA NA

C NA NA

G NA NA

C NA NA

G NA NA

G NA NA

A NA NA

T NA NA

T NA NA

A NA NA

A NA NA

A NA NA

G NA NA

G NA NA

G NA NA

C NA NA

T NA NA

A NA NA

A NA NA

C NA NA

C NA NA

A NA NA

C NA NA

C NA NA

T NA NA

G NA NA

T NA NA

G NA NA

A NA NA

T NA NA

G NA NA

G NA NA

A NA NA

G NA NA

T NA NA

G NA NA

A NA NA

A NA NA

T NA NA

G NA NA

C NA NA

T NA NA

G NA NA

A NA NA

T NA NA

G NA NA

T NA NA

G NA NA

T NA NA

C NA NA

C NA NA

C NA NA

C NA NA

C NA NA

C NA NA

A NA NA

A NA NA

A NA NA

A NA NA

T NA NA

T NA NA

C NA NA

C NA NA

T NA NA

G NA NA

T NA NA

G NA NA

T NA NA

T NA NA

G NA NA

A NA NA

A NA NA

G NA NA

C NA NA

C NA NA

T NA NA

C NA NA

A NA NA

C NA NA

C NA NA

C NA NA

C NA NA

C NA NA

A NA NA

G NA NA

T NA NA

G NA NA

A NA NA

C NA NA

A NA NA

C NA NA

G NA NA

G NA NA

T NA NA

A NA NA

T NA NA

C NA NA

A NA NA

G NA NA

G NA NA

A NA NA

G NA NA

A NA NA

G NA NA

G NA NA

G NA NA

A NA NA

G NA NA

C NA NA

C NA NA

T NA NA

T NA NA

C NA NA

G NA NA

T NA NA

T NA NA

A NA NA

C NA NA

G NA NA

T NA NA

T NA NA

A NA NA

G NA NA

C NA NA

C NA NA

A NA NA

G NA NA

G NA NA

A NA NA

A NA NA

T NA NA

C NA NA

C NA NA

C NA NA

A NA NA

C NA NA

C NA NA

C NA NA

T NA NA

C NA NA

A NA NA

C NA NA

C NA NA

A NA NA

G NA NA

G NA NA

A NA NA

A NA NA

C NA NA

C NA NA

T NA NA

G NA NA

A NA NA

C NA NA

C NA NA

A NA NA

T NA NA

G NA NA

C NA NA

T NA NA

A NA NA

C NA NA

A NA NA

C NA NA

G NA NA

G NA NA

T NA NA

G NA NA

A NA NA

T NA NA

C NA NA

C NA NA

A NA NA

A NA NA

G NA NA

A NA NA

C NA NA

T NA NA

C NA NA

C NA NA

C NA NA

A NA NA

A NA NA

C NA NA

C NA NA

T NA NA

C NA NA

C NA NA

A NA NA

G NA NA

A NA NA

A NA NA

C NA NA

C NA NA

G NA NA

T NA NA

G NA NA

G NA NA

G NA NA

A NA NA

A NA NA

G NA NA

T NA NA

A NA NA

A NA NA

G NA NA

T NA NA

T NA NA

T NA NA

C NA NA

T NA NA

G NA NA

C NA NA

T NA NA

G NA NA

T NA NA

T NA NA

T PRIMER NA NA

A PRIMER NA NA

A PRIMER NA NA

G PRIMER NA NA

C PRIMER NA NA

C PRIMER NA NA

C PRIMER NA NA

C PRIMER NA NA

A PRIMER NA NA

C PRIMER NA NA

A PRIMER NA NA

G PRIMER NA NA

T PRIMER NA NA

C PRIMER NA NA

T PRIMER NA NA

A PRIMER NA NA

T PRIMER NA NA

G PRIMER NA NA

T PRIMER NA NA

T PRIMER NA NA

A PRIMER NA NA

T PRIMER NA NA

T PRIMER NA NA

T PRIMER NA NA

T PRIMER NA NA

G NA NA

T NA NA

T NA NA

C NA NA

T NA NA

C NA NA

A NA NA

G NA NA

C NA NA

C NA NA

C NA NA

C NA NA

G NA NA

G NA NA

C NA NA

T NA NA

G NA NA

A NA NA

C NA NA

G NA NA

A NA NA

G NA NA

A NA NA

A NA NA

C NA NA

G NA NA

C NA NA

C NA NA

A NA NA

T NA NA

T NA NA

A NA NA

G NA NA

A NA NA

A NA NA

C NA NA

A NA NA

T NA NA

C NA NA

A NA NA

G NA NA

A NA NA

A NA NA

G NA NA

C NA NA

C NA NA

A NA NA

C NA NA

A NA NA

G NA NA

C NA NA

C NA NA

A NA NA

T NA NA

A NA NA

G NA NA

A NA NA

A NA NA

G NA NA

G NA NA

A NA NA

C NA NA

A NA NA

G NA NA

A NA NA

C NA NA

G NA NA

A NA NA

G NA NA

A NA NA

C NA NA

G NA NA

C NA NA

T NA NA

G NA NA

A NA NA

A NA NA

G NA NA

T NA NA

T NA NA

T NA NA

G NA NA

T NA NA

T NA NA

T NA NA

T NA NA

A NA NA

A NA NA

C NA NA

C NA NA

G NA NA

T NA NA

C NA NA

T NA NA

T NA NA

G NA NA

C NA NA

C NA NA

A NA NA

T NA NA

A NA NA

A NA NA

G NA NA

A NA NA

A NA NA

A NA NA

T NA NA

A NA NA

C NA NA

C NA NA

C NA NA

T NA NA

A NA NA

T NA NA

A NA NA

A NA NA

G NA NA

T NA NA

C NA NA

A NA NA

C NA NA

A NA NA

T NA NA

C NA NA

T NA NA

G NA NA

A NA NA

G NA NA

A NA NA

C NA NA

T NA NA

C NA NA

A NA NA

G NA NA

G NA NA

C NA NA

G NA NA

T NA NA

C NA NA

C NA NA

T NA NA

A NA NA

A NA NA

G NA NA

A NA NA

A NA NA

A NA NA

G NA NA

G NA NA

G NA NA

G NA NA

G NA NA

G NA NA

T NA NA

G NA NA

T NA NA

G NA NA

G NA NA

A NA NA

C NA NA

A NA NA

C NA NA

T NA NA

G NA NA

C NA NA

C NA NA

C NA NA

C NA NA

C NA NA

A NA NA

G NA NA

C NA NA

A NA NA

C NA NA

C NA NA

T NA NA

C NA NA

T NA NA

T NA NA

G NA NA

T NA NA

G NA NA

G NA NA

C NA NA

C NA NA

G NA NA

G NA NA

G NA NA

C NA NA

T NA NA

G NA NA

G NA NA

G NA NA

T NA NA

G NA NA

A NA NA

C NA NA

A NA NA

G NA NA

G NA NA

C NA NA

A NA NA

C NA NA

A NA NA

C NA NA

A NA NA

G NA NA

G NA NA

A NA NA

A NA NA

C NA NA

T NA NA

C NA NA

A NA NA

T NA NA

G NA NA

G NA NA

C NA NA

A NA NA

C NA NA

T NA NA

C NA NA

G NA NA

T NA NA

C NA NA

T NA NA

C NA NA

A NA NA

T NA NA

A NA NA

A NA NA

C NA NA

C NA NA

A NA NA

T NA NA

T NA NA

G NA NA

T NA NA

T NA NA

C NA NA

A NA NA

C NA NA

A NA NA

T NA NA

T NA NA

A NA NA

A NA NA

A NA NA

C NA NA

G NA NA

T NA NA

G NA NA

C NA NA

A NA NA

C NA NA

C NA NA

A NA NA

A NA NA

A NA NA

A NA NA

T NA NA

T NA NA

T NA NA

A NA NA

C NA NA

A NA NA

A NA NA

C NA NA

A NA NA

G NA NA

A NA NA

A NA NA

C NA NA

A NA NA

C NA NA

C NA NA

A NA NA

T NA NA

C NA NA

G NA NA

A NA NA

C NA NA

A NA NA

A NA NA

A NA NA

G NA NA

C NA NA

T NA NA

A NA NA

A NA NA

A NA NA

A NA NA

G NA NA

G NA NA

C NA NA

A NA NA

G NA NA

G NA NA

C NA NA

A NA NA

A NA NA

T NA NA

G NA NA

G NA NA

A NA NA

T NA NA

G NA NA

G NA NA

A NA NA

A NA NA

G NA NA

A NA NA

A NA NA

A NA NA

C NA NA

C NA NA

A NA NA

T NA NA

T NA NA

T NA NA

C NA NA

C NA NA

A NA NA

A NA NA

A NA NA

C NA NA

T NA NA

A NA NA

C NA NA

C NA NA

A NA NA

A NA NA

C NA NA

A NA NA

T NA NA

G NA NA

G NA NA

T NA NA

G NA NA

T NA NA

C NA NA

C NA NA

C NA NA

A NA NA

A NA NA

C NA NA

A NA NA

G NA NA

G NA NA

A NA NA

A NA NA

A NA NA

A NA NA

G NA NA

A NA NA

C NA NA

T NA NA

A NA NA

A NA NA

C NA NA

T NA NA

A NA NA

C NA NA

T NA NA

G NA NA

G NA NA

G NA NA

C NA NA

A NA NA

A NA NA

A NA NA

A NA NA

G NA NA

A NA NA

T NA NA

G NA NA

C NA NA

G NA NA

G NA NA

T NA NA

T NA NA

A NA NA

A NA NA

A NA NA

C NA NA

A NA NA

A NA NA

G NA NA

T NA NA

C NA NA

A NA NA

C NA NA

A NA NA

G NA NA

A NA NA

A NA NA

G NA NA

A NA NA

A NA NA

C NA NA

C NA NA

C NA NA

A NA NA

C NA NA

A NA NA

G NA NA

G NA NA

T NA NA

A NA NA

C NA NA

C NA NA

G NA NA

C NA NA

T NA NA

C NA NA

C NA NA

T NA NA

G NA NA

C NA NA

A NA NA

G NA NA

A NA NA

A NA NA

G NA NA

C NA NA

T NA NA

G NA NA

G NA NA

C NA NA

G NA NA

A NA NA

C NA NA

T NA NA

C NA NA

T NA NA

G NA NA

C NA NA

C NA NA

G NA NA

A NA NA

C NA NA

A NA NA

C NA NA

C NA NA

G NA NA

T NA NA

C NA NA

A NA NA

G NA NA

C NA NA

A NA NA

T NA NA

G NA NA

A NA NA

C NA NA

G NA NA

C NA NA

A NA NA

G NA NA

A NA NA

G NA NA

G NA NA

G NA NA

G NA NA

T NA NA

C NA NA

T NA NA

C NA NA

C NA NA

T NA NA

C NA NA

C NA NA

C NA NA

C NA NA

A NA NA

C NA NA

A NA NA

C NA NA

C NA NA

C NA NA

A NA NA

C NA NA

A NA NA

G NA NA

C NA NA

T NA NA

C NA NA

C NA NA

G NA NA

C NA NA

G NA NA

C NA NA

A NA NA

T NA NA

C NA NA

C NA NA

T NA NA

C NA NA

C NA NA

C NA NA

C NA NA

T NA NA

G NA NA

T NA NA

G NA NA

G NA NA

G NA NA

C NA NA

C NA NA

C NA NA

G NA NA

A NA NA

G NA NA

G NA NA

T NA NA

G NA NA

G NA NA

G NA NA

C NA NA

G NA NA

T NA NA

G NA NA

T NA NA

A NA NA

C NA NA

T NA NA

G NA NA

A NA NA

T NA NA

T NA NA

C NA NA

A NA NA

T NA NA

C NA NA

C NA NA

C NA NA

C NA NA

A NA NA

C NA NA

C NA NA

T NA NA

C NA NA

T NA NA

G NA NA

T NA NA

C NA NA

T NA NA

G NA NA

C NA NA

T NA NA

C NA NA

C NA NA

T NA NA

G NA NA

A NA NA

G NA NA

A NA NA

G NA NA

C NA NA

A NA NA

G NA NA

C NA NA

G NA NA

C NA NA

C NA NA

C NA NA

T NA NA

T NA NA

C NA NA

A NA NA

C NA NA

A NA NA

T NA NA

C NA NA

T NA NA

G NA NA

C NA NA

G NA NA

T NA NA

C NA NA

C NA NA

T NA NA

A NA NA

G NA NA

G NA NA

C NA NA

C NA NA

C NA NA

C NA NA

A NA NA

C NA NA

T NA NA

A NA NA

A NA NA

A NA NA

C NA NA

G NA NA

C NA NA

A NA NA

G NA NA

G NA NA

A NA NA

C NA NA

A NA NA

C NA NA

G NA NA

G NA NA

A NA NA

G NA NA

G NA NA

C NA NA

C NA NA

C NA NA

T NA NA

C NA NA

G NA NA

C NA NA

A NA NA

A NA NA

A NA NA

C NA NA

T NA NA

T NA NA

C NA NA

T NA NA

C NA NA

G NA NA

A NA NA

A NA NA

T NA NA

G NA NA

A NA NA

A NA NA

T NA NA

G NA NA

A NA NA

A NA NA

C NA NA

G NA NA

A NA NA

A NA NA

T NA NA

G NA NA

A NA NA

A NA NA

G NA NA

G NA NA

T NA NA

C NA NA

G NA NA

T NA NA

T NA NA

T NA NA

T NA NA

C NA NA

A NA NA

A NA NA

G NA NA

A NA NA

C NA NA

C NA NA

T NA NA

C NA NA

T NA NA

G NA NA

G NA NA

C NA NA

C NA NA

C NA NA

T NA NA

T NA NA

C NA NA

C NA NA

C NA NA

C NA NA

T NA NA

C NA NA

T NA NA

T NA NA

A NA NA

G NA NA

A NA NA

A NA NA

T NA NA

C NA NA

T NA NA

C NA NA

T NA NA

A NA NA

A NA NA

A NA NA

T NA NA

C NA NA

T NA NA

C NA NA

A NA NA

C NA NA

A NA NA

C NA NA

T NA NA

T NA NA

G NA NA

C NA NA

A NA NA

C NA NA

T NA NA

T NA NA

G NA NA

C NA NA

T NA NA

C NA NA

T NA NA

G NA NA

G NA NA

A NA NA

G NA NA

G NA NA

G NA NA

G NA NA

A NA NA

T NA NA

G NA NA

A NA NA

A NA NA

A NA NA

C NA NA

A NA NA

G NA NA

A NA NA

C NA NA

A NA NA

C NA NA

T NA NA

G NA NA

C NA NA

C NA NA

A NA NA

G NA NA

G NA NA

G NA NA

C NA NA

C NA NA

C NA NA

G NA NA

G NA NA

A NA NA

G NA NA

G NA NA

T NA NA

T NA NA

T NA NA

A NA NA

C NA NA

G NA NA

A NA NA

T NA NA

G NA NA

T NA NA

T NA NA

T NA NA

A NA NA

T NA NA

G NA NA

C NA NA

T NA NA

G NA NA

A NA NA

G NA NA

G NA NA

C NA NA

C NA NA

C NA NA

A NA NA

G NA NA

G NA NA

G NA NA

A NA NA

G NA NA

C NA NA

T NA NA

A NA NA

T NA NA

G NA NA

C NA NA

G NA NA

G NA NA

G NA NA

C NA NA

T NA NA

C NA NA

A NA NA

G NA NA

G NA NA

A NA NA

T NA NA

C NA NA

C NA NA

A NA NA

T NA NA

G NA NA

T NA NA

G NA NA

G NA NA

A NA NA

T NA NA

T NA NA

T NA NA

G NA NA

T NA NA

T NA NA

A NA NA

C NA NA

C NA NA

C NA NA

T NA NA

G NA NA

A NA NA

A NA NA

C NA NA

T NA NA

C NA NA

A NA NA

G NA NA

C NA NA

G NA NA

G NA NA

T NA NA

C NA NA

A NA NA

G NA NA

G NA NA

G NA NA

C NA NA

C NA NA

T NA NA

G NA NA

C NA NA

T NA NA

G NA NA

G NA NA

T NA NA

G NA NA

A NA NA

A NA NA

T NA NA

C NA NA

A NA NA

G NA NA

C NA NA

A NA NA

A NA NA

A NA NA

A NA NA

C NA NA

C NA NA

T NA NA

C NA NA

C NA NA

G NA NA

G NA NA

C NA NA

T NA NA

G NA NA

T NA NA

G NA NA

C NA NA

T NA NA

T NA NA

C NA NA

A NA NA

T NA NA

T NA NA

C NA NA

C NA NA

A NA NA

G NA NA

G NA NA

G NA NA

A NA NA

G NA NA

G NA NA

A NA NA

A NA NA

A NA NA

C NA NA

C NA NA

C NA NA

A NA NA

G NA NA

C NA NA

A NA NA

G NA NA

C NA NA

C NA NA

C NA NA

C NA NA

G NA NA

T NA NA

C NA NA

C NA NA

C NA NA

C NA NA

G NA NA

T NA NA

C NA NA

T NA NA

G NA NA

C NA NA

A NA NA

T NA NA

T NA NA

T NA NA

C NA NA

C NA NA

G NA NA

C NA NA

T NA NA

G NA NA

A NA NA

C NA NA

G NA NA

C NA NA

T NA NA

A NA NA

T NA NA

C NA NA

A NA NA

C NA NA

T NA NA

G NA NA

G NA NA

A NA NA

G NA NA

A NA NA

G NA NA

G NA NA

G NA NA

A NA NA

G NA NA

A NA NA

C NA NA

G NA NA

A NA NA

G NA NA

G NA NA

A NA NA

G NA NA

T NA NA

C NA NA

C NA NA

T NA NA

T NA NA

G NA NA

A NA NA

A NA NA

A NA NA

C NA NA

C NA NA

A NA NA

G NA NA

C NA NA

C NA NA

C NA NA

T NA NA

C NA NA

C NA NA

G NA NA

A NA NA

A NA NA

C NA NA

A NA NA

T NA NA

A NA NA

A NA NA

A NA NA

T NA NA

A NA NA

A NA NA

G NA NA

C NA NA

A NA NA

G NA NA

C NA NA

C NA NA

G NA NA

G NA NA

G NA NA

T NA NA

T NA NA

C NA NA

A NA NA

G NA NA

T NA NA

G NA NA

A NA NA

A NA NA

G NA NA

C NA NA

A NA NA

C NA NA

A NA NA

T NA NA

G NA NA

A NA NA

C NA NA

G NA NA

C NA NA

A NA NA

G NA NA

G NA NA

C NA NA

T NA NA

T NA NA

C NA NA

A NA NA

C NA NA

C NA NA

T NA NA

C NA NA

T NA NA

G NA NA

C NA NA

C NA NA

A NA NA

G NA NA

C NA NA

G NA NA

T NA NA

T NA NA

T NA NA

C NA NA

C NA NA

A NA NA

T NA NA

C NA NA

C NA NA

C NA NA

A NA NA

G NA NA

A NA NA

A NA NA

A NA NA

G NA NA

T NA NA

C NA NA

C NA NA

T NA NA

G NA NA

C NA NA

G NA NA

G NA NA

G NA NA

A NA NA

C NA NA

A NA NA

G NA NA

A NA NA

T NA NA

T NA NA

C NA NA

T NA NA

C NA NA

A NA NA

C NA NA

A NA NA

G NA NA

T NA NA

C NA NA

C NA NA

C NA NA

C NA NA

A NA NA

G NA NA

T NA NA

G NA NA

A NA NA

G NA NA

C NA NA

C NA NA

C NA NA

C NA NA

A NA NA

T NA NA

C NA NA

C NA NA

A NA NA

T NA NA

G NA NA

A NA NA

C NA NA

C NA NA

C NA NA

C NA NA

T NA NA

G NA NA

C NA NA

T NA NA

C NA NA

G NA NA

C NA NA

C NA NA

C NA NA

C NA NA

C NA NA

C NA NA

A NA NA

C NA NA

G NA NA

T NA NA

T NA NA

T NA NA

C NA NA

C NA NA

C NA NA

A NA NA

G NA NA

A NA NA

T NA NA

G NA NA

A NA NA

C NA NA

G NA NA

G NA NA

C NA NA

A NA NA

G NA NA

C NA NA

T NA NA

G NA NA

G NA NA

A NA NA

G NA NA

A NA NA

G NA NA

C NA NA

C NA NA

T NA NA

T NA NA

C NA NA

C NA NA

T NA NA

C NA NA

C NA NA

G NA NA

A NA NA

C NA NA

A NA NA

G NA NA

C NA NA

G NA NA

G NA NA

C NA NA

C NA NA

C NA NA

C NA NA

A NA NA

G NA NA

G NA NA

C NA NA

C NA NA

A NA NA

C NA NA

G NA NA

G NA NA

A NA NA

G NA NA

A NA NA

G NA NA

G NA NA

C NA NA

G NA NA

G NA NA

C NA NA

C NA NA

C NA NA

C NA NA

G NA NA

C NA NA

A NA NA

G NA NA

A NA NA

G NA NA

A NA NA

G NA NA

C NA NA

G NA NA

G NA NA

A NA NA

T NA NA

G NA NA

T NA NA

T NA NA

T NA NA

G NA NA

T NA NA

G NA NA

A NA NA

A NA NA

G NA NA

C NA NA

T NA NA

C NA NA

C NA NA

T NA NA

C NA NA

T NA NA

T NA NA

G NA NA

G NA NA

A NA NA

G NA NA

G NA NA

G NA NA

A NA NA

A NA NA

A NA NA

G NA NA

C NA NA

T NA NA

G NA NA

C NA NA

C NA NA

C NA NA

C NA NA

C NA NA

A NA NA

A NA NA

C NA NA

G NA NA

C NA NA

T NA NA

G NA NA

A NA NA

A NA NA

C NA NA

A NA NA

C NA NA

T NA NA

C NA NA

C NA NA

C NA NA

G NA NA

T NA NA

C NA NA

T NA NA

C NA NA

A NA NA

A NA NA

G NA NA

G NA NA

C NA NA

A NA NA

C NA NA

A NA NA

G NA NA

A NA NA

C NA NA

A NA NA

G NA NA

G NA NA

G NA NA

G NA NA

A NA NA

G NA NA

C NA NA

G NA NA

G NA NA

T NA NA

C NA NA

A NA NA

C NA NA

A NA NA

A NA NA

G NA NA

C NA NA

C NA NA

G NA NA

G NA NA

C NA NA

C NA NA

A NA NA

G NA NA

T NA NA

C NA NA

C NA NA

C NA NA

T NA NA

T NA NA

T NA NA

T NA NA

A NA NA

C NA NA

A NA NA

G NA NA

C NA NA

C NA NA

A NA NA

A NA NA

A NA NA

A NA NA

T NA NA

C NA NA

G NA NA

G NA NA

G NA NA

G NA NA

G NA NA

C NA NA

T NA NA

C NA NA

A NA NA

G NA NA

A NA NA

A NA NA

C NA NA

A NA NA

T NA NA

T NA NA

C NA NA

T NA NA

T NA NA

T NA NA

A NA NA

A NA NA

T NA NA

G NA NA

A NA NA

T NA NA

T NA NA

T NA NA

C NA NA

C NA NA

G NA NA

A NA NA

G NA NA

C NA NA

C NA NA

A NA NA

C NA NA

C NA NA

G NA NA

A NA NA

G NA NA

T NA NA

G NA NA

C NA NA

A NA NA

C NA NA

T NA NA

G NA NA

G NA NA

G NA NA

C NA NA

G NA NA

G NA NA

C NA NA

C NA NA

C NA NA

C NA NA

G NA NA

T NA NA

C NA NA

T NA NA

C NA NA

T NA NA

G NA NA

T NA NA

T NA NA

T NA NA

T NA NA

END CONTIG NA NA

T NA NA

T NA NA

G NA NA

T NA NA

T NA NA

G NA NA

T NA NA

G NA NA

A NA NA

C NA NA

C NA NA

T NA NA

T NA NA

T NA NA

A NA NA

C NA NA

C NA NA

G NA NA

A NA NA

A NA NA

A NA NA

T NA NA

A NA NA

T NA NA

C NA NA

A NA NA

A NA NA

T NA NA

A NA NA

A NA NA

G NA NA

C PRIMER NA NA

A PRIMER NA NA

G PRIMER NA NA

A PRIMER NA NA

A PRIMER NA NA

A PRIMER NA NA

T PRIMER NA NA

A PRIMER NA NA

T PRIMER NA NA

G PRIMER NA NA

G PRIMER NA NA

A PRIMER NA NA

A PRIMER NA NA

G PRIMER NA NA

C PRIMER NA NA

G PRIMER NA NA

T PRIMER NA NA

T PRIMER NA NA

T PRIMER NA NA

T PRIMER NA NA

G PRIMER NA NA

G PRIMER NA NA

T PRIMER NA NA

A PRIMER NA NA

A PRIMER NA NA

A PRIMER NA NA

G 0.93047299 1.163091238

A 0 0

T 0 0

A 0 0

G 4.354904355 1.241351241

T 0 0

A 0 0

G 5.960144928 0.887681159

C 4.23166577 1.380670611

T 0 0

A 0 0

G 4.097796143 0.654269972

G 3.891708968 0.947546531

A 0 0

T 0 0

G 2.371364653 0.164056674

T 0 0

C 2.794096575 1.017337727

C 3.159535279 0.949277416

A 0 0

A 0 0

G 1.196272082 0.264292669

A 0 0

A 0 0

G 1.708727655 0.026288118

T 0 0

C 1.031193607 1.753029131

T 0 0

A 0 0

T 0 0

A 0 0

A 0 0

C 4.786901637 1.587301587

A 0 0

T 0 0

T 0 0

T 0 0

T 0 0

T 0 0

A 0 0

G 0.961898813 0.074953154

G 3.8221334 0.087434424

A 0 0

A 0 0

G 1.736631684 0.387306347

G 6.835791052 0.062484379

T 0 0

G 0.9875 0.0125

T 0 0

T 0 0

T 0 0

G 0.950118765 0.012501563

T 0 0

G 1.363352095 0.062539087

G 2.851069151 0.050018757

G 11.80590295 0.012506253

T 0 0

A 0 0

G 2.801050394 0.012504689

C 3.626359885 0.037514068

A 0 0

T 0 0

G 0.775 0

A 0 0

T 0 0

G 0.687671918 0.012503126

A 0 0

C 2.963611354 0.012504689

C 0.725816544 0.100112627

A 0 0

A 0 0

A 0 0

G 1.53865399 0.012509382

G 4.842948317 0

C 2.714535902 0

A 0 0

G 0.375516335 0

A 0 0

T 0 0

T 0 0

T 0 0

C 0.337922403 0

A 0 0

A 0 0

T 0 0

G 2.976488244 0

T 0 0

G 2.650662666 0.025006252

T 0 0

A 0 0

T 0 0

A 0 0

G 1.526526527 0.012512513

C 1.851388541 0.025018764

A 0 0

G 1.551745714 0

T 0 0

G 1.464147166 0.037542235

G 2.96620776 0.025031289

G 2.939704779 0

A 0 0

A 0 0

A 0 0

T 0 0

G 1.888444222 0

T 0 0

G 0.563486101 0.025043827

A 0 0

G 0.763358779 0

A 0 0

G 1.163372529 0.012509382

A 0 0

G 2.265898848 0.012518778

G 4.792292292 0.05005005

A 0 0

C 4.618272841 0.050062578

C 1.289434151 0.025037556

A 0 0

A 0 0

A 0 0

A 0 0

G 0.625625626 0.012512513

A 0 0

T 0 0

G 1.926444834 0.025018764

T 0 0

G 2.816020025 0.012515645

T 0 0

A 0 0

G 0.914557755 0.037584565

A 0 0

G 0.852023556 0.062648791

A 0 0

T 0 0

A 0 0

C 3.828828829 0.037537538

A 0 0

T 0 0

G 1.953663118 0.062617408

G 2.994612204 0.13782734

C 2.944862155 0.062656642

A 0 0

A 0 0

G 1.127254509 0.175350701

G 3.180964308 0.175328741

A 0 0

A 0 0

T 0 0

A 0 0

C 1.050919555 0.012510947

A 0 0

G 0.401153316 0.325937069

A 0 0

T 0 0

C 0.325773713 0.037589275

T 0 0

G 0.864336716 0.037579857

G 2.805611222 0.062625251

C 2.126328956 0.025015635

A 0 0

A 0 0

G 0.751503006 0.0501002

A 0 0

T 0 0

G 0.287967948 0

A 0 0

A 0 0

A 0 0

T 0 0

T 0 0

T 0 0

C 0.46470736 0.050238634

A 0 0

T 0 0

T 0 0

T 0 0

T 0 0

A 0 0

T 0 0

T 0 0

T 0 0

T 0 0

A 0 0

T 0 0

T 0 0

T 0 0

T 0 0

A 0 0

T 0 0

T 0 0

A 0 0

T 0 0

T 0 0

T 0 0

T 0 0

T 0 0

A 0 0

T 0 0

T 0 0

C 0.513977686 0.012536041

A 0 0

T 0 0

T 0 0

T 0 0

C 0.463136813 0.087620478

T 0 0

C 0.839493798 0.050119033

T 0 0

T 0 0

A 0 0

C 2.402703041 0.062570392

A 0 0

T 0 0

T 0 0

T 0 0

T 0 0

T 0 0

A 0 0

T 0 0

A 0 0

A 0 0

C 1.067570962 0.037678975

T 0 0

T 0 0

C 0.539252571 0.112866817

A 0 0

A 0 0

C 0.388082123 0.225338007

T 0 0

T 0 0

T 0 0

T 0 0

A 0 0

T 0 0

T 0 0

T 0 0

T 0 0

A 0 0

C 0.819775508 0.517089166

A 0 0

G 0.674099041 0.362976407

A 0 0

T 0 0

G 3.974868573 0.128221567

G 5.58974359 0.41025641

C 3.973168215 0.528895769

A 0 0

C 3.221762567 0.727090385

A 0 0

C 1.98019802 0.538914651

A 0 0

C 7.552377368 0.087818341

C 1.924286253 0.528235442

T 0 0

G 3.388979541 0.100414209

T 0 0

A 0 0

G 4.655844973 1.107336102

T 0 0

C 4.64092567 0.301848824

C 4.840975859 1.13679908

C 2.117676896 0.938371798

A 0 0

G 3.996469995 2.91225416

C 3.398545272 0.84023075

T 0 0

A 0 0

C 5.843260188 0.213166144

C 2.527486415 1.70605333

A 0 0

G 3.101563493 1.461802466

G 7.642172524 1.661341853

G 8.108108108 1.415701416

A 0 0

G 2.822580645 0.705645161

G 8.272290983 1.616569841

C 5.815121829 0.113036925

T 0 0

G 1.749307828 0.679587214

A 0 0

G 3.339634531 0.856962823

G 10.37580666 0.620017715

C 4.081374779 0.29062421

A 0 0

G 2.98902762 1.109849918

G 5.515832482 0.319203269

A 0 0

G 1.072284597 0.504604516

A 0 0

A 0 0

T 0 0

C 0.940085234 0.275758335

A 0 0

T 0 0

T 0 0

T 0 0

G 0.840757937 0.351361526

A 0 0

A 0 0

C 10.91115026 0.667002265

C 3.603830645 0.428427419

C 2.108319657 0.580734756

T 0 0

G 3.97442327 0.351053159

G 3.543058883 0.088261253

A 0 0

G 3.08890005 0.288799598

G 8.401665195 0.100920903

T 0 0

G 10.60738885 0.112711334

G 4.551724138 0.338557994

A 0 0

G 8.040642248 0.288509784

G 10.20691395 0.214483977

T 0 0

G 2.073906486 0.138260432

G 4.599145514 0.18848957

C 3.162403931 0.088194532

A 0 0

C 3.570086434 0.13779281

A 0 0

T 0 0

G 3.34460729 0.150319429

T 0 0

G 2.32295329 0.113008538

T 0 0

A 0 0

A 0 0

G 1.003890074 0.06274313

T 0 0

C 0.150338261 0.037584565

G 0.212712713 0.225225225

T 0 0

T 0 0

T 0 0

A 0 0

C 0.925810084 0.100087577

T 0 0

G 0.563063063 0.237737738

A 0 0

T 0 0

G 1.877816725 0.087631447

C 1.513256628 0.037518759

T 0 0

G 0.350877193 0.112781955

A 0 0

G 0.552001004 0.213273115

A 0 0

T 0 0

C 0.52532833 0.087554722

T 0 0

G 1.428571429 0.213032581

G 3.305785124 0.22539444

G 8.900851277 0.050075113

T 0 0

G 0.90078819 0.025021894

T 0 0

G 0.425745054 0.100175307

A 0 0

A 0 0

T 0 0

G 0.800901014 0.15016894

A 0 0

T 0 0

C 3.569192235 0.037570445

C 2.598870056 0.238543628

C 1.981191223 0.02507837

A 0 0

T 0 0

C 0.588308925 0.062586056

A 0 0

C 8.586111807 0.037603409

C 2.795186764 0.175482577

C 1.177797269 0.162886856

A 0 0

G 1.002380654 0.037589275

A 0 0

C 0.851703407 0.0250501

A 0 0

G 1.342366077 0.163091206

T 0 0

G 0.990968389 0.275965881

C 0.062656642 0.137844612

G 0 0

C 1.440561193 0.112739572

A 0 0

T 0 0

A 0 0

G 5.433149725 0.075112669

T 0 0

A 0 0

C 10.91045899 0.05016303

C 3.430079156 0.25128785

C 1.982931727 0.087851406

A 0 0

A 0 0

T 0 0

C 0.163583742 0.075500189

G 0.188158555 0.175614651

T 0 0

T 0 0

G 0.778307808 0.175746924

G 2.820609252 0.526513727

C 2.654978084 0

T 0 0

T 0 0

T 0 0

T 0 0

C 0.338176353 0.425851703

A 0 0

A 0 0

C 9.634176898 0.025056377

C 3.992968358 0.439477649

C 1.90547825 0.050144164

T 0 0

T 0 0

T 0 0

T 0 0

C 0.58912008 0.075206819

T 0 0

G 2.715614785 0.025144581

C 7.018867925 0.150943396

C 1.97633434 0.176233635

T 0 0

C 1.291050389 0.037603409

T 0 0

T 0 0

C 6.076932715 0.062648791

C 6.220995861 0.087796313

C 3.096014039 0.050137879

T 0 0

T 0 0

C 6.843820506 0.062672349

C 7.50125439 0.075263422

C 3.053531038 0.050263885

T 0 0

C 1.453451948 0.175416614

T 0 0

C 1.066499373 0.112923463

T 0 0

A 0 0

G 2.111083187 0.175923599

C 1.678146525 0.050093926

A 0 0

G 5.710341365 0.16315261

T 0 0

C 6.694245957 0.22564874

C 9.123995984 0.301204819

C 6.842962777 0.501315954

C 3.44350886 0.452431821

A 0 0

G 2.108585859 0.340909091

T 0 0

G 1.899610014 0.050320795

T 0 0

C 0.89039378 0.012540757

T 0 0

G 1.441102757 0.964912281

T 0 0

T 0 0

G 2.072083386 0.138138892

T 0 0

T 0 0

G 1.154328733 0.941028858

T 0 0

C 1.304238776 0.075244545

A 0 0

T 0 0

C 0.550826239 0.225338007

T 0 0

T 0 0

T 0 0

A 0 0

T 0 0

C 0.67728584 0.213219616

T 0 0

C 1.164537941 0.288004007

T 0 0

A 0 0

C 6.047326906 0.075122073

A 0 0

T 0 0

G 9.796021153 0.100730295

T 0 0

A 0 0

C 10.59402194 0.201790894

C 2.502200428 0.150886458

T 0 0

G 0.540472599 0.791855204

A 0 0

T 0 0

G 2.169278997 0.463949843

T 0 0

T 0 0

T 0 0

A 0 0

G 2.508780733 1.078775715

C 2.669842066 0.250689396

T 0 0

C 4.438871473 0.188087774

C 3.546277666 0.176056338

C 1.593875502 0.991465863

A 0 0

C 1.840260391 0.087631447

T 0 0

T 0 0

A 0 0

C 1.001878522 0.125234815

A 0 0

A 0 0

A 0 0

T 0 0

G 0.91697023 0.200979776

A 0 0

G 0.85459344 0.113107955

A 0 0

A 0 0

C 4.381866397 0.075767142

A 0 0

T 0 0

G 1.678356713 0.175350701

T 0 0

G 0.389986162 0.075481193

A 0 0

A 0 0

A 0 0

T 0 0

T 0 0

T 0 0

G 0.663661407 0.175306787

G 1.785714286 0.264084507

T 0 0

T 0 0

T 0 0

T 0 0

C 0.22556391 0.112781955

T 0 0

G 1.314307172 0.062586056

T 0 0

T 0 0

C 3.781671499 0.063027858

C 1.432880845 0.238813474

T 0 0

G 1.540209569 0.08837268

T 0 0

A 0 0

T 0 0

T 0 0

A 0 0

A 0 0

T 0 0

T 0 0

C 0.328573234 0.227473777

A 0 0

C 1.229765341 0.564688167

T 0 0

T 0 0

A 0 0

G 1.405798921 0.426760387

G 3.027232426 0.633312223

A 0 0

T 0 0

A 0 0

A 0 0

T 0 0

G 8.269351794 0.226557583

G 9.833585477 0.605143722

C 10.97115506 0.579418063

C 6.057934509 0.100755668

T 0 0

C 3.578154426 0.251098556

C 2.360813029 0.593359424

A 0 0

G 3.121852971 0.465760322

C 2.850655903 0.491927346

T 0 0

G 1.836940111 0.276799195

C 2.122049221 0.364138624

A 0 0

T 0 0

C 2.835277882 0.351273366

C 2.791750503 0.251509054

A 0 0

T 0 0

G 3.819488214 0.516828438

T 0 0

T 0 0

G 1.196473552 0.629722922

C 1.543480989 0.552139541

T 0 0

G 1.265822785 0.250657977

T 0 0

A 0 0

A 0 0

A 0 0

T 0 0

G 1.169958485 0.226443578

A 0 0

C 1.154908361 0.100426814

A 0 0

G 1.317936488 0.313794402

G 3.525116455 0.163666121

A 0 0

T 0 0

G 1.354401806 0.15048909

T 0 0

A 0 0

A 0 0

T 0 0

T 0 0

A 0 0

T 0 0

T 0 0

G 1.910027645 0.100527771

T 0 0

A 0 0

T 0 0

A 0 0

T 0 0

G 7.347760443 0.037745345

G 5.191050779 0.377073906

C 4.685728681 0.113364404

T 0 0

G 1.956848971 0.050175615

C 5.80474934 0.226159065

A 0 0

T 0 0

A 0 0

G 3.690685413 0.313833794

T 0 0

A 0 0

C 5.911823647 0.400801603

T 0 0

C 2.192707681 0.087708307

C 5.456598093 0.200702459

A 0 0

T 0 0

G 0.81771292 1.270600075

A 0 0

T 0 0

G 4.192293209 0.050207104

T 0 0

A 0 0

T 0 0

A 0 0

T 0 0

G 12.6003009 1.466900702

T 0 0

A 0 0

C 7.658505964 0.163214062

C 2.289308176 0.578616352

A 0 0

A 0 0

G 0.641590137 0.830293119

A 0 0

T 0 0

G 4.913912278 0.955133844

G 2.13971051 1.636249213

A 0 0

G 2.863242497 0.803717192

T 0 0

T 0 0

T 0 0

T 0 0

A 0 0

A 0 0

A 0 0

A 0 0

C 0.201207243 0.075452716

A 0 0

G 0.552624969 0.175835217

A 0 0

A 0 0

G 0.552001004 0.075272864

A 0 0

A 0 0

A 0 0

A 0 0

T 0 0

T 0 0

G 0.57745418 0.263620387

A 0 0

G 2.191609267 0.062617408

T 0 0

A 0 0

T 0 0

G 6.382179952 0.025028157

T 0 0

T 0 0

T 0 0

A 0 0

T 0 0

T 0 0

T 0 0

T 0 0

C 0.350657483 0.388227927

T 0 0

A 0 0

A 0 0

G 2.710163112 0.188205772

G 7.815052142 0.062821963

G 6.809900741 0.25128785

A 0 0

C 4.96054115 0.025053238

A 0 0

C 7.624890447 0.037561037

C 2.025412002 0.138382186

T 0 0

C 0.790960452 0.050219711

A 0 0

G 0.866834171 0.138190955

A 0 0

G 1.153026695 0.175460584

A 0 0

G 1.457652677 1.005277708

A 0 0

G 3.422768305 0.363590772

G 8.11049445 0.214429869

G 7.224525694 1.017715793

A 0 0

G 1.880406168 0.288328946

A 0 0

A 0 0

G 2.415886668 0.480647609

G 7.237999497 0.27645137

T 0 0

G 1.116407426 0.426492725

T 0 0

C 0.953456279 0.025090955

A 0 0

G 4.381572359 0.087631447

T 0 0

A 0 0

C 2.883650953 0.062688064

A 0 0

A 0 0

T 0 0

G 0.613573754 0.11269722

A 0 0

A 0 0

A 0 0

G 3.1269622 0.251161623

G 5.776491316 0.07550969

A 0 0

G 5.292863414 0.175592625

G 6.564386318 0.150905433

G 6.247642992 0.100565682

A 0 0

A 0 0

T 0 0

G 1.067570962 0.326551118

A 0 0

T 0 0

C 0.426011778 0.238065405

A 0 0

A 0 0

T 0 0

A 0 0

G 1.189135061 0.663412192

A 0 0

G 2.01754386 0.162907268

C 2.130592806 0.125328989

A 0 0

A 0 0

G 3.649818136 0.551862536

T 0 0

C 4.87314745 0.087917609

C 4.874055416 0.188916877

C 2.547690763 0.200803213

A 0 0

C 1.56700514 0.062680206

T 0 0

G 0 0

A 0 0

T 0 0

G 1.515151515 0.788880541

A 0 0

G 2.669842066 0.238154926

G 10.9870814 0.213219616

T 0 0

A 0 0

G 1.204516939 0.138017566

A 0 0

A 0 0

G 5.14994306 0.126534228

G 8.551307847 0.326961771

G 8.444500378 0.578907626

G 7.109601107 0.465584497

A 0 0

T 0 0

A 0 0

A 0 0

G 0.639739087 0.551931761

A 0 0

T 0 0

T 0 0

C 1.242158093 0.062735257

A 0 0

T 0 0

A 0 0

G 0.801302116 0.726180043

A 0 0

A 0 0

A 0 0

G 1.128526646 0.827586207

G 7.210935541 0.652119388

T 0 0

T 0 0

G 1.704260652 0.614035088

G 2.67487128 0.22604546

A 0 0

G 1.393947005 0.037674243

A 0 0

G 1.948215184 0.414781297

G 5.914113511 0.075339026

A 0 0

A 0 0

A 0 0

G 1.792878636 0.213139418

C 1.380868692 0.07532011

A 0 0

G 2.592360676 0.050093926

T 0 0

A 0 0

T 0 0

C 0.904295403 0.025119317

T 0 0

T 0 0

C 8.250659299 0.100464649

C 8.649602824 0.100870004

C 7.033408691 0.113036925

C 3.087736915 0.025103552

T 0 0

A 0 0

A 0 0

A 0 0

T 0 0

A 0 0

C 0.926273626 0.225309801

A 0 0

A 0 0

A 0 0

G 2.98320381 0.726999248

G 5.292934373 0.18858436

A 0 0

G 2.020328774 0.3388129

A 0 0

G 1.191521385 0.363727581

A 0 0

A 0 0

G 1.685110664 0.238933602

G 6.493506494 0.189131257

T 0 0

T 0 0

A 0 0

A 0 0

A 0 0

A 0 0

T 0 0

G 4.442213578 0.087840381

T 0 0

T 0 0

T 0 0

T 0 0

T 0 0

A 0 0

A 0 0

C 1.342702974 0.050194504

A 0 0

A 0 0

T 0 0

T 0 0

G 0.942329438 0.30154542

G 2.726130653 0.288944724

T 0 0

T 0 0

T 0 0

T 0 0

G 0.476608554 0.163050295

T 0 0

C 0.113079533 0.075386355

G 0.100628931 0.490566038

G 2.945619335 0.088116818

T 0 0

G 0.802005013 0.15037594

A 0 0

G 1.252975818 0.238065405

A 0 0

G 2.194632556 0.15048909

G 7.058528008 0.200954534

C 3.676747396 0.150583511

A 0 0

A 0 0

A 0 0

G 1.56641604 0.213032581

A 0 0

C 4.028108922 0.200778015

C 1.361058601 0.315059861

A 0 0

A 0 0

G 0.752728641 0.451637185

A 0 0

T 0 0

C 1.192742038 0.02537749

T 0 0

C 4.58254865 0.11299435

C 2.057974652 0.138034885

T 0 0

A 0 0

C 0.388568564 0.150413638

G 0.251224721 0.175857304

T 0 0

T 0 0

A 0 0

T 0 0

T 0 0

T 0 0

A 0 0

T 0 0

G 11.40670097 0.200778015

T 0 0

T 0 0

T 0 0

T 0 0

T 0 0

C 0.477267018 0.150715901

A 0 0

A 0 0

G 0.79135787 0.18841854

T 0 0

T 0 0

A 0 0

T 0 0

T 0 0

C 0.526843954 0.100351229

A 0 0

T 0 0

T 0 0

T 0 0

C 0.616740088 0.088105727

T 0 0

T 0 0

G 2.436879789 0.138173596

G 5.297679112 0.59283552

G 6.532472465 0.139258134

G 5.837851469 0.138696255

G 13.35263025 0.088094639

T 0 0

T 0 0

G 2.197112367 0.539861896

G 3.783579266 0.264850549

G 4.709141274 0.289599597

A 0 0

A 0 0

T 0 0

A 0 0

C 0.263421977 0.539387858

G 0.240020212 0.846387064

G 4.253976269 0.02524615

T 0 0

C 1.456371626 0.414312618

T 0 0

C 0.726452906 0.538577154

A 0 0

C 1.866700075 0.576296668

T 0 0

C 1.151727591 0.375563345

T 0 0

G 1.47814105 0.626330953

T 0 0

T 0 0

G 4.668674699 0.225903614

C 7.144650929 0.17579106

C 3.699509249 0.440417768

C 1.960045232 0.326674205

A 0 0

G 0.930232558 0.276555625

A 0 0

C 1.442910916 0.100376412

T 0 0

G 3.757225434 0.46494094

G 3.150995715 0.189059743

A 0 0

G 3.942742341 0.163234555

T 0 0

G 1.960537891 0.289053663

C 1.383125864 0.037721614

A 0 0

G 1.526908636 0.287859825

T 0 0

A 0 0

A 0 0

A 0 0

G 1.654550013 0.965154174

C 2.382146439 0.150451354

A 0 0

A 0 0

T 0 0

C 0.576008014 0.062609567

A 0 0

C 1.377065598 0.087631447

A 0 0

T 0 0

C 0.651629073 0.112781955

T 0 0

C 0.676268003 0.100187852

A 0 0

C 1.276595745 0.225281602

T 0 0

G 1.167168675 0.125502008

C 1.027054108 0.137775551

A 0 0

G 0.86574655 0.363864492

T 0 0

T 0 0

T 0 0

T 0 0

G 0.202096754 0.606290261

A 0 0

T 0 0

C 0.90146488 0.22536622

T 0 0

C 4.574508084 0.250657977

C 3.236733158 0.727637687

C 2.246204041 0.376458778

A 0 0

G 1.748647629 0.176122783

G 7.430301501 0.403683613

C 7.090863454 0.175702811

T 0 0

C 1.015801354 0.388763481

A 0 0

A 0 0

G 1.094890511 0.176189278

C 0.300827275 0.977688644

G 0.088083554 0.528501321

A 0 0

T 0 0

C 3.804143126 0.40175769

C 2.433517311 0.501756147

T 0 0

C 7.651847214 0.125234815

C 5.7530461 0.703429217

C 3.422675223 0.251667296

A 0 0

C 9.886121887 0.175197097

C 2.25440806 0.23929471

T 0 0

C 0.890058919 0.150432493

A 0 0

G 6.720261274 0.414520789

C 7.21352536 0.425798372

C 2.339622642 0.037735849

T 0 0

T 0 0

C 1.052499687 0.27565468

C 0.289235412 0.201207243

G 0.214051876 0.176278016

A 0 0

G 2.194082247 0.162988967

T 0 0

A 0 0

G 3.827330907 0.627431296

C 3.344188377 0.751503006

T 0 0

G 1.769356255 0.188229389

G 3.293112117 0.251382604

G 3.507669097 0.251445813

A 0 0

T 0 0

T 0 0

G 1.518574297 0.15060241

C 1.016183666 0.138000251

A 0 0

G 1.692789969 0.213166144

G 5.486347049 0.125833648

C 0.376647834 0.11299435

G 0.288582183 0.138017566

T 0 0

T 0 0

T 0 0

G 2.169007021 0.288365095

C 3.354612592 0.21279259

C 1.528630497 0.175416614

A 0 0

C 5.409466566 0.162784874

C 1.612700013 0.176389064

A 0 0

C 3.531621791 0.025046963

A 0 0

T 0 0

C 2.307210031 0.150470219

C 1.180904523 0.06281407

A 0 0

G 2.03722334 0.213782696

C 1.891519479 0.363271953

T 0 0

A 0 0

A 0 0

T 0 0

T 0 0

T 0 0

T 0 0

T 0 0

A 0 0

G 0.050043788 97.73551858

T 0 0

T 0 0

T 0 0

T 0 0

T 0 0

T 0 0

G 1.156505343 0.037712131

T 0 0

A 0 0

G 0.540133149 0.100489888

A 0 0

A 0 0

A 0 0

C 2.7060887 0.175394638

A 0 0

T 0 0

G 3.077116302 0.07535795

G 4.433559407 0.062798292

T 0 0

G 1.495914519 0.100565682

T 0 0

C 1.75636683 0.150545728

A 0 0

C 2.189963709 0.175197097

T 0 0

A 0 0

T 0 0

T 0 0

T 0 0

G 3.253360131 0.138173596

C 6.012777151 0.050106476

C 2.14232022 0.125281884

T 0 0

A 0 0

G 2.098253549 0.25128785

G 6.263363099 0.452773236

C 5.281026028 0.100590972

T 0 0

G 0.833122949 0.896238324

G 4.044858871 0.441028226

T 0 0

C 1.36677116 0.388714734

T 0 0

C 0.714375235 0.050131595

A 0 0

A 0 0

A 0 0

T 0 0

T 0 0

C 3.036767474 0.037645878

C 0.978670013 0.125470514

T 0 0

G 1.745792514 0.07535795

G 3.659916992 0.201232549

G 7.929904186 0.189107413

C 7.699070585 0.050238634

T 0 0

C 0.41327489 0.175328741

A 0 0

A 0 0

A 0 0

A 0 0

A 0 0

A 0 0

T 0 0

C 2.380952381 0.213032581

C 1.693214599 0.163050295

T 0 0

C 5.24733876 0.037570445

C 2.480270575 0.212952524

T 0 0

G 4.022052374 0.025059516

C 7.53913588 0.037570445

C 2.069224981 0.075244545

T 0 0

C 0.763741079 0.175284838

A 0 0

G 5.025062657 0.012531328

C 7.426424546 0.250469631

C 2.392584242 0.100212953

T 0 0

C 1.141065831 0.188087774

T 0 0

C 0.450845335 0.200375704

A 0 0

G 0.715092209 0.062727387

A 0 0

G 2.182639237 0.401404917

T 0 0

G 1.854636591 0.162907268

T 0 0

T 0 0

G 1.831270523 0.416771912

G 3.028018595 0.552833271

G 2.802916038 0.515334339

A 0 0

T 0 0

T 0 0

A 0 0

T 0 0

A 0 0

G 1.015037594 0.914786967

A 0 0

T 0 0

G 1.364376017 0.976342471

T 0 0

G 0.804222166 0.603166625

A 0 0

A 0 0

C 4.587040983 0.714375235

C 1.348796168 0.592461868

A 0 0

C 1.754825771 0.213085986

T 0 0

G 7.164328657 0.688877756

T 0 0

A 0 0

C 13.6989732 0.125219133

C 5.363015431 0.771565899

C 2.607381282 1.032875677

A 0 0

G 4.961299327 0.203019921

C 7.179680624 0.666415189

C 1.853262249 0.444275197

T 0 0

C 0.760552668 0.418303968

A 0 0

G 0.364184353 0.678136381

A 0 0

T 0 0

T 0 0

A 0 0

T 0 0

T 0 0

T 0 0

T 0 0

T 0 0

A 0 0

T 0 0

A 0 0

T 0 0

T 0 0

T 0 0

A 0 0

G 2.811597841 0.251035522

G 3.546723683 0.742045026

A 0 0

G 1.866933968 0.238065405

G 8.503144654 0.13836478

T 0 0

A 0 0

T 0 0

C 10.78812179 0.200476131

T 0 0

C 3.2427695 0.250406911

C 12.29939819 0.062688064

A 0 0

T 0 0

A 0 0

T 0 0

C 0.480951778 0.113909632

T 0 0

T 0 0

A 0 0

A 0 0

A 0 0

C 0.388227927 0.150281778

A 0 0

A 0 0

A 0 0

C 5.268452156 0.33949453

A 0 0

T 0 0

G 2.335803089 1.205575788

T 0 0

G 1.368658965 0.226017077

T 0 0

T 0 0

C 1.053291536 0.438871473

T 0 0

C 0.764315249 0.451071294

A 0 0

C 1.390628915 0.087697319

T 0 0

T 0 0

A 0 0

T 0 0

A 0 0

A 0 0

T 0 0

A 0 0

C 1.227147508 0.275482094

A 0 0

G 0.78977059 0.426225398

A 0 0

T 0 0

A 0 0

A 0 0

T 0 0

A 0 0

T 0 0

G 17.55706045 0.200652119

C 4.695717506 0.07513148

T 0 0

C 0.977688644 0.062672349

T 0 0

A 0 0

G 3.492901118 0.25128785

C 2.720661986 0.08776329

T 0 0

G 0.502008032 0.928714859

A 0 0

T 0 0

G 0.715451236 0.06275888

A 0 0

G 0.703517588 0.163316583

A 0 0

A 0 0

A 0 0

G 1.055541593 0.100527771

C 1.231465192 0.100527771

A 0 0

A 0 0

A 0 0

T 0 0

A 0 0

A 0 0

T 0 0

T 0 0

G 0.70289946 0

G 1.318598518 0.11302273

A 0 0

A 0 0

A 0 0

A 0 0

A 0 0

A 0 0

A 0 0

C 0.590674877 0.087972854

A 0 0

C 1.07918183 0.075291756

T 0 0

G 1.465981707 0.100238065

G 2.350722816 0.113136392

A 0 0

A 0 0

A 0 0

C 0.901803607 0.0250501

T 0 0

T 0 0

C 1.979205812 0.100212953

C 0.928714859 0.062751004

A 0 0

G 1.253446979 0.087741289

G 3.154455197 0.087972854

A 0 0

C 1.305384712 0.163173089

A 0 0

A 0 0

A 0 0

A 0 0

T 0 0

G 0.888944535 0.012520346

A 0 0

A 0 0

C 11.87969925 0.162907268

A 0 0

T 0 0

A 0 0

G 1.466716811 0.087752288

G 3.601108033 0.100730295

A 0 0

A 0 0

T 0 0

A 0 0

A 0 0

A 0 0

A 0 0

C 0.526645768 0.07523511

T 0 0

G 0.437937938 0.137637638

A 0 0

A 0 0

G 1.718514802 0.13798294

T 0 0

A 0 0

G 0.751314801 0.212872527

A 0 0

A 0 0

T 0 0

G 1.189283926 0.075112669

A 0 0

G 1.64057608 0.100187852

G 5.045813983 0.138069537

A 0 0

A 0 0

G 1.704901592 0.288328946

A 0 0

G 2.167376597 0.375845653

G 4.973000126 0.288835866

A 0 0

A 0 0

C 2.15755143 0.23833417

T 0 0

A 0 0

G 1.103448276 0.326018809

A 0 0

A 0 0

C 1.052631579 0.187969925

A 0 0

A 0 0

G 1.921145153 0.188347564

G 4.795380367 0.338940497

A 0 0

A 0 0

G 1.793103448 0.10031348

A 0 0

G 5.153729839 0.403225806

G 7.835962145 0.252365931

A 0 0

C 10.40070343 0.301469665

C 4.189343121 0.493608404

C 2.296240222 0.126167045

A 0 0

G 0.755001888 0.490751227

A 0 0

A 0 0

G 1.597363083 0.393002028

T 0 0

A 0 0

A 0 0

C 0.802005013 0.413533835

A 0 0

A 0 0

A 0 0

T 0 0

C 0.338685399 0.351229303

A 0 0

G 0.376695128 0.125565043

A 0 0

G 2.079158317 0.237975952

T 0 0

A 0 0

A 0 0

T 0 0

G 6.519834814 0.30033788

C 2.072343632 0.113036925

T 0 0

G 0.753106565 0.263587298

G 2.00929298 0.301393947

A 0 0

A 0 0

A 0 0

A 0 0

T 0 0

C 1.944792974 0.138017566

C 0.501882058 0.903387704

T 0 0

G 0.88883325 0.237856785

T 0 0

C 1.152448954 0.112739572

A 0 0

G 2.557673019 0.112838516

G 8.011534604 0.225677031

G 7.724242043 0.30192477

A 0 0

G 4.096717615 0.250563768

G 6.717171717 0.353535354

G 6.058697569 0.39047739

A 0 0

A 0 0

T 0 0

G 3.272910373 0.906344411

G 3.642758447 1.080266298

T 0 0

T 0 0

A 0 0

A 0 0

G 7.706790511 0.200828417

C 8.390819014 0.326100589

C 2.449748744 1.155778894

T 0 0

A 0 0

T 0 0

G 10.20304569 0.736040609

T 0 0

C 2.337398374 0.406504065

T 0 0

C 1.129584973 0.12691966

A 0 0

G 0 0

T 0 0

A 0 0

A 0 0

C 3.642727508 2.281565984

C 2.225693089 0.312377977

A 0 0

T 0 0

G 0 0

A 0 0

A 0 0

C 1.28440367 1.19266055

A 0 0

A 0 0

A 0 0

G 4.873190811 0.398353472

G 12.80382521 0.624252889

G 9.328010645 0.279441118

C 16.49580615 1.41126348

C 7.274413674 0.159003578

A 0 0

A 0 0

A 0 0

G 0.598722725 0.199574242

A 0 0

A 0 0

A 0 0

C 8.903636112 0.235033873

C 4.395150179 0.606227611

C 2.451452968 0.041316623

T 0 0

T 0 0

C 1.200496757 0.12418932

A 0 0

T 0 0

C 1.592531576 0.604063701

C 0.892244338 0.123541524

A 0 0

A 0 0

T 0 0

C 1.013650493 0.081092039

C 0.498450761 0.30984777

G 0.418410042 0.944796869

G 3.35308376 0.363587396

T 0 0

G 0 0

T 0 0

T 0 0

G 1.444774507 0.675128274

G 2.524298056 0.458963283

G 3.056004308 0.861604739

A 0 0

A 0 0

A 0 0

C 0.799566337 0.528526901

T 0 0

G 0.688895012 0.509782309

T 0 0

T 0 0

A 0 0

A 0 0

C 0.36131184 0.250138966

G 0.626915575 0.083588743

A 0 0

C 0.978610373 0.167761778

A 0 0

C 0.699888018 0.055991041

A 0 0

G 0.593136563 0.15534529

A 0 0

A 0 0

G 1.058049757 0.171575636

T 0 0

C 0.986794369 0.043535046

A 0 0

T 0 0

C 0.550724638 0.115942029

T 0 0

G 0.378512156 0.276605037

T 0 0

T 0 0

T 0 0

G 1.690679212 0.1764187

C 1.613613026 0.073346047

T 0 0

C 0.647249191 0.044130627

A 0 0

A 0 0

T 0 0

G 0.384217526 0.650214275

A 0 0

T 0 0

T 0 0

C 0.462893833 0.074660296

A 0 0

C 2.190876351 0.030012005

A 0 0

T 0 0

T 0 0

C 4.306802618 0.015218384

C 2.021129995 0

T 0 0

T 0 0

A 0 0

G 0.652883569 0.310896938

A 0 0

G 1.017488076 1.128775835

A 0 0

G 2.342606149 1.106230682

G 7.384968069 0.392991649

G 5.119341564 1.41563786

A 0 0

G 1.330008313 1.014131338

T 0 0

T 0 0

C 1.101738681 0.051644001

T 0 0

C 1.385521302 0.207828195

A 0 0

G 1.014163315 0.611995104

A 0 0

C 3.534376648 0.140671707

C 1.269670959 1.555793991

A 0 0

A 0 0

T 0 0

C 0.655021834 0.127365357

A 0 0

T 0 0

T 0 0

T 0 0

T 0 0

G 0 0

A 0 0

A 0 0

A 0 0

A 0 0

C 0.798023941 0.1900057

T 0 0

C 0.403380715 0.038417211

A 0 0

G 0.811907984 0.212642567

T 0 0

G 0.291318703 0.213633715

A 0 0

T 0 0

T 0 0

T 0 0

T 0 0

C 0.257171118 0.098911968

A 0 0

C 1.712806214 0.039832703

A 0 0

T 0 0

T 0 0

C 0.707499495 0.101071356

A 0 0

T 0 0

T 0 0

A 0 0

A 0 0

T 0 0

T 0 0

C 0.31282586 0.020855057

A 0 0

G 0.581145071 0.538097288

T 0 0

G 0.719110917 0.740902157

A 0 0

G 2.351897049 0.44375416

T 0 0

G 2.569303584 1.262114041

G 3.324225865 0.364298725

A 0 0

C 2.547475683 0.254747568

T 0 0

C 0.890922225 0.168552853

T 0 0

T 0 0

T 0 0

A 0 0

A 0 0

A 0 0

T 0 0

C 0.368227249 0.105207785

T 0 0

G 0.823395798 3.038046564

G 1.542416452 3.02770637

A 0 0

T 0 0

G 1.540697674 5.930232558

T 0 0

C 1.929928741 0.178147268

A 0 0

T 0 0

T 0 0

G 0 0

G 3.519798869 1.571338781

A 0 0

C 5.844778026 1.564995209

C 2.742103436 1.214855953

A 0 0

G 3.066566941 1.159311892

C 8.094879518 6.09939759

C 4.309687262 0.305110603

A 0 0

T 0 0

T 0 0

A 0 0

A 0 0

A 0 0

T 0 0

C 0.597426471 0.413602941

T 0 0

T 0 0

T 0 0

G 1.022494888 3.732106339

G 3.816400206 3.455389376

C 5.711318795 2.128764278

C 1.51187905 0.539956803

G 0.49833887 8.139534884

G 0 0

T 0 0

A 0 0

A PRIMER NA NA

T PRIMER NA NA

T PRIMER NA NA

G PRIMER NA NA

A PRIMER NA NA

C PRIMER NA NA

T PRIMER NA NA

G PRIMER NA NA

A PRIMER NA NA

A PRIMER NA NA

A PRIMER NA NA

T PRIMER NA NA

G PRIMER NA NA

G PRIMER NA NA

A PRIMER NA NA

G PRIMER NA NA

G PRIMER NA NA

A PRIMER NA NA

C PRIMER NA NA

A PRIMER NA NA

T PRIMER NA NA

T PRIMER NA NA

T PRIMER NA NA

T PRIMER NA NA

G PRIMER NA NA

C NA NA

A NA NA

A NA NA

A NA NA

T NA NA

T NA NA

G NA NA

T NA NA

A NA NA

A NA NA

T NA NA

G NA NA

C NA NA

C NA NA

T NA NA

C NA NA

C NA NA

C NA NA

A NA NA

C NA NA

C NA NA

T NA NA

G NA NA

G NA NA

A NA NA

G NA NA

T NA NA

G NA NA

T NA NA

G NA NA

G NA NA

T NA NA

T NA NA

G NA NA

G NA NA

G NA NA

A NA NA

A NA NA

T NA NA

T NA NA

T NA NA

T NA NA

G NA NA

A NA NA

A NA NA

A NA NA

T NA NA

A NA NA

A NA NA

T NA NA

C NA NA

T NA NA

C NA NA

A NA NA

G NA NA

T PRIMER NA NA

T PRIMER NA NA

G PRIMER NA NA

A PRIMER NA NA

A PRIMER NA NA

T PRIMER NA NA

T PRIMER NA NA

T PRIMER NA NA

T PRIMER NA NA

T PRIMER NA NA

C PRIMER NA NA

T PRIMER NA NA

T PRIMER NA NA

C PRIMER NA NA

C PRIMER NA NA

T PRIMER NA NA

T PRIMER NA NA

T PRIMER NA NA

G PRIMER NA NA

A PRIMER NA NA

A PRIMER NA NA

A PRIMER NA NA

A PRIMER NA NA

C PRIMER NA NA

C PRIMER NA NA

A PRIMER NA NA

T 0 0

T 0 0

T 0 0

G 0 0

A 0 0

A 0 0

A 0 0

A 0 0

A 0 0

C 2.957746479 12.3943662

T 0 0

A 0 0

T 0 0

A 0 0

G 2.320185615 1.778808971

A 0 0

A 0 0

A 0 0

G 14.5309626 0.122624157

G 19.80558931 0.546780073

A 0 0

T 0 0

A 0 0

G 3.135888502 0.149328024

T 0 0

G 1.406401552 0.09699321

T 0 0

T 0 0

A 0 0

A 0 0

A 0 0

T 0 0

T 0 0

T 0 0

C 0.685483871 2.620967742

T 0 0

A 0 0

C 1.86382655 2.548497528

T 0 0

G 3.498950315 0.034989503

G 4.175152749 1.697216565

G 8.532934132 0.119760479

T 0 0

T 0 0

T 0 0

A 0 0

G 6.28757515 0.0250501

T 0 0

C 4.818678589 0.645802285

C 5.234567901 0.864197531

C 3.535965125 0.435940906

A 0 0

C 2.337662338 0.25974026

T 0 0

T 0 0

A 0 0

T 0 0

A 0 0

T 0 0

G 2.569213732 0.110741971

A 0 0

A 0 0

A 0 0

A 0 0

G 0.525279054 0.043773255

A 0 0

T 0 0

T 0 0

C 0.5427703 0.390794616

G 0 0

A 0 0

A 0 0

A 0 0

A 0 0

A 0 0

G 1.779359431 0

C 1.788685524 0.062396007

A 0 0

T 0 0

C 0.483578481 0.261938344

T 0 0

T 0 0

T 0 0

C 7.262780607 0.169779287

C 1.781027372 0.318710161

T 0 0

T 0 0

C 3.737280296 0.07400555

C 0.994292027 0.662861352

T 0 0

A 0 0

T 0 0

T 0 0

T 0 0

T 0 0

T 0 0

G 0.403792135 0.087780899

A 0 0

A 0 0

T 0 0

C 0.238785605 1.483881972

G 0.907065563 0.190961171

G 1.42610876 0.720890143

A 0 0

C 5.403736298 4.461942257

C 1.535155051 4.943199263

A 0 0

G 1.552975327 0.290275762

A 0 0

C 1.704945752 2.324926025

A 0 0

G 2.339994941 0.898052112

A 0 0

G 7.11961962 0.963463463

G 13.59939947 0.963342925

A 0 0

G 7.257257257 0.087587588

G 16.57078539 0.050025013

T 0 0

A 0 0

A 0 0

G 6.20465349 0.025018764

C 11.01376721 0.400500626

A 0 0

T 0 0

A 0 0

G 1.489734602 0.012518778

T 0 0

G 0.739255732 0.263124922

A 0 0

G 1.300975732 0.12509382

T 0 0

T 0 0

A 0 0

C 1.676257193 0.012509382

A 0 0

G 3.705094505 0.062586056

G 7.444541923 0.037598697

G 11.50043799 0.025028157

C 9.533341674 0.025021894

T 0 0

G 2.918702242 0.025053238

G 4.419128693 0.062593891

G 10.18773467 0.050062578

C 7.783756726 0

T 0 0

T 0 0

G 1.839109221 0

T 0 0

T 0 0

C 3.453021394 0.025021894

C 0.988612189 0.012514078

T 0 0

G 0.750375188 0.012506253

G 2.065598398 0.13770656

A 0 0

T 0 0

C 0.33809166 0.025043827

A 0 0

G 0.887943972 0.025012506

T 0 0

T 0 0

A 0 0

C 4.495929869 0.037570445

C 1.403157103 0.062640942

A 0 0

C 2.739212008 0.05003127

A 0 0

T 0 0

G 2.74401704 0.075178549

G 4.682023035 0.050075113

C 4.971822167 0.075140889

T 0 0

T 0 0

G 1.17824016 0.037603409

T 0 0

C 1.039448967 0.087664371

T 0 0

C 0.525722869 0.062586056

A 0 0

G 0.826549781 0.062617408

T 0 0

C 0.613036407 0.025021894

A 0 0

A 0 0

A 0 0

C 0.900900901 0.037537538

A 0 0

C 0.776067092 0

T 0 0

T 0 0

T 0 0

G 1.454363089 0.112838516

C 1.364717666 0.11268311

A 0 0

C 1.588095536 0.062523446

A 0 0

C 7.760670923 0.087620478

C 1.770689439 0.062790406

T 0 0

C 4.604028525 0.075065682

C 2.404207363 0.03756574

C 0.626959248 0.12539185

G 1.076884548 0.200350614

T 0 0

T 0 0

C 0.539861896 0.062774639

A 0 0

T 0 0

C 0.488110138 0.100125156

T 0 0

G 1.277235162 0.062609567

G 3.448275862 0.10031348

C 1.652271874 0.100137689

A 0 0

A 0 0

A 0 0

T 0 0

A 0 0

A 0 0

G 4.131089905 0.075339026

G 6.078111265 0.22604546

G 9.953500063 0.113107955

T 0 0

C 2.303743583 0

T 0 0

T 0 0

A 0 0

A 0 0

G 0.902142589 0.162886856

T 0 0

T 0 0

T 0 0

G 0.45112782 0.426065163

A 0 0

T 0 0

G 7.184197282 0.037745345

G 10.58986291 0.477927305

C 10.19666792 0.100212953

C 5.189270494 0.075206819

T 0 0

T 0 0

G 0.917777219 0.150867488

A 0 0

A 0 0

G 0.772445232 0.164619476

A 0 0

T 0 0

A 0 0

A 0 0

A 0 0

C 0.90101364 0.025028157

T 0 0

T 0 0

C 1.943573668 0.087774295

C 0.864878416 0.162948107

A 0 0

G 2.044912809 0.075272864

C 1.731710378 0.188229389

A 0 0

C 1.128809733 0.125423304

T 0 0

A 0 0

A 0 0

A 0 0

T 0 0

T 0 0

G 1.904761905 0.213032581

T 0 0

T 0 0

C 0.764794383 0.075225677

T 0 0

C 0.915590117 0.163050295

T 0 0

A 0 0

G 1.169958485 0.226443578

C 0.518134715 0.075824592

G 0.430107527 0.151802657

T 0 0

C 0.238065405 0.162886856

T 0 0

A 0 0

A 0 0

A 0 0

T 0 0

T 0 0

T 0 0

T 0 0

A 0 0

A 0 0

G 1.145662848 0.037769105

T 0 0

G 0.832072617 0.239536056

A 0 0

C 1.128102281 0.050137879

T 0 0

G 2.69524884 0.075216247

C 5.035790531 0.163255055

A 0 0

T 0 0

A 0 0

G 5.173929424 0.351626272

C 4.381866397 0.315696426

T 0 0

T 0 0

A 0 0

T 0 0

T 0 0

T 0 0

C 0.734456123 0.240597695

A 0 0

A 0 0

G 6.231829099 0.29073442

G 12.87744227 0.380614057

G 12.55117707 0.447799386

C 15.74205174 0.553481787

C 7.374744376 0.677402863

A 0 0

C 1.683205627 0.326592137

T 0 0

T 0 0

T 0 0

T 0 0

A 0 0

T 0 0

A 0 0

T 0 0

T 0 0

G 2.027027027 0.777664457

T 0 0

T 0 0

C 2.906460211 0.329991116

C 4.189733266 0.188726724

A 0 0

T 0 0

A 0 0

A 0 0

A 0 0

A 0 0

A 0 0

T 0 0

G 0.734084293 0.253132515

A 0 0

A 0 0

A 0 0

G 2.20308939 0.126614333

G 11.29500948 0.075805433

T 0 0

A 0 0

T 0 0

G 5.079844084 0.062869357

T 0 0

G 1.729579599 0.126246686

T 0 0

T 0 0

T 0 0

G 1.270163851 0.050806554

G 3.186397985 0.617128463

C 2.694911005 0.062672349

T 0 0

T 0 0

T 0 0

A 0 0

A 0 0

T 0 0

A 0 0

A 0 0

A 0 0

T 0 0

G 3.859649123 0.263157895

T 0 0

G 0.728094401 0.489580718

A 0 0

T 0 0

C 0.725998248 0.21279259

T 0 0

A 0 0

T 0 0

T 0 0

T 0 0

C 0.767199094 0.062885172

A 0 0

A 0 0

G 4.657878217 0.464532329

C 4.003514056 0.125502008

T 0 0

G 2.232814852 0.112895133

C 2.869314622 0.463601052

C 1.798968424 0.138382186

A 0 0

T 0 0

T 0 0

G 0.804626603 0.18858436

A 0 0

A 0 0

G 0.777722027 0.23833417

A 0 0

A 0 0

A 0 0

C 6.401904285 0.087697319

A 0 0

T 0 0

T 0 0

G 0.705023291 0.07553821

A 0 0

A 0 0

T 0 0

G 0.748161299 0.126807

A 0 0

A 0 0

A 0 0

G 0.807071483 0.115295926

T 0 0

T 0 0

T 0 0

C 2.654978084 0.262993112

C 5.014416447 0.137896452

A 0 0

T 0 0

A 0 0

T 0 0

T 0 0

A 0 0

A 0 0

A 0 0

C 10.31894934 0.262664165

A 0 0

T 0 0

T 0 0

A 0 0

C 1.476476476 0.137637638

T 0 0

T 0 0

C 0.664326899 0.050137879

A 0 0

A 0 0

T 0 0

A 0 0

G 1.174218251 0.102105935

A 0 0

G 1.418528747 0.200853628

A 0 0

G 1.495162709 0.15077271

A 0 0

G 1.506591337 0.138104206

A 0 0

G 2.333458788 0.138000251

G 8.209519013 0.163686729

T 0 0

G 1.956603537 0.025084661

T 0 0

T 0 0

C 5.162907268 0.025062657

C 1.859997486 0.025135101

T 0 0

T 0 0

A 0 0

A 0 0

C 0.802206067 0.02506894

A 0 0

G 3.547616052 0.553528746

C 2.659307577 0.037631711

T 0 0

A 0 0

A 0 0

T 0 0

G 4.482283711 0.050081382

T 0 0

A 0 0

A 0 0

T 0 0

A 0 0

A 0 0

T 0 0

T 0 0

A 0 0

A 0 0

C 0.788584303 0.050068845

A 0 0

G 1.480923695 0.338855422

T 0 0

T 0 0

C 0.663661407 0.087653393

A 0 0

T 0 0

T 0 0

C 0.41327489 0.012523482

A 0 0

A 0 0

A 0 0

T 0 0

T 0 0

A 0 0

A 0 0

T 0 0

T 0 0

A 0 0

T 0 0

A 0 0

A 0 0

G 4.483798041 0.07535795

G 4.909365559 0.075528701

A 0 0

G 16.29685345 0.075216247

T 0 0

A 0 0

C 4.668088844 0.012548626

T 0 0

T 0 0

C 0.715810624 0.238603541

T 0 0

C 0.7023705 0.163050295

T 0 0

A 0 0

T 0 0

T 0 0

T 0 0

T 0 0

C 0.576730191 0.388665998

T 0 0

T 0 0

C 0.843085441 0

T 0 0

T 0 0

T 0 0

T 0 0

C 0.325569747 0.087653393

T 0 0

G 0.716260367 0.100527771

G 1.55519029 0.139082058

A 0 0

T 0 0

C 0.664326899 0.313361745

T 0 0

T 0 0

A 0 0

A 0 0

G 0.753106565 0.075310656

A 0 0

A 0 0

T 0 0

A 0 0

A 0 0

A 0 0

C 1.026154424 0.175197097

T 0 0

A 0 0

T 0 0

G 0.601277715 0.162846048

A 0 0

A 0 0

A 0 0

T 0 0

G 5.45112782 0.07518797

T 0 0

T 0 0

A 0 0

A 0 0

T 0 0

T 0 0

T 0 0

C 0.601729974 0.22564874

T 0 0

T 0 0

G 1.932003513 0.125454774

C 1.733450572 0.075367416

T 0 0

T 0 0

A 0 0

G 4.815612723 0.10138132

G 8.416909991 0.050780754

G 11.56669198 0.101240192

C 13.15326633 0.087939698

T 0 0

C 7.554996857 0.02514142

C 3.347759674 0.114562118

T 0 0

G 2.389336016 0.100603622

C 2.011566507 0.163439779

T 0 0

A 0 0

A 0 0

G 2.273869347 0.389447236

T 0 0

A 0 0

T 0 0

C 1.018483591 0.440085502

A 0 0

A 0 0

C 1.351351351 0.1001001

A 0 0

G 1.105249937 0.263752826

T 0 0

T 0 0

T 0 0

T 0 0

C 0.112980166 0.138086869

G 0.239596469 0.466582598

T 0 0

G 1.554858934 0.087774295

T 0 0

G 1.50526844 0.313597592

T 0 0

C 1.214777708 0.175328741

T 0 0

T 0 0

G 0.628930818 0.163522013

A 0 0

A 0 0

A 0 0

C 0.806654903 0.277287623

T 0 0

A 0 0

A 0 0

T 0 0

A 0 0

C 5.960430754 0.15026296

C 1.363636364 0.492424242

T 0 0

A 0 0

A 0 0

A 0 0

T 0 0

A 0 0

T 0 0

T 0 0

T 0 0

C 1.313813814 0.275275275

T 0 0

C 6.760316067 0.150507964

C 7.174268124 0.20103028

C 3.304020101 0.125628141

T 0 0

A 0 0

T 0 0

T 0 0

G 1.33316564 0.226386618

A 0 0

G 2.709514808 0.403276623

G 7.704938739 0.113679424

C 5.325368582 0.076258261

A 0 0

T 0 0

C 0.578034682 0.213621513

T 0 0

T 0 0

T 0 0

A 0 0

T 0 0

T 0 0

A 0 0

A 0 0

A 0 0

G 2.78999623 0.113107955

C 2.195183141 0.363773206

C 0.653594771 0.02513826

A 0 0

G 0.439588043 0.100477267

A 0 0

A 0 0

T 0 0

T 0 0

G 0.790464241 0.062735257

A 0 0

T 0 0

T 0 0

A 0 0

C 4.937343358 0.137844612

C 1.659125189 0.087983912

A 0 0

T 0 0

C 0.589267803 0

T 0 0

T 0 0

A 0 0

A 0 0

G 1.871624168 0.025122472

G 4.060339409 0.100565682

A 0 0

A 0 0

A 0 0

A 0 0

G 1.12895133 0.100351229

T 0 0

G 1.594676042 0.276243094

G 4.137412237 0.413741224

C 3.154058809 0.113093742

T 0 0

T 0 0

A 0 0

C 2.029312289 0.050106476

T 0 0

T 0 0

A 0 0

C 2.00325529 0.075122073

A 0 0

C 1.80338134 0.012523482

T 0 0

A 0 0

G 0.941146944 0.175680763

A 0 0

A 0 0

T 0 0

A 0 0

A 0 0

A 0 0

G 1.527482159 0.187805183

T 0 0

A 0 0

A 0 0

T 0 0

A 0 0

A 0 0

A 0 0

G 1.633371027 0.125643925

T 0 0

C 1.140493796 0.11279609

C 1.003009027 0.476429288

A 0 0

A 0 0

A 0 0

A 0 0

T 0 0

G 4.231347021 0

T 0 0

T 0 0

T 0 0

T 0 0

T 0 0

T 0 0

A 0 0

A 0 0

T 0 0

T 0 0

A 0 0

C 1.853011143 0.062601728

T 0 0

C 6.386449184 0.163111669

C 6.826753835 0.113150616

C 4.68416426 0.075348487

C 2.932662052 0.201384519

A 0 0

A 0 0

T 0 0

A 0 0

A 0 0

A 0 0

C 0.752445448 0.10032606

T 0 0

T 0 0

A 0 0

C 1.329653788 0.050175615

A 0 0

A 0 0

C 5.715718225 0.137879168

A 0 0

T 0 0

T 0 0

T 0 0

T 0 0

G 1.485771846 0.176278016

G 3.283431878 0.088061391

C 3.24683465 0.087752288

A 0 0

T 0 0

T 0 0

G 1.871389098 0.087917609

T 0 0

C 1.039969929 0.037589275

T 0 0

T 0 0

C 0.463542972 0.050112754

A 0 0

G 3.672599649 0.075206819

C 2.968808719 0.100212953

T 0 0

G 1.091319619 0.050175615

T 0 0

G 1.961277345 0.477747046

T 0 0

C 1.467268623 0.10032606

T 0 0

T 0 0

G 1.296249685 0.465643091

A 0 0

C 1.227608668 0.112739572

A 0 0

C 2.852814946 0.02524615

A 0 0

T 0 0

G 3.881307124 0.613496933

T 0 0

A 0 0

T 0 0

T 0 0

T 0 0

A 0 0

T 0 0

A 0 0

C 1.002255074 0.388373841

T 0 0

T 0 0

T 0 0

T 0 0

A 0 0

T 0 0

A 0 0

T 0 0

C 0.376600552 0.251067035

A 0 0

G 0.690434346 0.251067035

A 0 0

G 1.444542143 0.100489888

T 0 0

G 0.627037873 0.714823175

A 0 0

T 0 0

T 0 0

T 0 0

T 0 0

T 0 0

A 0 0

C 1.06449593 0.175328741

A 0 0

C 1.078234704 0.112838516

A 0 0

G 1.79225041 0.100971854

G 5.253164557 0.17721519

A 0 0

A 0 0

G 1.618975904 0.075301205

T 0 0

T 0 0

T 0 0

C 0.488232349 0.075112669

A 0 0

G 3.640472006 0.225960331

C 2.983951856 0.150451354

T 0 0

C 0.514106583 0.12539185

T 0 0

G 1.566808724 0.112810228

T 0 0

A 0 0

A 0 0

T 0 0

T 0 0

A 0 0

G 1.672745567 0.062885172

C 0.977443609 0.15037594

A 0 0

A 0 0

T 0 0

C 1.653513717 0.275585619

C 0.892632638 0.088006035

A 0 0

C 1.365230461 0.03757515

A 0 0

A 0 0

T 0 0

A 0 0

G 0.703782833 0.150810607

A 0 0

A 0 0

A 0 0

C 0.84192008 0.037697914

T 0 0

T 0 0

T 0 0

G 0.79265224 0.075490689

A 0 0

C 0.728917934 0.025135101

A 0 0

C 0.876533934 0.087653393

T 0 0

T 0 0

A 0 0

A 0 0

T 0 0

T 0 0

A 0 0

C 1.114589856 0.050093926

A 0 0

G 3.396893547 0.037883571

C 5.863027546 0.101086682

A 0 0

T 0 0

T 0 0

G 2.020582329 0.439257028

C 1.604814443 0.08776329

T 0 0

A 0 0

A 0 0

G 2.904929577 0.062877264

G 7.146458281 0.201663726

C 3.082148698 0.138382186

A 0 0

A 0 0

T 0 0

A 0 0

A 0 0

T 0 0

A 0 0

A 0 0

T 0 0

A 0 0

T 0 0

G 1.998994217 0.150867488

A 0 0

G 0.57817999 0.226244344

A 0 0

A 0 0

A 0 0

T 0 0

C 1.405798921 0.213380193

C 0.615887381 0.175967823

T 0 0

A 0 0

A 0 0

A 0 0

C 0.590229813 0.037674243

T 0 0

T 0 0

G 0.967579794 0.087961799

A 0 0

A 0 0

T 0 0

G 3.258962146 0.137879168

C 1.667920742 0.087785302

A 0 0

G 1.64160401 0.050125313

T 0 0

A 0 0

T 0 0

T 0 0

T 0 0

C 0.780365009 0.251730648

T 0 0

T 0 0

G 1.534977353 0.075490689

A 0 0

C 1.18149824 0.175967823

A 0 0

C 1.58013544 0.087785302

A 0 0

C 13.48328726 0.062829857

C 7.97074032 0.201790894

C 7.848069425 0.138347378

C 5.079844084 0.100590972

T 0 0

A 0 0

G 4.833040422 0.251067035

C 4.022261573 0.139134834

T 0 0

A 0 0

C 9.185463659 0.112781955

C 2.429200755 0.327249843

T 0 0

T 0 0

C 0.989478958 0.03757515

T 0 0

T 0 0

A 0 0

T 0 0

T 0 0

G 1.434142659 0.213863379

G 3.782832726 0.842025889

C 2.954488308 0.050289163

T 0 0

G 2.275298554 0.188560654

G 4.107862903 0.856854839

G 4.853756934 0.201714574

A 0 0

A 0 0

A 0 0

C 1.541739784 0.062672349

A 0 0

A 0 0

T 0 0

T 0 0

A 0 0

T 0 0

A 0 0

T 0 0

A 0 0

T 0 0

G 2.990325418 0.05025757

A 0 0

C 2.05694218 0.075253982

T 0 0

A 0 0

G 2.493109496 0.125281884

T 0 0

A 0 0

A 0 0

A 0 0

T 0 0

A 0 0

A 0 0

T 0 0

C 1.89057218 0.388130712

C 2.075732797 0.10064159

A 0 0

T 0 0

A 0 0

C 1.427855711 0.062625251

T 0 0

G 0.477267018 0.150715901

A 0 0

A 0 0

A 0 0

T 0 0

G 0.989850896 0.037589275

A 0 0

A 0 0

T 0 0

G 3.774294671 0.087774295

T 0 0

G 2.830306825 0.125234815

T 0 0

A 0 0

T 0 0

G 0.902029567 0.075169131

A 0 0

A 0 0

A 0 0

A 0 0

A 0 0

A 0 0

G 2.478922864 0.02516673

G 6.892641129 0.163810484

C 3.913697943 0.112895133

A 0 0

A 0 0

C 3.910754575 0.188017047

A 0 0

T 0 0

G 4.05015674 0.02507837

C 3.836028582 0.062680206

A 0 0

T 0 0

T 0 0

T 0 0

A 0 0

C 1.21492986 0.01252505

A 0 0

A 0 0

C 1.256755058 0.062837753

A 0 0

G 2.219157472 0.112838516

G 10.54220657 0.088061391

T 0 0

A 0 0

C 3.792714983 0.050068845

T 0 0

T 0 0

C 0.916394677 0.163193573

T 0 0

A 0 0

G 1.818409832 0.125407575

T 0 0

T 0 0

A 0 0

G 4.085994468 0.201156651

G 8.614185111 0.226358149

C 6.251569169 0.087873462

C 2.78789401 0.163255055

A 0 0

A 0 0

G 1.306696821 0.10051514

T 0 0

T 0 0

C 0.601805416 0.100300903

A 0 0

G 1.453087812 0.087686333

T 0 0

C 0.681990402 0.088406163

A 0 0

C 0.802709143 0.188134955

A 0 0

G 4.479859455 0.263521144

C 3.512293026 0.37631711

T 0 0

A 0 0

C 1.190923906 0.087752288

T 0 0

G 0.376175549 0.238244514

A 0 0

T 0 0

T 0 0

T 0 0

G 1.683205627 0.288908429

G 2.218601153 0.388568564

A 0 0

C 1.527673428 0.1878287

T 0 0

A 0 0

A 0 0

A 0 0

A 0 0

C 1.40969163 0.239144116

G 1.294620412 0.213675214

T 0 0

T 0 0

A 0 0

T 0 0

G 9.032663317 0.050251256

G 5.789076265 0.213944123

G 10.14839034 0.050301811

C 4.775034465 0.125328989

A 0 0

G 2.524808441 0.301469665

C 1.835323696 0.301697046

A 0 0

G 3.393238658 0.138243056

C 3.159478435 0.376128385

C 1.204819277 0.062751004

A 0 0

A 0 0

G 2.69761606 0.464240903

G 5.936990084 0.364001506

A 0 0

G 2.673863922 0.225960331

A 0 0

A 0 0

C 1.638104839 0.075604839

A 0 0

T 0 0

C 0.592536561 0.239536056

A 0 0

T 0 0

C 0.613958151 0.100238065

A 0 0

A 0 0

A 0 0

G 1.479438315 0.300902708

A 0 0

C 1.490480962 0.125250501

T 0 0

T 0 0

C 0.652446675 0.125470514

T 0 0

C 0.751503006 0.187875752

T 0 0

A 0 0

G 1.342366077 0.163091206

A 0 0

C 1.729106628 0.13782734

T 0 0

C 0.338770389 0.112923463

A 0 0

A 0 0

A 0 0

A 0 0

G 4.082146907 0.050396875

G 9.520814992 0.176078481

C 8.127119709 0.075367416

T 0 0

T 0 0

C 2.68338558 0.150470219

C 1.939058172 0.125912868

A 0 0

C 0.713570356 0.175262894

G 1.133215815 0.251825737

T 0 0

T 0 0

C 0.952380952 0.15037594

T 0 0

A 0 0

C 3.431433939 0.087664371

A 0 0

T 0 0

C 0.730110775 0.062940584

T 0 0

T 0 0

G 1.33316564 0.08803924

A 0 0

G 3.441778671 0.037683708

C 2.670846395 0.10031348

A 0 0

T 0 0

C 0.913756415 0.150206534

T 0 0

T 0 0

C 1.042189854 0.414364641

T 0 0

A 0 0

C 5.185370741 0.263026052

C 1.596680915 0.201156651

A 0 0

C 1.316944688 0.200677286

T 0 0

C 0.90146488 0.062601728

C 0.327744863 0.352956007

G 0.504477235 0.227014756

A 0 0

A 0 0

T 0 0

T 0 0

G 1.411824026 0.315139292

A 0 0

A 0 0

C 3.398971529 0.501693215

C 0.919048219 0.176255823

A 0 0

G 1.619992465 0.075348487

T 0 0

C 0.852130326 0.025062657

T 0 0

T 0 0

C 0.564051141 0.363499624

A 0 0

A 0 0

A 0 0

G 1.167022211 0.100389007

T 0 0

A 0 0

A 0 0

A 0 0

G 2.486500063 0.062790406

G 6.983767459 0.125833648

C 4.148334381 0.150848523

A 0 0

A 0 0

T 0 0

G 4.231010672 0.075329567

G 4.488872124 0.201181944

C 4.129534329 0.087862433

A 0 0

T 0 0

T 0 0

T 0 0

T 0 0

A 0 0

T 0 0

C 5.591775326 0.112838516

C 5.962093636 0.100414209

C 2.709546721 0.063307166

T 0 0

T 0 0

G 1.701108871 0.138608871

C 1.130085384 0.113008538

A 0 0

A 0 0

A 0 0

T 0 0

T 0 0

G 1.735630738 0.792353163

C 1.267093213 0.200727638

T 0 0

G 1.787287602 0.188797986

G 3.225806452 0.126008065

G 8.254449072 0.580588161

C 6.244514107 0.05015674

T 0 0

G 1.130795326 0.238723458

G 3.955298845 0.803616273

T 0 0

T 0 0

C 0.766042949 0.138138892

T 0 0

T 0 0

G 2.34688755 0.188253012

G 3.280130703 0.326756315

G 10.2638191 0.251256281

T 0 0

T 0 0

C 5.197087622 0.163193573

C 2.233655415 0.778014807

T 0 0

T 0 0

G 1.93540279 0.389594068

G 4.759512746 0.100464649

C 3.653942742 0.08789553

A 0 0

T 0 0

G 4.725992961 0.138260432

G 7.224669604 0.289490245

T 0 0

G 5.053560176 0.075614367

G 8.301886792 0.465408805

G 10.45546049 0.4781077

G 8.554125662 0.264950795

A 0 0

C 4.861546172 0.087708307

T 0 0

C 1.306696821 0.138208318

T 0 0

T 0 0

G 2.781273597 0.641832368

C 4.259050482 0.588751096

C 1.619789051 0.062782521

A 0 0

C 1.865064464 0.362999124

A 0 0

A 0 0

C 10.87202718 0.201333837

C 4.690243291 1.203912716

C 2.951149064 0.037674243

T 0 0

T 0 0

C 1.302278988 0.288004007

T 0 0

G 4.25879397 0.314070352

C 7.165226105 0.275585619

C 1.984924623 0.037688442

T 0 0

C 0.715271678 0.112937633

A 0 0

G 1.521438451 0.238903558

T 0 0

G 6.834487099 0.302076778

G 3.357314149 0.833017796

A 0 0

G 6.264908977 0.364092906

A 0 0

G 3.342128408 0.213594673

T 0 0

A 0 0

T 0 0

C 0.438816449 0.162988967

A 0 0

G 3.11678558 0.388033546

C 2.555430289 0.062633095

T 0 0

T 0 0

T 0 0

T 0 0

G 1.027182763 0.162846048

T 0 0

T 0 0

G 0.996216898 0.138713745

G 3.326638212 0.188300276

C 1.302442079 0.037570445

A 0 0

G 1.793328317 0.438926511

C 1.392198671 0.777624483

A 0 0

A 0 0

C 10.78812179 0.400952262

A 0 0

T 0 0

T 0 0

A 0 0

T 0 0

T 0 0

G 1.294295049 0.025131943

T 0 0

C 0.852878465 0.313558259

T 0 0

T 0 0

T 0 0

G 0.779188136 0.251351012

A 0 0

G 1.006669183 0.188750472

A 0 0

G 2.896551724 0.175548589

G 10.75241804 0.200979776

C 7.721327968 0.062877264

T 0 0

C 1.18987976 0.100200401

T 0 0

G 2.11567351 0.237856785

G 4.791170199 0.188134955

G 6.458909274 0.100527771

A 0 0

A 0 0

G 6.019100276 0.326715255

G 9.233865895 0.012580199

G 11.59657517 0.352556031

C 13.55292934 0.100578325

T 0 0

C 1.727591387 0.13770656

T 0 0

G 1.592077222 0.175504576

G 3.013561025 0.678051231

A 0 0

T 0 0

G 0.777137127 0.476309852

A 0 0

A 0 0

T 0 0

T 0 0

G 2.085427136 0.201005025

C 1.39272271 0.037641154

A 0 0

T 0 0

C 1.02847109 0.188134955

C 0.351273366 0.112909296

G 0.363864492 0.188205772

A 0 0

C 0.890840652 0.112923463

A 0 0

A 0 0

G 4.419889503 0.08789553

C 4.302019315 0.576947197

C 1.557006529 0.062782521

A 0 0

G 2.521640948 0.138000251

G 6.234288587 0.062845651

G 12.51889169 0.113350126

T 0 0

C 2.657973922 0.037612839

C 0.828625235 0.100439422

G 0.565824217 0.238903558

G 3.589807958 0.100414209

T 0 0

T 0 0

G 1.746011808 0.12561236

C 1.354741596 0.188158555

A 0 0

A 0 0

T 0 0

G 3.752039152 0.112937633

C 1.944305068 0.037631711

A 0 0

A 0 0

G 1.604211054 0.062664494

T 0 0

T 0 0

C 0.66391081 0.162846048

T 0 0

A 0 0

T 0 0

A 0 0

G 4.966762825 0.150507964

C 5.438751102 0.050358806

T 0 0

C 5.102168735 0.062680206

C 3.196174657 0.138417013

T 0 0

T 0 0

G 1.871389098 0.037678975

T 0 0

T 0 0

G 1.77349886 0.152014188

G 5.005030181 0.352112676

C 3.188551343 0.062766759

T 0 0

C 1.193317422 0.18841854

T 0 0

C 4.945536497 0.125203456

C 2.418546366 0.313283208

C 1.129801657 0.112980166

G 1.048112135 0.113650713

C 5.653754544 0.087752288

C 1.655379985 0.313518937

T 0 0

G 7.172465771 0.050244944

C 9.04988719 0.075206819

C 5.683101242 0.062727387

C 2.920897581 0.150432493

T 0 0

G 2.032571645 0.429238732

G 2.671707624 0.315059861

A 0 0

A 0 0

A 0 0

C 0.614343029 0.401203611

A 0 0

G 6.522829905 0.363773206

C 6.028324351 0.714375235

C 2.333166081 0.163070748

C 1.268047709 0.652856246

G 2.201811777 0.163563161

G 13.13905288 0.602939329

G 27.38813474 0.075414781

C 15.98288447 0.604077523

C 15.03144654 0.578616352

C 9.916939341 0.100679587

T 0 0

C 2.241422489 0.03756574

A 0 0

T 0 0

G 3.739612188 0.125912868

T 0 0

G 3.029922052 0.666331406

T 0 0

G 3.40366742 0.590303944

T 0 0

G 3.156438632 0.628772636

G 6.4775295 0.803414512

C 2.810186928 0.025090955

T 0 0

G 1.56897201 0.828417221

T 0 0

T 0 0

G 2.771828147 0.201587502

C 1.692789969 0.150470219

T 0 0

C 0.851490108 0.400701227

T 0 0

C 1.628256513 0.162825651

T 0 0

C 4.531797697 0.150225338

C 2.853566959 0.387984981

T 0 0

T 0 0

G 0.643127364 0.189155107

A 0 0

T 0 0

C 0.239385158 0.188988283

G 1.77180196 0.389545112

C 6.51465798 0.889501378

C 5.337676983 0.288184438

C 2.378270121 0.75103267

T 0 0

G 3.193361831 0.138295197

C 2.5785455 0.137689323

T 0 0

T 0 0

A 0 0

T 0 0

T 0 0

G 2.065231079 0.528900642

G 4.116982553 0.100414209

C 2.588264857 0.20103028

A 0 0

T 0 0

C 0.738977956 0.237975952

T 0 0

G 1.355081556 0.163111669

T 0 0

G 1.632755589 0.301431801

G 4.832433789 0.364001506

C 3.82685069 0.188205772

A 0 0

T 0 0

G 1.019637462 0.51611279

A 0 0

A 0 0

G 3.110107851 0.15048909

C 2.193807196 0.488905604

A 0 0

G 1.984924623 0.603015075

G 9.532780708 0.489826677

T 0 0

C 3.396415591 0.11279609

C 2.279273638 0.100187852

A 0 0

G 2.95517155 0.15026296

T 0 0

G 3.261005895 0.526777875

C 1.963499056 0.151038389

A 0 0

C 1.727591387 0.150225338

A 0 0

G 2.482136141 0.576657891

G 9.616593338 0.452545569

C 7.027027027 0.51539912

T 0 0

C 0.989850896 0.501190327

T 0 0

A 0 0

A 0 0

C 0.389007404 0.250972519

G 0.466876972 0.113564669

A 0 0

G 1.307025261 0.289053663

A 0 0

G 7.530120482 0.828313253

G 13.88435971 0.200677286

G 13.15195587 0.313440321

C 14.68848332 0.742605412

C 5.758374106 0.890728892

A 0 0

A 0 0

A 0 0

G 5.221997982 0.239656912

C 9.700176367 0.264550265

A 0 0

T 0 0

A 0 0

C 9.136483269 0.025065798

C 1.835785238 0.264051301

T 0 0

T 0 0

C 0.714733542 0.10031348

T 0 0

G 1.531508913 0.615114236

G 3.977415307 0.476787955

G 4.602457988 0.464008026

A 0 0

A 0 0

C 1.492537313 0.326100589

T 0 0

T 0 0

C 0.689568706 0.162988967

A 0 0

G 1.718730398 0.47672814

G 4.79056935 0.075244545

A 0 0

G 5.195131133 0.288618396

T 0 0

C 5.437233776 0.087697319

C 2.760697704 0.313715648

T 0 0

C 1.514772158 0.262894342

T 0 0

T 0 0

C 0.828105395 0.012547051

A 0 0

T 0 0

C 3.092138207 0.112669004

C 1.051445738 0.312930279

T 0 0

G 0.852878465 0.326100589

A 0 0

C 0.816377795 0.037678975

G 0 0

G 3.312421581 0.326223338

G 7.304216867 0.263554217

C 5.170033881 0.100389007

A 0 0

T 0 0

C 0.589267803 0.351053159

T 0 0

T 0 0

C 0.350789276 0.187922826

G 0.388763481 0.188111362

T 0 0

T 0 0

C 0.388033546 0.1126549

T 0 0

G 0.439588043 0.251193168

A 0 0

T 0 0

T 0 0

C 0.939849624 0.062656642

C 0.389007404 0.250972519

G 0.338897954 0.301242626

G 2.817964524 0.037740596

T 0 0

G 0.655737705 0.239596469

A 0 0

G 4.252916823 0.100363819

C 3.434875266 0.175504576

T 0 0

G 1.654964895 0.100300903

G 2.859651323 0.050169321

A 0 0

C 1.581126867 0.112937633

A 0 0

G 3.047786279 0.175592625

C 2.964079377 0.414468726

C 1.429108687 0.175504576

A 0 0

A 0 0

T 0 0

A 0 0

T 0 0

A 0 0

A 0 0

T 0 0

C 0.489335006 0.025094103

A 0 0

T 0 0

C 0.663412192 0.075103267

A 0 0

G 0.414364641 0.26368659

A 0 0

G 0.200476131 0.501190327

A 0 0

T 0 0

T 0 0

T 0 0

C 0.525657071 0.525657071

T 0 0

A 0 0

C 1.879228264 0.037584565

A 0 0

A 0 0

C 7.751160166 0.200677286

C 2.338740098 0.213755815

C 1.116407426 0.539387858

A 0 0

G 4.064734663 0.18818216

C 3.456480902 0.275516594

C 2.021851061 0.037674243

A 0 0

T 0 0

C 3.155917345 0.626174076

C 2.321204517 0.501882058

A 0 0

C 5.339014914 0.526381752

A 0 0

T 0 0

A 0 0

G 1.497081959 1.192590713

G 7.569215138 0.508001016

T 0 0

C 0.849822425 0.03805175

A 0 0

G 0.303528519 1.214114076

A 0 0

A 0 0

A 0 0

C 0.303912878 0.012663037

G 0.342378899 0.342378899

A 0 0

G 0.445576066 0.420114577

A 0 0

G 3.393893515 0.492051476

C 3.010833963 0.062988158

T 0 0

G 1.147107021 0.289928148

G 4.198184569 0.340393343

G 5.770930673 1.250157848

A 0 0

G 2.114271331 0.151019381

C 1.749748238 0.944108761

A 0 0

G 2.121948102 0.166176659

C 1.549692623 0.358606557

A 0 0

C 0.93326515 0.063922271

T 0 0

T 0 0

T 0 0

T 0 0

C 3.788069469 0.113264536

C 1.817042607 0.012531328

T 0 0

T 0 0

G 1.332662811 0.138295197

G 3.750786658 0.339836375

C 2.932662052 0.226557583

T 0 0

G 2.255064339 0.293030959

G 4.179982104 0.242873578

G 7.607722798 0.242935686

C 4.70090944 0.115281158

A 0 0

A 0 0

G 2.153171738 0.06446622

C 0.559095046 0.364061891

G 0.744319666 0.287281274

C 0.889586604 0.104657248

T 0 0

G 1.498225785 0.27598896

C 1.906389692 0.05259006

T 0 0

G 3.40684009 0.079228839

T 0 0

C 5.293181215 0.145927302

C 2.410596026 0.17218543

T 0 0

C 1.546666667 0.08

T 0 0

T 0 0

C 1.259884734 0.013403029

A 0 0

T 0 0

T 0 0

G 2.950730915 0.839198701

G 5.075315511 0.312118334

A 0 0

G 8.05137412 0.428117663

G 13.85022272 0.389755011

G 11.79775281 0.842696629

G 10.01839536 0.679213245

G 13.60602187 0.113620224

T 0 0

C 8.350217077 0.04341534

T 0 0

G 2.156212304 0.693606755

C 2.708427901 0.196701468

T 0 0

T 0 0

T 0 0

G 0 0

T 0 0

G 0 0

G 0.879049324 3.288295621

A 0 0

T 0 0

T 0 0

T 0 0

T 0 0

G 1.440536013 0.887772194

C 0.856423174 0.470193115

T 0 0

G 1.724137931 1.910074375

C 1.458368662 2.102764117

T 0 0

G 0.992131372 0.872391379

C 0.78915766 1.57831532

A 0 0

A 0 0

C 1.110532709 0.24292903

A 0 0

G 0.438212095 0.227870289

A 0 0

A 0 0

A 0 0

G 0.667629015 0.523276795

A 0 0

A 0 0

G 2.361911847 0.092988655

C 2.942830366 0.056232427

A 0 0

A 0 0

G 4.217325228 0.227963526

G 7.31055545 0.286314182

G 15.78137028 0.173210162

T 0 0

A 0 0

C 11.75318315 0.117531832

A 0 0

G 0.605798356 2.87754219

A 0 0

T 0 0

A 0 0

T 0 0

C 3.742314889 2.058273189

C 5.263157895 0.026716538

A 0 0

G 0 0

T 0 0

G 0 0

C 10.51068884 1.454869359

C 4.375377188 2.534701267

T 0 0

G 0 0

G 5.229387231 6.641000962

C 7.210750574 2.523762701

T 0 0

A 0 0

C 9.268465909 0.745738636

C 4.129032258 1.419354839

G 0 0

T 0 0

G 2.685851319 8.872901679

T 0 0

G 3.864229765 14.09921671

C 13.09836928 0.999473961

C 9.180868609 4.507971413

A 0 0

C 15.85081585 0.466200466

A 0 0

C 7.45301361 4.795852236

A 0 0

C 2.873563218 0.359195402

A 0 0

G 0 0

A PRIMER NA NA

T PRIMER NA NA

A PRIMER NA NA

A PRIMER NA NA

G PRIMER NA NA

C PRIMER NA NA

G PRIMER NA NA

A PRIMER NA NA

A PRIMER NA NA

G PRIMER NA NA

A PRIMER NA NA

A PRIMER NA NA

A PRIMER NA NA

T PRIMER NA NA

A PRIMER NA NA

C PRIMER NA NA

G PRIMER NA NA

A PRIMER NA NA

C PRIMER NA NA

A PRIMER NA NA

A PRIMER NA NA

T PRIMER NA NA

G PRIMER NA NA

C PRIMER NA NA

T PRIMER NA NA

T PRIMER NA NA

A NA NA

G NA NA

T NA NA

A NA NA

A NA NA

G NA NA

A NA NA

C NA NA

C NA NA

T NA NA

C NA NA

C NA NA

A NA NA

C NA NA

C NA NA

A NA NA

G NA NA

T NA NA

T NA NA

A NA NA

T NA NA

G NA NA

T NA NA

C NA NA

T NA NA

A NA NA

A NA NA

G NA NA

G NA NA

T NA NA

C NA NA

A NA NA

A NA NA

C NA NA

A NA NA

T NA NA

T NA NA

A NA NA

T NA NA

T NA NA

A NA NA

T NA NA

C NA NA

A NA NA

A NA NA

A NA NA

T NA NA

T NA NA

T PRIMER NA NA

G PRIMER NA NA

T PRIMER NA NA

G PRIMER NA NA

A PRIMER NA NA

T PRIMER NA NA

G PRIMER NA NA

A PRIMER NA NA

A PRIMER NA NA

A PRIMER NA NA

A PRIMER NA NA

A PRIMER NA NA

T PRIMER NA NA

A PRIMER NA NA

T PRIMER NA NA

C PRIMER NA NA

C PRIMER NA NA

T PRIMER NA NA

T PRIMER NA NA

T PRIMER NA NA

C PRIMER NA NA

T PRIMER NA NA

G PRIMER NA NA

C PRIMER NA NA

C PRIMER NA NA

T PRIMER NA NA

A 0 0

T 0 0

C 1.096033403 6.941544885

A 0 0

A 0 0

A 0 0

C 7.331596576 7.88983997

C 2.159827214 15.26277898

T 0 0

C 6.765776699 5.976941748

C 4.598387578 5.165721111

A 0 0

G 11.35893309 0.390894458

C 6.951987834 1.716271997

C 2.696629213 3.942798774

T 0 0

C 7.783882784 2.454212454

C 11.06115108 1.744604317

C 4.651162791 1.196013289

T 0 0

C 2.436287867 0.336592403

A 0 0

C 3.950426026 0.821068939

A 0 0

T 0 0

G 5.765581667 0.145964093

C 4.096593359 0.186862153

T 0 0

T 0 0

A 0 0

G 4.381298992 0.321948488

C 3.882139366 0.809942745

T 0 0

C 0.935350757 0.577716644

A 0 0

T 0 0

T 0 0

T 0 0

T 0 0

A 0 0

C 1.181102362 1.25984252

T 0 0

C 0.721701697 0.873638896

T 0 0

C 0.550206327 0.712767288

A 0 0

A 0 0

A 0 0

T 0 0

G 5.900737592 0.062507813

C 4.551137784 0.037509377

T 0 0

T 0 0

G 4.301613105 0.037514068

G 8.228085532 0.025009379

C 11.75146893 0.450056257

C 5.025 0.4

T 0 0

T 0 0

G 1.362329709 0

T 0 0

A 0 0

A 0 0

A 0 0

T 0 0

G 0.750469043 0

A 0 0

T 0 0

T 0 0

G 1.389062696 0.037542235

C 1.738151807 0.100037514

T 0 0

G 0.337753315 0.025018764

T 0 0

G 0.312734551 0.025018764

A 0 0

T 0 0

T 0 0

C 0.275171982 0.012507817

A 0 0

G 0.375187594 0.012506253

T 0 0

T 0 0

T 0 0

T 0 0

G 0.351097179 0

A 0 0

G 1.000250063 0

T 0 0

G 0.450394095 0.025021894

A 0 0

A 0 0

A 0 0

G 0.437992742 0

A 0 0

A 0 0

T 0 0

T 0 0

G 1.277075247 0.012520346

C 1.92668585 0.012510947

T 0 0

C 0.887832937 0

A 0 0

T 0 0

T 0 0

T 0 0

C 1.451088316 0.037528146

C 0.663412192 0

A 0 0

G 4.828621466 0.037528146

C 7.445876611 0.025028157

C 1.688766575 0

T 0 0

G 0.625782228 0

T 0 0

G 0.237707994 0.025021894

A 0 0

T 0 0

A 0 0

A 0 0

T 0 0

T 0 0

A 0 0

T 0 0

T 0 0

G 1.614518148 0

T 0 0

A 0 0

T 0 0

A 0 0

A 0 0

G 1.664997496 0

G 5.581977472 0.050062578

C 6.937139995 0.012521913

T 0 0

T 0 0

A 0 0

G 0.313087038 0.037570445

A 0 0

T 0 0

T 0 0

G 0.976220275 0.037546934

T 0 0

T 0 0

C 0.825825826 0.05005005

T 0 0

A 0 0

T 0 0

A 0 0

C 1.426247967 0.050043788

A 0 0

A 0 0

A 0 0

T 0 0

G 1.002255074 0.037584565

A 0 0

G 0.550964187 0.062609567

A 0 0

G 1.026796895 0.050087653

C 1.137926723 0.037514068

A 0 0

A 0 0

A 0 0

T 0 0

T 0 0

A 0 0

T 0 0

C 2.21416062 0.12509382

C 1.293807311 0.025122472

A 0 0

T 0 0

C 0.801001252 0.075093867

A 0 0

G 0.889055848 0

T 0 0

G 0.588677355 0.062625251

A 0 0

T 0 0

G 0.638777555 0.0501002

A 0 0

A 0 0

G 0.551033187 0.050093926

A 0 0

T 0 0

A 0 0

A 0 0

T 0 0

G 3.283619501 0.087730292

G 3.695324284 0.113122172

G 8.508495909 0.088105727

T 0 0

A 0 0

G 2.885097842 0.100351229

T 0 0

A 0 0

G 2.244795586 0.313518937

T 0 0

T 0 0

C 5.476190476 0.087719298

C 1.87044941 0.225960331

T 0 0

C 0.050131595 0.150394786

G 0.125565043 0.464590658

A 0 0

T 0 0

C 0.851810096 0.162846048

T 0 0

C 2.180724402 0.32585537

T 0 0

C 7.339679359 0.551102204

C 3.432293624 0.175372667

T 0 0

C 1.817270335 0.313322471

A 0 0

T 0 0

C 0.840863454 0.401606426

T 0 0

G 1.653099562 0.062617408

C 2.58242447 0.313401028

T 0 0

C 6.443137746 0.162642312

C 5.082831325 0.16315261

T 0 0

A 0 0

T 0 0

T 0 0

A 0 0

C 12.23003516 0.100452034

C 5.291139241 2.379746835

C 3.741967998 0.32757969

A 0 0

T 0 0

C 8.752515091 0.125754527

C 12.46829021 0.469304921

C 13.47607053 0.768261965

C 7.399497487 1.193467337

T 0 0

C 2.021343377 0.828625235

A 0 0

C 1.740763932 0.250469631

T 0 0

T 0 0

C 0.872423821 1.378176761

A 0 0

C 0.238304277 1.078640411

G 0 0

C 1.286254729 1.500630517

T 0 0

C 0.928016052 0.351141209

T 0 0

G 1.623662681 2.580239144

C 1.59107993 3.958907542

A 0 0

G 1.191086066 0.512295082

A 0 0

C 2.980382294 0.326961771

A 0 0

T 0 0

G 1.305349373 0.614282058

A 0 0

G 0.744103922 1.336864674

A 0 0

A 0 0

T 0 0

A 0 0

A 0 0

T 0 0

A 0 0

A 0 0

T 0 0

C 0.550826239 0.150225338

T 0 0

T 0 0

C 0.67694622 0.213112699

A 0 0

C 1.026796895 0.200350614

A 0 0

A 0 0

G 0.912894637 0.11411183

A 0 0

C 1.30309485 0.676606941

T 0 0

T 0 0

T 0 0

T 0 0

T 0 0

G 1.065449011 0.304414003

C 0.693918748 0.176633863

T 0 0

G 0.440030173 0.138295197

A 0 0

G 1.145230305 0.667002265

T 0 0

T 0 0

G 0.717884131 0.277078086

A 0 0

A 0 0

G 0.653184273 0.540133149

T 0 0

T 0 0

T 0 0

G 0.488844322 0.200551517

A 0 0

G 1.31876413 0.288872143

G 6.283911672 0.416403785

T 0 0

T 0 0

G 2.176680244 0.534623218

T 0 0

G 3.833987094 0.202454764

C 6.910415881 0.552833271

C 2.692162536 0.578689143

T 0 0

A 0 0

A 0 0

C 2.16018191 0.202122284

T 0 0

A 0 0

C 8.220734941 0.366207855

A 0 0

T 0 0

A 0 0

G 2.894538132 0.289453813

T 0 0

G 1.913814956 0.215462611

A 0 0

C 1.11766922 0.087906568

A 0 0

G 1.766695903 0.187946373

C 1.681726908 0.225903614

A 0 0

A 0 0

T 0 0

T 0 0

G 2.536413862 0.150678051

G 4.60476788 0.125470514

C 6.189700539 0.100238065

C 0.289745528 0.100781053

G 0.13782734 0.100238065

T 0 0

G 1.527099762 0.062586056

T 0 0

A 0 0

A 0 0

T 0 0

T 0 0

T 0 0

T 0 0

T 0 0

T 0 0

C 0.50018757 0.087532825

T 0 0

T 0 0

A 0 0

C 2.865006881 0.137620418

A 0 0

T 0 0

T 0 0

C 1.565043195 0.075122073

C 0.903047786 0.025084661

A 0 0

A 0 0

G 1.64057608 0.062617408

G 4.075746175 0.037622272

A 0 0

A 0 0

A 0 0

G 1.603608118 0.037584565

G 3.912225705 0.062695925

A 0 0

T 0 0

G 0.425425425 0.075075075

A 0 0

T 0 0

T 0 0

A 0 0

T 0 0

T 0 0

A 0 0

A 0 0

T 0 0

T 0 0

T 0 0

C 0.350262697 0.075056292

A 0 0

T 0 0

T 0 0

G 0.778698819 0.087917609

A 0 0

A 0 0

C 0.888721993 0.025034422

A 0 0

A 0 0

C 8.533533534 0.1001001

A 0 0

T 0 0

T 0 0

G 0.942092702 0.06280618

T 0 0

C 0.375751503 0.062625251

A 0 0

A 0 0

A 0 0

T 0 0

T 0 0

A 0 0

A 0 0

A 0 0

A 0 0

T 0 0

T 0 0

G 1.302931596 0.125281884

T 0 0

G 2.769076557 0.112767824

T 0 0

A 0 0

G 0.865637938 0.087818341

A 0 0

G 1.414799048 0.062601728

T 0 0

T 0 0

T 0 0

C 0.313047834 0.03756574

A 0 0

T 0 0

C 0.551102204 0.0501002

A 0 0

A 0 0

G 7.235109718 0.062695925

C 7.616184392 0.125266191

T 0 0

T 0 0

A 0 0

T 0 0

G 6.615712317 0.037589275

T 0 0

G 1.395875252 0.025150905

A 0 0

C 1.602203029 0.062586056

T 0 0

G 1.040100251 0.037593985

T 0 0

C 0.751879699 0.012531328

A 0 0

G 5.704613842 0.17552658

T 0 0

A 0 0

C 7.806039343 0.050119033

A 0 0

T 0 0

G 4.162487462 0.188064193

T 0 0

T 0 0

T 0 0

C 0.789869609 0.075225677

T 0 0

T 0 0

T 0 0

A 0 0

C 1.17661785 0.100137689

T 0 0

T 0 0

T 0 0

T 0 0

G 0.945060484 0.541834677

C 0.715002509 0.589563472

A 0 0

A 0 0

C 15.06076933 0.238065405

A 0 0

T 0 0

A 0 0

C 2.747459541 0.138000251

A 0 0

G 1.918465228 0.252429635

T 0 0

G 0.691823899 0.805031447

A 0 0

A 0 0

T 0 0

G 3.662360467 0.087796313

C 1.456188802 0.150640221

A 0 0

G 0.930700541 0.955854609

A 0 0

T 0 0

A 0 0

A 0 0

A 0 0

G 1.020022667 0.755572346

A 0 0

G 1.157960982 0.201384519

T 0 0

C 0.551516671 0.150413638

A 0 0

A 0 0

A 0 0

G 1.822982147 0.238873523

T 0 0

G 2.821316614 0.463949843

C 2.685405948 0.100389007

T 0 0

T 0 0

G 2.864681493 0.263852243

C 2.78824416 0.175835217

A 0 0

T 0 0

G 2.657973922 0.26328987

T 0 0

G 1.181795323 0.213728941

A 0 0

C 1.754825771 0.100275758

A 0 0

A 0 0

T 0 0

A 0 0

A 0 0

A 0 0

A 0 0

T 0 0

G 3.312421581 0.35131744

C 2.472389558 0.012550201

A 0 0

T 0 0

G 1.155633714 0.401959553

A 0 0

G 0.869346101 0.088194532

A 0 0

G 2.29305783 0.239385158

T 0 0

A 0 0

T 0 0

C 0.940674777 0.213219616

A 0 0

A 0 0

G 2.884735984 0.112880973

T 0 0

A 0 0

G 5.291603821 0.56561086

C 5.162019593 0.313991459

T 0 0

T 0 0

G 4.498183185 0.213005889

C 8.220381526 0.050200803

C 4.203372766 0.478228039

T 0 0

A 0 0

C 11.96021654 0.100717613

C 3.82278481 0.683544304

T 0 0

C 1.740110165 0.175262894

T 0 0

G 1.820236003 0.715541049

T 0 0

G 3.402646503 0.919974795

T 0 0

G 4.646746012 1.240820461

G 6.808457085 1.107475459

C 5.089218397 0.075395828

A 0 0

T 0 0

G 4.484476811 1.149865849

G 5.044659706 1.434142659

T 0 0

G 1.962025316 1.278481013

T 0 0

A 0 0

T 0 0

T 0 0

T 0 0

T 0 0

C 0.250783699 0.413793103

A 0 0

G 0.601880878 0.401253918

A 0 0

G 0.690001255 0.564546481

A 0 0

A 0 0

T 0 0

A 0 0

G 1.955867603 0.300902708

C 1.468005019 0.376411543

A 0 0

G 1.092415871 0.050226017

A 0 0

G 0.992088409 0.414416677

A 0 0

A 0 0

G 1.725012684 0.253678336

T 0 0

G 2.13680617 0.139082058

T 0 0

A 0 0

T 0 0

C 0.163748583 0.176344628

G 0.125533517 0.364047201

A 0 0

T 0 0

T 0 0

T 0 0

T 0 0

T 0 0

C 0.339025615 0.08789553

A 0 0

C 0.225281602 0.050062578

G 0.691562932 0.062869357

T 0 0

T 0 0

G 1.167754897 0.414364641

G 3.288565332 0.564829923

C 1.88253012 0.050200803

A 0 0

A 0 0

G 2.231186184 0.012605572

A 0 0

C 6.642436396 0.025065798

C 1.621214025 0.138243056

T 0 0

T 0 0

T 0 0

A 0 0

T 0 0

G 1.58013544 0.288437422

A 0 0

G 0.541629928 0.125960448

A 0 0

A 0 0

A 0 0

T 0 0

G 6.217940698 0.175153259

T 0 0

T 0 0

A 0 0

T 0 0

T 0 0

C 0.300638858 0.087686333

A 0 0

C 0.7508447 0.100112627

A 0 0

A 0 0

A 0 0

C 1.402805611 0.200400802

T 0 0

C 0.262894342 0.062593891

T 0 0

G 0.187969925 0.07518797

A 0 0

A 0 0

A 0 0

T 0 0

C 0.688360451 0.050062578

A 0 0

T 0 0

T 0 0

T 0 0

T 0 0

A 0 0

A 0 0

T 0 0

A 0 0

T 0 0

A 0 0

A 0 0

G 1.382430564 0.025135101

A 0 0

C 1.326658323 0.050062578

T 0 0

T 0 0

C 0.288220551 0.112781955

A 0 0

G 0.338345865 0.087719298

A 0 0

A 0 0

A 0 0

T 0 0

A 0 0

A 0 0

T 0 0

C 0.362953692 0.087609512

T 0 0

G 2.078117176 0.062593891

C 2.240861292 0.025037556

T 0 0

T 0 0

G 2.893286573 0.0501002

T 0 0

A 0 0

G 2.158363659 0.050194504

T 0 0

C 1.019765832 0.07553821

A 0 0

C 1.289918597 0.037570445

A 0 0

A 0 0

T 0 0

G 4.499874655 0.388568564

T 0 0

A 0 0

A 0 0

G 4.575655008 0.200576658

C 4.331450094 0.050219711

T 0 0

G 0.4411394 0.201663726

A 0 0

A 0 0

G 0.97977641 0.050244944

T 0 0

T 0 0

T 0 0

C 0.639418255 0.025075226

T 0 0

T 0 0

T 0 0

T 0 0

A 0 0

A 0 0

T 0 0

A 0 0

A 0 0

T 0 0

T 0 0

A 0 0

A 0 0

T 0 0

C 0.513334168 0.33804933

T 0 0

A 0 0

A 0 0

G 2.610340479 0.100882724

G 6.71547589 0.1009846

G 4.831162261 0.177058303

A 0 0

T 0 0

C 4.22411632 0.250689396

T 0 0

A 0 0

C 2.530377051 0.338218715

T 0 0

C 0.778796634 0.288908429

A 0 0

A 0 0

C 4.810824355 0.150338261

A 0 0

T 0 0

T 0 0

T 0 0

T 0 0

C 4.480984059 0.138069537

C 6.778169014 0.100603622

C 2.754716981 0.327044025

T 0 0

C 1.477585775 0.288004007

T 0 0

A 0 0

T 0 0

T 0 0

T 0 0

A 0 0

G 1.975091206 0.10064159

C 1.329487019 0.075253982

A 0 0

C 5.996738176 0.138000251

C 0.998735777 0.594184576

T 0 0

G 0.427726758 0.062900994

A 0 0

A 0 0

A 0 0

A 0 0

A 0 0

T 0 0

G 2.173093832 0.075367416

G 3.784533872 0.378453387

C 2.1456519 0.113593336

A 0 0

G 1.415929204 0.139064475

A 0 0

C 2.168191503 0.76450683

T 0 0

C 0.601805416 0.125376128

T 0 0

G 1.278035334 0.250595164

G 2.176100629 0.188679245

A 0 0

A 0 0

A 0 0

A 0 0

T 0 0

G 2.898185484 0.126008065

G 3.023431595 0.491307634

A 0 0

T 0 0

A 0 0

A 0 0

G 1.345742674 0.037731103

A 0 0

C 1.001376893 0.075103267

T 0 0

G 0.302152839 0.088127911

A 0 0

A 0 0

A 0 0

A 0 0

A 0 0

T 0 0

C 0.676522175 0.087697319

A 0 0

A 0 0

T 0 0

T 0 0

C 0.552139541 0.150583511

A 0 0

C 1.364888555 0.062609567

T 0 0

T 0 0

A 0 0

T 0 0

C 0.700788387 0.087598548

T 0 0

G 2.042862514 0.187993483

C 1.658083155 0.150734832

T 0 0

A 0 0

C 0.251667296 0.151000378

G 0.328407225 0.328407225

C 0.805842357 0.214051876

T 0 0

C 0.463426854 0.087675351

T 0 0

G 0.338897954 0.35144973

A 0 0

A 0 0

A 0 0

A 0 0

T 0 0

T 0 0

T 0 0

G 1.241223671 0.238214644

T 0 0

T 0 0

G 1.459670316 0.100666918

T 0 0

G 2.20959596 0.126262626

T 0 0

G 2.669842066 0.137879168

C 2.546093064 0.238304277

A 0 0

C 1.92042174 0.012551776

A 0 0

G 3.752039152 0.27606977

C 3.374889812 0.277043194

T 0 0

A 0 0

T 0 0

T 0 0

A 0 0

T 0 0

A 0 0

C 8.351231838 0.31585597

A 0 0

T 0 0

A 0 0

A 0 0

T 0 0

T 0 0

A 0 0

T 0 0

A 0 0

A 0 0

T 0 0

T 0 0

T 0 0

C 0.267379679 0.343773873

A 0 0

A 0 0

A 0 0

C 1.239669421 0.175306787

T 0 0

A 0 0

T 0 0

A 0 0

G 5.165496489 0.188064193

C 4.823956898 0.225535647

T 0 0

T 0 0

C 0.844573301 0.340350435

A 0 0

A 0 0

G 5.100755668 0.16372796

C 4.283030683 0.288040075

T 0 0

A 0 0

T 0 0

T 0 0

A 0 0

A 0 0

T 0 0

G 11.38812328 0.137810073

T 0 0

T 0 0

T 0 0

C 0.613881233 0.075169131

T 0 0

C 0.926505572 0.187805183

T 0 0

A 0 0

T 0 0

T 0 0

C 0.463949843 0.10031348

T 0 0

T 0 0

T 0 0

T 0 0

T 0 0

T 0 0

G 1.459854015 0.176189278

C 1.353722738 0.112810228

T 0 0

T 0 0

T 0 0

G 0.627904056 0.138138892

T 0 0

T 0 0

T 0 0

G 2.727501257 0.075414781

T 0 0

A 0 0

G 2.162162162 0.062853551

T 0 0

G 2.025060119 0.075939754

A 0 0

C 7.932330827 0.100250627

C 1.935889378 0.113136392

T 0 0

T 0 0

G 2.01830262 0.188040617

T 0 0

G 1.931276649 0.388763481

T 0 0

C 1.615933859 0.037579857

T 0 0

T 0 0

A 0 0

T 0 0

T 0 0

C 0.325569747 0.07513148

T 0 0

A 0 0

A 0 0

G 1.399042097 0.17645576

G 2.502829833 0.138347378

A 0 0

T 0 0

T 0 0

A 0 0

A 0 0

A 0 0

A 0 0

A 0 0

T 0 0

T 0 0

G 1.175881911 0.100075056

T 0 0

T 0 0

A 0 0

A 0 0

A 0 0

T 0 0

T 0 0

G 1.242625832 0.112965985

G 1.997989445 0.150791656

A 0 0

T 0 0

A 0 0

C 1.138923655 0.025031289

A 0 0

G 0.703164239 0.125565043

A 0 0

A 0 0

T 0 0

G 1.19107322 0.075225677

A 0 0

A 0 0

C 0.813822461 0.050081382

A 0 0

A 0 0

A 0 0

A 0 0

C 9.290371113 0.112838516

A 0 0

T 0 0

A 0 0

C 0.238184781 0.100288329

G 0.441473259 0.15136226

A 0 0

A 0 0

G 2.69761606 0.075282309

T 0 0

A 0 0

G 3.496240602 0.137844612

G 4.788167461 0.037603409

A 0 0

C 2.541311968 0.062593891

A 0 0

G 1.992980697 0.125344698

C 1.74573055 0.215053763

A 0 0

G 4.042179262 0.175746924

T 0 0

G 3.588006524 0.401455275

C 3.265101093 0.025116162

T 0 0

T 0 0

G 2.080721985 0.313361745

T 0 0

G 1.884185404 0.175857304

A 0 0

C 1.077424204 0.16286645

T 0 0

T 0 0

A 0 0

C 1.840951785 0.375704446

T 0 0

A 0 0

T 0 0

T 0 0

T 0 0

T 0 0

A 0 0

T 0 0

T 0 0

T 0 0

A 0 0

T 0 0

T 0 0

T 0 0

C 2.201257862 0.100628931

C 2.637197036 0.087906568

A 0 0

T 0 0

T 0 0

G 1.834631817 0.263885398

T 0 0

T 0 0

A 0 0

A 0 0

T 0 0

C 0.588529927 0.025043827

A 0 0

A 0 0

T 0 0

T 0 0

G 0.818846057 0.100781053

A 0 0

T 0 0

G 0.815967863 0.225960331

A 0 0

A 0 0

C 5.49905838 0.11299435

C 1.424788803 0.201740008

T 0 0

A 0 0

G 1.393596987 0.188323917

A 0 0

A 0 0

G 1.293644813 0.062798292

A 0 0

G 0.657977983 0.341642414

A 0 0

A 0 0

A 0 0

T 0 0

G 1.066098081 0.062711652

A 0 0

T 0 0

T 0 0

A 0 0

T 0 0

A 0 0

T 0 0

C 4.082654978 0.100187852

C 1.88253012 0.288654618

T 0 0

T 0 0

T 0 0

A 0 0

T 0 0

A 0 0

A 0 0

T 0 0

G 6.630666834 0.251161623

T 0 0

C 1.144654088 0.075471698

A 0 0

T 0 0

C 0.89162376 0.326510109

A 0 0

A 0 0

T 0 0

G 0.93081761 0.050314465

A 0 0

A 0 0

G 1.85976376 0.062829857

T 0 0

A 0 0

T 0 0

T 0 0

G 0.893306492 0.138399597

A 0 0

T 0 0

T 0 0

G 0.966972247 0.075348487

A 0 0

T 0 0

T 0 0

G 0.690434346 0.175746924

A 0 0

T 0 0

T 0 0

A 0 0

A 0 0

A 0 0

A 0 0

A 0 0

T 0 0

A 0 0

A 0 0

C 0.789275871 0.175394638

A 0 0

A 0 0

A 0 0

T 0 0

T 0 0

C 0.427189345 0.05025757

T 0 0

T 0 0

G 0.914328657 0.062625251

T 0 0

T 0 0

C 0.564475665 0.075263422

A 0 0

C 1.10330993 0.238214644

A 0 0

C 1.503382611 0.062640942

T 0 0

T 0 0

G 1.545420279 0.037693178

A 0 0

A 0 0

G 2.479234835 0.062924742

G 6.445904954 0.088473205

A 0 0

A 0 0

G 4.131396083 0.176879343

G 7.622527403 0.037797657

C 6.534126884 0.088641256

A 0 0

T 0 0

C 1.116127414 0.426385754

T 0 0

C 1.041797414 0.200828417

A 0 0

T 0 0

C 0.790166813 0.589489527

A 0 0

C 3.741241241 0.675675676

A 0 0

T 0 0

T 0 0

T 0 0

A 0 0

A 0 0

A 0 0

T 0 0

A 0 0

T 0 0

C 3.533834586 0.187969925

C 1.392547986 0.301091457

T 0 0

C 0.814332248 0.037584565

A 0 0

G 1.972361809 0.037688442

T 0 0

G 1.842567059 0.150413638

T 0 0

G 0.917431193 0.238783461

A 0 0

G 1.566808724 0.087741289

T 0 0

T 0 0

G 1.888693024 0.125912868

G 5.452713764 0.176300214

T 0 0

A 0 0

G 1.63029847 0.200652119

T 0 0

T 0 0

C 0.250815149 0.037622272

A 0 0

G 1.21706399 0.150564617

A 0 0

G 1.67982951 0.075216247

A 0 0

C 2.331703648 0.100288329

T 0 0

C 0.588456241 0.150244147

T 0 0

G 0.713481036 0.175240956

T 0 0

T 0 0

T 0 0

T 0 0

A 0 0

G 0.826446281 0.125219133

T 0 0

T 0 0

G 0.617828773 0.063043752

A 0 0

A 0 0

T 0 0

C 0.563274502 0.075103267

T 0 0

C 0.626802056 0.062680206

A 0 0

A 0 0

C 0.853627919 0.100426814

T 0 0

T 0 0

A 0 0

G 1.331491019 0.06280618

T 0 0

C 0.727820304 0.138034885

A 0 0

A 0 0

A 0 0

T 0 0

A 0 0

T 0 0

A 0 0

A 0 0

A 0 0

C 1.038928527 0.175240956

T 0 0

G 1.82911551 0.062640942

C 1.616541353 0.087719298

A 0 0

T 0 0

G 2.483381412 0.263388938

A 0 0

C 6.203692076 0.11302273

C 1.647384306 0.213782696

T 0 0

T 0 0

G 1.145374449 0.151038389

G 2.215787486 0.377691049

C 0.175746924 0.564900829

G 0.17721519 0.139240506

A 0 0

G 4.594526739 0.100426814

C 4.074724173 0.639418255

C 2.018808777 0.150470219

A 0 0

C 11.08605787 0.563697858

C 4.772670183 0.439588043

C 3.223378904 0.225761947

A 0 0

G 6.153459751 0.138138892

G 10.10369246 0.227617602

G 16.86397985 0.138539043

T 0 0

C 10.83688299 0.16286645

C 10.29060259 0.251603975

C 3.571428571 0.050301811

T 0 0

G 0.840757937 0.690174426

A 0 0

A 0 0

C 1.217522279 0.251035522

T 0 0

T 0 0

C 0.23803558 0.588824856

A 0 0

G 3.573667712 1.31661442

C 6.290726817 0.526315789

T 0 0

C 2.629272568 0.56341555

C 4.249717939 0.188040617

A 0 0

G 2.018049637 0.100275758

T 0 0

A 0 0

T 0 0

G 5.326482015 0.238125078

T 0 0

G 3.128926866 0.251319427

A 0 0

C 3.301117108 0.06275888

A 0 0

T 0 0

G 1.382256848 0.628298567

A 0 0

T 0 0

A 0 0

C 8.432527252 0.526249843

C 2.592639433 0.177058303

T 0 0

A 0 0

C 4.765487835 0.250815149

T 0 0

C 2.573760201 0.376647834

C 4.067497796 0.125928724

A 0 0

C 1.064229373 0.025040691

A 0 0

G 0.80351538 0.477087257

A 0 0

G 1.544450025 0.489703666

T 0 0

T 0 0

G 2.274440814 0.703694396

T 0 0

T 0 0

G 2.553830746 0.025037556

T 0 0

A 0 0

A 0 0

G 0.764698508 0.952739125

A 0 0

A 0 0

C 1.481667504 0.062782521

T 0 0

C 0.551033187 0.075140889

A 0 0

G 1.329320291 0.363681966

T 0 0

G 1.003512293 0.526843954

A 0 0

G 0.889501378 0.250563768

A 0 0

T 0 0

G 0.85609971 0.113307315

A 0 0

G 1.657250471 0.263653484

T 0 0

T 0 0

G 3.581715717 0.037570445

T 0 0

A 0 0

C 3.042824944 0.22539444

A 0 0

A 0 0

A 0 0

C 7.326236694 0.100187852

C 1.078775715 0.13798294

T 0 0

T 0 0

T 0 0

A 0 0

T 0 0

T 0 0

T 0 0

T 0 0

T 0 0

T 0 0

T 0 0

A 0 0

A 0 0

A 0 0

T 0 0

T 0 0

T 0 0

T 0 0

A 0 0

T 0 0

T 0 0

T 0 0

A 0 0

T 0 0

T 0 0

T 0 0

T 0 0

T 0 0

T 0 0

T 0 0

G 0.358285349 0.179142674

A 0 0

G 0.519513431 0.152052712

A 0 0

C 0.527770797 0.037697914

G 0 0

G 1.046394352 0.252143217

A 0 0

G 2.788594398 0.113051124

T 0 0

C 1.392897478 0.464299159

T 0 0

C 1.628460479 0.613804334

T 0 0

C 1.101790409 0.626017278

T 0 0

C 0.989231155 0.525920361

T 0 0

G 2.03313253 0.087851406

T 0 0

C 1.858129316 0.351537979

A 0 0

C 10.44776119 0.313558259

C 4.299182904 0.52796983

C 2.024393311 0.276625173

A 0 0

G 5.278371245 0.301621214

C 6.752008032 2.484939759

C 1.745354093 0.037669513

T 0 0

G 2.485563645 0.602560884

G 2.94413689 0.201308505

A 0 0

G 3.28650276 0.125439037

T 0 0

G 2.331442974 0.365469439

C 1.792204537 0.087730292

A 0 0

G 2.06948451 0.426439232

T 0 0

G 2.287375205 0.404397826

G 5.173716012 0.86858006

C 4.236861956 0.088006035

T 0 0

C 0.866725286 0.715990453

G 0 0

A 0 0

T 0 0

C 0.376222724 0.163029847

T 0 0

T 0 0

G 2.499685969 0.464765733

G 4.398642705 0.314188765

C 4.247055876 0.187922826

T 0 0

C 1.065697091 0.08776329

A 0 0

C 1.501313649 0.250218942

T 0 0

G 2.352793156 0.150981379

C 1.679408447 0.32585537

A 0 0

A 0 0

G 7.117750439 1.343208637

C 6.475090978 0.125486259

T 0 0

C 1.091045899 0.163029847

C 0.92101943 0.227100681

G 0 0

C 11.9077463 0.188017047

C 8.416959358 0.100351229

C 7.82837787 0.150545728

C 3.981469889 0.626017278

C 0.991963837 0.163234555

G 0 0

G 1.916288452 0.214321735

G 9.505175461 0.2524615

T 0 0

T 0 0

C 1.228070175 0.388471178

A 0 0

T 0 0

G 4.963558683 0.113093742

C 4.009522616 0.162886856

C 2.24817885 0.866616428

A 0 0

T 0 0

T 0 0

C 0.789374765 0.125297582

T 0 0

T 0 0

C 0.965517241 0.363636364

T 0 0

G 3.437460795 0.200727638

C 6.784328452 0.175240956

C 1.992481203 0.087719298

T 0 0

T 0 0

A 0 0

G 6.483571608 0.125407575

C 7.405087082 0.150357098

C 2.522273811 0.27606977

T 0 0

C 5.961177207 0.125234815

C 2.632898696 0.288365095

C 1.458385718 0.440030173

G 0 0

A 0 0

G 2.166519713 0.088172314

T 0 0

A 0 0

G 3.962382445 0.413793103

C 3.649818136 0.388812241

T 0 0

G 2.316844083 0.300563557

G 5.123363545 0.289526687

G 4.968009033 0.639819345

A 0 0

C 5.787298008 0.338218715

T 0 0

A 0 0

C 3.886792453 0.125786164

A 0 0

G 2.106847254 0.564334086

G 5.772367926 0.363910152

C 1.756381097 0.657063432

G 0 0

C 7.753006012 0.263026052

C 4.161443971 0.827275006

C 4.141046555 0.200778015

A 0 0

C 5.911823647 0.313126253

C 2.536732387 0.364184353

A 0 0

C 7.854190154 0.463484905

C 2.254124166 0.705200856

T 0 0

C 1.797837566 0.502891627

G 0 0

C 4.535204209 0.63893761

C 1.192568416 0.07532011

T 0 0

G 0 0

G 3.641633065 0.378024194

C 3.045112782 0.175438596

T 0 0

A 0 0

A 0 0

T 0 0

T 0 0

T 0 0

T 0 0

T 0 0

T 0 0

G 0 0

T 0 0

A 0 0

T 0 0

T 0 0

T 0 0

T 0 0

T 0 0

A 0 0

G 1.969392875 0.275965881

T 0 0

A 0 0

G 1.342702974 0.188229389

A 0 0

G 1.002757583 0.300827275

A 0 0

C 2.040816327 0.187805183

G 0 0

G 2.858934169 0.388714734

G 4.044209997 0.200954534

G 8.542713568 0.301507538

T 0 0

T 0 0

T 0 0

C 0.739811912 0.163009404

A 0 0

C 5.273046092 0.162825651

C 4.83219783 0.227100681

A 0 0

T 0 0

G 3.710203058 1.002757583

T 0 0

T 0 0

A 0 0

G 4.064224787 0.426492725

C 3.846153846 0.452488688

C 1.582914573 0.150753769

A 0 0

G 1.909787662 0.464882523

G 3.789977336 0.314782171

A 0 0

T 0 0

G 1.030539148 0.527837125

A 0 0

T 0 0

C 0.640462137 0.690694462

T 0 0

C 1.535556954 0.805538074

G 0 0

A 0 0

T 0 0

C 1.192418727 0.200828417

T 0 0

C 4.530053959 0.213326641

C 2.458296752 0.288473598

T 0 0

G 2.020582329 0.175702811

A 0 0

C 7.008525577 0.363590772

C 1.750602562 0.215653939

T 0 0

C 1.293644813 0.577744285

G 0 0

T 0 0

G 0.529767911 0.441473259

A 0 0

T 0 0

C 0.952978056 0.689655172

T 0 0

G 4.477050414 0.313518937

C 7.252004008 0.338176353

C 2.494046873 0.513848853

T 0 0

G 5.061388123 0.300676522

C 8.054196462 0.42654623

C 2.643447757 0.300676522

T 0 0

T 0 0

T 0 0

G 4.57114153 0.138138892

C 6.906492855 0.764602657

C 2.922362975 0.238304277

T 0 0

C 6.147283904 0.313636934

C 4.003514056 0.903614458

C 1.793778224 0.928248871

A 0 0

A 0 0

A 0 0

G 1.406328478 0.364138624

T 0 0

G 1.715072609 0.338007011

C 2.178540128 0.751220734

T 0 0

A 0 0

G 2.105527008 0.413585662

G 2.910915935 0.376411543

A 0 0

T 0 0

T 0 0

A 0 0

C 2.258185924 0.489273617

A 0 0

G 1.205575788 1.054878815

G 5.88382888 0.439091707

C 1.284497011 0.11446013

G 0 0

T 0 0

G 1.281407035 0.27638191

A 0 0

G 5.312068316 0.188371217

C 4.809619238 0.563627255

C 1.961277345 0.289162685

A 0 0

C 3.706950532 1.077019411

C 0.70626813 0.517089166

G 0.414677055 0.427243026

T 0 0

G 5.616989193 0.125659713

C 7.031837553 1.090498872

C 2.772202709 0.163070748

C 0.288365095 1.291374122

G 0 0

G 2.804325956 0.289235412

C 2.455832602 0.13782734

T 0 0

G 0.376884422 0.226130653

A 0 0

G 2.211610958 0.490072883

T 0 0

T 0 0

G 3.292438658 0.162744116

T 0 0

A 0 0

C 3.528970091 0.312851958

A 0 0

A 0 0

A 0 0

C 8.822055138 0.087719298

C 1.04127462 0.326182411

T 0 0

T 0 0

T 0 0

A 0 0

A 0 0

C 0.877412885 0.263223866

T 0 0

C 0.728003012 0.125517761

A 0 0

A 0 0

T 0 0

G 7.273410115 0.41311968

C 7.400903161 0.401404917

C 2.560240964 0.138052209

T 0 0

G 0.965396189 0.488966901

G 2.298417483 0.339110776

A 0 0

A 0 0

G 1.145038168 0.547073791

A 0 0

G 0.997442455 0.74168798

A 0 0

G 1.814928425 0.677402863

T 0 0

T 0 0

G 1.237591853 0.567229599

T 0 0

G 1.373412801 0.518268982

T 0 0

G 2.484150602 0.090567991

C 2.378182758 0.129249063

T 0 0

T 0 0

A 0 0

A 0 0

A 0 0

A 0 0

G 1.243589744 0.153846154

A 0 0

C 1.686622892 0.090124887

A 0 0

C 1.926534806 0.642178269

T 0 0

C 0.681935152 0.038600103

A 0 0

T 0 0

T 0 0

T 0 0

T 0 0

C 0.498466258 0

T 0 0

T 0 0

T 0 0

T 0 0

T 0 0

T 0 0

C 2.356350806 0.050403226

C 0.740926786 0.075348487

T 0 0

G 0.327044025 0.150943396

A 0 0

A 0 0

G 0.996719657 0.012616705

T 0 0

G 0.544166034 0.088585168

A 0 0

T 0 0

G 2.72738805 0.15222631

G 2.796760314 0.101240192

A 0 0

A 0 0

G 0.863601727 0.088900178

A 0 0

T 0 0

A 0 0

T 0 0

T 0 0

T 0 0

T 0 0

C 0.508453032 0.050845303

T 0 0

A 0 0

G 1.285477918 0.21636757

G 2.668198647 0.191497511

A 0 0

T 0 0

C 3.627275058 0.128172264

C 1.90269442 0.012769761

T 0 0

T 0 0

T 0 0

T 0 0

A 0 0

T 0 0

C 0.716479017 0.115148414

T 0 0

G 0.97698933 0.218537087

T 0 0

A 0 0

A 0 0

T 0 0

T 0 0

T 0 0

C 1.215255333 0.206851972

C 0.595161082 0.116444559

A 0 0

G 3.223290876 0.337925656

C 2.682640969 0.039067587

T 0 0

T 0 0

T 0 0

C 0.378837361 0.078380144

A 0 0

A 0 0

T 0 0

G 3.278688525 0.092543628

G 5.323951142 0.17259692

G 5.398526457 0.066979236

A 0 0

G 1.449275362 0.582419071

A 0 0

G 1.324503311 0.676048565

A 0 0

C 1.958589815 0

A 0 0

C 1.648429729 0.454739235

A 0 0

T 0 0

C 0.798954096 0.087158629

A 0 0

A 0 0

G 1.231852178 0.205308696

A 0 0

C 1.714961561 0.916617386

A 0 0

A 0 0

G 1.959883632 0.122492727

G 7.762065785 0.292038119

T 0 0

A 0 0

A 0 0

T 0 0

A 0 0

A 0 0

C 0.899887514 0

T 0 0

T 0 0

A 0 0

T 0 0

A 0 0

C 2.467489163 0

A 0 0

A 0 0

T 0 0

T 0 0

G 1.179941003 2.169009197

T 0 0

C 0.736842105 0.01754386

T 0 0

G 0.918079096 1.288841808

T 0 0

C 1.464737794 0.054249548

A 0 0

T 0 0

T 0 0

C 2.220216606 0.342960289

C 1.329206118 0.163874727

A 0 0

G 0.848551928 3.578675521

G 2.515840477 0.298173686

A 0 0

A 0 0

T 0 0

G 2.395326193 3.193768257

T 0 0

G 1.599841991 0.592534071

G 2.863961814 0.656324582

C 2.616859768 0.039952058

A 0 0

G 3.00954753 0.705687007

T 0 0

A 0 0

G 2.798134577 0.777259605

G 7.669815565 0.719748088

C 6.537585421 0.045558087

A 0 0

C 3.478664193 0.185528757

T 0 0

G 0.218658892 0.923226433

A 0 0

A 0 0

A 0 0

C 0.362131402 0.129332644

G 0.340403247 2.356637863

T 0 0

T 0 0

C 2.551705614 0.376040827

T 0 0

C 8.22749862 0.055218112

C 8.030008336 0.083356488

T 0 0

T 0 0

T 0 0

G 0 0

T 0 0

A 0 0

A 0 0

C 1.176844784 2.194656489

T 0 0

C 1.340206186 0.378006873

T 0 0

A 0 0

C 7.297970808 3.41758633

T PRIMER NA NA

A PRIMER NA NA

C PRIMER NA NA

T PRIMER NA NA

C PRIMER NA NA

A PRIMER NA NA

C PRIMER NA NA

T PRIMER NA NA

T PRIMER NA NA

C PRIMER NA NA

A PRIMER NA NA

G PRIMER NA NA

A PRIMER NA NA

T PRIMER NA NA

T PRIMER NA NA

T PRIMER NA NA

T PRIMER NA NA

G PRIMER NA NA

G PRIMER NA NA

G PRIMER NA NA

A PRIMER NA NA

A PRIMER NA NA

T PRIMER NA NA

T PRIMER NA NA

G PRIMER NA NA

C PRIMER NA NA

C NA NA

A NA NA

C NA NA

A NA NA

G NA NA

T NA NA

T NA NA

A NA NA

A NA NA

G NA NA

T NA NA

A NA NA

C NA NA

T NA NA

C NA NA

T NA NA

T NA NA

C NA NA

C NA NA

A NA NA

T NA NA

A NA NA

G NA NA

T NA NA

A NA NA

G NA NA

C NA NA

C NA NA

T NA NA

G NA NA

T NA NA

G NA NA

A NA NA

A NA NA

C NA NA

C NA NA

T NA NA

G NA NA

T NA NA

G NA NA

G NA NA

C NA NA

T NA NA

G NA NA

G NA NA

G NA NA

A NA NA

G NA NA

A NA NA

A NA NA

A NA NA

T NA NA

A NA NA

C NA NA

C NA NA

C NA NA

T NA NA

C NA NA

T NA NA

G NA NA

T NA NA

G NA NA

G NA NA

T NA NA

G NA NA

G NA NA

G NA NA

A NA NA

A NA NA

G NA NA

A NA NA

G NA NA

C NA NA

A NA NA

T NA NA

A NA NA

G NA NA

C NA NA

C NA NA

A PRIMER NA NA

C PRIMER NA NA

A PRIMER NA NA

A PRIMER NA NA

C PRIMER NA NA

A PRIMER NA NA

T PRIMER NA NA

T PRIMER NA NA

G PRIMER NA NA

T PRIMER NA NA

G PRIMER NA NA

G PRIMER NA NA

A PRIMER NA NA

A PRIMER NA NA

A PRIMER NA NA

G PRIMER NA NA

T PRIMER NA NA

A PRIMER NA NA

G PRIMER NA NA

T PRIMER NA NA

C PRIMER NA NA

A PRIMER NA NA

T PRIMER NA NA

C PRIMER NA NA

T PRIMER NA NA

C PRIMER NA NA

A PRIMER NA NA

T 0 0

T 0 0

A 0 0

A 0 0

T 0 0

T 0 0

T 0 0

C 0.400291121 1.528384279

A 0 0

C 10.95505618 10.11235955

C 5.3161918 1.389854065

C 2.518720218 1.497617427

C 8.251150559 1.873767258

G 0.377687391 0.261475886

T 0 0

T 0 0

C 4 0.781609195

C 2.375138735 7.480577137

T 0 0

G 7.246989225 1.669131629

G 13.72793354 0.228452752

G 12.90001988 0.298151461

A 0 0

A 0 0

C 1.752072344 0.753579503

A 0 0

C 2.079866889 4.908485857

A 0 0

T 0 0

G 8.851828095 0.898011546

G 11.41312741 0.463320463

T 0 0

G 2.599731062 0.104586882

A 0 0

G 4.143963292 0.100372813

T 0 0

C 1.167477415 1.959694232

A 0 0

C 1.401208382 2.056819643

T 0 0

G 2.950737684 0.150037509

A 0 0

G 7.64132066 0.212606303

G 14.29643527 0.175109443

G 12.16858429 1.213106553

A 0 0

G 4.803001876 0.150093809

G 6.540770385 0.4002001

G 12.36713768 0.087532825

T 0 0

C 2.476857643 0.225168877

A 0 0

G 2.338376891 0

G 4.914342879 0.012504689

A 0 0

C 5.538884721 0.200050013

A 0 0

T 0 0

T 0 0

G 0.81321156 0.012510947

A 0 0

G 1.450181273 0.025003125

G 6.800850106 0.025003125

T 0 0

C 0.950356384 0.025009379

T 0 0

G 0.32508127 0.025006252

A 0 0

T 0 0

A 0 0

T 0 0

C 0.612653163 0

T 0 0

G 1.038408608 0

G 2.263914947 0.062539087

G 2.976116044 0.012504689

A 0 0

A 0 0

T 0 0

C 0.512756378 0.012506253

A 0 0

T 0 0

G 3.139069535 0.062531266

C 2.614133834 0.025015635

T 0 0

A 0 0

T 0 0

T 0 0

G 2.390787333 0.012517211

C 2.091683367 0.01252505

T 0 0

C 0.463136813 0

A 0 0

C 0.687585948 0.025003125

A 0 0

G 2.127393317 0.07508447

C 2.02575966 0.037514068

A 0 0

C 12.16977589 0.012520346

C 7.185778668 0.050075113

C 2.59009009 0.037537538

T 0 0

C 2.302014262 0.012510947

T 0 0

T 0 0

C 0.939025917 0

T 0 0

T 0 0

T 0 0

G 1.480737859 0.075291756

G 2.68136825 0.125297582

G 9.175115784 0.050068845

T 0 0

G 1.727375141 0.037551633

T 0 0

G 0.939143501 0.025043827

T 0 0

C 0.112584438 0

G 0.112669004 0.012518778

T 0 0

T 0 0

A 0 0

G 0.763167772 0.025021894

T 0 0

T 0 0

T 0 0

C 1.677306296 0.012517211

C 0.663744521 0.012523482

A 0 0

A 0 0

T 0 0

T 0 0

A 0 0

G 1.618163573 0.025087807

G 4.12642669 0.062711652

G 3.036386449 0.025094103

A 0 0

T 0 0

T 0 0

T 0 0

C 0.312656328 0.037518759

T 0 0

G 0.501253133 0.025062657

A 0 0

T 0 0

G 0.55130936 0.012529758

A 0 0

G 2.975517891 0.037664783

T 0 0

C 3.528970091 0.025028157

C 1.866466241 0.050106476

T 0 0

G 1.753946379 0.025056377

A 0 0

C 8.970349055 0.025021894

A 0 0

T 0 0

A 0 0

A 0 0

C 0.175219024 0.025031289

G 0.150451354 0.075225677

C 0.801402454 0

T 0 0

G 0.676606941 0.012529758

T 0 0

T 0 0

T 0 0

G 1.426962073 0.1126549

C 1.67604753 0.037523452

T 0 0

T 0 0

T 0 0

G 0.628377529 0.012567551

T 0 0

G 0.752351097 0.05015674

G 1.607232546 0.100452034

A 0 0

T 0 0

A 0 0

T 0 0

T 0 0

T 0 0

A 0 0

A 0 0

A 0 0

A 0 0

C 9.196842501 0.162886856

A 0 0

T 0 0

A 0 0

G 5.418286718 0.250846607

C 5.132056578 0.050068845

T 0 0

T 0 0

A 0 0

C 8.326029798 0.025040691

C 1.655795283 0.175614651

T 0 0

G 4.459798995 0.263819095

T 0 0

A 0 0

G 12.20373172 0.226928896

C 9.689971131 0.188276641

C 7.560267575 0.037864445

C 4.120982987 0.126023945

T 0 0

C 1.040230605 0.100263191

A 0 0

A 0 0

A 0 0

T 0 0

A 0 0

C 2.467125861 0.050093926

A 0 0

T 0 0

G 2.680256701 0.302000755

G 2.518574487 0.780758091

A 0 0

A 0 0

A 0 0

T 0 0

C 1.528163678 0.328365749

C 1.762558227 0.125897016

A 0 0

T 0 0

T 0 0

T 0 0

T 0 0

A 0 0

T 0 0

T 0 0

C 5.757385292 0.100565682

C 7.054673721 0.365331318

C 3.028779691 0.037702652

T 0 0

T 0 0

A 0 0

C 0.175989943 0.301697046

G 0.203899579 0.254874474

C 1.875792142 0.659062104

T 0 0

C 8.22141333 0.451863939

C 9.920884089 0.11302273

C 3.891687657 0.277078086

T 0 0

C 3.090840558 0.263852243

A 0 0

T 0 0

G 4.194925898 0.263752826

T 0 0

A 0 0

A 0 0

G 5.637223572 0.715837914

C 4.666917576 0.602182913

T 0 0

A 0 0

G 1.155343464 1.494411654

T 0 0

T 0 0

T 0 0

A 0 0

A 0 0

A 0 0

A 0 0

A 0 0

T 0 0

G 0.870992174 1.237061348

A 0 0

G 1.488207845 0.668432337

T 0 0

C 0.829458339 0.251351012

A 0 0

G 0.679330733 0.629009938

A 0 0

T 0 0

G 3.501205125 0.266396042

C 2.834922228 0.250878073

T 0 0

A 0 0

C 2.491819784 0.163604329

A 0 0

G 3.181242079 0.51964512

C 3.331657028 0.440030173

T 0 0

G 0.539658635 0.188253012

T 0 0

T 0 0

T 0 0

T 0 0

G 2.058672156 0.566134843

G 5.279503106 0.207039337

G 5.429571077 0.505377737

A 0 0

C 2.80169644 0.179925459

A 0 0

G 0.327208658 0.843191543

A 0 0

A 0 0

A 0 0

T 0 0

A 0 0

C 4.805406082 0.212739332

C 1.614530777 0.681130172

A 0 0

C 1.807455755 0.364001506

T 0 0

G 0.883615249 0.32819995

A 0 0

C 4.348370927 0.125313283

C 1.383821864 0.389986162

A 0 0

C 1.667084482 0.037603409

T 0 0

T 0 0

G 1.25284738 0.189825361

A 0 0

A 0 0

G 0.981892374 0.382555471

C 0.114547537 0.432735141

G 0.126758778 0.469007479

A 0 0

T 0 0

G 1.960784314 0.062845651

T 0 0

T 0 0

G 0.6655783 0.200929298

A 0 0

A 0 0

G 0.831653226 0.113407258

T 0 0

T 0 0

G 1.6797171 0.164182874

C 2.183516345 0.037864445

T 0 0

T 0 0

A 0 0

T 0 0

T 0 0

T 0 0

T 0 0

G 1.278157428 0.050620096

C 2.94375548 0.238005762

C 0.852023556 0.125297582

A 0 0

G 0.801101515 0.012517211

A 0 0

G 1.275956968 0.225168877

T 0 0

T 0 0

G 0.889501378 0.087697319

A 0 0

G 1.704260652 0.050125313

G 6.433846539 0.100137689

C 3.909774436 0.175438596

A 0 0

G 0.914099674 0.050087653

T 0 0

T 0 0

T 0 0

A 0 0

T 0 0

T 0 0

C 0.963704631 0.200250313

A 0 0

T 0 0

C 0.850531582 0.175109443

T 0 0

A 0 0

T 0 0

G 10.5058853 0

T 0 0

T 0 0

C 0.95214232 0.075169131

A 0 0

G 1.241534989 0.225733634

G 6.629834254 0.08789553

T 0 0

G 3.272727273 0.112852665

G 4.529485571 0.17565872

A 0 0

C 10.44645097 0.05016303

C 2.762084118 0.163214062

C 1.415507954 0.025053238

A 0 0

A 0 0

A 0 0

C 0.13770656 0.125187782

G 0.188111362 0.213192877

A 0 0

G 3.422768305 0.062688064

C 4.496492986 0.288076152

C 1.754385965 0.025062657

A 0 0

T 0 0

C 0.952261621 0.476130811

A 0 0

T 0 0

T 0 0

C 0.575791714 0.012517211

T 0 0

G 0.915016295 0.125344698

G 3.347962382 0.062695925

T 0 0

G 0.613958151 0.100238065

T 0 0

T 0 0

T 0 0

A 0 0

A 0 0

A 0 0

A 0 0

T 0 0

T 0 0

C 0.764123763 0.112739572

A 0 0

T 0 0

T 0 0

A 0 0

T 0 0

A 0 0

T 0 0

T 0 0

G 2.648092369 0.15060241

T 0 0

A 0 0

G 1.54154656 0.050131595

T 0 0

G 0.666666667 0.075471698

A 0 0

G 0.639578631 0.10032606

A 0 0

T 0 0

G 0.426011778 0.13782734

A 0 0

A 0 0

A 0 0

T 0 0

A 0 0

A 0 0

A 0 0

A 0 0

T 0 0

A 0 0

G 0.764698508 0.050144164

A 0 0

G 1.479067435 0.451240913

T 0 0

G 0.752351097 0.300940439

A 0 0

T 0 0

A 0 0

T 0 0

C 6.348011542 0.23836407

C 6.370147003 0.05025757

C 3.706495791 0.263852243

T 0 0

A 0 0

T 0 0

A 0 0

G 4.640080261 0.376222724

C 4.896421846 0.025109856

T 0 0

T 0 0

C 7.870660484 0.363454067

C 7.320441989 0.100452034

C 5.025757005 0.314109813

C 2.725445868 0.07535795

A 0 0

A 0 0

G 5.332496863 0.125470514

C 15.21603006 0.025046963

A 0 0

T 0 0

A 0 0

A 0 0

A 0 0

A 0 0

A 0 0

G 0.502638854 0.075395828

A 0 0

A 0 0

T 0 0

A 0 0

A 0 0

T 0 0

T 0 0

A 0 0

A 0 0

A 0 0

T 0 0

A 0 0

T 0 0

A 0 0

G 2.374073609 0.087928652

C 2.333458788 0.250909547

A 0 0

C 1.791530945 0.313204711

A 0 0

C 2.08385639 0.050213407

T 0 0

C 1.053291536 0.10031348

T 0 0

T 0 0

C 1.557006529 0.075339026

A 0 0

C 1.903092525 0.050081382

A 0 0

C 2.207173313 0.087785302

A 0 0

T 0 0

C 1.457469531 0.175901495

T 0 0

C 1.325662831 0.037518759

T 0 0

T 0 0

C 0.964428858 0.137775551

A 0 0

T 0 0

T 0 0

A 0 0

C 3.650275966 0.188158555

C 1.162349968 0.795957044

A 0 0

A 0 0

A 0 0

C 9.845341381 0.867597133

A 0 0

T 0 0

T 0 0

G 0.781545443 0.365561578

A 0 0

T 0 0

T 0 0

A 0 0

A 0 0

G 2.200427512 0.0880171

T 0 0

G 4.149848638 0.126135217

C 7.352941176 0.56561086

C 2.477987421 0.377358491

T 0 0

G 2.656092649 0.163645519

C 2.757929046 0.087752288

T 0 0

T 0 0

C 11.55728448 0.163132137

C 11.99445634 0.32757969

C 11.57988091 0.126694539

C 9.960863527 0.416614064

C 4.281037522 0.27700831

A 0 0

G 4.733727811 0.528767468

C 4.23814329 0.340565086

T 0 0

C 0.791059769 50.86639879

T 0 0

G 5.560481317 0.278657378

C 9.155811623 0.263026052

C 5.549278092 0.376647834

T 0 0

C 14.82039689 0.502386335

C 16.20323559 0.442366026

C 14.71513017 0.176078481

C 15.56141673 0.38934941

C 7.931904161 0.176544767

T 0 0

T 0 0

A 0 0

T 0 0

C 6.187248996 0.514558233

C 6.118594989 0.188845524

C 2.473010294 0.50213407

T 0 0

G 1.193317422 0.602939329

A 0 0

T 0 0

G 7.516998237 0.881390078

G 2.741793485 0.679159854

A 0 0

G 7.280701754 0.37593985

A 0 0

C 1.264396595 0.212819229

A 0 0

A 0 0

T 0 0

G 1.884185404 0.175857304

A 0 0

C 7.176973353 0.301659125

C 2.310977141 0.125596584

T 0 0

T 0 0

G 1.167022211 0.025097252

A 0 0

A 0 0

G 1.667084482 0.175482577

A 0 0

G 8.635621941 0.112965985

C 8.384586419 0.150621313

C 3.03334169 0.037603409

T 0 0

T 0 0

T 0 0

A 0 0

G 1.17661785 0.100137689

T 0 0

T 0 0

T 0 0

G 1.668548488 0.062727387

T 0 0

C 2.604231877 0.100162764

C 4.958677686 0.050087653

A 0 0

T 0 0

A 0 0

A 0 0

T 0 0

T 0 0

A 0 0

A 0 0

A 0 0

A 0 0

T 0 0

G 2.793086172 0.01252505

C 2.4066182 0.112810228

A 0 0

T 0 0

T 0 0

C 0.82592917 0.100112627

T 0 0

A 0 0

T 0 0

C 0.76450683 0.062664494

T 0 0

A 0 0

T 0 0

A 0 0

T 0 0

G 9.873449442 0.112767824

T 0 0

G 0.989602906 0.175372667

T 0 0

G 0.288256674 0.275723775

A 0 0

A 0 0

A 0 0

A 0 0

T 0 0

A 0 0

A 0 0

A 0 0

T 0 0

A 0 0

T 0 0

C 0.625547354 0.075065682

T 0 0

T 0 0

T 0 0

T 0 0

G 0.464124436 0.037631711

A 0 0

A 0 0

C 22.21387428 0.025043827

A 0 0

T 0 0

A 0 0

T 0 0

T 0 0

G 1.017971597 0.062837753

A 0 0

A 0 0

C 1.054216867 0.025100402

A 0 0

A 0 0

A 0 0

T 0 0

T 0 0

G 1.885843601 0.213728941

G 3.611880191 0.125849484

G 4.975437713 0.075576269

A 0 0

G 0.953934982 0.037655328

A 0 0

A 0 0

A 0 0

G 1.290726817 0.037593985

A 0 0

C 1.702980215 0.062609567

A 0 0

C 1.754825771 0.037603409

T 0 0

G 6.240601504 0.07518797

T 0 0

A 0 0

C 16.0667252 0.087796313

C 7.614978638 0.125659713

C 4.147755925 0.13867877

T 0 0

T 0 0

T 0 0

C 0.35131744 0.025094103

A 0 0

A 0 0

A 0 0

T 0 0

T 0 0

A 0 0

A 0 0

C 10.54804805 0.025025025

A 0 0

T 0 0

T 0 0

A 0 0

T 0 0

G 6.549780839 0.062617408

A 0 0

T 0 0

A 0 0

A 0 0

T 0 0

T 0 0

T 0 0

T 0 0

A 0 0

A 0 0

A 0 0

A 0 0

A 0 0

T 0 0

T 0 0

A 0 0

A 0 0

C 1.188242652 0.025015635

T 0 0

A 0 0

A 0 0

G 6.955099987 0.075462206

C 7.1150709 0.06274313

C 2.651753173 0.025135101

T 0 0

T 0 0

T 0 0

T 0 0

C 2.345121645 0.112866817

C 1.86327584 0.07553821

A 0 0

C 1.579739218 0.025075226

T 0 0

G 3.054298643 0.150829563

G 7.73711536 0.189945549

C 13.26286649 0.075500189

A 0 0

T 0 0

A 0 0

T 0 0

T 0 0

A 0 0

A 0 0

T 0 0

G 1.890597429 0.189059743

A 0 0

G 3.004291845 0.113607675

A 0 0

C 8.390905665 0.113051124

C 3.307146477 0.266092245

C 2.118804389 0.075671585

A 0 0

G 2.786885246 0.567465322

G 4.673487432 0.113679424

A 0 0

G 2.796674225 0.07558579

T 0 0

C 1.490651844 0.429509853

A 0 0

C 2.36575291 0.488171235

T 0 0

A 0 0

T 0 0

T 0 0

A 0 0

T 0 0

C 2.984326019 0.313479624

C 2.157184307 0.113536016

A 0 0

C 1.66520596 0.037561037

T 0 0

G 1.278676194 0.150432493

T 0 0

T 0 0

A 0 0

T 0 0

T 0 0

A 0 0

A 0 0

A 0 0

T 0 0

T 0 0

A 0 0

A 0 0

A 0 0

C 5.218263924 0.062719518

A 0 0

T 0 0

T 0 0

T 0 0

T 0 0

A 0 0

G 1.194367614 0.364596429

G 2.765500695 0.050511428

A 0 0

A 0 0

T 0 0

A 0 0

A 0 0

A 0 0

G 5.470321932 0.238933602

C 4.340190667 0.163070748

T 0 0

C 0.74176515 0.025144581

A 0 0

T 0 0

C 2.179631717 0.212952524

C 1.270120724 0.163480885

A 0 0

A 0 0

T 0 0

T 0 0

T 0 0

A 0 0

C 1.213865599 0.125140783

T 0 0

T 0 0

A 0 0

G 0.477147162 0.062782521

A 0 0

A 0 0

A 0 0

A 0 0

G 1.914357683 0.289672544

G 9.274040289 0.050677816

T 0 0

C 1.742072941 0.200526382

T 0 0

T 0 0

G 1.661840614 0.07553821

G 4.448645243 0.365469439

C 3.235553317 0.239204331

T 0 0

A 0 0

C 2.066633267 0.100200401

A 0 0

A 0 0

A 0 0

T 0 0

A 0 0

C 1.101265823 0.075949367

A 0 0

G 1.507159005 0.113036925

T 0 0

T 0 0

G 1.735412475 0.100603622

C 1.329113924 0.113924051

A 0 0

C 1.40562249 0.037650602

T 0 0

T 0 0

A 0 0

G 1.608646475 0.087972854

T 0 0

T 0 0

A 0 0

G 3.718542632 0.262927257

C 3.16662478 0.138225685

T 0 0

T 0 0

C 0.478047553 0.113221789

T 0 0

A 0 0

A 0 0

T 0 0

T 0 0

T 0 0

G 1.468373494 0.213353414

C 1.103448276 0.05015674

T 0 0

T 0 0

T 0 0

G 1.055011304 0.113036925

T 0 0

T 0 0

G 1.279317697 0.388812241

G 3.706495791 0.087950748

T 0 0

T 0 0

G 1.052631579 0.513784461

A 0 0

A 0 0

C 20.02776376 0.630994447

A 0 0

T 0 0

A 0 0

A 0 0

A 0 0

G 7.149150409 0.276903713

G 9.463881198 0.226529071

G 14.86741234 0.125675506

T 0 0

G 6.214689266 0.100439422

G 8.63871049 0.201485959

G 15.50748334 0.176078481

T 0 0

C 9.637799223 0.087730292

C 7.90668348 0.214375788

C 4.751701538 0.214267709

A 0 0

C 3.505257887 0.287931898

T 0 0

G 9.77443609 0.501253133

C 9.773869347 0.326633166

C 8.503956789 0.06280618

C 4.875297656 0.213059281

T 0 0

C 1.689400576 0.387936428

T 0 0

A 0 0

G 8.449291714 0.463833521

C 7.569870911 0.451184359

C 2.800803818 0.037678975

T 0 0

T 0 0

C 5.789473684 0.07518797

C 3.741368487 0.251098556

C 1.684475173 0.339409177

A 0 0

C 0.250815149 0.025081515

G 0 0

G 2.386634845 0.12561236

G 7.347760443 0.163563161

C 5.777666374 0.087730292

A 0 0

T 0 0

C 0.801804059 0.526183914

T 0 0

T 0 0

G 1.164537941 0.050087653

T 0 0

T 0 0

T 0 0

G 0.627116518 0.075253982

A 0 0

G 1.654342649 0.062664494

T 0 0

C 0.525722869 0.012517211

A 0 0

G 0.826653307 0.0501002

T 0 0

T 0 0

G 0.626017278 0.062601728

A 0 0

T 0 0

T 0 0

A 0 0

A 0 0

T 0 0

T 0 0

T 0 0

T 0 0

G 0.690261044 0.075301205

A 0 0

G 0.940320963 0.112838516

A 0 0

G 4.390366282 0.025087807

C 3.720871962 0.150338261

C 1.07728924 0.037579857

A 0 0

G 4.046604861 0.062640942

C 3.14221332 0.087631447

T 0 0

C 0.363362987 0.037589275

A 0 0

G 0.601729974 0.050144164

A 0 0

C 1.001376893 0.1126549

T 0 0

T 0 0

T 0 0

G 1.065697091 0.050150451

G 2.355594537 0.213005889

A 0 0

T 0 0

A 0 0

C 1.67982951 0.11282437

A 0 0

A 0 0

G 2.707106154 0

T 0 0

C 1.140207994 0.062648791

T 0 0

C 0.926157697 0.075093867

T 0 0

G 3.720871962 0.087697319

T 0 0

G 9.630374654 0.025144581

C 9.207225289 0.112895133

C 7.968277946 0.075528701

C 4.3122957 0.100578325

T 0 0

T 0 0

T 0 0

G 1.441102757 0.401002506

T 0 0

T 0 0

C 4.441656211 0.250941029

C 1.531316681 0.389105058

T 0 0

G 1.31595438 0.200526382

T 0 0

C 0.866072549 0.075310656

A 0 0

A 0 0

G 1.796482412 0.314070352

T 0 0

G 2.181544634 0.213139418

T 0 0

T 0 0

G 1.698968034 0.276868865

T 0 0

C 0.991465863 0.062751004

A 0 0

C 1.428392432 0.200476131

T 0 0

G 1.865998748 0.32561052

T 0 0

T 0 0

G 1.245126399 0.477927305

T 0 0

C 0.916739922 0.075348487

T 0 0

T 0 0

G 0.606290261 0.543135026

A 0 0

A 0 0

A 0 0

C 17.74375706 0.326264274

A 0 0

T 0 0

A 0 0

T 0 0

T 0 0

C 0.679159854 0.301848824

A 0 0

A 0 0

G 3.929248263 0.240050537

G 6.484986122 0.517284885

A 0 0

G 5.612886987 0.352378555

G 9.848009044 0.276347193

C 5.222753587 0.07550969

A 0 0

C 8.477335337 0.41322314

C 2.434409687 0.643289606

T 0 0

T 0 0

C 5.374858722 0.213487379

C 3.518471978 0.251319427

C 2.013591744 0.289453813

A 0 0

A 0 0

T 0 0

G 2.300884956 0.303413401

A 0 0

G 3.921075782 0.175945708

G 6.966800805 0.477867203

A 0 0

G 7.177033493 0.629564341

C 4.663407296 0.325937069

T 0 0

C 0.952380952 0.263157895

C 0.401808137 0.163234555

G 0.213836478 0.113207547

T 0 0

T 0 0

A 0 0

G 2.044399849 0.476608554

G 7.098068723 0.137948332

T 0 0

G 2.135410124 0.251224721

T 0 0

G 2.09273183 0.288220551

G 6.019563582 0.087785302

T 0 0

A 0 0

G 1.73062453 0.238274392

A 0 0

G 2.470839082 0.238304277

T 0 0

G 1.241134752 0.189969605

A 0 0

C 0.977198697 0.037584565

T 0 0

G 0.413171404 0.388130712

A 0 0

T 0 0

T 0 0

A 0 0

A 0 0

G 1.327987973 0.212979203

T 0 0

T 0 0

A 0 0

T 0 0

G 18.8752505 0.125250501

T 0 0

T 0 0

T 0 0

A 0 0

A 0 0

A 0 0

A 0 0

G 0.401606426 0.200803213

A 0 0

T 0 0

A 0 0

T 0 0

T 0 0

A 0 0

A 0 0

G 2.180177923 0.037589275

T 0 0

C 1.984426024 0.025119317

C 1.441283369 0.087730292

A 0 0

G 1.503947863 0.125328989

T 0 0

G 0.81321156 0.150131365

A 0 0

C 0.952500313 0.087730292

A 0 0

A 0 0

A 0 0

T 0 0

G 0.514042126 0.050150451

A 0 0

A 0 0

A 0 0

C 1.00565682 0.01257071

A 0 0

G 3.931173072 0.07535795

G 8.767295597 0.062893082

G 11.56214654 0.100540405

C 9.287419882 0.125675506

A 0 0

T 0 0

G 5.265141995 0.113093742

G 4.763705104 0.214240706

G 10.85728631 0.225931969

C 9.082779765 0.02554931

T 0 0

T 0 0

G 2.54290367 0.112739572

T 0 0

A 0 0

A 0 0

T 0 0

T 0 0

G 2.861804945 0.615037028

G 6.958050741 0.439588043

T 0 0

C 4.495793043 0.263719704

C 2.659307577 0.363773206

T 0 0

G 3.258145363 0.238095238

G 7.697137117 0.439477649

T 0 0

A 0 0

G 5.29153605 0.150470219

G 10.86407276 0.151591713

G 11.48716658 0.918470055

A 0 0

G 6.818751567 0.162948107

G 11.1657835 0.277567499

G 10.37522035 0.881390078

A 0 0

G 3.072100313 0.188087774

A 0 0

G 2.25846926 0.526976161

A 0 0

G 2.934169279 0.250783699

T 0 0

G 2.26788623 0.989850896

T 0 0

G 2.828889723 1.051445738

T 0 0

G 1.651858341 0.788386935

A 0 0

T 0 0

G 3.298219212 0.401304239

G 7.646985082 0.501441645

T 0 0

A 0 0

G 2.172820899 0.46470736

G 4.869149472 0.176144942

A 0 0

T 0 0

G 3.073641952 0.301091457

G 5.625313913 0.477147162

T 0 0

G 2.768729642 0.375845653

G 5.127240817 0.33847311

C 2.572791165 0.050200803

A 0 0

T 0 0

C 0.513784461 0.162907268

T 0 0

T 0 0

T 0 0

G 1.22684026 0.363044567

G 5.727534779 0.275723775

T 0 0

A 0 0

G 2.317424527 0.363271953

G 4.634760705 0.188916877

A 0 0

T 0 0

G 1.440380762 0.087675351

A 0 0

G 2.467125861 0.388227927

T 0 0

G 1.614316106 0.362908272

T 0 0

C 1.065162907 0.100250627

A 0 0

G 1.34034824 0.613804334

G 6.908224674 0.288365095

T 0 0

C 1.093239507 0.402111083

A 0 0

A 0 0

C 1.454363089 0.188064193

A 0 0

C 1.405446104 0.075291756

T 0 0

C 1.127254509 0.137775551

A 0 0

T 0 0

C 1.527099762 0.062586056

A 0 0

T 0 0

A 0 0

A 0 0

G 2.591387081 0.388082123

C 1.816359764 0.112739572

A 0 0

A 0 0

G 0.890505456 0.112880973

A 0 0

T 0 0

G 0.751408892 0.075140889

A 0 0

A 0 0

T 0 0

G 2.040816327 0.550895205

A 0 0

G 4.335839599 0.162907268

G 10.53623006 0.025116162

G 8.675396626 0.742885923

A 0 0

C 3.996492107 0.563768479

A 0 0

A 0 0

C 0.254097319 0.050819464

G 0.338940497 0.087873462

T 0 0

G 3.53560682 0.514042126

T 0 0

G 4.989344365 0.288328946

G 5.499622452 0.176189278

G 11.51720673 0.339110776

C 7.428786548 0.125486259

T 0 0

G 2.314174318 0.503081373

G 4.177151485 0.591343734

G 5.251256281 0.74120603

A 0 0

T 0 0

G 2.372285678 0.301242626

A 0 0

C 3.292698253 0.087972854

C 1.392547986 0.464182662

A 0 0

A 0 0

T 0 0

A 0 0

G 4.754139488 0.501756147

C 2.950778503 0.376695128

T 0 0

T 0 0

T 0 0

G 0.528967254 0.16372796

A 0 0

A 0 0

G 5.111139018 0.288835866

C 3.970846946 0.213621513

T 0 0

G 1.856497742 0.012543904

C 2.921996489 0.15048909

C 1.054746359 0.138121547

A 0 0

A 0 0

C 3.211239338 0.13798294

C 0.853306563 0.552139541

A 0 0

G 6.737766625 0.150564617

C 5.902995363 0.350921168

C 2.401609456 0.238903558

T 0 0

T 0 0

C 0.864661654 0.112781955

T 0 0

T 0 0

A 0 0

A 0 0

T 0 0

C 0.413067968 0.300413068

A 0 0

G 1.078369906 0.12539185

A 0 0

C 1.126549005 0.21279259

T 0 0

A 0 0

A 0 0

G 3.621100113 0.050119033

T 0 0

A 0 0

C 3.247648903 0.062695925

A 0 0

G 4.096204434 0.13779281

T 0 0

A 0 0

C 3.010914565 0.075272864

A 0 0

G 0.954077089 0.826866811

A 0 0

A 0 0

G 1.580278129 0.581542351

A 0 0

C 1.762780156 0.062956434

T 0 0

T 0 0

G 1.896984925 0.364321608

T 0 0

G 0.542792224 0.441807624

A 0 0

T 0 0

T 0 0

T 0 0

A 0 0

G 1.465063862 0.175306787

T 0 0

T 0 0

A 0 0

C 3.135975916 0.112895133

C 0.654252642 0.465525918

A 0 0

A 0 0

A 0 0

C 1.165559594 0.125328989

T 0 0

A 0 0

C 1.456737411 0.163255055

T 0 0

A 0 0

A 0 0

A 0 0

C 0.438816449 0.125376128

T 0 0

T 0 0

T 0 0

C 0.552070263 0.188205772

T 0 0

T 0 0

T 0 0

A 0 0

T 0 0

G 2.452521695 0.08803924

A 0 0

G 3.67341804 0.10064159

G 7.045797685 0.176144942

G 13.96901373 0.251920897

T 0 0

G 1.09145653 0.062727387

A 0 0

A 0 0

G 2.903104185 0.251351012

G 8.999243761 0.17645576

C 6.90479185 0.08803924

T 0 0

T 0 0

C 0.801001252 0.175219024

T 0 0

T 0 0

A 0 0

A 0 0

G 0.928016052 0.213192877

A 0 0

G 1.190327027 0.150357098

A 0 0

C 1.653099562 0

A 0 0

A 0 0

T 0 0

T 0 0

A 0 0

A 0 0

T 0 0

G 5.860989355 0.187852223

T 0 0

G 2.481825019 0.150413638

T 0 0

A 0 0

G 1.079317269 0.288654618

A 0 0

T 0 0

T 0 0

C 0.513977686 0.22564874

A 0 0

C 1.089679359 0.288076152

A 0 0

A 0 0

G 1.00590972 0.352068402

A 0 0

T 0 0

G 2.194907814 0.326100589

T 0 0

T 0 0

C 0.66449348 0.137913741

T 0 0

A 0 0

A 0 0

G 2.086737901 0.163419233

G 6.922688226 0.13895907

C 3.294892916 0.05069066

A 0 0

A 0 0

A 0 0

C 0.741019844 0.200954534

T 0 0

G 1.24246988 0.840863454

G 4.342353682 0.704845815

T 0 0

C 1.090635577 0.11282437

T 0 0

G 0.577744285 0.728460186

A 0 0

G 3.648902821 0.401253918

C 3.046707793 0.503588065

C 0.969162996 0.138451857

A 0 0

A 0 0

A 0 0

G 1.093376901 0.276486113

A 0 0

A 0 0

G 4.35655995 0.376647834

C 3.416656199 1.180756186

C 0.78223568 0.52990159

A 0 0

C 0.150696974 0.653020219

G 0 0

G 1.749528005 0.302076778

A 0 0

G 1.774477725 0.226529071

A 0 0

C 4.641244355 0.301053688

C 1.963004908 0.968919089

A 0 0

G 3.558405633 0.125738715

G 8.566762969 0.175857304

G 8.472012103 0.416036309

A 0 0

G 3.553936958 0.715810624

A 0 0

G 4.238776022 0.928016052

A 0 0

G 16.65621472 1.128809733

C 11.65072974 0.301962758

C 10.65222866 0.188869302

C 8.819453194 0.226785939

C 5.625790139 0.265486726

A 0 0

C 12.22417252 0.075225677

C 5.18713891 0.439588043

C 3.261964736 0.113350126

A 0 0

C 5.575742388 0.050119033

C 1.771579344 0.540268878

A 0 0

G 2.23505776 0.464590658

C 1.678146525 1.051972448

A 0 0

A 0 0

G 2.532597793 0.376128385

G 5.520702635 0.702634881

A 0 0

T 0 0

A 0 0

T 0 0

A 0 0

T 0 0

A 0 0

A 0 0

A 0 0

A 0 0

G 4.264392324 0.112880973

C 3.703703704 0.025109856

T 0 0

C 0.588972431 0.087719298

A 0 0

G 1.640987098 0.889389954

G 3.80952381 0.050125313

A 0 0

G 2.996489468 0.050150451

T 0 0

C 0.888610763 0.125156446

T 0 0

G 1.740545955 0.237916354

G 2.745048884 0.188017047

A 0 0

G 2.37946149 0.225422668

T 0 0

G 1.854171887 0.23803558

A 0 0

C 3.400677626 0.112937633

A 0 0

T 0 0

T 0 0

C 0.425425425 0.137637638

A 0 0

C 0.926157697 0.187734668

A 0 0

A 0 0

C 1.667084482 0.087741289

T 0 0

C 0.501002004 0.0501002

A 0 0

G 1.429287864 0.238214644

G 4.355466298 0.087862433

A 0 0

G 2.371987952 0.138052209

G 9.333835152 0.100363819

T 0 0

A 0 0

A 0 0

C 1.427676894 0.125234815

T 0 0

T 0 0

C 0.288365095 0.363590772

A 0 0

G 1.791530945 0.300676522

C 1.516100739 0.175416614

A 0 0

A 0 0

G 5.015082956 0.314228255

C 4.477050414 0.489089541

T 0 0

A 0 0

C 8.461634748 0.025034422

C 1.479623824 0.438871473

T 0 0

G 1.506402209 0.251067035

C 1.618366579 0.163091206

A 0 0

G 11.20873604 0.100414209

C 10.4140527 0.476787955

C 8.429793659 0.163563161

C 4.874371859 0.27638191

T 0 0

C 1.101790409 0.262927257

T 0 0

G 1.06449593 0.212899186

T 0 0

C 0.966001756 0.163091206

T 0 0

G 0.991092711 0.439091707

A 0 0

C 2.505951635 0.238065405

A 0 0

T 0 0

C 0.924518744 0.240628166

A 0 0

T 0 0

G 5.847733534 0.313047834

T 0 0

C 5.164201554 0.188017047

C 3.383882692 0.087730292

T 0 0

T 0 0

C 1.027568922 0.275689223

A 0 0

A 0 0

C 1.151871792 0.162764492

T 0 0

G 1.832559307 0.527174595

C 2.91018565 0.338685399

T 0 0

C 2.515329746 0.30033788

C 3.060712494 0.188158555

A 0 0

C 1.17824016 0.313361745

A 0 0

A 0 0

G 0.491369535 0.075595313

A 0 0

A 0 0

A 0 0

T 0 0

T 0 0

G 1.129092962 0.250909547

C 1.36454682 0.27541312

T 0 0

C 1.063696659 0.262795645

T 0 0

T 0 0

C 2.614133834 0.037523452

C 2.080721985 0.125344698

A 0 0

G 6.428930186 0.050226017

G 18.00929999 0.201080809

C 13.87181738 0.087796313

C 9.263448969 0.150829563

C 9.234828496 0.062821963

A 0 0

T 0 0

T 0 0

G 2.412363362 0.288981028

G 2.836253624 0.30253372

A 0 0

G 2.801833928 0.254712175

G 3.990341848 0.127080951

A 0 0

C 0.190621426 0.406659042

G 0.267481849 0.40759139

C 1.143002286 0.292100584

T 0 0

G 1.917460317 0.317460317

C 4.537964254 0.304221067

A 0 0

T 0 0

T 0 0

G 2.09983654 0.264051301

T 0 0

T 0 0

C 3.481963928 0.175350701

C 2.038505096 0.213917201

A 0 0

G 2.770117824 0.275758335

T 0 0

G 5.278371245 0.188513259

G 12.03377016 0.277217742

C 11.92394863 0.100730295

C 7.903995979 0.364413169

C 4.624639679 0.338388269

A 0 0

A 0 0

G 1.05434919 0.050207104

T 0 0

T 0 0

A 0 0

C 4.363914763 0.574199311

C 1.122592167 0.229621125

A 0 0

C 0.151381355 0.428913839

G 0 0

A 0 0

C 1.125584925 0.088529151

T 0 0

T 0 0

C 2.693898008 0.300714196

C 2.018049637 0.250689396

A 0 0

C 5.242742743 0.112612613

C 1.8555667 0.501504514

A 0 0

C 1.439659489 0.27541312

T 0 0

G 0.5799294 0.718608169

A 0 0

T 0 0

G 2.735409051 0.138661288

C 1.764335224 0.050409578

T 0 0

G 0.705200856 0.491122025

A 0 0

C 1.555248965 0.213219616

T 0 0

G 3.905953735 0.189609405

C 6.461810824 0.075872534

C 2.155552754 0.037816715

T 0 0

G 1.939790906 0.188940673

G 3.04414003 0.164890918

G 6.604013208 0.368300737

C 0.266768293 0.228658537

G 0 0

G 2.698554802 0.025578719

C 2.219354839 0.361290323

A 0 0

T 0 0

C 0.439901669 0.090567991

T 0 0

G 0.570465448 0.453779334

T 0 0

T 0 0

T 0 0

G 4.940197608 0.02600104

C 7.456482203 0.012990387

C 4.255871286 0.298429999

C 2.246169826 0.129836406

A 0 0

G 1.750261233 0.222048067

T 0 0

T 0 0

C 5.004609509 0.250230475

C 2.513559995 0.039687789

T 0 0

T 0 0

C 2.91913215 0.499671269

C 1.695360757 0.354842949

A 0 0

G 0.793544048 0.349697377

A 0 0

C 1.252693966 0.161637931

T 0 0

G 1.328585125 0.109574031

G 4.377150723 1.114934618

C 4.043052298 0.027597627

T 0 0

C 1.252261027 0.431334354

T 0 0

T 0 0

G 0 0

G 4.305477132 0.889328063

C 3.832505323 0.127750177

T 0 0

C 4.771486059 0.172463352

C 2.157652474 0.115074799

T 0 0

G 2.605531295 0.247452693

G 3.088407494 0.234192037

A 0 0

C 4.604486423 0.147579693

C 1.954057279 0.164081146

A 0 0

C 1.759175008 0.197148923

T 0 0

G 1.045992924 0.430702969

T 0 0

C 1.102142192 0.015523129

A 0 0

A 0 0

G 1.39258332 0.829291191

A 0 0

G 2.106607086 0.941589531

A 0 0

C 7.977161501 0.57096248

C 1.84285475 0.8711677

T 0 0

G 1.659891599 0.54200542

C 2.104191414 0.016969286

T 0 0

G 1.309216193 1.705426357

T 0 0

G 1.3319313 0.560813179

A 0 0

G 11.76470588 0.425230333

C 12.96855569 0.213181737

C 5.992779783 0.324909747

C 4.991801785 0.182182547

A 0 0

C 2.520235467 0.018395879

T 0 0

G 2.783184118 0.622810432

C 3.022520743 1.44211774

T 0 0

T 0 0

G 2.471636953 1.235818476

C 3.924359496 1.138674258

C 1.817810458 1.000816993

A 0 0

G 2.761627907 0.49833887

C 3.478625314 1.40402347

C 1.691689575 0.888137027

A 0 0

A 0 0

C 6.465610762 0.065090041

C 1.243455497 0.283595113

T 0 0

G 1.440283625 0.310214935

T 0 0

T 0 0

A 0 0

C 4.899070084 0.09072352

C 0.759843426 1.174303477

G 48.13073125 0.658358806

G 1.75147929 0.544378698

C 0.095442615 1.026008113

G 0 0

A 0 0

A 0 0

C 1.188369153 0.278128951

T 0 0

T 0 0

C 1.264516129 0.103225806

A 0 0

T 0 0

G 3.031094852 0.156780768

T 0 0

G 2.311370882 0.18597237

T 0 0

C 4.351418342 0.082621867

T 0 0

C 7.156945618 0.028176951

C 6.054576464 0.198976691

A 0 0

A 0 0

C 14.12478336 0.202195263

C 5.631298162 0.918790753

C 3.938520653 0.160102466

T 0 0

T 0 0

G 2.895480226 0.776836158

C 5.180979418 1.312987935

C 2.838663313 0.179662235

A 0 0

G 1.367261679 2.848461831

G 6.360153257 1.340996169

T 0 0

G 1.083467095 1.886035313

A PRIMER NA NA

C PRIMER NA NA

T PRIMER NA NA

T PRIMER NA NA

G PRIMER NA NA

C PRIMER NA NA

T PRIMER NA NA

C PRIMER NA NA

T PRIMER NA NA

C PRIMER NA NA

G PRIMER NA NA

A PRIMER NA NA

C PRIMER NA NA

A PRIMER NA NA

A PRIMER NA NA

A PRIMER NA NA

C PRIMER NA NA

T PRIMER NA NA

A PRIMER NA NA

C PRIMER NA NA

C PRIMER NA NA

T PRIMER NA NA

G PRIMER NA NA

T PRIMER NA NA

A PRIMER NA NA

T PRIMER NA NA

T PRIMER NA NA

T PRIMER NA NA

C NA NA

C NA NA

A NA NA

A NA NA

C NA NA

C NA NA

C NA NA

C NA NA

T NA NA

G NA NA

C NA NA

T NA NA

C NA NA

A NA NA

A NA NA

C NA NA

T NA NA

A NA NA

C NA NA

C NA NA

T NA NA

A NA NA

G NA NA

C NA NA

C NA NA

C NA NA

C NA NA

A NA NA

G NA NA

A NA NA

A NA NA

T NA NA

T NA NA

G NA NA

C NA NA

C NA NA

A NA NA

A NA NA

G NA NA

C NA NA

A NA NA

T NA NA

T NA NA

T NA NA

T NA NA

T NA NA

G NA NA

T NA NA

T NA NA

G NA NA

G NA NA

C NA NA

A PRIMER NA NA

G PRIMER NA NA

G PRIMER NA NA

A PRIMER NA NA

C PRIMER NA NA

T PRIMER NA NA

C PRIMER NA NA

C PRIMER NA NA

T PRIMER NA NA

T PRIMER NA NA

T PRIMER NA NA

T PRIMER NA NA

A PRIMER NA NA

A PRIMER NA NA

A PRIMER NA NA

A PRIMER NA NA

C PRIMER NA NA

A PRIMER NA NA

T PRIMER NA NA

G PRIMER NA NA

G PRIMER NA NA

T PRIMER NA NA

G PRIMER NA NA

C PRIMER NA NA

T PRIMER NA NA

A PRIMER NA NA

C 4.429301533 0.511073254

A 0 0

T 0 0

G 6.693877551 0.040816327

G 8.560157791 0.315581854

C 3.763230106 0.274402195

A 0 0

C 15.07112649 0.30757401

C 5.627871363 0.45941807

C 2.3158694 0.303720577

C 0.075843762 0.379218809

G 0.075159714 0.037579857

A 0 0

A 0 0

T 0 0

T 0 0

C 2.583156405 0.141542817

C 0.492957746 0.422535211

A 0 0

G 1.955403087 0.171526587

C 3.559322034 0.372881356

A 0 0

T 0 0

T 0 0

C 0.547680412 1.030927835

T 0 0

C 0.686427457 0.655226209

A 0 0

C 0.972818312 2.17453505

A 0 0

G 3.561643836 0

T 0 0

A 0 0

A 0 0

C 1.828376402 1.755241346

A 0 0

C 1.184944238 1.138475836

A 0 0

C 1.376146789 0.131061599

T 0 0

G 7.195075029 0

C 9.893048128 0.13368984

C 7.182320442 0.076204991

C 6.335356601 0.189681335

C 3.881105642 0.189322226

A 0 0

A 0 0

T 0 0

T 0 0

C 0.363768643 0.127319025

T 0 0

C 0.288392213 0.16222062

A 0 0

G 1.350870956 0.124422325

T 0 0

G 1.55703289 0.034989503

G 3.891387063 0.069180214

C 4.386116387 0.086340874

T 0 0

T 0 0

A 0 0

C 0.22260274 0.205479452

G 0 0

A 0 0

C 0.591615957 0.185936444

A 0 0

G 2.799195441 0.033523299

C 7.787640261 0.150728521

A 0 0

T 0 0

T 0 0

G 0.372288767 0.016186468

A 0 0

T 0 0

T 0 0

T 0 0

C 0.474984167 0.031665611

T 0 0

T 0 0

G 0.611093701 0

T 0 0

T 0 0

T 0 0

G 0.561797753 0

T 0 0

G 1.014997728 0.03029844

T 0 0

T 0 0

A 0 0

A 0 0

T 0 0

G 4.877008389 0.071093417

T 0 0

G 4.768603179 0.014066676

G 7.719151312 0.111669458

C 6.955070246 0.389483934

C 3.099805393 0.347511815

A 0 0

C 2.006734007 0.026936027

A 0 0

G 2.566788895 0.183342064

G 7.187663356 0.078410873

C 9.486578056 0.16940318

C 3.109954457 0.065061809

A 0 0

G 3.27510917 0.077061392

C 4.286628279 0.166346769

C 1.593981127 0.267788829

A 0 0

C 0.125486259 0.06274313

G 0 0

G 2.179904786 0.212979203

C 2.767689418 0.187852223

T 0 0

G 0.852450796 0.062680206

A 0 0

G 9.997494362 0.025056377

C 10.94023506 0.150037509

C 5.855123233 0.175153259

C 4.290718039 0.487865899

T 0 0

A 0 0

G 1.73084159 0.112880973

G 5.804914744 0.012537613

T 0 0

G 1.052499687 0.012529758

T 0 0

G 0.388373841 0.100225507

A 0 0

A 0 0

A 0 0

A 0 0

G 2.385436284 0

C 2.946339017 0.338515547

C 1.118230934 0.05025757

A 0 0

G 1.153315783 0

G 4.811427139 0.087708307

C 4.871923656 0.037669513

T 0 0

G 0.702987698 0.025106703

A 0 0

A 0 0

G 4.124357528 0.062680206

G 7.308511972 0.050144164

G 6.724375863 0.112909296

G 11.1027663 0.037551633

T 0 0

G 7.625736862 0.01254233

T 0 0

C 11.28224393 0.062609567

C 12.59082937 0.062640942

C 11.62266984 0.050043788

C 4.83406387 0.037570445

T 0 0

G 1.654964895 0.037612839

T 0 0

C 1.140207994 0.012529758

A 0 0

G 1.642839227 0.263355907

G 5.110860579 0.062633095

C 5.310621242 0.0250501

A 0 0

T 0 0

G 0.476309852 0.188017047

A 0 0

A 0 0

A 0 0

G 2.268454694 0.075197393

C 3.721338178 0.062648791

C 0.764123763 0.012526619

A 0 0

G 0.664743509 0.075253982

A 0 0

G 0.978056426 0.163009404

A 0 0

G 2.292084168 0.075150301

G 6.295460246 0.025081515

G 5.785123967 0.062609567

A 0 0

A 0 0

C 1.526717557 0.025028157

A 0 0

G 0.402111083 0.113093742

A 0 0

A 0 0

A 0 0

T 0 0

C 1.266616504 0.087785302

C 0.564051141 0.100275758

A 0 0

G 4.511278195 0.012531328

C 7.51503006 0.03757515

C 2.104735655 0.16286645

T 0 0

C 2.929394091 0.225338007

C 1.627848735 0.22539444

A 0 0

A 0 0

G 9.658377292 0.07535795

C 11.61363067 0.363317464

C 8.51944793 0.075282309

C 7.262009281 0.275931268

C 3.834106002 0.037589275

A 0 0

C 5.118898623 0.150187735

C 2.080721985 0.263223866

A 0 0

A 0 0

G 4.531151668 0.251730648

C 5.811472323 0.06275888

T 0 0

C 4.868585732 0.100125156

C 2.128725269 0.100175307

T 0 0

G 3.039055632 0

G 6.572003016 0.163357628

C 8 0.238244514

C 2.921263791 0.125376128

T 0 0

G 0.414937759 0.377216145

A 0 0

T 0 0

G 1.204365826 0.47672814

T 0 0

G 1.458752515 0.440140845

T 0 0

C 1.315295002 0.388325191

A 0 0

G 8.287292818 0.238573581

C 10.18286573 0.388276553

C 7.416237922 0.087840381

C 6.09327984 0.488966901

C 2.313010685 0.226272784

A 0 0

G 2.871600253 0.341555977

C 3.154574132 0.492113565

T 0 0

G 1.61229374 0.352689256

C 1.909787662 0.226159065

T 0 0

C 0.82697201 0.216284987

A 0 0

C 2.11567351 0.075112669

A 0 0

T 0 0

C 5.255912902 0.187711175

C 4.323850107 0.538914651

C 2.734570998 0.200702459

A 0 0

T 0 0

G 2.331442974 0.126023945

G 3.839838323 0.618921309

C 4.931264977 0.126119309

T 0 0

C 2.160650737 0.305033045

C 1.381845842 0.342292089

A 0 0

A 0 0

A 0 0

C 3.332915675 0.075178549

A 0 0

T 0 0

G 3.740429271 0.326346178

G 5.133676415 0.502071043

G 6.301886792 0.729559748

A 0 0

G 3.355957768 0.263951735

G 6.92124105 0.351714609

C 5.032079507 0.037740596

A 0 0

C 5.063291139 0.76450683

C 1.905717151 0.66449348

A 0 0

G 1.195871098 0.465760322

G 5.402342274 0.314821811

T 0 0

C 1.045735164 0.163789845

A 0 0

G 0.400751409 0.551033187

C 0.075263422 0.338685399

G 0 0

A 0 0

G 3.026877669 0.175835217

G 8.793969849 0.351758794

C 9.613453815 0.489457831

C 3.221358737 0.162948107

A 0 0

G 0.676776538 0.275723775

A 0 0

G 1.708757382 0.087950748

T 0 0

A 0 0

T 0 0

G 3.571428571 0.15037594

T 0 0

C 1.484090052 0.150924412

A 0 0

A 0 0

C 12.53600501 0.237946149

C 5.815121829 0.288872143

C 7.701956849 0.263421977

C 4.802011314 0.175989943

T 0 0

T 0 0

C 6.761833208 0.288004007

C 2.771855011 0.175592625

T 0 0

C 2.43902439 0.037523452

A 0 0

G 10.5626727 0.326551118

G 19.68265961 0.163707342

G 20.218923 0.176144942

G 23.24792766 0.452147702

C 32.50345781 0.25147743

C 23.42002764 0.062821963

C 18.36425596 0.340780008

C 8.887484197 0.252844501

A 0 0

G 2.09656925 0.139771283

C 2.142857143 0.357142857

A 0 0

A 0 0

A 0 0

C 3.069790753 0.062648791

C 1.782799749 0.175768989

A 0 0

T 0 0

G 2.690131992 0.238843495

G 2.936746988 0.389056225

A 0 0

C 2.91760581 0.087653393

T 0 0

G 1.543868457 0.050207104

C 2.286719437 0.10051514

A 0 0

T 0 0

G 2.157010283 0.075244545

G 3.860616696 0.376034094

C 3.819575324 0.05025757

T 0 0

G 1.255335174 0.087873462

T 0 0

G 1.847197788 0.226187484

G 3.423105965 0.062924742

G 8.451946089 0.277112987

C 9.114910737 0.075433744

T 0 0

G 0.62672349 0.125344698

C 0.351229303 0.13798294

G 0.163029847 0.275896664

T 0 0

G 0.994836922 0.302228938

A 0 0

C 1.188986233 0.187734668

A 0 0

G 8.891671885 0.187852223

C 9.779061009 0.175746924

C 7.93272248 0.100414209

C 5.187319885 0.125297582

T 0 0

T 0 0

G 1.328487279 0.025065798

T 0 0

C 2.120717781 0.238423893

C 1.190327027 0.050119033

A 0 0

A 0 0

A 0 0

G 0.7023705 0.112880973

A 0 0

A 0 0

G 6.741149887 0.150640221

G 9.597290177 0.18818216

G 8.497552404 0.188276641

G 8.729461934 0.100338643

G 13.9631163 0.075272864

T 0 0

G 4.504391468 0

G 6.591478697 0.213032581

C 7.994477912 0.087851406

C 4.605923695 0.112951807

A 0 0

C 2.584692597 0.062735257

T 0 0

G 1.216606045 0.087796313

T 0 0

G 0.877192982 0.062656642

A 0 0

G 2.255073916 0.137810073

T 0 0

G 5.309956168 0.225422668

G 2.483692925 0.188158555

A 0 0

G 4.640662235 0.175592625

A 0 0

T 0 0

A 0 0

C 1.945037018 0.112937633

A 0 0

A 0 0

T 0 0

G 2.267318051 0.037579857

A 0 0

C 5.732165207 0.112640801

C 1.026668336 0.025040691

T 0 0

G 0.614805521 0.225846926

A 0 0

T 0 0

G 0.677370798 0.100351229

A 0 0

C 1.051445738 0.125172112

T 0 0

G 1.226686694 0.225309801

C 1.541160256 0.150357098

T 0 0

G 0.6655783 0.075348487

A 0 0

G 0.942566294 0.037702652

A 0 0

G 3.418054338 0.175284838

C 4.223057644 0.263157895

C 1.691729323 0.162907268

A 0 0

A 0 0

G 2.03109328 0.08776329

C 2.205790199 0.263190876

A 0 0

G 4.076257369 0.125423304

T 0 0

G 2.158634538 0.301204819

C 2.692885772 0.288076152

T 0 0

C 1.352705411 0.125250501

T 0 0

G 3.132469493 0.050320795

G 10.37463977 0.087708307

C 12.48743719 0.125628141

C 7.214680744 0.075414781

C 4.768477852 0.100389007

A 0 0

G 3.073641952 0.125454774

G 6.626430278 0.100590972

G 7.143757087 0.226785939

A 0 0

G 3.620537404 0.605525419

G 5.69668008 0.113179074

A 0 0

G 3.538712511 0.112937633

C 3.290630495 0.037678975

A 0 0

T 0 0

C 1.21706399 0.17565872

A 0 0

C 9.981214778 0.125234815

C 4.135338346 0.238095238

C 1.742946708 0.576802508

A 0 0

G 5.133032129 0.050200803

C 7.375298404 0.10051514

C 1.578947368 0.062656642

T 0 0

G 0 0

C 1.691305437 0.100225507

A 0 0

A 0 0

G 10.03890074 0.087840381

C 12.08239136 0.489826677

C 8.446285141 0.037650602

C 5.213032581 0.350877193

T 0 0

G 5.487037503 0.07550969

C 11.48055207 0.401505646

C 7.736677116 0.501567398

C 4.770273663 0.464474015

T 0 0

G 3.027627097 0.277532484

G 3.953809464 0.301242626

G 6.907812108 0.364230093

C 5.271084337 0.200803213

A 0 0

G 3.427065026 0.188300276

A 0 0

G 12.18013046 0.288509784

G 26.16388505 0.188229389

C 19.21578484 0.075405304

C 18.60670194 0.302343159

C 21.03478588 0.251161623

C 14.01129944 0.28876334

T 0 0

C 3.42019544 0.100225507

A 0 0

G 2.936746988 0.527108434

G 6.727480046 0.443430888

G 7.096124811 0.377453447

A 0 0

G 7.2198546 0.564051141

C 8.744817188 0.276416635

C 2.619375862 0.175460584

T 0 0

G 1.428571429 0.350877193

G 3.233082707 0.538847118

T 0 0

T 0 0

G 1.103171618 0.351009151

T 0 0

C 1.076076076 0.162662663

T 0 0

G 0.964670509 0.187922826

C 1.215082049 0.175372667

A 0 0

A 0 0

G 1.955622414 0.150432493

G 5.441065594 0.075395828

A 0 0

A 0 0

G 2.793786019 0.125281884

G 6.358164033 0.326059694

C 6.18336887 0.33864292

T 0 0

G 0.663661407 0.025043827

C 1.115567811 0.200551517

A 0 0

A 0 0

A 0 0

C 2.907632535 0.401052764

C 0.85384229 0.552486188

A 0 0

C 1.365059487 0.375704446

T 0 0

G 1.753726669 0.062633095

G 2.834566662 0.33864292

A 0 0

T 0 0

G 3.616887209 0.214240706

G 4.518639387 0.28869085

G 4.690831557 0.238304277

G 8.836450358 0.263587298

T 0 0

C 4.168240776 0.012592872

A 0 0

T 0 0

G 2.184831743 0.012556504

G 3.763171099 0.702458605

C 4.960967011 0.151095442

T 0 0

C 0.552208835 0.188253012

A 0 0

G 0.539184953 0.188087774

A 0 0

T 0 0

C 3.821097469 0.375845653

C 3.992968358 0.26368659

C 1.929099336 0.313165477

A 0 0

G 2.957764131 0.676776538

T 0 0

A 0 0

G 4.719467805 0.225931969

G 8.961968118 0.225931969

G 7.556169198 0.125517761

G 9.696437531 0.225790266

C 13.06311959 0.087840381

T 0 0

G 0.577091958 0.175636683

A 0 0

A 0 0

C 1.66163142 0.264350453

T 0 0

G 1.791082164 0.087675351

T 0 0

A 0 0

T 0 0

C 8.109465227 0.050213407

C 8.242378623 0.175636683

C 7.837973414 0.025081515

C 4.875908749 0.062672349

C 2.331411381 0.175482577

A 0 0

A 0 0

A 0 0

A 0 0

T 0 0

T 0 0

C 0.413689357 0.050144164

A 0 0

T 0 0

C 0.450845335 0.450845335

T 0 0

G 1.240601504 0.07518797

C 1.277715145 0.037579857

T 0 0

G 0.326469111 0.301356102

A 0 0

A 0 0

G 1.931034483 0.213166144

T 0 0

C 3.544589178 0.075150301

C 2.054880341 0.325773713

T 0 0

A 0 0

A 0 0

C 13.96543086 0.150300601

C 6.765407305 0.150621313

C 7.700037684 0.163296068

C 5.918495298 0.313479624

C 2.122049221 0.17579106

A 0 0

G 0.880503145 0.050314465

A 0 0

A 0 0

T 0 0

G 1.925254813 0.264250661

C 1.376032024 0.06254691

A 0 0

A 0 0

C 6.864587248 0.125266191

C 1.706613126 0.025097252

T 0 0

T 0 0

A 0 0

T 0 0

T 0 0

T 0 0

G 0.214024928 0.012589702

A 0 0

A 0 0

A 0 0

A 0 0

C 0.476548784 0.125407575

A 0 0

G 0.576441103 0.250626566

G 4.003514056 0.062751004

T 0 0

T 0 0

A 0 0

A 0 0

C 0.764602657 0.100275758

T 0 0

G 0.63893761 0.112753696

C 0.903387704 0.17565872

A 0 0

G 0.414677055 0.251319427

A 0 0

T 0 0

G 0.641590137 0.251603975

C 0.37697914 0.27645137

G 0 0

G 2.561526871 0.200904068

T 0 0

C 0.726908134 0.238125078

A 0 0

G 1.479067435 0.100275758

T 0 0

A 0 0

A 0 0

G 1.671484228 0.050270202

G 2.899460274 0.414208611

A 0 0

T 0 0

C 0.913870806 0.388082123

A 0 0

A 0 0

G 0.967701395 0.351891416

A 0 0

G 2.076255191 0.352334214

G 8.084999371 0.603545832

T 0 0

C 5.90966123 0.062735257

C 4.151511351 0.388812241

T 0 0

A 0 0

G 1.09145653 0.263455024

A 0 0

G 2.120185673 0.414000753

T 0 0

A 0 0

G 2.652086476 0.389643037

T 0 0

G 3.305265804 0.691215282

T 0 0

G 11.86142839 0.364001506

G 16.65198238 0.226557583

G 26.92452593 0.150696974

C 26.5560166 0.226329687

C 22.90277952 0.314425858

C 23.67388277 0.151917964

C 16.96018377 0.638080653

C 12.22378337 0.702516286

A 0 0

C 14.74513792 0.470319054

C 7.317698321 1.94796873

C 5.054105665 0.24188415

A 0 0

T 0 0

C 1.015801354 0.476548784

A 0 0

G 2.323245008 0.966972247

G 4.773869347 0.326633166

A 0 0

C 2.907488987 0.075519194

T 0 0

G 1.530740276 1.04140527

G 4.017072558 0.878734622

T 0 0

G 5.570890841 0.288582183

T 0 0

C 8.408634538 0.213353414

C 9.302033643 0.062766759

C 4.087261785 0.137913741

T 0 0

A 0 0

T 0 0

A 0 0

A 0 0

A 0 0

A 0 0

A 0 0

G 1.329153605 0.376175549

G 2.887995982 0.414364641

A 0 0

G 0.566465257 0.138469285

A 0 0

A 0 0

A 0 0

T 0 0

T 0 0

T 0 0

G 0.676522175 0.025056377

G 0.964549668 0.162846048

A 0 0

A 0 0

A 0 0

C 1.102756892 0.112781955

A 0 0

C 10.50651956 0.17552658

C 4.194925898 0.288872143

C 2.786494289 0.100414209

A 0 0

C 12.21832749 0.050075113

C 4.568846492 0.050207104

C 2.25846926 0.037641154

A 0 0

G 1.218133869 0.125580811

A 0 0

G 0.940674777 0.075253982

A 0 0

C 2.028295981 0

T 0 0

G 1.993730408 0.476489028

G 3.65119197 0.439146801

G 4.770273663 0.50213407

A 0 0

T 0 0

G 1.069047919 0.327002893

A 0 0

T 0 0

G 2.029312289 0.025053238

C 2.035687359 0.125659713

A 0 0

T 0 0

C 0.688274309 0.162683018

T 0 0

G 1.039709382 0.67643743

T 0 0

G 1.693639443 0.263455024

T 0 0

G 2.443609023 0.288220551

C 4.025583145 0.087785302

C 1.770467102 0.037669513

A 0 0

A 0 0

G 2.349246231 0.150753769

C 2.70134439 0.30154542

A 0 0

A 0 0

C 2.14905115 0.138243056

A 0 0

C 13.34169279 0.388714734

C 5.790533736 0.23917422

C 4.667253743 0.176122783

C 3.244058846 0.289199044

A 0 0

A 0 0

A 0 0

T 0 0

C 0.577236793 0.100389007

A 0 0

C 4.723127036 0.175394638

C 0.965275166 0.100288329

A 0 0

G 1.383473777 0.163501446

C 1.256913022 0.188536953

A 0 0

A 0 0

A 0 0

C 2.156739812 0.10031348

C 0.539794125 0.200853628

A 0 0

C 0.187946373 0.062648791

G 0.176034201 0.125738715

G 1.446540881 0.075471698

G 3.88909798 0.125454774

A 0 0

A 0 0

G 3.158290513 0.213059281

C 4.269972452 0.250438267

T 0 0

G 1.215538847 0.112781955

G 2.346593048 0.112937633

A 0 0

C 1.544256121 0.138104206

A 0 0

G 1.129518072 0.125502008

A 0 0

G 4.385415361 0.225535647

G 11.71894605 0.138017566

C 14.98743719 0.163316583

C 6.802806314 0.263091957

T 0 0

G 1.64325138 0.275965881

G 2.219992475 0.250846607

A 0 0

A 0 0

C 1.873327386 0.229387027

A 0 0

G 2.082288008 0.200702459

G 4.819428715 0.528501321

G 8.94023104 0.163234555

T 0 0

C 4.287863591 0.112838516

T 0 0

G 5.56251566 0.212979203

C 12.26048842 0.037570445

C 8.563189569 0.100300903

C 5.412890232 0.213997986

T 0 0

A 0 0

G 1.043522525 0.496309494

A 0 0

G 1.106821107 0.527670528

A 0 0

C 2.35386426 0.14384726

T 0 0

T 0 0

C 0.805729633 0.140682952

C 0.330915108 0.470917653

G 0.13918765 0.164494496

A 0 0

G 3.138048842 0.265721878

G 7.65646644 0.302228938

G 6.425702811 0.351405622

A 0 0

G 2.593984962 0.30075188

C 3.574564154 0.200677286

A 0 0

C 2.317134269 0.137775551

A 0 0

G 9.193528158 0.050169321

C 10.60491968 0.414156627

C 9.168550615 0.276312484

C 6.0400151 0.176167107

T 0 0

C 6.46049831 0.025040691

C 3.303716681 0.175197097

T 0 0

G 1.2783192 0.063283129

A 0 0

C 1.373871009 0.076326167

A 0 0

C 7.026541864 0.179510194

C 1.258669407 0.35961983

T 0 0

C 0.08910387 0.190936864

G 0.213836478 0.125786164

A 0 0

T 0 0

G 3.47553325 0.112923463

T 0 0

G 2.134605726 0.100452034

G 6.372855891 0.037561037

T 0 0

A 0 0

C 2.464964965 0.05005005

T 0 0

T 0 0

C 0.901803607 0.187875752

T 0 0

G 3.227021597 0.301356102

G 8.898092369 0.326305221

C 11.21284335 0.100338643

C 4.523242701 0.112767824

T 0 0

C 3.067100651 0.212819229

C 1.78347149 0.100477267

A 0 0

G 0.615037028 0.426760387

A 0 0

G 3.757044458 0.41327489

C 4.283567134 0.175350701

T 0 0

G 0.902821317 0.363636364

T 0 0

G 0.680272109 0.125976316

A 0 0

G 1.127254509 0.200400802

A 0 0

C 4.235588972 0.100250627

C 1.406681738 0.100477267

A 0 0

G 0.779678068 0.050301811

A 0 0

G 1.794678715 0.200803213

A 0 0

C 8.982561786 0.087818341

C 2.068446785 0.33847311

T 0 0

C 2.567635271 0.0501002

T 0 0

G 1.191820349 0.050181909

C 1.453451948 0.363362987

T 0 0

G 0.626880642 0.150451354

T 0 0

T 0 0

T 0 0

A 0 0

A 0 0

G 3.267810192 0.200325529

C 4.094152999 0.350569676

T 0 0

A 0 0

C 2.229179712 0.300563557

T 0 0

C 0.88883325 0.100150225

A 0 0

G 1.278195489 0.350877193

G 6.548516843 0.100553042

T 0 0

G 1.54483798 0.150715901

T 0 0

G 1.116827707 0.163132137

G 3.095626018 0.32585537

T 0 0

T 0 0

C 1.127395716 0.150319429

T 0 0

T 0 0

T 0 0

G 0.727455161 0.476608554

T 0 0

G 0.690261044 0.46435743

A 0 0

T 0 0

G 1.968158456 0.413689357

G 3.434875266 0.488905604

C 2.830661323 0.250501002

A 0 0

A 0 0

C 12.60809625 0.563980449

C 5.33785481 0.276312484

C 4.304718876 0.276104418

C 2.498430634 0.163214062

A 0 0

C 1.039709382 0.300638858

A 0 0

A 0 0

A 0 0

A 0 0

C 0.708681346 0.316375601

T 0 0

A 0 0

A 0 0

T 0 0

A 0 0

T 0 0

A 0 0

G 7.890762648 0.037754845

G 19.14439844 0.125454774

C 16.50949327 0.062869357

C 17.3405324 0.025113009

C 10.39175782 0.063597049

T 0 0

T 0 0

G 1.591478697 0.050125313

T 0 0

A 0 0

A 0 0

G 1.232371998 0.304916783

T 0 0

G 1.439939371 0.290514084

C 1.648006038 0.276764373

T 0 0

A 0 0

A 0 0

G 0.776164246 0.300450676

T 0 0

T 0 0

C 0.914672347 0.125297582

T 0 0

T 0 0

A 0 0

G 1.315789474 0.100250627

G 7.047905694 0.012540757

T 0 0

A 0 0

G 2.280130293 0.075169131

G 6.185696361 0.288582183

C 10.51771802 0

A 0 0

T 0 0

A 0 0

G 0.852771507 0.300978179

A 0 0

C 0.250532381 0.062633095

G 0 0

T 0 0

T 0 0

T 0 0

C 0.488293477 0.062601728

T 0 0

G 0.550964187 0.100175307

T 0 0

G 1.933458883 0.037664783

T 0 0

A 0 0

T 0 0

C 0.45050682 0.212739332

A 0 0

A 0 0

A 0 0

A 0 0

A 0 0

A 0 0

G 1.746231156 0.288944724

G 4.09805154 0.100565682

A 0 0

G 3.908794788 0.062640942

G 6.243718593 0.025125628

G 7.589846695 0.201055542

C 7.888456224 0.150734832

A 0 0

G 2.693560511 0.087697319

T 0 0

A 0 0

C 4.583124058 0.075339026

A 0 0

T 0 0

T 0 0

T 0 0

T 0 0

T 0 0

A 0 0

T 0 0

T 0 0

C 0.501315954 0.087730292

T 0 0

G 0.450958286 0.012526619

G 2.42127713 0.075272864

C 2.383640698 0.025090955

T 0 0

G 0.200677286 0.050169321

A 0 0

A 0 0

A 0 0

T 0 0

T 0 0

G 1.101514583 0.062586056

C 1.40175219 0.075093867

T 0 0

A 0 0

T 0 0

G 6.173303281 0.07513148

T 0 0

T 0 0

T 0 0

T 0 0

T 0 0

G 1.203761755 0.02507837

T 0 0

A 0 0

G 4.048633743 0.037603409

C 5.445420326 0.125470514

T 0 0

T 0 0

C 0.062695925 0.012539185

G 0.075206819 0.112810228

T 0 0

T 0 0

G 0.390084309 0.100666918

A 0 0

T 0 0

C 0.600750939 0.212765957

T 0 0

T 0 0

A 0 0

G 1.003764115 0.138017566

T 0 0

T 0 0

C 0.6649103 0.250909547

A 0 0

C 1.503194288 0.187899286

T 0 0

G 1.128668172 0.125407575

T 0 0

C 1.458935983 0.037731103

A 0 0

T 0 0

G 2.898005269 0.062727387

G 1.988922457 0.213997986

A 0 0

A 0 0

T 0 0

A 0 0

G 3.044256843 0.332565874

G 6.930566231 0.204970535

C 10.43188517 0.256311675

C 0.371413934 0.537909836

G 0 0

C 1.267888526 0.338940497

A 0 0

G 4.182940658 0.352778128

G 9.128735342 0.163913756

G 9.438259109 0.303643725

G 7.953970663 0.139099646

A 0 0

G 4.289476985 0.137965634

C 5.919554769 0.18972932

T 0 0

T 0 0

C 0.727272727 0.300940439

T 0 0

G 1.819550759 0.200778015

G 4.973000126 0.426974758

C 6.650801603 0.200400802

C 2.959618761 0.10032606

A 0 0

G 1.491414964 0.426118561

G 5.10114336 0.15077271

C 5.280983442 0.037631711

A 0 0

T 0 0

C 0.125897016 0.201435226

G 0 0

C 6.403508772 0.025062657

C 1.683205627 0.025122472

C 0.125770343 0.100616275

G 0 0

G 2.378255946 0.251667296

G 5.070457977 0.150981379

G 3.774534474 0.088072471

A 0 0

T 0 0

C 1.279478174 0.125439037

C 0.17579106 0.226017077

G 0.112923463 0.652446675

G 2.032112393 0.200702459

C 2.826278106 0.050244944

T 0 0

T 0 0

T 0 0

G 1.052763504 0.288256674

G 2.254509018 0.0250501

G 5.259194176 0.326346178

C 7.438120367 0.075386355

T 0 0

G 0.488966901 0.062688064

T 0 0

G 0.326633166 0.301507538

A 0 0

A 0 0

G 0.9429218 0.062861453

T 0 0

T 0 0

C 1.563868385 0.250218942

T 0 0

T 0 0

G 0 0

T 0 0

G 0.426760387 0.376553282

A 0 0

T 0 0

G 0 0

A 0 0

C 0.050119033 0.814434281

G 0 0

G 18.28011064 0.062861453

C 12.68506901 0.414052698

C 14.18876461 0.43986427

C 17.86161945 0.350965154

C 9.621174109 0.489212243

T 0 0

C 54.76190476 0.363408521

C 3.929199096 0.401707256

T 0 0

A 0 0

C 16.40784114 0.458248473

C 5.983692793 0.644397685

C 3.129108598 0.144622666

A 0 0

G 1.527112722 0.543548596

T 0 0

G 1.160443996 0.618062563

A 0 0

T 0 0

G 12.79567187 0.113236034

G 17.36729828 0.527042289

C 18.00401204 0.200601805

C 16.01053159 0.225677031

C 17.88956127 0.252143217

C 9.537095751 0.253646164

T 0 0

T 0 0

G 6.35061478 0.2281658

G 13.31398284 0.605754669

C 14.04501446 0.490380988

C 15.00063028 0.100844573

C 8.409689971 0.564829923

T 0 0

C 2.268170426 0.313283208

T 0 0

G 2.008284172 0.715451236

C 3.05764411 0.401002506

T 0 0

G 7.250376317 0.012543904

C 16.02203029 0.075103267

C 12.6503006 0.288076152

C 13.2004513 0.11282437

C 6.018054162 0.200601805

T 0 0

G 1.700037779 0.554086387

C 1.435045317 0.579053374

A 0 0

G 3.382013836 0.320266462

C 5.671028997 0.307929176

T 0 0

C 0.694087404 0.102827763

A 0 0

C 1.290888583 0.375986966

T 0 0

G 1.341861048 0.351141209

C 1.940410616 0.663495243

T 0 0

G 0.590303944 0.150715901

C 0.676691729 0.238095238

G 0 0

T 0 0

C 0.788880541 0.500876534

C 0.33804933 0.663578315

G 0 0

C 0.713481036 0.413067968

A 0 0

C 12.26202405 0.300601202

C 2.304897908 0.162846048

C 0.400751409 0.350657483

G 0.565113651 0.075348487

G 3.512293026 0.112895133

G 5.790199273 0.050131595

G 8.662404413 0.087752288

C 10.01129093 0.125454774

A 0 0

C 5.085170341 0.0250501

A 0 0

G 1.828038326 0.15128593

G 3.175403226 0

A 0 0

C 3.195296524 0.012781186

T 0 0

G 0.502189029 0.038629925

T 0 0

G 0.321626142 0.115785411

A 0 0

A 0 0

T 0 0

T 0 0

G 1.687116564 0.06390593

C 3.405612245 0.114795918

C 2.112496819 0.050903538

A 0 0

T 0 0

T 0 0

C 0.65343051 0.075395828

A 0 0

T 0 0

T 0 0

T 0 0

C 2.939337086 0.05003127

C 1.953663118 0.050093926

A 0 0

C 1.877111751 0

T 0 0

T 0 0

C 5.474818341 0.062640942

C 1.728673431 0.100212953

T 0 0

G 0.739070525 0

T 0 0

T 0 0

G 0.327044025 0.100628931

A 0 0

T 0 0

G 0.851276915 0.037556335

T 0 0

T 0 0

T 0 0

C 8.069560866 0.200175153

C 9.031692346 0.162846048

C 4.169797145 0.087653393

T 0 0

A 0 0

C 3.404255319 0.062578223

A 0 0

A 0 0

T 0 0

G 3.358447197 0.166006896

T 0 0

G 1.269035533 0.139593909

G 3.404201468 0.151860289

T 0 0

C 1.683757438 0.113938473

T 0 0

T 0 0

T 0 0

A 0 0

G 0.803011292 0.08782936

T 0 0

G 0.796057619 0.101086682

T 0 0

G 1.974433616 0.063283129

G 1.419878296 0.139452333

A 0 0

G 1.473764452 0.038114598

A 0 0

T 0 0

T 0 0

G 1.03355876 0.140359832

T 0 0

C 1.489307536 0.063645621

A 0 0

T 0 0

T 0 0

C 3.130500377 0.276590395

C 4.519702883 0.251794032

A 0 0

T 0 0

C 0.852557673 0.363590772

T 0 0

C 0.78878177 0.125203456

T 0 0

G 0.314070352 0.087939698

A 0 0

A 0 0

G 1.832789355 0.037660055

G 5.142713441 0.163460329

C 5.377287541 0.188017047

A 0 0

C 3.193087904 0.22539444

T 0 0

G 2.405412177 0.150338261

G 3.863037752 0.100338643

G 6.23432012 0.150526844

C 8.223642974 0.087752288

A 0 0

C 3.997493734 0.125313283

T 0 0

G 1.87899286 0.175372667

C 5.212379401 0.288184438

C 3.014318011 0.150715901

A 0 0

G 9.327657443 0.325528985

C 16.24874624 0.150451354

C 12.91212235 0.087752288

C 14.50842343 0.163439779

C 10.682418 0.150810607

C 5.829031852 0.692433589

A 0 0

G 5.29180909 0.164577795

C 11.12514206 0.542997853

C 6.160657812 0.392156863

C 4.647334431 0.55717361

A 0 0

G 4.886934673 0.226130653

T 0 0

C 13.30073963 0.188040617

C 17.25175527 0.238214644

C 18.1200453 0.427834403

C 13.89903059 0.566536573

C 6.628169721 0.338940497

A 0 0

C 7.963380988 0.10032606

C 0.64077416 0.340002615

G 0 0

G 3.8517346 0.184672207

T 0 0

G 2.478231748 0.44206296

G 5.03875969 0.641539695

G 7.493677625 0.559031013

C 8.047808765 0.15936255

A 0 0

C 6.480388299 0.354191263

C 3.333776066 0.531279054

A 0 0

C 49.68203498 0.463698993

G 0 0

C 1.640826873 0.245478036

A 0 0

A 0 0

G 6.084142395 0.349514563

G 10.66924067 0.463320463

G 10.4863613 0.321667524

G 8.507903868 0.488369104

G 10.01919386 0.524632118

C 22.15683766 0.08954842

T 0 0

T 0 0

G 1.862149231 0.400879348

C 4.883391316 0.56693725

C 2.056921087 0.375161708

A 0 0

C 2.703053931 0.207927225

A 0 0

T 0 0

C 0.285417748 0.38920602

A 0 0

G 0.33748702 0.54517134

A 0 0

G 0.983182406 0.155239327

T 0 0

C 1.359047373 0.220036241

A 0 0

C 2.858614473 0.605202163

A 0 0

T 0 0

T 0 0

T 0 0

C 4.042821159 0.151133501

C 1.343545957 0.439477649

T 0 0

A 0 0

C 0.213085986 0.714464778

G 0 0

C 1.516100739 0.175416614

A 0 0

T 0 0

T 0 0

G 1.504890895 0.163029847

T 0 0

G 2.658974037 0.087796313

G 3.730218538 0.037678975

A 0 0

C 16.38892369 0.225535647

C 9.45792982 0.364733996

C 4.635594291 0.265251989

T 0 0

G 1.69964485 0.114155251

C 2.680045726 0.520767179

T 0 0

G 1.003890074 0.690174426

G 3.447843531 0.890170512

T 0 0

T 0 0

A 0 0

C 21.82569497 0.212872527

C 12.13763549 1.05873456

C 12.57672554 0.212952524

C 7.671026157 0.955734406

T 0 0

C 3.204800817 0.217058223

A 0 0

C 2.804568528 0.126903553

A 0 0

C 11.38557994 0.07523511

C 2.191159804 0.755572346

C 0.642884155 0.743728728

G 0.26468364 0.138643811

C 12.24771217 0.275792905

C 7.150025088 0.150526844

C 3.720405862 0.688964049

C 0.18858436 0.125722907

G 0.175945708 0.113107955

T 0 0

G 0.785008863 0.101291466

A 0 0

C 1.86240973 0.076016724

A 0 0

G 1.671732523 0.303951368

G 3.650652808 0.240841678

G 6.910055584 0.682162708

T 0 0

C 3.42509197 0.748446023

A 0 0

T 0 0

G 0.660904931 0.076258261

A 0 0

T 0 0

G 0.166282937 0.127909951

A 0 0

T 0 0

T 0 0

T 0 0

A 0 0

A 0 0

C 6.493669299 0.33847311

C 1.591279288 0.388422503

T 0 0

A 0 0

G 1.704260652 0.112781955

T 0 0

G 2.693560511 0.16286645

G 5.255895635 0.175614651

G 5.866867243 0.062680206

G 7.817589577 0.250563768

C 12.50626566 0.563909774

T 0 0

G 1.481109577 0.213380193

G 3.137944019 0.06275888

T 0 0

G 0.983482537 0.113478754

A 0 0

G 2.194357367 0.200626959

G 6.020318575 0.363727581

C 6.677544873 0.514622819

A 0 0

C 3.154732098 0.287931898

A 0 0

G 0.742325113 0.176144942

A 0 0

G 0.789275871 0.350789276

A 0 0

G 1.740566637 0.190572989

G 5.049993672 0.265789141

C 7.067003793 0.189633375

T 0 0

A 0 0

A 0 0

G 2.247614264 0.150678051

G 3.221746791 0.088094639

A 0 0

C 6.120906801 0.088161209

C 2.389636524 0.264117721

A 0 0

A 0 0

A 0 0

C 0.989850896 0.100238065

A 0 0

G 2.166792643 0.100781053

C 4.60087995 0.201131364

C 1.467636729 0.13798294

A 0 0

G 1.29008016 0

T 0 0

C 1.075941449 0.125109471

T 0 0

G 1.189581768 0.313047834

G 2.845324643 0.225620456

G 7.366574793 0.187922826

T 0 0

G 1.495726496 0.100553042

C 1.838327877 0.402921179

A 0 0

G 0.941236327 0.165352328

T 0 0

G 1.117820892 0.089939612

G 3.360266769 0.705399513

C 5.9997425 0.437749453

T 0 0

C 0.726706462 0.35037633

A 0 0

C 1.635544108 0.309079202

A 0 0

A 0 0

C 4.931295749 0.667779633

C 2.323193428 0.064176614

A 0 0

A 0 0

A 0 0

T 0 0

T 0 0

C 3.111278384 0.175636683

C 2.9856387 0.012597632

C 1.318598518 0.33906819

A 0 0

G 1.28189016 0.075405304

C 1.43360161 0.088028169

A 0 0

C 1.01758794 0.100502513

T 0 0

T 0 0

T 0 0

G 0.764315249 0.613958151

G 1.842336132 0.238125078

G 2.772202709 0.326141495

A 0 0

A 0 0

G 2.521432173 0.15128593

C 4.351116156 0.479253374

C 1.610062893 0.113207547

A 0 0

A 0 0

G 1.617352056 0.476429288

G 6.130892678 0.26328987

T 0 0

G 2.500315696 0.454602854

G 3.282413837 0.353490721

G 5.765357503 0.138469285

C 5.237253912 0.037859667

A 0 0

G 0.317339426 0.55851739

A 0 0

T 0 0

C 0.542775911 0.155078832

A 0 0

C 1.598762249 0.335224342

T 0 0

T 0 0

G 0.977743471 0.154380548

A 0 0

G 0.843498573 0.168699715

G 5.046704722 0.480020758

T 0 0

C 1.679250195 0.11715699

A 0 0

G 1.080729167 0.325520833

G 2.449511401 0.182410423

A 0 0

G 1.113148245 0.104766894

T 0 0

T 0 0

C 1.484230056 0.238536973

A 0 0

T 0 0

G 1.272197191 0.079512324

A 0 0

C 5.732484076 0.557324841

C 3.025413473 0.228586796

A 0 0

T 0 0

C 4.765109705 0.134607619

C 1.938610662 0.296176629

T 0 0

G 1.403843533 0.177184135

G 4.160982265 0.190995907

C 6.066402514 0.273261375

C 2.794137789 0.164361046

A 0 0

A 0 0

C 5.131396957 0.290456432

A 0 0

T 0 0

G 2.080679406 0.368011323

G 3.552221908 0.438720634

T 0 0

A 0 0

A 0 0

A 0 0

A 0 0

C 7.893974508 0.362108922

C 4.666083406 0.160396617

C 5.532786885 0.731850117

C 5.06891952 0.192678227

A 0 0

T 0 0

C 6.697494033 0.089498807

C 8.287127229 0.089914581

C 3.386699507 0.015394089

T 0 0

A 0 0

C 3.436479552 0.435391074

T 0 0

A 0 0

A 0 0

A 0 0

A 0 0

A 0 0

T 0 0

A 0 0

C 0.671785029 0.063979527

A 0 0

A 0 0

A 0 0

A 0 0

C 0.562169312 0.347222222

A 0 0

A 0 0

A 0 0

T 0 0

T 0 0

A 0 0

G 1.567398119 0.049496783

C 2.217074785 0.066181337

T 0 0

G 1.362126246 0.132890365

G 2.948034644 0.066622252

G 6.038365304 0.133444537

C 8.329152032 0.100351229

A 0 0

T 0 0

G 2.670272097 0.202805476

G 3.543574093 0.1865039

T 0 0

G 1.518288475 0.189786059

G 3.162806775 0.259246457

C 5.701830863 0.034873583

A 0 0

C 5.493924987 0.281739743

A 0 0

T 0 0

G 5.336721728 0.254129606

C 9.917506874 0.018331806

C 5.15100982 0.166759311

T 0 0

A 0 0

T 0 0

A 0 0

A 0 0

T 0 0

C 3.485670023 0.038729667

C 3.627852545 0.27306417

C 1.337529504 1.101494886

A 0 0

G 2.638888889 0.654761905

C 3.758949881 0.278440732

T 0 0

A 0 0

C 2.86637061 0

T 0 0

T 0 0

G 1.644736842 0.657894737

G 0 0

G 4.111570248 1.17768595

A 0 0

G 1.810907559 0.210570646

G 4.965753425 0.663527397

C 7.878787879 0.108225108

T 0 0

A 0 0

A 0 0

G 0 0

G 6.490872211 0.405679513

T 0 0

G 4.107060452 0.41532072

G 4.371963914 0.647698358

G 5.705009276 0.185528757

A 0 0

G 1.632749645 0.42593469

G 2.945402299 0.215517241

A 0 0

T 0 0

T 0 0

G 1.296344309 0.88151413

C 2.215849844 0.31282586

T 0 0

T 0 0

G 0 0

A 0 0

A 0 0

C 7.568868681 0.026745119

C 1.719043782 0.134300295

T 0 0

G 1.487193611 1.349490498

G 2.736318408 1.630735213

G 3.849349211 0.083079479

A 0 0

G 2.45832156 0.536874823

G 5.773606371 1.336746303

C 10.06884682 0

C 50.02918856 0.75890251

G 0.234192037 0.322014052

A 0 0

G 0.722673893 0.752785306

G 3.62296592 1.535155051

T 0 0

T 0 0

G 0 0

C 1.882430647 0.066050198

A 0 0

G 1.667778519 0.800533689

T 0 0

A 0 0

A 0 0

G 1.279833967 0.172950536

C 3.258232236 0

A 0 0

G 0 0

A 0 0

A 0 0

A 0 0

T 0 0

C 0.819366853 0.074487896

T 0 0

C 1.709726444 0.721884498

A 0 0

C 7.704042715 0.266971777

C 3.519510329 0.420811018

A 0 0

C 3.6643026 0.039401103

T 0 0

G 3.259949196 0.635055038

T 0 0

A 0 0

C 12.62472885 0.21691974

T 0 0

C 7.542026352 0.454338937

C 9.26187118 0.282087447

A 0 0

G 3.854166667 0.729166667

C 11.34296415 0.588550027

C 3.220524017 0.054585153

T 0 0

G 0 0

G 3.238866397 5.802968961

G 6.83994528 4.924760602

C 11.22807018 4.350877193

A 0 0

A 0 0

C 5.130111524 0.223048327

A 0 0

G 0.974313552 7.1744907

A 0 0

G 0 0

C 1.449275362 4.013377926

G 0 0

A 0 0

G 0 0

A 0 0

C 18.18181818 0.245700246

T 0 0

C 11.12676056 8.873239437

C 30.29850746 0.149253731

A 0 0

T 0 0

C 4.139072848 4.801324503

T 0 0

C NA NA

A NA NA

A NA NA

G NA NA

C NA NA

A NA NA

A NA NA

A NA NA

C NA NA

A NA NA

A PRIMER NA NA

A PRIMER NA NA

A PRIMER NA NA

A PRIMER NA NA

A PRIMER NA NA

G PRIMER NA NA

A PRIMER NA NA

A PRIMER NA NA

A PRIMER NA NA

C PRIMER NA NA

A PRIMER NA NA

A PRIMER NA NA

A PRIMER NA NA

G PRIMER NA NA

A PRIMER NA NA

G PRIMER NA NA

C PRIMER NA NA

C PRIMER NA NA

A PRIMER NA NA

G PRIMER NA NA

G PRIMER NA NA

T PRIMER NA NA

C PRIMER NA NA

A PRIMER NA NA

C NA NA

A NA NA

C NA NA

G NA NA

G NA NA

A NA NA

C NA NA

A NA NA

A NA NA

T NA NA

G NA NA

A NA NA

C NA NA

A NA NA

G NA NA

G NA NA

G NA NA

T NA NA

G NA NA

G NA NA

A NA NA

A NA NA

C NA NA

C NA NA

A NA NA

T NA NA

T NA NA

G NA NA

C NA NA

C PRIMER NA NA

T PRIMER NA NA

T PRIMER NA NA

T PRIMER NA NA

G PRIMER NA NA

T PRIMER NA NA

C PRIMER NA NA

C PRIMER NA NA

T PRIMER NA NA

G PRIMER NA NA

T PRIMER NA NA

T PRIMER NA NA

A PRIMER NA NA

A PRIMER NA NA

T PRIMER NA NA

T PRIMER NA NA

C PRIMER NA NA

A PRIMER NA NA

G PRIMER NA NA

G PRIMER NA NA

A PRIMER NA NA

A PRIMER NA NA

A PRIMER NA NA

G PRIMER NA NA

C PRIMER NA NA

A PRIMER NA NA

T PRIMER NA NA

T 0 0

T 0 0

G 1.353965184 8.704061896

C 1.538461538 2.115384615

T 0 0

T 0 0

G 0.727272727 10.36363636

A 0 0

T 0 0

C 0.968188105 9.405255878

T 0 0

T 0 0

T 0 0

C 3.69928401 1.909307876

C 2.573099415 7.134502924

T 0 0

T 0 0

C 0.32967033 2.747252747

T 0 0

G 1.736465781 0.204290092

T 0 0

G 1.129943503 0.564971751

A 0 0

T 0 0

T 0 0

G 0 0

G 8.702175544 0.300075019

A 0 0

G 5.802292264 0.716332378

A 0 0

G 12.2705314 0.628019324

G 18.17355747 1.499318492

C 6.697171381 1.663893511

C 7.52688172 0.915969733

A 0 0

T 0 0

G 12.59018759 0.649350649

G 17.83687943 0.177304965

G 16.94200351 0.07029877

A 0 0

G 2.32172471 0.033167496

A 0 0

G 3.146853147 0.286077559

T 0 0

G 5.071119357 0.463821892

G 6.370772947 0.966183575

A 0 0

T 0 0

G 4.506627393 0

C 2.257597685 0.463096961

A 0 0

T 0 0

G 4.626532887 0.390189521

C 1.664355062 0.388349515

T 0 0

G 3.140096618 0.026838433

G 6.050955414 0.291932059

C 3.487152596 0.157315155

T 0 0

C 0.831384775 0.337750065

T 0 0

G 1.588465298 0.293255132

G 3.506208912 0.949598247

A 0 0

T 0 0

G 5.988304094 0.046783626

G 7.468243539 0.547525186

C 5.68547522 0.236000858

T 0 0

C 2.646062659 0.232853514

C 3.435919056 0.569139966

A 0 0

G 2.034883721 0.166112957

G 9.691991786 0.061601643

T 0 0

G 2.513683357 0.12162984

C 1.514547629 0.139497808

T 0 0

G 1.761942052 0.117462803

C 1.113716295 0.429855412

A 0 0

G 2.375893375 0.173845857

G 9.465972624 0.038557933

T 0 0

G 2.571321321 0.168918919

C 2.420856611 0.09310987

T 0 0

T 0 0

C 2.026656929 0.456454263

C 1.0715583 0.236106066

A 0 0

G 3.570787727 0.053830971

G 9.903811899 0.035625223

T 0 0

G 3.619989405 0.088292425

G 7.282816022 0.156060343

C 4.53917846 0.241629272

C 1.895571256 0.103394796

A 0 0

G 2.95685061 0.137527935

G 4.703266632 0.068411151

A 0 0

A 0 0

C 1.201150398 0.304517002

T 0 0

T 0 0

C 0.517615629 0.133578227

A 0 0

T 0 0

C 0.245780764 0.245780764

A 0 0

G 2.100705581 0.064143682

C 1.667201026 0.112215454

A 0 0

C 1.341328704 0.063121351

A 0 0

C 0.83617219 0.108392691

A 0 0

G 1.624883937 0.216651192

A 0 0

G 2.729224164 0.12266176

G 8.035441491 0.137488543

C 0.410771337 0.121710026

G 0 0

C 0.728486872 0.151768098

T 0 0

G 1.282825234 0.045276185

G 2.851538926 0.196137598

C 0.105389943 0.406504065

G 0.240132073 0.270148582

C 0.943396226 0.619103774

T 0 0

T 0 0

T 0 0

C 1.612903226 0.342563517

C 2.608323831 0.085518814

A 0 0

T 0 0

A 0 0

G 1.285310734 0.056497175

A 0 0

G 2.056338028 0.309859155

T 0 0

C 0.571747316 0.264956073

A 0 0

G 0.793976728 0.095824778

C 0.191152376 0.327689787

G 0 0

T 0 0

G 4.089709763 0.158311346

C 3.245488771 0.337530832

C 1.61103235 0.567083387

A 0 0

G 4.82274834 0.012526619

G 9.247176913 0.037641154

G 12.44984955 0.075225677

C 5.911823647 0.062625251

A 0 0

G 3.66419877 0.037645878

G 6.737053796 0.062845651

A 0 0

G 2.569244266 0.037598697

G 7.275231655 0.03756574

C 0.48841578 0.087664371

G 0.275723775 0.125328989

G 3.00413068 0.1126549

G 9.674185464 0.112781955

C 4.232936756 0.075140889

A 0 0

C 2.090635954 0.050075113

T 0 0

G 5.259862242 0.050093926

G 8.948489786 0.012532899

G 9.024726999 0.06275888

G 12.60188088 0.062695925

C 11.58039855 0.012532899

T 0 0

T 0 0

C 0.562781391 0.087543772

T 0 0

G 1.178979054 0.050169321

G 2.41915267 0.100275758

A 0 0

C 1.627033792 0.025031289

T 0 0

T 0 0

C 0.21279259 0.162723745

G 0.150451354 0.08776329

C 4.181795418 0.137723801

C 0.965154174 0.137879168

T 0 0

C 0.913299137 0.175153259

T 0 0

T 0 0

T 0 0

A 0 0

C 3.443095029 0.175284838

C 1.140779742 0.150432493

A 0 0

A 0 0

G 6.939390137 0.125486259

C 4.248652713 0.150394786

T 0 0

G 1.377755511 0.275551102

C 1.102618719 0.225535647

A 0 0

C 1.326990486 0.500751127

A 0 0

C 1.215691189 0.162927685

T 0 0

G 2.503128911 0.125156446

T 0 0

A 0 0

A 0 0

C 0.100288329 0.250720822

G 0.226130653 0.138190955

C 1.07918183 0.3388129

T 0 0

C 5.220330496 0.225338007

C 5.360721443 0.588677355

C 1.853707415 0.501002004

T 0 0

G 1.431801055 0.38934941

A 0 0

T 0 0

G 2.248461249 0.288908429

T 0 0

G 6.776232617 0.328697851

G 2.969421279 0.366439222

A 0 0

G 8.877448518 0.514816675

C 2.868595766 0.338218715

T 0 0

G 1.016566265 0.138052209

A 0 0

G 3.630197211 0.314030901

G 9.164149043 0.27693857

G 10 0.113493064

A 0 0

A 0 0

G 1.481950602 0.278657378

A 0 0

G 5.287616177 0.439588043

C 3.59522313 0.138277813

T 0 0

C 0.825309491 0.087532825

C 0.225422668 0.087664371

G 0.087862433 0.075310656

C 5.669586984 0.050062578

C 2.330243047 0.075169131

C 1.27755511 0.062625251

A 0 0

G 1.279959844 0.27606977

C 0.15026296 0.525920361

G 0.138347378 0.050308137

G 4.036720322 0.238933602

C 2.352201258 0.100628931

T 0 0

G 1.589319771 0.152574698

T 0 0

G 1.361669636 0.165436498

A 0 0

C 2.445172675 0.415931434

C 0.100971854 0.176700745

G 0.276868865 0.050339794

A 0 0

G 6.58043451 0.125580811

G 14.24623116 0.226130653

C 9.562052955 0.037645878

C 4.160401003 0.162907268

T 0 0

G 1.041535952 0.100389007

A 0 0

G 2.617735471 0.150300601

C 1.704474245 0.062664494

A 0 0

G 2.072343632 0.037678975

C 0.977198697 0.050112754

A 0 0

G 2.478718077 0.087631447

C 2.557031838 0.250689396

A 0 0

T 0 0

C 0.475415989 0.125109471

T 0 0

T 0 0

T 0 0

T 0 0

C 0.438431667 0.150319429

T 0 0

G 6.155005653 0.06280618

G 18.28915965 0.288908429

C 9.11937868 0.225479143

C 7.531065646 0.138069537

C 4.247055876 0.16286645

C 0.251067035 0.112980166

G 0.289928148 0.063027858

G 29.11838791 0.151133501

C 9.841827768 0.175746924

C 9.287148594 0.188253012

C 11.52784747 0.062719518

C 4.45252728 0.263388938

T 0 0

G 2.851711027 0.063371356

T 0 0

G 4.372069446 0.392852617

C 2.570888469 0.100819156

T 0 0

T 0 0

G 2.360106585 0.203019921

G 3.144894321 0.534759358

A 0 0

T 0 0

G 3.797142489 0.592096795

C 2.260177218 0.282522152

T 0 0

G 0.666666667 0.402515723

C 0.364688129 0.13832998

G 0.163583742 0.251667296

T 0 0

G 3.975690048 0.354520132

G 7.505686126 0.341167551

G 8.872810358 0.21579081

A 0 0

C 4.837471206 0.396723829

A 0 0

T 0 0

T 0 0

C 0.792207792 0.298701299

A 0 0

T 0 0

T 0 0

C 0.56693725 0.489627625

A 0 0

C 9.893455099 0.443937088

C 4.750443375 0.329364074

C 2.269877101 0.313518937

T 0 0

C 0.788880541 0.15026296

A 0 0

G 5.297514436 0.276173738

G 8.127119709 0.087928652

G 12.2734139 0.302114804

C 8.851342832 0.037826251

T 0 0

T 0 0

T 0 0

A 0 0

G 1.825557809 0.266227181

T 0 0

G 3.052456073 0.282159805

C 1.578339535 0.295136661

T 0 0

C 0.153964588 0.102643059

A 0 0

G 0.512360702 0.256180351

A 0 0

A 0 0

C 0.140718946 0.217474735

G 0 0

C 0.355284862 0.114198706

A 0 0

A 0 0

G 1.875079184 0.228050171

A 0 0

A 0 0

C 7.705912077 0.252652855

C 3.554771209 0.201689147

C 1.744696875 0.138069537

T 0 0

G 4.425222515 0.025072082

T 0 0

C 4.247055876 0.087697319

C 2.468671679 0.062656642

T 0 0

T 0 0

G 0.376884422 0.251256281

A 0 0

A 0 0

G 1.039290241 0.10139417

T 0 0

T 0 0

T 0 0

G 1.212886924 0.31585597

C 1.491971172 0.025287647

A 0 0

T 0 0

G 1.318432948 0.163234555

C 0.163029847 0.313518937

G 0 0

C 0.82696404 0.062648791

A 0 0

G 2.723735409 0.200828417

G 8.226415094 0.075471698

C 3.900843085 0.138417013

A 0 0

C 7.613323316 0.33809166

C 2.057716437 0.35131744

T 0 0

C 1.36711401 0.175592625

T 0 0

G 2.251289146 0.276694755

G 6.938519448 0.075282309

T 0 0

C 4.108731053 0.112739572

C 2.689393939 0.113636364

T 0 0

T 0 0

C 5.37796164 0.087752288

C 3.834586466 0.162907268

C 2.156739812 0.275862069

A 0 0

G 5.0821935 0.501945037

G 7.587597681 0.075623897

G 12.13114754 0.731399748

C 7.313376279 0.063155236

T 0 0

G 2.317672251 0.264516942

C 1.489710895 0.378740058

T 0 0

G 1.856730649 0.539455526

G 5.810742972 0.112951807

T 0 0

G 0 0

A 0 0

G 6.932430738 0.413689357

G 13.3902776 0.175857304

G 9.886506936 0.756620429

G 12.49686638 0.200551517

C 0.774898374 0.19054878

G 0 0

T 0 0

G 0 0

C 1.95238695 0.113364404

A 0 0

G 0 0

G 9.325969625 0.150621313

A 0 0

G 9.90447461 0.226244344

G 17.80994336 0.062932662

G 16.03619455 0.364458967

G 15.59241111 0.113079533

G 12.99321438 0.238753456

A 0 0

T 0 0

G 4.034469252 1.018409714

C 2.437137331 0.245003224

T 0 0

G 4.923076923 0.307692308

C 6.287616852 0.025611474

C 2.338359315 0.49833887

T 0 0

T 0 0

G 1.30685458 0.358744395

A 0 0

C 0.814975169 0.827709156

A 0 0

G 1.9947309 0.514364572

A 0 0

G 4.893617021 0.175219024

G 11.51674821 0.878183415

G 18.65680992 0.513720085

T 0 0

G 11.06798548 0.087642419

G 8.949416342 0.213380193

A 0 0

G 13.71932033 0.176211454

G 16.1151806 0.252589038

G 24.64443046 0.113278792

T 0 0

C 12.0633325 0.050263885

C 12.74374135 0.125801988

C 9.637188209 0.100781053

C 4.909365559 0.27693857

T 0 0

G 1.912190213 0.226443578

A 0 0

G 4.924050633 0.493670886

C 2.768957195 0.152419662

T 0 0

G 4.069621838 0.11269722

G 11.73156379 0.300488293

C 6.448375361 0.050181909

C 2.796588914 0.275896664

C 0.125659713 0.439808997

G 0 0

A 0 0

G 5.707476167 0.075263422

C 4.09033877 0.313676286

T 0 0

G 2.11567351 0.162744116

C 1.30489335 0.188205772

T 0 0

C 0.13779281 0.175372667

G 0.125344698 0.112810228

T 0 0

G 8.680381335 0.012543904

T 0 0

C 8.081694023 0.062648791

C 9.192375219 0.10032606

C 3.975420115 0.137948332

T 0 0

C 1.314142678 0.062578223

T 0 0

G 1.169811321 0.050314465

T 0 0

C 0.90146488 0.125203456

C 0.238274392 0.188111362

G 0.22564874 0.263256863

G 3.703703704 0.225988701

C 2.062114925 0.125738715

T 0 0

T 0 0

C 0.400801603 0.137775551

T 0 0

C 0.500625782 0.112640801

T 0 0

G 0.903954802 0.025109856

A 0 0

G 10.56655803 0.075206819

G 17.89883268 0.112965985

C 13.38760175 0.025046963

C 6.684223727 0.200652119

T 0 0

T 0 0

T 0 0

C 0.538779602 0.087708307

T 0 0

T 0 0

T 0 0

G 2.262159105 0.389594068

T 0 0

G 6.621434854 0.075386355

C 5.296769346 0.125219133

C 2.506265664 0.07518797

T 0 0

T 0 0

C 4.203152364 0.012509382

C 1.527673428 0.087653393

T 0 0

T 0 0

G 0.697084918 0.152091255

A 0 0

A 0 0

G 2.935273457 0.125439037

C 2.091683367 0.475951904

A 0 0

G 4.287863591 0.188064193

T 0 0

A 0 0

G 4.953599197 0.200652119

G 11.5649668 0.125297582

C 8.815426997 0.1878287

C 0.213997986 0.51611279

G 0.691997987 0.138399597

G 15.7782516 0.062711652

C 7.876884422 0.213567839

C 5.269777386 0.226386618

C 4.887830555 0.313322471

A 0 0

G 11.42390348 0.037702652

G 23.66335388 0.364825764

C 13.59016188 0.050194504

C 13.46178063 0.088150107

C 5.814100139 0.126119309

T 0 0

G 2.353092452 0.141442716

T 0 0

T 0 0

G 1.324503311 0.519413063

T 0 0

G 0.478151149 0.717226723

A 0 0

A 0 0

A 0 0

G 1.431918771 0.650872169

A 0 0

C 3.389175258 0.051546392

A 0 0

T 0 0

T 0 0

C 3.766861797 0.076355307

C 2.123601221 0.089013225

T 0 0

A 0 0

G 2.151081868 0.050613691

T 0 0

G 0.750731645 0.190863978

A 0 0

A 0 0

A 0 0

C 1.835554438 0.201156651

C 0.163296068 0.590378093

G 0.100452034 0.113008538

A 0 0

A 0 0

T 0 0

G 5.139776858 0.11282437

T 0 0

C 1.641398321 0.075178549

A 0 0

C 2.180177923 0.112767824

A 0 0

T 0 0

C 0.517415447 0.126198889

A 0 0

C 0.665661894 0.100477267

G 0.289380976 0.830397584

C 1.039188682 0.162764492

T 0 0

G 2.662313199 0.125580811

C 3.588907015 0.250972519

C 1.467636729 0.087807326

A 0 0

A 0 0

G 1.806775408 0.025094103

T 0 0

C 0.680443548 0.113407258

T 0 0

G 0.490319336 0.113150616

A 0 0

G 0.689482262 0.137896452

A 0 0

T 0 0

T 0 0

C 0.577236793 0.928598318

A 0 0

C 1.778111695 0.788880541

A 0 0

T 0 0

T 0 0

C 4.038123903 0.125407575

C 3.624733475 0.263388938

T 0 0

C 11.5640286 0.175592625

C 11.91403795 0.289053663

C 11.82890115 0.050175615

C 6.373350094 0.150848523

T 0 0

C 1.910267689 0.339323866

A 0 0

C 2.658564375 0.253196607

T 0 0

C 5.01190327 0.075178549

C 2.676551897 0.238753456

T 0 0

C 1.278996865 0.112852665

A 0 0

G 8.859329903 0.100389007

C 8.022060667 0.188017047

C 2.209389907 0.326387145

T 0 0

C 0.965638325 0.175570604

T 0 0

G 1.468912245 0.253260732

T 0 0

C 1.515151515 0.313047834

C 0.227301427 0.353579997

G 0 0

C 4.934251722 0.751408892

C 1.378618875 0.275723775

C 0.150621313 0.276139074

G 0.213702074 0.678818353

G 3.821015586 0.087983912

G 9.477780567 0.753201105

C 6.721105528 0.213567839

T 0 0

C 1.239824671 0.100187852

T 0 0

T 0 0

G 2.084379709 0.150678051

G 4.138449962 0.175570604

G 6.041954528 0.06280618

A 0 0

A 0 0

G 1.363292098 0.07573845

A 0 0

G 0.881001021 0.191521961

A 0 0

A 0 0

G 2.23533844 0.138138892

T 0 0

G 1.502629602 0.15026296

G 4.042047303 0.725816544

T 0 0

T 0 0

C 0.977443609 0.388471178

A 0 0

G 2.040050063 0.125156446

G 5.637684791 0.062640942

G 6.182593429 0.137948332

A 0 0

T 0 0

A 0 0

G 2.281845537 0.188064193

G 8.35214447 0.163029847

T 0 0

G 1.050366996 0.164515313

A 0 0

T 0 0

C 3.38770389 0.125470514

C 1.417816813 0.100376412

T 0 0

G 0.677031093 0.137913741

A 0 0

C 0.187875752 0.100200401

G 0 0

A 0 0

T 0 0

G 0.664077183 0.13782734

A 0 0

T 0 0

C 1.128243701 0.137896452

T 0 0

C 4.583020285 0.125219133

C 2.164123092 0.162621966

T 0 0

G 1.49122807 0.050125313

G 4.239839438 0.062719518

T 0 0

G 0.898961762 0.063307166

T 0 0

T 0 0

A 0 0

A 0 0

T 0 0

A 0 0

T 0 0

T 0 0

T 0 0

C 0.275378646 0.150206534

T 0 0

A 0 0

A 0 0

T 0 0

G 4.856318233 0.188229389

C 3.78256513 0.112725451

C 1.491041223 0.075178549

A 0 0

A 0 0

G 1.002004008 0.062625251

G 4.878354653 0.05016303

T 0 0

T 0 0

T 0 0

T 0 0

G 0.276035132 0.075282309

A 0 0

A 0 0

A 0 0

A 0 0

A 0 0

C 2.78544542 0.037641154

A 0 0

T 0 0

G 5.237377543 0.062798292

T 0 0

T 0 0

A 0 0

A 0 0

G 0.603166625 0.037697914

T 0 0

T 0 0

T 0 0

C 0.488293477 0.075122073

T 0 0

A 0 0

A 0 0

A 0 0

T 0 0

A 0 0

T 0 0

C 0.463601052 0.025059516

T 0 0

T 0 0

C 0.92883143 0.050207104

C 0.16286645 0.338261087

G 0 0

T 0 0

A 0 0

A 0 0

A 0 0

G 0.526183914 0.062640942

T 0 0

T 0 0

A 0 0

A 0 0

G 6.803658689 0.012529758

C 7.278877474 0.125281884

C 1.556029615 0.112937633

T 0 0

G 1.629685345 0.050144164

C 1.897700138 0.326756315

A 0 0

C 2.417940366 0.225507392

T 0 0

C 0.676691729 0.062656642

A 0 0

A 0 0

G 2.659974906 0.100376412

G 6.659964815 0.050263885

G 6.264938986 0.062900994

A 0 0

A 0 0

G 1.41959799 0.037688442

T 0 0

C 1.011673152 0.181582361

A 0 0

C 2.151495863 0.216422661

T 0 0

A 0 0

C 3.289057559 0.08855155

A 0 0

A 0 0

T 0 0

G 1.14957049 0.404244568

A 0 0

C 1.705970898 0.087807326

T 0 0

T 0 0

A 0 0

G 3.043968433 0.187899286

C 3.546810377 0.175460584

C 1.203158291 0.025065798

A 0 0

A 0 0

G 1.370207417 0.062853551

A 0 0

G 4.339102082 0.188111362

G 6.42282554 0.087983912

A 0 0

G 8.006042296 0.025176234

C 5.323433174 0.364963504

T 0 0

C 0.666415189 0.050295486

A 0 0

G 1.192269076 0.075301205

G 3.851944793 0.050188206

A 0 0

G 0.811050564 0.05069066

T 0 0

T 0 0

T 0 0

T 0 0

T 0 0

T 0 0

T 0 0

A 0 0

A 0 0

A 0 0

C 0.476250157 0.11279609

G 0.677455777 0

T 0 0

A 0 0

T 0 0

A 0 0

G 1.966432866 0.03757515

T 0 0

G 1.404036605 0.075216247

T 0 0

T 0 0

G 0.674471876 0.330872996

A 0 0

T 0 0

G 2.531328321 0

T 0 0

C 1.527673428 0.1878287

T 0 0

G 2.581777165 0.250657977

G 5.503322051 0.376081234

G 7.183222404 0.175813136

A 0 0

G 1.72175443 0.21364836

A 0 0

G 2.027460542 0.526113178

G 4.460487302 0.270723218

A 0 0

T 0 0

C 0.658319349 0.464696011

A 0 0

C 0.741485484 0.113107955

G 0 0

G 1.920662817 0.439367311

G 10.78197565 0.138069537

T 0 0

G 2.604884158 0.087664371

G 3.250094115 0.501945037

A 0 0

T 0 0

A 0 0

G 2.765208648 0.477626948

G 4.887548687 0.10051514

A 0 0

G 1.40969163 0.163624921

A 0 0

G 3.899197593 0.514042126

C 3.726082578 0.327291037

T 0 0

G 0.99009901 0.263190876

A 0 0

G 1.593675493 0.138034885

A 0 0

G 6.514371784 0.28869085

C 7.955828837 0.40155603

C 1.68004012 0.075225677

T 0 0

G 3.006765222 0.388373841

C 2.798343581 0.213326641

T 0 0

T 0 0

C 6.027122049 0.100452034

C 4.03621275 0.276625173

T 0 0

C 10.74069432 0.488783056

C 11.54714142 0.037612839

C 5.35062319 0.012589702

T 0 0

C 7.738542449 0.050087653

C 5.754942702 0.251857449

C 2.640264026 0.495049505

A 0 0

G 4.635008841 0.227330134

C 3.940388987 0.46728972

C 2.212109721 0.037921881

A 0 0

T 0 0

C 1.078099536 0.388617275

A 0 0

C 1.12626705 0.162683018

T 0 0

G 2.696939288 0.37631711

C 4.237713139 0.526579739

C 1.177797269 0.313243954

A 0 0

G 2.959247649 0.087774295

T 0 0

C 4.900977689 0.225620456

C 3.041777105 0.075728891

T 0 0

T 0 0

G 1.260239445 0.163831128

A 0 0

G 1.0164387 0.225875267

A 0 0

G 2.108169155 0.476847785

G 8.785140562 0.138052209

C 8.445225248 0.050194504

T 0 0

T 0 0

G 4.850464941 0.113093742

G 9.692404269 0.175768989

T 0 0

C 11.2406015 0.22556391

C 13.36515513 0.200979776

C 13.94817073 0.101626016

C 9.017651573 0.358147864

T 0 0

C 11.45385588 0.113780025

C 10.5076592 0.177237625

C 4.858556387 0.088798681

C 0.268199234 0.472541507

G 0.153393839 0.677489454

G 4.495238095 0.444444444

T 0 0

G 1.608646475 0.314188765

T 0 0

C 1.165121523 0.275620145

T 0 0

G 4.202207727 0.602107376

G 4.772070828 0.502323245

A 0 0

G 8.335425559 0.338940497

G 10.62437059 0.163645519

G 13.44580133 0.365101347

C 15.55751989 0.202045713

T 0 0

C 9.227683049 0.100300903

C 5.436573311 0.595615258

T 0 0

C 5.059486537 0.137758297

C 2.607169717 0.238154926

T 0 0

G 3.783783784 0.213702074

G 3.594319467 0.351891416

A 0 0

G 2.559598494 0.238393977

G 9.684316438 0.176078481

T 0 0

T 0 0

C 6.143430291 0.313440321

C 5.435190983 0.425798372

C 2.691874296 0.313008639

A 0 0

G 2.006772858 0.163050295

A 0 0

C 8.216080402 0.150753769

C 1.460502921 0.30480061

T 0 0

C 0.976342471 0.1126549

T 0 0

T 0 0

G 1.919458035 0.062727387

G 4.231541939 0.150678051

C 2.854638788 0.137723801

T 0 0

C 1.038798498 0.087609512

T 0 0

G 0.977566111 0.175460584

G 4.250783699 0.087774295

T 0 0

G 0.66424364 0.050131595

A 0 0

T 0 0

G 1.890335503 0.062593891

G 3.536493604 0.225733634

G 4.401253918 0.07523511

A 0 0

A 0 0

T 0 0

G 6.932430738 0.037608123

C 6.940261044 0.125502008

C 2.90726817 0.062656642

T 0 0

G 7.201903808 0.0250501

G 20.11292346 0.100376412

C 12.61916708 0.037631711

C 11.67168675 0.025100402

C 6.833855799 0.037617555

T 0 0

C 5.989318413 0.07629705

C 2.775655717 0.165520754

T 0 0

T 0 0

G 0.938252821 0.11411183

A 0 0

C 0.700700701 0.012512513

T 0 0

T 0 0

T 0 0

G 1.291212235 0.075216247

G 3.799373041 0.263322884

T 0 0

C 1.152448954 0.075159714

A 0 0

C 0.988364819 0.08757663

A 0 0

G 0.901577761 0.03756574

A 0 0

G 1.967665121 0.050131595

C 2.229738194 0.150319429

A 0 0

G 2.129258517 0.137775551

G 5.533944033 0.125486259

A 0 0

A 0 0

G 7.891430008 0.075395828

C 8.025621703 0.200954534

C 2.872260015 0.088183422

T 0 0

T 0 0

A 0 0

G 4.027098231 0.18818216

G 8.732358871 0.189012097

G 8.778337531 0.138539043

A 0 0

G 4.797884397 0.062964362

C 3.885714286 0.101587302

A 0 0

T 0 0

G 1.730407524 0.275862069

C 0.200727638 0.18818216

G 0.364230093 0.364230093

G 2.882928052 0.225620456

T 0 0

T 0 0

G 1.905239408 0.02506894

T 0 0

G 2.003506136 0.050087653

C 1.676466909 0.012510947

A 0 0

C 8.23117338 0.012509382

C 2.157184307 0.126151129

T 0 0

T 0 0

C 0.513591382 0.062633095

A 0 0

C 0.938321031 0.025021894

T 0 0

T 0 0

T 0 0

T 0 0

G 0.915934755 0.150564617

C 0.964670509 0.062640942

A 0 0

G 1.039318808 0.26296018

G 7.921539042 0.050295486

T 0 0

G 0.40175769 0.087884495

A 0 0

A 0 0

A 0 0

A 0 0

G 1.315624608 0.025059516

T 0 0

A 0 0

A 0 0

C 0.726270974 0.087653393

T 0 0

G 0.978179082 0.05016303

A 0 0

G 3.108548508 0.087741289

G 6.412347848 0.087840381

A 0 0

C 5.05075824 0.062664494

C 1.707041546 0.163173089

A 0 0

G 0.701666458 0.062648791

A 0 0

A 0 0

A 0 0

G 0.941383206 0.263587298

A 0 0

T 0 0

G 1.505079644 0.087796313

A 0 0

C 2.508466073 0.062711652

A 0 0

T 0 0

G 2.588264857 0.075386355

C 1.705115346 0.112838516

A 0 0

G 3.591160221 0.075339026

C 3.137156481 0.125486259

T 0 0

G 1.647177166 0.062869357

G 4.943538269 0.363864492

C 4.212637914 0.025075226

T 0 0

C 0.977933801 0.012537613

A 0 0

G 3.588006524 0.175636683

G 6.628097095 0.050308137

G 10.88760372 0.264018104

C 9.153865525 0.113321581

T 0 0

G 0.39205767 0.07588213

A 0 0

G 1.479327349 0.328739411

T 0 0

T 0 0

C 0.929765046 0.125643925

C 0.476967491 0.5773817

G 0.226443578 0.327085168

G 1.347607053 0.440806045

A 0 0

G 7.687484324 0.037622272

C 7.051120625 0.453286326

C 2.311557789 0.27638191

T 0 0

G 3.204807211 0.463194792

G 5.945810336 0.100351229

G 5.051520483 0.18848957

G 10.52565392 0.037726358

T 0 0

T 0 0

T 0 0

G 2.58891542 0.175945708

T 0 0

C 4.102371095 0.18818216

C 3.031821599 0.526183914

T 0 0

G 7.636272293 0.113036925

G 13.80737397 0.213192877

G 17.41870431 0.239475674

G 19.72575167 0.176122783

C 26.08805349 0.063075565

C 15.51854656 0.164017159

T 0 0

C 7.484954865 0.17552658

C 4.091653028 0.037769105

T 0 0

T 0 0

A 0 0

T 0 0

C 1.287664177 0.193149627

T 0 0

C 1.859635715 0.089160616

C 1.774397972 0.114068441

A 0 0

A 0 0

T 0 0

G 3.879472693 0.225988701

G 5.078566939 0.263984915

G 6.949855473 0.201080809

A 0 0

A 0 0

G 0.608828006 0.050735667

A 0 0

A 0 0

A 0 0

A 0 0

C 2.02122284 0.012632643

C 0.138941518 0.063155236

G 0.088116818 0.03776435

T 0 0

C 0.681216097 0.012615113

T 0 0

T 0 0

G 0.413430218 0.062640942

G 2.255639098 0.137844612

T 0 0

T 0 0

T 0 0

T 0 0

G 0.45129748 0.075216247

C 0.175306787 0.200350614

G 0.11299435 0.11299435

G 1.733232856 0.175835217

C 0.12561236 0.100489888

G 0.289308176 0.13836478

G 3.910965795 0.075452716

G 11.21388748 0.076026356

G 9.9381235 0.037883571

A 0 0

C 12.23596084 0.077279753

C 4.425684318 0.127909951

T 0 0

C 1.490216405 0.207334456

A 0 0

G 2.841572596 0.103801739

G 7.625470108 0.337180651

C 4.368297149 0.143866074

A 0 0

C 0.208441897 0.143303804

G 0.522807476 0.16991243

G 1.839140008 0.712342961

G 9.003880983 0.258732212

T 0 0

G 3.18388564 0.285899935

C 1.827437671 0.078318757

T 0 0

T 0 0

G 1.734178853 0.064708166

G 2.780292254 0.155179103

A 0 0

G 0.848342469 0.117462803

A 0 0

A 0 0

A 0 0

G 3.22496749 0.455136541

C 2.97311272 0.555842813

T 0 0

G 1.600813111 0.06352433

T 0 0

T 0 0

G 2.766497462 0.114213198

A 0 0

C 9.711779449 0.200501253

C 6.303258145 0.125313283

C 4.845593774 0.150640221

T 0 0

C 5.782951558 0.1126549

C 4.406779661 0.100439422

T 0 0

T 0 0

C 0.864986837 0.025072082

T 0 0

T 0 0

T 0 0

G 1.31826742 0.11299435

G 1.490480962 0.187875752

G 4.915360502 0.225705329

T 0 0

T 0 0

T 0 0

T 0 0

C 0.727820304 0.351361526

C 0.338176353 0.325651303

G 0.150621313 0.050207104

C 0.578252671 0.163419233

A 0 0

C 6.948791787 0.212845875

C 1.56641604 0.250626566

T 0 0

A 0 0

A 0 0

T 0 0

G 1.993480441 0

A 0 0

G 6.182728411 0.037546934

G 11.79055751 0.050226017

G 12.18013046 0.062719518

G 11.25313283 0.07518797

A 0 0

G 2.882566738 0.037598697

A 0 0

G 2.248042435 0.151553423

G 10.30953885 0.088439672

T 0 0

G 1.941003703 0.076618567

A 0 0

C 4.693049214 0.03805175

A 0 0

T 0 0

T 0 0

T 0 0

G 1.055939661 0.037712131

T 0 0

C 0.613958151 0.050119033

A 0 0

G 1.514392991 0.763454318

G 7.440401506 0.100376412

T 0 0

G 0.555485419 0.164120692

C 0.250501002 0.125250501

G 0.062774639 0.075329567

G 4.357625845 0.11269722

C 3.278482603 0.113051124

T 0 0

C 0.625625626 0.037537538

A 0 0

G 1.91537306 0.062593891

G 7.811717576 0.062593891

C 4.711189074 0.714196216

A 0 0

T 0 0

G 0.826446281 0.012521913

A 0 0

A 0 0

G 3.982467126 0.551033187

C 4.20115375 0.802608478

T 0 0

A 0 0

A 0 0

G 2.303455183 0.025037556

C 1.917053001 0.050119033

A 0 0

G 2.015270998 0.187758167

G 4.2318768 0.050081382

A 0 0

C 2.080721985 0.062672349

A 0 0

G 4.266532815 0.163132137

C 4.72887768 0.075662043

T 0 0

C 0.57745418 0.087873462

A 0 0

G 0.922649141 0.113751264

A 0 0

G 1.001502253 0.375563345

C 0.527704485 0.10051514

G 0.07535795 0.100477267

T 0 0

C 1.802478408 0.225309801

C 1.251094708 0.462905042

A 0 0

G 0.67576023 0.112626705

A 0 0

T 0 0

C 0.830502076 0.125833648

A 0 0

T 0 0

G 10.08508509 0

T 0 0

A 0 0

C 12.61904762 0.025062657

C 3.178791305 0.226159065

T 0 0

G 1.307692308 0.461538462

A 0 0

G 1.275280175 0.656962514

A 0 0

G 4.345596718 0.153826433

C 4.519119351 0.06437492

A 0 0

T 0 0

G 5.357366379 0.250344223

T 0 0

C 5.792377131 0.17552658

C 3.121082978 0.350965154

T 0 0

C 0.113164843 0.352068402

G 0.163234555 0.050226017

G 3.033721951 0.300864987

C 2.521324636 0.451580532

T 0 0

G 1.991482966 0.125250501

T 0 0

A 0 0

A 0 0

C 1.978957916 0.100200401

A 0 0

G 3.099899598 0.15060241

G 5.865360462 0.100477267

A 0 0

G 3.466465712 0.062798292

T 0 0

G 2.598385469 0.07568113

C 2.078580482 0.076045627

A 0 0

T 0 0

C 0.889389954 0.225479143

T 0 0

G 1.164392137 0.287967948

T 0 0

G 1.104140527 0.112923463

A 0 0

G 7.739511584 0.150281778

G 15.82706767 0.338345865

C 13.17771084 0.062751004

C 6.662484316 0.100376412

C 0.150829563 0.188536953

G 0.050774308 0.203097233

A 0 0

T 0 0

A 0 0

C 2.530840646 0.076306753

A 0 0

C 0.113036925 0.376789751

G 0.150867488 0.075433744

A 0 0

A 0 0

C 3.501506024 0.125502008

C 1.179866951 0.238483745

A 0 0

C 1.830262003 0.037608123

A 0 0

C 0.564475665 0.13798294

G 0.376884422 0.025125628

T 0 0

G 1.680141486 0.075795856

G 4.153032973 0.113264536

C 4.043330394 0.088172314

T 0 0

A 0 0

T 0 0

T 0 0

C 0.731491991 0

A 0 0

T 0 0

T 0 0

C 0.439919558 0.037707391

A 0 0

A 0 0

A 0 0

T 0 0

T 0 0

A 0 0

C 1.740981964 0

A 0 0

T 0 0

G 6.518741381 0.025072082

G 2.480270575 0.125266191

A 0 0

G 5.614167295 0.025119317

T 0 0

T 0 0

C 0.188347564 0.062782521

G 0.225535647 0.062648791

A 0 0

A 0 0

G 1.705970898 0.062719518

T 0 0

C 1.764485046 0.050056313

C 1.202856785 0.012529758

A 0 0

G 1.540967176 0.037584565

T 0 0

T 0 0

C 4.096204434 0.037579857

C 1.624685139 0.088161209

T 0 0

T 0 0

G 1.431082099 0.037660055

G 3.333333333 0.012531328

T 0 0

C 0.11302273 0.025116162

G 0.087730292 0.187993483

C 0.565042692 0.238573581

A 0 0

G 2.381250783 0.213059281

T 0 0

A 0 0

G 2.056168506 0.188064193

T 0 0

C 1.19077463 0.050137879

A 0 0

C 2.548970367 0.037669513

A 0 0

T 0 0

T 0 0

T 0 0

C 0.350789276 0.313204711

A 0 0

G 0.802507837 0.07523511

A 0 0

G 5.01945037 0.100389007

C 4.522676021 0.388373841

C 1.852318548 0.163810484

A 0 0

C 2.517219787 0.300563557

A 0 0

C 2.007024586 0.112895133

A 0 0

G 2.159447583 0.225988701

G 9.378147029 0.100704935

T 0 0

G 3.044082354 0.265251989

G 7.336343115 0.15048909

T 0 0

A 0 0

C 3.532677265 0.151400454

T 0 0

G 2.287880769 0.379134527

G 3.941663382 0.709499409

A 0 0

T 0 0

G 3.155792277 0.772303595

G 5.046604527 0.639147803

C 3.429410195 0.133440085

A 0 0

C 2.192806525 0.066853857

T 0 0

G 1.198721364 0.612679808

T 0 0

C 1.49887253 0.013264359

A 0 0

G 4.527661795 0.352296451

G 7.721452835 0.274366344

G 10.59377452 0.340047083

C 5.463836478 0.117924528

A 0 0

G 1.513954713 0.236966825

A 0 0

A 0 0

C 6.736306415 0.227668408

C 1.832247557 0.244299674

T 0 0

T 0 0

C 3.697300173 0.212794255

C 2.496348427 0.491302616

A 0 0

G 2.323111642 0.132749237

T 0 0

G 1.202726179 0.50781772

A 0 0

C 1.388332666 0.333733814

T 0 0

G 0.695373094 0.401176785

A 0 0

G 4.497494065 0.527565286

C 3.926701571 0.981675393

C 1.995305164 0.299947835

A 0 0

C 1.974538841 0.610548194

T 0 0

C 0.634879502 0.272091215

T 0 0

G 0.747037609 0.231839258

A 0 0

G 5.705209657 0.228716645

C 4.232469994 0.606443462

T 0 0

G 10.93416928 0.037617555

C 12.82919488 0.313518937

C 11.59764794 0.250218942

C 12.62550201 0.276104418

C 9.96481528 0.477506911

C 7.707936508 0.203174603

A 0 0

T 0 0

G 5.163077698 0.163707342

C 4.509853461 0.429509853

C 1.81678608 0.166325486

A 0 0

G 6.596070426 0.306200561

C 7.113975234 0.328531716

C 3.734544537 0.13878375

T 0 0

C 9.250219051 0.21279259

C 10.42136945 0.213192877

C 8.790656788 0.100464649

C 3.749686481 0.388763481

T 0 0

G 2.356647763 0.478890989

G 2.512943553 0.277812855

A 0 0

A 0 0

T 0 0

C 0.398765114 0.115770517

T 0 0

G 0.720535255 0.308800823

T 0 0

T 0 0

A 0 0

G 2.193318031 0.344299923

C 1.441142711 0.446371636

A 0 0

A 0 0

T 0 0

G 5.463092364 0.203277855

C 2.247048369 0.177732639

T 0 0

G 0.277742709 0.45448807

A 0 0

T 0 0

T 0 0

C 0.438871473 0.213166144

T 0 0

C 0.538172716 0.13767209

A 0 0

G 5.827067669 0.162907268

G 16.25955155 0.150319429

C 11.09163746 0.150225338

C 7.22528851 0.163070748

C 3.396841314 0.188017047

A 0 0

G 7.507886435 0.151419558

C 5.80693816 0.188536953

C 2.236180905 0.075376884

C 1.136650669 0.20207123

A 0 0

G 1.527599487 0.26957638

A 0 0

C 4.199106573 0.357370772

C 1.008811135 0.204316179

T 0 0

G 0.659681801 0.28456862

A 0 0

C 0.607078274 0.064582795

A 0 0

G 0.674798858 0.519076045

A 0 0

T 0 0

C 0.29719602 0.090450963

A 0 0

G 0.564899217 0.141224804

A 0 0

C 1.546523517 0.460122699

T 0 0

C 0.662167325 0.904113078

T 0 0

C 0.931989924 0.125944584

T 0 0

G 1.114729459 0.313126253

G 3.14221332 0.050075113

C 1.355761988 0.138086869

G 0 0

G 5.904475367 0.125360411

G 11.26902276 0.201232549

G 11.60512434 0.301431801

G 19.55756662 0.113122172

T 0 0

G 10.53690431 0.075443229

G 14.84463455 0.30192477

G 17.2197084 0.201106083

G 18.43161857 0.225846926

G 17.22271182 0.100717613

G 14.07193159 0.251509054

A 0 0

C 9.238500193 0.038654813

A 0 0

G 4.033796675 0.081766149

C 3.024523161 0.163487738

C 0.991052994 0.151410874

G 0.989010989 0.054945055

C 9.453896817 0.068605928

C 5.706856439 0.191597099

C 3.281826884 0.095719951

C 1.217676837 0.068408811

G 0.629188893 0.533442757

T 0 0

C 3.27005681 0.540390744

T 0 0

C 3.005844698 0.584469802

C 3.668704836 0.041689828

A 0 0

C 0.92682252 0.027666344

A 0 0

G 4.390655784 0.661412891

C 3.397782201 0.099516633

T 0 0

C 1.301487414 0.028604119

T 0 0

C 5.263906672 0.099587424

C 3.957446809 0.09929078

C 1.698994761 0.226532635

A 0 0

G 1.03958986 0.655084022

G 2.425721257 0.114827042

A 0 0

T 0 0

T 0 0

C 0.17644464 0.10292604

G 0 0

G 0.839346193 0.176704462

A 0 0

T 0 0

G 1.942179392 0.044477391

C 0.904641851 0.266943497

A 0 0

G 3.031654035 0.163471541

C 2.694655352 0.148875986

T 0 0

G 1.776915037 0.119456473

G 2.511961722 0.044856459

A 0 0

G 1.693121693 0.030234316

T 0 0

T 0 0

T 0 0

G 2.576762845 0.108008023

G 4.972205065 0.092649784

G 7.909779082 0.108141511

G 8.425925926 0.030864198

A 0 0

C 7.58396533 0.092864882

C 3.439449688 0.063989762

A 0 0

C 1.965601966 0.065520066

T 0 0

C 1.029729281 0.016608537

T 0 0

G 0.89602705 0.473372781

T 0 0

G 0.5092514 0.594126634

A 0 0

G 1.718271521 0.578428037

T 0 0

C 1.41955836 0.035050824

A 0 0

T 0 0

C 0.703605981 0.158311346

T 0 0

G 2.163504167 0.620677425

G 2.662406816 0.124245651

A 0 0

G 1.822079314 0.107181136

A 0 0

G 2.754469172 0.656694637

C 2.905342081 0.07497657

A 0 0

C 0.681302044 0.227100681

G 0.531813865 0.189933523

C 0.932622764 0.114198706

T 0 0

G 0.442392768 0.288517023

A 0 0

T 0 0

A 0 0

A 0 0

C 1.524628616 0.117279124

T 0 0

C 0.352802822 0.078400627

A 0 0

G 0.712025316 0.336234177

T 0 0

T 0 0

T 0 0

C 0.738375574 0.079824386

A 0 0

T 0 0

T 0 0

T 0 0

C 0.422535211 0.040241449

T 0 0

G 1.434343434 0.060606061

T 0 0

G 2.538071066 0.060913706

G 5.525086329 0.589071704

C 5.707297187 0.081532817

C 2.00122524 0

A 0 0

G 0.601909506 0.269821503

A 0 0

T 0 0

C 0.634249471 0.105708245

A 0 0

A 0 0

G 2.068671358 0.149285562

T 0 0

A 0 0

G 1.800043375 0.195185426

T 0 0

T 0 0

T 0 0

C 0.485758446 0

A 0 0

T 0 0

T 0 0

T 0 0

C 0.650515926 0.157021086

T 0 0

C 2.219705549 0.090600227

T 0 0

C 10.29680365 0.068493151

C 9.168003667 0.343800138

C 3.661906955 0.092123445

T 0 0

G 1.444547996 0.139794967

T 0 0

T 0 0

T 0 0

G 1.067527309 0.521350546

T 0 0

T 0 0

G 0.533943555 0.686498856

A 0 0

T 0 0

G 0.571577033 0.727461678

A 0 0

C 0.287281274 0.052232959

G 0.607983082 0.634417129

G 0.92936803 0.424853956

A 0 0

G 2.055164954 0.081124932

C 3.171591217 0.054215234

A 0 0

G 4.656930284 0.523560209

G 10.30470914 0.110803324

G 11.96438509 0.250417362

G 10.1152009 0.196684462

A 0 0

G 4.621347645 0.14907573

G 6.68767507 0.700280112

G 4.347826087 1.436672968

A 0 0

G 0.829531315 2.82040647

T 0 0
