# Supplementary material for: Bisulfite probing reveals DNA structural intricacies
Source: Nucleic Acids Res. 2023 Mar 7;51(7):3261–9. doi: 10.1093/nar/gkad115 (PMC10123088; doi:10.1093/nar/gkad115)
Supplement: gkad115_Supplemental_Files [file gkad115_supplemental_files.zip › Table S3.docx]

**Table S3:** Bisfulite reactivity (percentage of reads modified C->T) for the central (fourth) nucleotides of all possible heptamers with a G or C at their fourth position, based on the data from Dumelie and Jaffrey (2017). Heptamers modified in more than 40% of reads were excluded in view of the possibility of heterozygosity.

**Heptamer %Reads modified C->T**

| GGGGCCC | 39.82 |
| --- | --- |
| GGGCCCC | 39.64 |
| GAACATA | 37.47 |
| TATGTTC | 37.42 |
| AAAGCTT | 37.28 |
| AAGCTTT | 36.67 |
| CCCCCCC | 33.38 |
| GGGGGGG | 33.30 |
| AAACATA | 32.35 |
| TATGTTT | 32.09 |
| AGGGCCC | 31.62 |
| GGGCCCT | 31.32 |
| TAACATA | 30.62 |
| TATGTTA | 30.54 |
| CGGGCCC | 30.32 |
| GGGCCTG | 30.12 |
| CAGGCCC | 29.95 |
| TGGGCCC | 29.92 |
| GGGCCCA | 29.80 |
| GGGCCCG | 29.07 |
| GGGGGCC | 28.02 |
| GGCCCCC | 27.99 |
| AAGGCCC | 27.89 |
| GGGCCTT | 27.83 |
| GGGCCTA | 26.42 |
| GGGCCTC | 26.38 |
| GAGGCCC | 26.38 |
| TAGGCCC | 26.24 |
| GGGGGGC | 25.89 |
| CAGCATA | 25.73 |
| GCCCCCC | 25.66 |
| TATGCTG | 25.50 |
| GGACATA | 25.39 |
| GAGCATA | 25.33 |
| TATGCTC | 25.27 |
| TATGTCC | 25.27 |
| AGGCCCC | 25.18 |
| GGGGCCT | 25.01 |
| TATGTCT | 24.38 |
| AGACATA | 24.27 |
| CCGGCCC | 23.95 |
| GGGCCGG | 23.92 |
| TATGCTT | 23.77 |
| AAGCATA | 23.66 |
| GGCCCCT | 23.12 |
| AGGGGCC | 23.11 |
| GGGCCAG | 23.03 |
| CTGGCCC | 23.01 |
| GTACATA | 22.89 |
| TATGTAC | 22.82 |
| CAGGGCC | 22.81 |
| GGCCCTG | 22.79 |
| CGGGGGG | 22.77 |
| CACCATA | 22.73 |
| TATGGTG | 22.63 |
| CCCCCCG | 22.52 |
| GGCCCCG | 22.41 |
| GACCATA | 22.30 |
| CGGGGCC | 22.17 |
| TATGGTC | 22.05 |
| CGGGCCT | 21.88 |
| GGCCCCA | 21.87 |
| TGGGGCC | 21.82 |
| CGACATA | 21.76 |
| AGGCCCT | 21.66 |
| AGGCCTG | 21.52 |
| TATGTCG | 21.45 |
| TGGCCCC | 21.43 |
| AGGCCCG | 21.40 |
| GGGCCAC | 21.25 |
| AGGGCCT | 21.22 |
| CAGGCCT | 21.19 |
| AGGCCCA | 21.14 |
| GGGGCCA | 21.05 |
| CCCCCCA | 21.02 |
| TGGGCCT | 20.99 |
| GGGCCAT | 20.80 |
| TGGGGGG | 20.79 |
| GTGGCCC | 20.73 |
| CCCCCCT | 20.71 |
| AGGGGGG | 20.70 |
| GGGCCGA | 20.69 |
| GGGCCGT | 20.64 |
| ATGGCCC | 20.62 |
| CCGCCCC | 20.51 |
| TAGCATA | 20.45 |
| ACGGCCC | 20.45 |
| CGGCCCC | 20.34 |
| AGGCCTC | 20.32 |
| GCGGCCC | 20.22 |
| GGGGCCG | 20.21 |
| TATGCTA | 20.21 |
| AATGTTG | 20.19 |
| CAACATT | 20.15 |
| GGGGCGG | 20.10 |
| GGGGTCC | 20.09 |
| AGGCCTT | 20.08 |
| GCCCCCG | 20.04 |
| CGCCCCC | 20.04 |
| GGACCCC | 20.01 |
| GGGCCGC | 19.98 |
| GAGGCCT | 19.87 |
| AAGGCCT | 19.87 |
| TATGGTT | 19.74 |
| AGGGGGC | 19.71 |
| GGGGGCG | 19.68 |
| GAACATT | 19.59 |
| CGGGGGC | 19.58 |
| GAGGGCC | 19.55 |
| TCGGCCC | 19.51 |
| AATGTTC | 19.51 |
| CGGGCCG | 19.49 |
| AACCATA | 19.46 |
| GGCCCTC | 19.39 |
| CGGCCCG | 19.37 |
| GCCCCCT | 19.36 |
| ACCCCCC | 19.25 |
| GGCCCTA | 19.15 |
| GGCCCTT | 19.01 |
| TTGGCCC | 18.90 |
| GGGGGGT | 18.84 |
| GCCCCCA | 18.83 |
| TGGGGGC | 18.83 |
| TAGGGCC | 18.81 |
| GGGGGGA | 18.81 |
| GGGCCAA | 18.74 |
| TCCCCCC | 18.73 |
| GGAGCCC | 18.72 |
| AAGGGCC | 18.71 |
| GCGCCCC | 18.66 |
| GAGCCCC | 18.65 |
| TAGGCCT | 18.60 |
| GGGCTCC | 18.59 |
| GGGGCTC | 18.58 |
| AGGCCTA | 18.35 |
| TATGCCC | 18.33 |
| GGGCATA | 18.27 |
| CAGGGGG | 18.27 |
| TATGTCA | 18.27 |
| TGACATA | 18.26 |
| GGTCCCC | 18.23 |
| CGGGGCG | 18.21 |
| CCCCCTG | 18.20 |
| GCCCCTG | 18.20 |
| CAGGGGC | 18.08 |
| CCACCCC | 18.07 |
| GGGGTGG | 18.06 |
| GGGGCGC | 18.04 |
| TATGGTA | 17.93 |
| GGGGACC | 17.92 |
| CGCCCCG | 17.75 |
| GTCCCCC | 17.75 |
| AGGCCGG | 17.73 |
| TACCATA | 17.66 |
| GGGGGAC | 17.57 |
| GAGGGGG | 17.54 |
| CCGGCCT | 17.48 |
| CCCCCTC | 17.45 |
| GGCCCAG | 17.33 |
| GGGGCAC | 17.30 |
| CTGGGCC | 17.19 |
| GCCCCTC | 17.15 |
| GTGCCCC | 17.11 |
| GAGGGGC | 17.03 |
| GTACCCC | 16.96 |
| GGGGTAC | 16.83 |
| GGGGGCT | 16.82 |
| TGCCCCC | 16.78 |
| GGGGGCA | 16.78 |
| GGGCTGG | 16.74 |
| CCAGCCC | 16.69 |
| CTCCATA | 16.68 |
| GGGCATG | 16.65 |
| CATGTAC | 16.65 |
| TGGCCCT | 16.64 |
| CATGCCC | 16.62 |
| AGGGCCA | 16.58 |
| GTACATG | 16.58 |
| AGCCCCC | 16.57 |
| CTCCCCC | 16.55 |
| TATGGAG | 16.52 |
| GGGGGAG | 16.47 |
| AATGTTT | 16.28 |
| CCCCCAC | 16.25 |
| AAACATT | 16.24 |
| AGGCATA | 16.22 |
| GGGCTTG | 16.18 |
| GGCCCAC | 16.15 |
| GTGGGGG | 16.09 |
| GTGGGCC | 16.07 |
| CAGCCCC | 16.07 |
| GGGGCTG | 16.02 |
| TATGCCT | 15.99 |
| TGGCCCA | 15.98 |
| CGGGCCA | 15.98 |
| CCGGGCC | 15.95 |
| TGGGCCA | 15.93 |
| CTGGCCT | 15.92 |
| AGGCCAG | 15.90 |
| GCACCCC | 15.88 |
| CCCCCAG | 15.86 |
| CAAGCCC | 15.85 |
| GGACCCT | 15.84 |
| AGGGTCC | 15.77 |
| CTGGGGG | 15.64 |
| GGACCCG | 15.55 |
| GCCCCTT | 15.52 |
| GGGGTGC | 15.51 |
| CGGGTCC | 15.47 |
| AAGGGGC | 15.43 |
| GGCCCGG | 15.38 |
| CACCCCC | 15.38 |
| GGGGGTG | 15.33 |
| GGGCTAG | 15.28 |
| GGGCTCG | 15.15 |
| CATGGAG | 15.13 |
| CTCCATT | 15.11 |
| CGAGCCC | 15.10 |
| CCGGGGG | 15.08 |
| CTCCATG | 15.08 |
| AGGCCGA | 15.08 |
| TGGGTCC | 15.08 |
| GGCCCAT | 15.06 |
| CAGGCCA | 15.03 |
| GGGGCAG | 15.02 |
| CTGCCCC | 15.02 |
| GACCCCC | 14.99 |
| CAGCCTG | 14.98 |
| TGGCCCG | 14.97 |
| GGGCAGG | 14.97 |
| CAGGCTG | 14.97 |
| AATGGAG | 14.96 |
| CCCCCGG | 14.95 |
| TGGCCTG | 14.94 |
| AGGCCAT | 14.93 |
| GGGGGTC | 14.93 |
| GCCCCAG | 14.90 |
| CGGCCCT | 14.89 |
| CCTGCCC | 14.88 |
| CTAGCCC | 14.83 |
| GCGGGGG | 14.83 |
| CCCCCTT | 14.82 |
| AAGGGGG | 14.81 |
| GGACCCA | 14.79 |
| CTGGGGC | 14.79 |
| TCGGCCT | 14.77 |
| ATGGGCC | 14.77 |
| AGGGCTC | 14.68 |
| GCCCCGG | 14.68 |
| CCCCCTA | 14.68 |
| CCGGGGC | 14.68 |
| ATGGCCT | 14.66 |
| GCCCCTA | 14.64 |
| TAGGGGC | 14.63 |
| GAGCCCT | 14.60 |
| AGGCCAC | 14.55 |
| CCCCTCC | 14.51 |
| CAGGCTC | 14.49 |
| GTGGCCT | 14.49 |
| GGGGCTT | 14.44 |
| GAGCCTG | 14.44 |
| CCCCCGC | 14.40 |
| AAGGCCA | 14.38 |
| TGGCCTT | 14.34 |
| GCCCCAC | 14.34 |
| TATGGCC | 14.33 |
| TAGGGGG | 14.33 |
| TATGGGC | 14.32 |
| GAGCCCA | 14.30 |
| AGGCCGT | 14.28 |
| GGGGCAT | 14.27 |
| GGAGGGG | 14.24 |
| TGGGCTC | 14.21 |
| GGGGTCG | 14.20 |
| GGGGCGT | 14.18 |
| CGGCCTG | 14.16 |
| CGGGCTC | 14.14 |
| GCCCATA | 14.13 |
| GTGGGGC | 14.12 |
| AGGGCCG | 14.10 |
| ATGCCCC | 14.09 |
| CGGCCCA | 14.05 |
| AAGCCCC | 14.05 |
| GGGCTGC | 14.04 |
| CCTCCCC | 14.04 |
| TAGCCCC | 14.04 |
| GGCCATA | 14.01 |
| TAACATT | 14.00 |
| GCAGCCC | 13.98 |
| GTACATT | 13.93 |
| GGGGCTA | 13.92 |
| AGGCCAA | 13.91 |
| CAACATG | 13.91 |
| GGGCTAC | 13.89 |
| GGGGAGG | 13.88 |
| AGGGACC | 13.86 |
| GAGCCCG | 13.84 |
| AATGTTA | 13.82 |
| GTACCCT | 13.78 |
| GAGGCTC | 13.77 |
| ACGGCCT | 13.76 |
| CGGGGGT | 13.76 |
| CGACCCC | 13.75 |
| AATGTAC | 13.73 |
| GAGCCTC | 13.71 |
| GGTCCCT | 13.69 |
| TTGGCCT | 13.68 |
| CTCCATC | 13.66 |
| CATGTTG | 13.65 |
| GTAGCCC | 13.65 |
| GATGGAG | 13.63 |
| GGGCTCA | 13.63 |
| CAGGCCG | 13.61 |
| GTCCCCT | 13.60 |
| TAAGCCC | 13.59 |
| TGGGCCG | 13.58 |
| GGGCTTA | 13.53 |
| GTCCCCA | 13.49 |
| TGAGCCC | 13.49 |
| AGGGGGT | 13.49 |
| TTGGGCC | 13.48 |
| GGCCCAA | 13.48 |
| AGGGGAC | 13.47 |
| GTACCCG | 13.46 |
| AGGGTAC | 13.44 |
| AGGGGCG | 13.42 |
| TGGGGAC | 13.41 |
| GGGCTTC | 13.41 |
| GGTGCCC | 13.39 |
| GGGCACC | 13.38 |
| ACGCCCC | 13.37 |
| AGGGGGA | 13.36 |
| ACCCCCG | 13.35 |
| AGGCTCC | 13.35 |
| ACCCCCT | 13.35 |
| GGAGCCT | 13.34 |
| GAAGCCC | 13.34 |
| TATGCCA | 13.33 |
| TCCCCCT | 13.31 |
| GGCCTGG | 13.30 |
| TGGCCTC | 13.28 |
| CCAGGCC | 13.27 |
| GGGGTAG | 13.26 |
| GCCCCGC | 13.24 |
| TGGCATA | 13.23 |
| GAGGCCA | 13.22 |
| GTCCCCG | 13.21 |
| CTACCCC | 13.21 |
| ATGGGGG | 13.20 |
| CGTGTAC | 13.19 |
| CGCGCGG | 13.18 |
| GGGGTGT | 13.17 |
| ACACCCC | 13.15 |
| CGGGCTG | 13.13 |
| AAGCCTG | 13.13 |
| AGGCCGC | 13.13 |
| CGGGGAC | 13.12 |
| GCGGCCT | 13.11 |
| CCCCCAT | 13.11 |
| CAAGCCT | 13.11 |
| GAGCCTT | 13.11 |
| TAGGCCA | 13.11 |
| AGGCTTG | 13.09 |
| AAGGCTC | 13.09 |
| CGCCCCT | 13.07 |
| TGGGGGT | 13.06 |
| CAGGCTT | 13.06 |
| GCGGGCC | 13.06 |
| CGGCCTT | 13.04 |
| GGCCATG | 13.03 |
| GGCCCGC | 13.01 |
| TGGCCTA | 12.99 |
| CAGCCCG | 12.99 |
| GGGCTGT | 12.98 |
| ACCCCCA | 12.97 |
| CATGGCC | 12.94 |
| ACAGCCC | 12.93 |
| GCGGCCG | 12.91 |
| TCCCCCG | 12.87 |
| CCGCGCG | 12.86 |
| AGGGGCA | 12.86 |
| AAGGCCG | 12.84 |
| GCGGGGC | 12.83 |
| GGACATT | 12.83 |
| AATGTCC | 12.80 |
| TCCCCCA | 12.80 |
| CGGGTAC | 12.80 |
| AGACCCC | 12.74 |
| CCGGCCG | 12.74 |
| GGGGTCT | 12.72 |
| GGGCACG | 12.72 |
| TGGGGGA | 12.71 |
| CGGCCGG | 12.71 |
| CGCCGCG | 12.71 |
| TGCCCCT | 12.69 |
| CGGGGGA | 12.68 |
| GGTCCCA | 12.65 |
| GCCCCAT | 12.63 |
| CGTGCCC | 12.60 |
| GGACCTG | 12.57 |
| AGCCCCG | 12.55 |
| TATGCCG | 12.53 |
| ATGGGGC | 12.53 |
| GTACACG | 12.52 |
| TCACCCC | 12.52 |
| TGGGACC | 12.52 |
| AGCCCCT | 12.51 |
| TCAGCCC | 12.47 |
| CAGCCCT | 12.47 |
| AGGGGCT | 12.46 |
| GGGCTGA | 12.44 |
| GGGGTGA | 12.43 |
| GTACAGG | 12.42 |
| CCGGCCA | 12.39 |
| CCTGTAC | 12.37 |
| GTACCCA | 12.37 |
| GGGGCGA | 12.36 |
| AGGGCTG | 12.36 |
| GTGCCCT | 12.35 |
| CGGCATA | 12.35 |
| CAGGTCC | 12.34 |
| CGACCCG | 12.34 |
| ATGCCTG | 12.34 |
| TGGCCGG | 12.32 |
| TCGCCCC | 12.31 |
| AGGGCAC | 12.31 |
| CGGCCGC | 12.30 |
| ACGGGCC | 12.29 |
| CCACCCT | 12.28 |
| CGGGCTT | 12.28 |
| TGTCCCC | 12.27 |
| CGGGTCG | 12.27 |
| CCGCGGG | 12.25 |
| CAGGCAT | 12.24 |
| CCCCTGG | 12.23 |
| GGGGACG | 12.23 |
| TGGGTAC | 12.20 |
| AGGGTGG | 12.19 |
| GGGGACA | 12.18 |
| TCGGGCC | 12.15 |
| GAGCCTA | 12.13 |
| GGACATG | 12.11 |
| TATGTAT | 12.10 |
| CTCCCCT | 12.09 |
| TATGTGC | 12.09 |
| CGAGCCT | 12.09 |
| CACCATT | 12.07 |
| CGGGGCT | 12.06 |
| CATGTCC | 12.06 |
| CCCCATA | 12.06 |
| GCACATA | 12.06 |
| CCAGGGG | 12.06 |
| AATGCTG | 12.05 |
| AGAGCCC | 12.05 |
| ATACATA | 12.04 |
| TAGGCTC | 12.02 |
| GGGCTCT | 12.00 |
| GGGCATT | 12.00 |
| GTGCCCA | 12.00 |
| GGGGCAA | 11.99 |
| AATGGTG | 11.99 |
| AATGTCT | 11.97 |
| GTCCCTG | 11.96 |
| TCCCCTC | 11.96 |
| CAGCCCA | 11.95 |
| TTGCCCC | 11.92 |
| AAGCCCG | 11.90 |
| CAGCATT | 11.90 |
| TGGGCTG | 11.90 |
| AGGGTGC | 11.90 |
| TAGGCCG | 11.89 |
| TGACCCC | 11.89 |
| CTCCACT | 11.89 |
| CCCGGGG | 11.89 |
| CCCCAGG | 11.89 |
| GCACCCT | 11.88 |
| AATGCCC | 11.88 |
| CGCGGGG | 11.88 |
| GGGGTCA | 11.87 |
| CAGGGAC | 11.87 |
| AGGCTCG | 11.86 |
| AAGCCTC | 11.86 |
| TATGGGG | 11.86 |
| GGACCGG | 11.85 |
| CATGTTC | 11.85 |
| CCCGCGC | 11.85 |
| GAACATG | 11.84 |
| AGGGGAG | 11.84 |
| GGGCTAT | 11.82 |
| GTGCCTG | 11.82 |
| AAGCCCA | 11.81 |
| CCTGGGG | 11.80 |
| CGTCCCC | 11.78 |
| AGACATT | 11.76 |
| GAGGCTT | 11.75 |
| AGCCCCA | 11.75 |
| TGGGCAC | 11.75 |
| GAGGGGA | 11.73 |
| GTACACA | 11.73 |
| AAGCCTA | 11.72 |
| TAGGCTT | 11.71 |
| CCGGTCC | 11.71 |
| GGCCCGA | 11.70 |
| CGGGGCA | 11.68 |
| ATAGCCC | 11.67 |
| TGCCCCA | 11.67 |
| TGGGGCT | 11.67 |
| CCCCACC | 11.64 |
| TCCCCTG | 11.62 |
| GGGCACA | 11.62 |
| AGTGGAG | 11.62 |
| CCCCGCG | 11.61 |
| CAGGCAC | 11.61 |
| AAAGCCC | 11.61 |
| TGGGCTT | 11.60 |
| TGTGCCC | 11.59 |
| TGTGTAC | 11.59 |
| ATGCCCG | 11.59 |
| CCACCCA | 11.58 |
| TGGGTGG | 11.58 |
| TGGGGCA | 11.58 |
| GAGCATT | 11.58 |
| GCCCTGG | 11.57 |
| GGCCCGT | 11.56 |
| GACCATT | 11.56 |
| CGGGCTA | 11.55 |
| CAGGGGA | 11.54 |
| CCGCCGC | 11.53 |
| CCAGGGC | 11.53 |
| GCCCTCC | 11.52 |
| AGGCATG | 11.50 |
| TGCCCCG | 11.49 |
| CAGGCTA | 11.49 |
| GGTGGGG | 11.48 |
| AGGGGTC | 11.47 |
| GGGCTTT | 11.46 |
| CGCCCCA | 11.46 |
| CGCGGCG | 11.46 |
| TAGCCTG | 11.45 |
| AAGCCTT | 11.44 |
| AAGGCTT | 11.44 |
| TAGCCCG | 11.44 |
| AATGCTC | 11.42 |
| AAGCCCT | 11.42 |
| GTACATC | 11.41 |
| CATGCCT | 11.39 |
| ACCCCTG | 11.39 |
| GGAGGGC | 11.39 |
| CGGCCTA | 11.38 |
| GCGGCGG | 11.38 |
| GACCCCT | 11.38 |
| GAGGCCG | 11.38 |
| GTGCATA | 11.37 |
| AGGGCTT | 11.36 |
| TACCCCC | 11.36 |
| AATGGTC | 11.35 |
| GATGCCC | 11.35 |
| CAGCCTA | 11.35 |
| CAGGGGT | 11.34 |
| TAGGCTG | 11.33 |
| GGGGGTA | 11.33 |
| GCACCCG | 11.32 |
| AGGCTTA | 11.31 |
| AGGGCGC | 11.31 |
| GTCCCTC | 11.31 |
| TAAGCCT | 11.31 |
| CCCCGGG | 11.28 |
| CGGCCTC | 11.28 |
| GGGCATC | 11.27 |
| GAGGGAC | 11.26 |
| GATGTAC | 11.25 |
| CCACCCG | 11.24 |
| CGGGTGG | 11.23 |
| CCCGCGG | 11.22 |
| CTCCCTC | 11.20 |
| TATGCAC | 11.19 |
| AATGCTT | 11.18 |
| ATGCCCA | 11.18 |
| CGGGTGC | 11.18 |
| ATCCCCC | 11.17 |
| CTACATA | 11.12 |
| GGCCTTG | 11.12 |
| TGGGGCG | 11.11 |
| GAGGGAG | 11.11 |
| GGCCTGT | 11.09 |
| AGGCTCA | 11.09 |
| GTGCCCG | 11.08 |
| TGAGCCT | 11.08 |
| CGGGACC | 11.07 |
| GGACCTT | 11.04 |
| GGGGGAT | 11.03 |
| CTTGCCC | 11.02 |
| AAGGTCC | 11.02 |
| CCCCCAA | 11.01 |
| GGGCTAA | 11.00 |
| ACACATA | 10.99 |
| TGGGCAT | 10.99 |
| CAAGGCC | 10.98 |
| AGGGCGG | 10.98 |
| TTAGCCC | 10.97 |
| GCAGCCT | 10.97 |
| GCACCCA | 10.96 |
| CGGCGCG | 10.94 |
| GGGCAAG | 10.94 |
| TATGTAG | 10.94 |
| GGACCTC | 10.93 |
| CGGGCAT | 10.93 |
| AGGCTGC | 10.92 |
| GAAGCCT | 10.92 |
| ATACCCC | 10.92 |
| TATGTGT | 10.91 |
| AGGGGTG | 10.91 |
| CTCCCCG | 10.90 |
| GGGGTTG | 10.90 |
| TGGGTGC | 10.89 |
| TTGGGGC | 10.87 |
| CCGCCCT | 10.87 |
| CAACCCC | 10.87 |
| AGCCCTG | 10.86 |
| CACCCCT | 10.86 |
| CGGGCAC | 10.85 |
| CAGGGCT | 10.85 |
| AAGCATT | 10.84 |
| TTGGGGG | 10.83 |
| ACAGGCC | 10.83 |
| AGGCTGA | 10.82 |
| ACCCCTC | 10.80 |
| CGGGGAG | 10.80 |
| CCGGCGG | 10.78 |
| GGGGTAT | 10.77 |
| GAGGTCC | 10.77 |
| CAGGTAC | 10.75 |
| GGCCATT | 10.74 |
| GCCCCAA | 10.73 |
| TCAGCCT | 10.73 |
| AGGGCAG | 10.73 |
| GAACCCC | 10.73 |
| GGGGTAA | 10.73 |
| AGGCTTC | 10.72 |
| AGTGCCC | 10.71 |
| TAGCCCT | 10.70 |
| GGTCCCG | 10.70 |
| GACCCCG | 10.69 |
| GAGGGGT | 10.69 |
| GGGGTTC | 10.69 |
| TTACCCC | 10.67 |
| CAGCCTT | 10.67 |
| AGGGCTA | 10.67 |
| CCCCCGT | 10.66 |
| CAGCCTC | 10.65 |
| GGGCACT | 10.65 |
| GCGCCCT | 10.64 |
| GGACCTA | 10.63 |
| CGGCCAG | 10.63 |
| CGCGCCG | 10.63 |
| AAGGCTG | 10.62 |
| AATGGCC | 10.62 |
| CCTCCCT | 10.62 |
| ATGCCCT | 10.62 |
| CGGGCGG | 10.60 |
| TGGCCAG | 10.60 |
| ACGGGGG | 10.60 |
| GTACCTG | 10.60 |
| AGGCTAG | 10.59 |
| CAGGGCA | 10.59 |
| CTGCCCT | 10.59 |
| TTCCCCC | 10.58 |
| TGCCCTC | 10.58 |
| TGCCCTG | 10.58 |
| GAGGCTG | 10.57 |
| CCGCCCG | 10.55 |
| TAGGTCC | 10.54 |
| GTCCATA | 10.53 |
| GGGGGAA | 10.52 |
| TGGCCAC | 10.52 |
| CTGGCCA | 10.52 |
| GAGGGCA | 10.51 |
| TGGGGTC | 10.50 |
| CTCCCCA | 10.50 |
| TAGCCCA | 10.49 |
| TGGGGAG | 10.48 |
| AGGCTAC | 10.46 |
| AGGGAGG | 10.45 |
| AGGGCAT | 10.44 |
| GCGGCGC | 10.44 |
| CGGGGTC | 10.44 |
| TGGGCTA | 10.44 |
| GTGGCCA | 10.43 |
| GCTCCCC | 10.43 |
| CTAGCCT | 10.43 |
| GACCCCA | 10.41 |
| CACCCCA | 10.41 |
| CCGGCGC | 10.40 |
| AGACCCT | 10.40 |
| GTAGCCT | 10.39 |
| GTACACC | 10.37 |
| AGGGTCT | 10.36 |
| ACACCCT | 10.35 |
| GCGCCGC | 10.34 |
| AATGTCG | 10.33 |
| CCCCATG | 10.32 |
| GCGCGGG | 10.31 |
| GGGCAGC | 10.30 |
| AGGGTCG | 10.29 |
| TAGGCAT | 10.28 |
| CATGGGG | 10.28 |
| AGCCATA | 10.27 |
| TCCCTCC | 10.27 |
| AGGGTGT | 10.26 |
| GGCCTCG | 10.26 |
| GCTGCCC | 10.26 |
| CGGGGTG | 10.25 |
| TATGGAC | 10.24 |
| TATGGCT | 10.21 |
| GAGCCGG | 10.21 |
| GGGGAGC | 10.19 |
| GGAGGGA | 10.18 |
| CCGCCGG | 10.18 |
| GCCCATG | 10.17 |
| CGGGTAG | 10.17 |
| GCAGGCC | 10.16 |
| TAGGCTA | 10.14 |
| GCGCGCG | 10.14 |
| GGCCTGC | 10.13 |
| TAGCCTA | 10.13 |
| CGCGCAC | 10.13 |
| ATGCCTA | 10.13 |
| GTGCCTC | 10.13 |
| CCAGCCT | 10.13 |
| CTACCCG | 10.12 |
| GGTGTAC | 10.12 |
| TGGCCAT | 10.11 |
| TGGGGTG | 10.10 |
| AGGGTAG | 10.10 |
| CATGGGC | 10.10 |
| TAGCCTT | 10.09 |
| GAGGCAC | 10.09 |
| AAGGCTA | 10.07 |
| TAACCCC | 10.07 |
| AATGCCT | 10.07 |
| CTACCCT | 10.06 |
| AGGCTGG | 10.05 |
| GCGCCTG | 10.05 |
| GGGGTTA | 10.05 |
| CACCCCG | 10.05 |
| GGACCAG | 10.04 |
| GTAGGCC | 10.03 |
| CTGGCCG | 10.02 |
| TCGCCCG | 10.02 |
| GCCCGCG | 10.01 |
| GTGCCTA | 10.00 |
| AGTCCCC | 9.99 |
| AGGCTGT | 9.98 |
| GGCCTCA | 9.97 |
| ACGGGGC | 9.96 |
| TGGCCAA | 9.95 |
| CTCCCTG | 9.95 |
| CGGGCAG | 9.94 |
| ACAGCCT | 9.94 |
| GCACCTG | 9.94 |
| CGACATT | 9.93 |
| GAGGGCG | 9.93 |
| GGACAGG | 9.91 |
| CGAGGCC | 9.91 |
| AGGCATT | 9.90 |
| GGCCAGG | 9.90 |
| TTGGCCA | 9.89 |
| AGCCCTC | 9.89 |
| GGGGACT | 9.89 |
| TAGGCAC | 9.89 |
| CCTGTCC | 9.88 |
| CGTCCCG | 9.88 |
| GAGGGCT | 9.88 |
| GGCCTCC | 9.87 |
| TAGCATT | 9.87 |
| ATGGCCA | 9.87 |
| CCTGGCC | 9.86 |
| CTAGGCC | 9.86 |
| CTGGTCC | 9.85 |
| CGTGGAG | 9.85 |
| CGGGTCT | 9.84 |
| AGACCCG | 9.83 |
| CTGCCCG | 9.83 |
| CAGGTGC | 9.82 |
| GGAGGCC | 9.82 |
| CTCCACC | 9.82 |
| AATGCTA | 9.82 |
| GGCCTAG | 9.81 |
| GACCCTG | 9.81 |
| GGTGGAG | 9.80 |
| TAACATG | 9.80 |
| GCGCCGG | 9.79 |
| ATGCATA | 9.79 |
| TATGCAT | 9.78 |
| CGGGACG | 9.76 |
| CAGGGAG | 9.76 |
| GAGCATG | 9.76 |
| GCCCCGT | 9.75 |
| CGCCCTC | 9.75 |
| CCCCCGA | 9.75 |
| GGCGGGG | 9.75 |
| GCAGGGG | 9.74 |
| TCCCCTT | 9.74 |
| CGGCCGT | 9.74 |
| CGCCCTG | 9.74 |
| GGCCTAC | 9.73 |
| GGTGGCC | 9.73 |
| AACCATT | 9.72 |
| TTACATA | 9.72 |
| GTCCCAG | 9.72 |
| TAGCCTC | 9.71 |
| ACCCCTT | 9.71 |
| CGTGGCC | 9.71 |
| TGAGGCC | 9.70 |
| CCCCGCC | 9.69 |
| CAGGCGC | 9.69 |
| CGACCCT | 9.69 |
| CATGTTA | 9.69 |
| CATGCTC | 9.67 |
| CCGGCTC | 9.66 |
| TATGTAA | 9.66 |
| GAGCCAG | 9.65 |
| CAGGGTC | 9.65 |
| AAGGGGA | 9.64 |
| CTCCACG | 9.64 |
| TGACCCG | 9.62 |
| CAGGGCG | 9.62 |
| AAGGGCT | 9.62 |
| GGCCACC | 9.62 |
| CTGGGAC | 9.61 |
| TAGGGAC | 9.60 |
| AGCCCTT | 9.60 |
| TCGGGGG | 9.59 |
| CCCCTGC | 9.59 |
| TGGGCAG | 9.59 |
| GTCCCTT | 9.58 |
| AAGGGAC | 9.58 |
| TCACCCT | 9.58 |
| GGGCAGT | 9.57 |
| CTGGCTC | 9.57 |
| GGCCACG | 9.56 |
| GAGGCTA | 9.56 |
| CAACCCT | 9.55 |
| ACTGCCC | 9.54 |
| AAGGGGT | 9.53 |
| CCGCCCA | 9.53 |
| AATGGTT | 9.52 |
| ACACCCG | 9.52 |
| AGGGTTG | 9.52 |
| CGGGTTC | 9.52 |
| CGCGGGC | 9.51 |
| TGGCTCC | 9.50 |
| CTGCCCA | 9.50 |
| GGAGCCA | 9.47 |
| GCGCCCA | 9.46 |
| GGGCGGG | 9.46 |
| GAACCCG | 9.46 |
| AATGGTA | 9.45 |
| TAGGGGA | 9.45 |
| GGTCCTG | 9.45 |
| TGGGCGG | 9.44 |
| AGGGTGA | 9.43 |
| AAGGTAC | 9.43 |
| CGGGTGT | 9.42 |
| GTCCCTA | 9.42 |
| CGGCGGC | 9.41 |
| ATACCCG | 9.37 |
| TCCCCTA | 9.36 |
| TCCCCAC | 9.36 |
| GTGCGCG | 9.36 |
| GGCCTTA | 9.36 |
| TGGGTAG | 9.35 |
| CGGGTGA | 9.35 |
| TAGGTAC | 9.35 |
| AAGGCAC | 9.35 |
| TAAGGCC | 9.34 |
| GTGCCTT | 9.33 |
| CGGGCGC | 9.33 |
| AGGGCGT | 9.33 |
| ACCCCAC | 9.33 |
| CCCGCCC | 9.33 |
| AACCCCC | 9.32 |
| CGCCATA | 9.32 |
| GGACCAC | 9.31 |
| CGCGGCC | 9.31 |
| CTACCCA | 9.31 |
| CGCGCGC | 9.31 |
| ACGCCCT | 9.31 |
| TGGCCGT | 9.31 |
| GGGGGTT | 9.31 |
| AGACCCA | 9.31 |
| GTACCTT | 9.30 |
| TAGGGGT | 9.30 |
| CAGGACC | 9.29 |
| AGGGTCA | 9.29 |
| GTGGGGA | 9.28 |
| GCGCCCG | 9.28 |
| TCACCCG | 9.28 |
| GAACCCT | 9.28 |
| GTGGTCC | 9.28 |
| TATGCAG | 9.27 |
| CTGGGGT | 9.27 |
| ACACCCA | 9.24 |
| GTACCTA | 9.24 |
| GTGGGGT | 9.24 |
| ACCCCTA | 9.24 |
| ACGGCCA | 9.23 |
| GGCCATC | 9.23 |
| TATGGCG | 9.23 |
| ACCCCAG | 9.23 |
| TCCCCAG | 9.22 |
| AGGGTTC | 9.21 |
| CATGCTG | 9.21 |
| CGGCTCC | 9.20 |
| TGGGTGT | 9.19 |
| CGGCCAC | 9.18 |
| CGACCCA | 9.17 |
| ATAGCCT | 9.16 |
| TACCATT | 9.15 |
| GAGGGTC | 9.15 |
| CTGGGGA | 9.15 |
| ATGGTCC | 9.15 |
| CGGGTCA | 9.15 |
| TGGGTCT | 9.15 |
| GCCGCCG | 9.14 |
| GACCCTC | 9.14 |
| CCCCTCG | 9.12 |
| TCGGCCA | 9.12 |
| GGCCTTC | 9.12 |
| TCTGCCC | 9.12 |
| GCGCGGC | 9.11 |
| GGCCGCG | 9.11 |
| TGACCCT | 9.11 |
| TGGGCGC | 9.10 |
| CAGCATG | 9.10 |
| AGGCTAT | 9.09 |
| ATGCCTC | 9.09 |
| GGGCAGA | 9.09 |
| CAGGCGT | 9.09 |
| GTACCGG | 9.08 |
| ACGGTCC | 9.08 |
| ACGGCCG | 9.06 |
| GGACCAT | 9.06 |
| CCACCTG | 9.05 |
| CAGGCAG | 9.03 |
| ACGCCTG | 9.03 |
| TGGGTCG | 9.03 |
| CTGCCTG | 9.03 |
| CGGGTAT | 9.02 |
| GGGCAAC | 9.02 |
| CATGTTT | 9.02 |
| CTGCATA | 9.01 |
| AAACATG | 9.00 |
| TGGCCGC | 8.99 |
| AGGCTAA | 8.98 |
| GATGGCC | 8.98 |
| CTCCCTT | 8.97 |
| GAAGGCC | 8.97 |
| GTGGCCG | 8.96 |
| AGGCAGG | 8.95 |
| AGCCCTA | 8.95 |
| GAGGCGC | 8.95 |
| GAGGCAT | 8.94 |
| CGTGCCT | 8.94 |
| GCCCTGC | 8.94 |
| CAGGTGG | 8.94 |
| TTAGCCT | 8.94 |
| AAGCATG | 8.93 |
| CGCCGCC | 8.93 |
| GCAGGGC | 8.93 |
| AAGGGAG | 8.93 |
| GTACCTC | 8.92 |
| AGGCTTT | 8.91 |
| GCGGCCA | 8.91 |
| AGGCTCT | 8.89 |
| GAGGTAC | 8.89 |
| GGCCAAG | 8.89 |
| GGAGCCG | 8.88 |
| TCGGGGC | 8.88 |
| CGGCGGG | 8.87 |
| CGGCCAT | 8.86 |
| CCAGCCA | 8.85 |
| GTACAAG | 8.85 |
| TTACCCG | 8.85 |
| AGGCACG | 8.85 |
| CCTGCCT | 8.85 |
| TATGGCA | 8.85 |
| TGCCATA | 8.85 |
| GTTGCCC | 8.85 |
| TGGCTGG | 8.83 |
| TAGGGCT | 8.83 |
| CTGCCTC | 8.83 |
| AAAGCCT | 8.83 |
| AGAGCCT | 8.82 |
| TATGGAT | 8.81 |
| TGCCCTA | 8.80 |
| TATGGGT | 8.80 |
| ATCCATA | 8.80 |
| CATGCTT | 8.79 |
| AAGGCAT | 8.79 |
| GGACACG | 8.79 |
| CTTGGCC | 8.79 |
| TGCCCTT | 8.78 |
| ACCCCGG | 8.78 |
| CTTGTAC | 8.78 |
| GGCGGCG | 8.77 |
| CCGGGGT | 8.77 |
| CGAGGGG | 8.77 |
| GTCCCAC | 8.76 |
| GGCCTGA | 8.76 |
| TAGGGCA | 8.76 |
| ATGGCTC | 8.76 |
| ATACCCT | 8.74 |
| CACCCTC | 8.74 |
| CAGCCGG | 8.73 |
| AAGGGCA | 8.72 |
| ATGCCTT | 8.72 |
| TAGCCGG | 8.71 |
| CCCCTAG | 8.70 |
| TGGCCGA | 8.69 |
| CCACCTC | 8.69 |
| CGGGTAA | 8.69 |
| TGAGCCA | 8.68 |
| CCCGGGC | 8.68 |
| ATGGCCG | 8.67 |
| CCGGGGA | 8.66 |
| ACCCATA | 8.66 |
| CGTGTCC | 8.66 |
| GAGGTGG | 8.65 |
| AGGCACC | 8.64 |
| GAGGCAG | 8.64 |
| GAGCCAT | 8.63 |
| GAGGGTG | 8.62 |
| CACCCTG | 8.62 |
| GTGGGAC | 8.61 |
| GCGCCTC | 8.61 |
| AGGGTAT | 8.61 |
| CTAGGGG | 8.59 |
| TTACCCT | 8.58 |
| TGGCTCA | 8.57 |
| CAGGGTG | 8.56 |
| TCAGGCC | 8.56 |
| GCCCCGA | 8.56 |
| CCCCTAC | 8.55 |
| CCGGTAC | 8.55 |
| TGGGTCA | 8.54 |
| TCTCCCC | 8.54 |
| CAAGGGG | 8.52 |
| CGGCCGA | 8.52 |
| TCCCCGG | 8.52 |
| AAGGTGG | 8.51 |
| TGACATT | 8.51 |
| GGCCACA | 8.51 |
| GGTGCCT | 8.50 |
| GGCCTCT | 8.50 |
| TGGCATG | 8.50 |
| CCGGGAC | 8.50 |
| TACCCCT | 8.49 |
| GCGCCTA | 8.49 |
| CCCCTTG | 8.49 |
| TGACCCA | 8.48 |
| AATGTCA | 8.47 |
| AGGCATC | 8.47 |
| AGACCGG | 8.47 |
| AGGGTAA | 8.47 |
| CCGGTCT | 8.47 |
| CCCCACG | 8.47 |
| TAGGTCG | 8.46 |
| GTAGGGG | 8.46 |
| CCGGCTA | 8.46 |
| GCACCTT | 8.45 |
| CCACCTT | 8.45 |
| CTGGCTG | 8.43 |
| AGAGGCC | 8.42 |
| CGGGCGA | 8.42 |
| CGTGGGG | 8.42 |
| GGGCAAT | 8.42 |
| GGACCGT | 8.42 |
| GTCCCAT | 8.41 |
| GTACAGC | 8.41 |
| GATGCCT | 8.41 |
| CTTCCCC | 8.40 |
| TGTGGCC | 8.40 |
| TTGCCCT | 8.39 |
| CCGCCTG | 8.39 |
| ATGGGAC | 8.39 |
| AAGGTGC | 8.39 |
| ACAGGGG | 8.37 |
| CGGCGCC | 8.37 |
| ATTGCCC | 8.36 |
| GCCCGGG | 8.36 |
| CAGCCAG | 8.35 |
| GAGCTTG | 8.34 |
| CGCGCGA | 8.33 |
| AGGGCAA | 8.33 |
| GTCCCGG | 8.32 |
| CTCCTCC | 8.32 |
| TCACCCA | 8.32 |
| CATGCCA | 8.31 |
| CACCATG | 8.29 |
| AGGGGTA | 8.29 |
| TAGGCGC | 8.28 |
| TGGGCGT | 8.28 |
| CATGGTG | 8.28 |
| CAAGCTC | 8.28 |
| ACGCCCA | 8.27 |
| GCGCGCC | 8.26 |
| GAGGACC | 8.26 |
| CCGCGGC | 8.26 |
| GCACCTA | 8.26 |
| GAGGTGC | 8.26 |
| CATCCCC | 8.25 |
| CGACGCG | 8.25 |
| GTACACT | 8.25 |
| CCCCTGT | 8.24 |
| GCCGCGG | 8.24 |
| CAGGTCG | 8.23 |
| CGGGTTG | 8.23 |
| GGGGAGA | 8.23 |
| CGGCCAA | 8.21 |
| GGAGGAG | 8.21 |
| CAGGCGG | 8.20 |
| GTACCAG | 8.20 |
| GGACACA | 8.20 |
| GCCCTAG | 8.19 |
| TATGTGG | 8.18 |
| TCGGCCG | 8.18 |
| GGCCTTT | 8.17 |
| AGGGCGA | 8.16 |
| GGTCCTA | 8.16 |
| GACCCTT | 8.16 |
| CTGGTAC | 8.15 |
| CTGGGCT | 8.15 |
| CTGCCTT | 8.15 |
| AGCCCAG | 8.15 |
| GGGGAAG | 8.15 |
| GACCATG | 8.14 |
| CCGGCTG | 8.14 |
| TGGGTGA | 8.14 |
| CGACCTG | 8.14 |
| CAGGTGT | 8.14 |
| GGTCCTC | 8.13 |
| CCACATA | 8.13 |
| AAGGCAG | 8.13 |
| CTGGGTC | 8.13 |
| GGGGATG | 8.12 |
| CCCCAGC | 8.12 |
| GCTGTAC | 8.12 |
| TAGGACC | 8.11 |
| CCACCTA | 8.10 |
| CTAGGGC | 8.10 |
| GGCCTAT | 8.10 |
| GCACCTC | 8.10 |
| ACACCTG | 8.10 |
| TCGCCCT | 8.10 |
| TTGGCCG | 8.09 |
| ACCCTCC | 8.09 |
| TGTGTCC | 8.08 |
| CATGGTC | 8.08 |
| TAACCCT | 8.08 |
| ACTCCCC | 8.08 |
| CCCCGGC | 8.07 |
| CGTCCCT | 8.07 |
| GCCCTCG | 8.06 |
| AAGGGTC | 8.06 |
| AAGCCAG | 8.05 |
| CAACCCG | 8.05 |
| GCCCTTG | 8.05 |
| TGTCCCT | 8.05 |
| GGACTCC | 8.04 |
| GACCCAG | 8.04 |
| AGGGTTA | 8.03 |
| GACCCGG | 8.03 |
| GGAGTCC | 8.02 |
| TGGGTTC | 8.02 |
| TAGGTGC | 8.02 |
| TTGCCCG | 8.02 |
| AGTGTAC | 8.01 |
| CCCGGCG | 8.00 |
| AGGGACA | 8.00 |
| CAAGCCA | 8.00 |
| GGACCAA | 7.98 |
| TGCCCAC | 7.98 |
| GCCGCGC | 7.98 |
| TGGCTTG | 7.97 |
| AAAGGCC | 7.97 |
| TAGGCGT | 7.97 |
| GAACCCA | 7.96 |
| TAGGTGG | 7.96 |
| GTGGGCA | 7.96 |
| CCGCCTT | 7.95 |
| CGAGGGC | 7.95 |
| GGGGAGT | 7.95 |
| CAAGGGC | 7.95 |
| GGTCCTT | 7.94 |
| TCCCATA | 7.94 |
| ACGCCTA | 7.93 |
| TTGGTCC | 7.92 |
| GTGCCAG | 7.92 |
| CCGGCTT | 7.92 |
| GCTGGGG | 7.91 |
| ATACCCA | 7.91 |
| CTGGCTT | 7.91 |
| GAAGGGG | 7.90 |
| AAGGGCG | 7.90 |
| CAACCCA | 7.90 |
| GGAGGGT | 7.90 |
| GACCCTA | 7.90 |
| TGGGTAT | 7.90 |
| TCGGCTC | 7.89 |
| CATGTCT | 7.89 |
| GTGGCTC | 7.89 |
| CCTCCCA | 7.89 |
| TGTGCCT | 7.89 |
| AGTCCCA | 7.89 |
| CGGGGAT | 7.88 |
| CGTGGGC | 7.88 |
| TAGGGAG | 7.88 |
| CCCCTTC | 7.88 |
| GAGCCAC | 7.87 |
| CTGGCAC | 7.87 |
| AAGCCGG | 7.87 |
| TATGGGA | 7.87 |
| AAGGCGG | 7.87 |
| AGGCACA | 7.87 |
| AGACATG | 7.86 |
| GAGCCGT | 7.86 |
| GCCCACC | 7.86 |
| CTCCCTA | 7.86 |
| CCCGCCG | 7.86 |
| CGCCCTT | 7.84 |
| TGGGGAT | 7.84 |
| GAGCCGA | 7.83 |
| AGCCCGG | 7.83 |
| ATAGGCC | 7.82 |
| ATCCCCA | 7.82 |
| CGCCGGG | 7.81 |
| CGGGCAA | 7.81 |
| GGACCGA | 7.80 |
| CCTCCTC | 7.80 |
| GGTGGGC | 7.80 |
| AAACCCC | 7.80 |
| AAGGACC | 7.79 |
| GCGCCTT | 7.79 |
| TAGGCAG | 7.79 |
| TGGGAGG | 7.78 |
| ACTGGCC | 7.78 |
| ACGCCCG | 7.77 |
| CGACCTA | 7.77 |
| GGGGAAC | 7.77 |
| GTACTCC | 7.76 |
| GTTCCCC | 7.76 |
| GAGCTCC | 7.76 |
| GAGCCGC | 7.76 |
| GTGCCAC | 7.76 |
| TACCCCA | 7.76 |
| CGGGGTA | 7.75 |
| GGAGCTC | 7.75 |
| CAGCCAC | 7.75 |
| CTGCCTA | 7.74 |
| CGCCCTA | 7.74 |
| AGACCTG | 7.74 |
| ATCCCCG | 7.74 |
| GGGCGCC | 7.74 |
| CCGGGCT | 7.74 |
| GTGGCAC | 7.74 |
| AAGGTCG | 7.73 |
| CTACCTG | 7.73 |
| TTGCCTG | 7.72 |
| AATGGGC | 7.72 |
| CGGGCGT | 7.71 |
| AGGGGAT | 7.71 |
| AGGGACG | 7.71 |
| TGGGACT | 7.71 |
| CCAGTCC | 7.70 |
| CAGGCAA | 7.70 |
| TAGGGTC | 7.70 |
| GTAGGGC | 7.70 |
| CAGGTAG | 7.69 |
| GCCCATT | 7.69 |
| CGACCTT | 7.68 |
| GGAGTAC | 7.68 |
| GGACGGG | 7.67 |
| TTCCCCT | 7.67 |
| TGGGTTG | 7.67 |
| GGCCACT | 7.65 |
| CCGGGTC | 7.65 |
| AGCCCAC | 7.65 |
| CAGGTCT | 7.65 |
| GCCCTGT | 7.65 |
| ACAGGGC | 7.65 |
| ATGGTAC | 7.64 |
| ATCCCCT | 7.64 |
| CCACCGG | 7.64 |
| TCGGTCC | 7.63 |
| CCCCATT | 7.63 |
| AGGCAAG | 7.63 |
| GCGGCTC | 7.63 |
| CCTCCCG | 7.62 |
| GGGGTTT | 7.62 |
| CGGCTCG | 7.62 |
| TGGGGTA | 7.62 |
| CTTGCCT | 7.62 |
| AAGGCGC | 7.62 |
| CTGGTGC | 7.61 |
| GTACCAT | 7.61 |
| GGACTGG | 7.60 |
| GTGGCTG | 7.60 |
| CGAGCCA | 7.60 |
| TAGCCAG | 7.60 |
| AATGGGG | 7.60 |
| TCCCCAT | 7.60 |
| GGACGTG | 7.60 |
| CGGCATG | 7.59 |
| GCACCAG | 7.59 |
| GAGCCAA | 7.58 |
| GGCCAGT | 7.58 |
| CGACCTC | 7.58 |
| CCACCAG | 7.58 |
| ACCCCAT | 7.57 |
| GTGGGCT | 7.57 |
| TCACATA | 7.57 |
| GAGGAGG | 7.57 |
| TAGGGCG | 7.56 |
| CTGGTGG | 7.56 |
| TGGGTAA | 7.56 |
| TTACCCA | 7.55 |
| TACCCCG | 7.55 |
| GGCCAGC | 7.55 |
| GCCCTAC | 7.55 |
| GCTCCCT | 7.55 |
| TAGGCGG | 7.55 |
| ACGGCTC | 7.55 |
| CGGGAGG | 7.55 |
| GGTCCAG | 7.55 |
| CCTGGGC | 7.54 |
| AAGCCAT | 7.54 |
| TGAGGGG | 7.54 |
| CTGGCTA | 7.54 |
| TTGGCTC | 7.54 |
| CCGGTGG | 7.53 |
| ATGGCTT | 7.53 |
| GCCCACG | 7.52 |
| TATGTGA | 7.52 |
| GCCCAGG | 7.51 |
| AGTGGCC | 7.51 |
| GTCCTCC | 7.50 |
| ATGGGGA | 7.50 |
| ATGGCTG | 7.50 |
| CGGCTGG | 7.50 |
| GACCCAC | 7.50 |
| GGCGCGC | 7.50 |
| CAGCCAT | 7.49 |
| GCCGGCG | 7.48 |
| TAGCATG | 7.48 |
| GGACATC | 7.47 |
| CCGGTGC | 7.47 |
| TGGCTCG | 7.47 |
| GCGGTCC | 7.47 |
| CTGGGAG | 7.46 |
| AAGGTAG | 7.45 |
| CAAGCTG | 7.45 |
| GCACCGG | 7.45 |
| ACCCCGC | 7.45 |
| AAGGTCT | 7.45 |
| AGGGGAA | 7.45 |
| AGAGGGG | 7.45 |
| GGACTAC | 7.45 |
| GCTGGCC | 7.44 |
| CTCCCAG | 7.44 |
| AGACCTT | 7.44 |
| AAGGGTG | 7.43 |
| CAGCTTG | 7.42 |
| CCCCACA | 7.42 |
| AGGGAGC | 7.42 |
| TTCCCCG | 7.42 |
| CCCCTCT | 7.41 |
| GTACCAC | 7.41 |
| CTGGACC | 7.41 |
| GAACCTT | 7.41 |
| CCCCTCA | 7.40 |
| TGGGCAA | 7.40 |
| CCCCAAG | 7.40 |
| CACCCTT | 7.39 |
| AAGCCAC | 7.39 |
| GAGGTCG | 7.39 |
| GTGGGTC | 7.38 |
| CTACCTT | 7.38 |
| GGCGCCG | 7.38 |
| ACACCGG | 7.37 |
| TTGCCCA | 7.37 |
| GATGTCC | 7.37 |
| CCACCAC | 7.36 |
| GGAGGAC | 7.36 |
| CATGCTA | 7.35 |
| TGGCATT | 7.35 |
| CTTGGGG | 7.34 |
| AATGCCA | 7.34 |
| GCCGGGG | 7.34 |
| GATCCCC | 7.33 |
| TTGCCTC | 7.33 |
| GTGGCTT | 7.32 |
| GCCCTCA | 7.32 |
| TGGGACA | 7.32 |
| ATGGGGT | 7.32 |
| TGTGGGG | 7.31 |
| ACGCCTC | 7.31 |
| GTGGGAG | 7.30 |
| CTCCCAC | 7.30 |
| AAGGTTC | 7.30 |
| CCAGCCG | 7.30 |
| TGTCCCA | 7.29 |
| ACGCCTT | 7.29 |
| CCCCATC | 7.28 |
| CCGCCTC | 7.28 |
| TGAGGGC | 7.27 |
| CGCCCAC | 7.27 |
| CTTCCTT | 7.26 |
| TCACCTG | 7.25 |
| CGGCATT | 7.25 |
| GGCGCCC | 7.24 |
| GCAGTAC | 7.24 |
| CAGCTCC | 7.24 |
| GGGGATC | 7.23 |
| GCCCTGA | 7.23 |
| GGACCGC | 7.23 |
| GCGGGGA | 7.23 |
| TGCCCAG | 7.22 |
| TGACCTG | 7.22 |
| CCTCCTG | 7.22 |
| GGAGCTG | 7.21 |
| AAGGTGT | 7.21 |
| GAGGCAA | 7.21 |
| GTGGTGG | 7.21 |
| CAGGTGA | 7.20 |
| GTACAGA | 7.20 |
| GTGGGCG | 7.20 |
| GTGGTAC | 7.19 |
| CCACCAT | 7.19 |
| GATGGGG | 7.18 |
| AAGGAAG | 7.18 |
| GAACATC | 7.18 |
| CTGGGCA | 7.18 |
| CCCCAGT | 7.18 |
| AGTCCCT | 7.18 |
| CATGCCG | 7.18 |
| GAGGCGG | 7.18 |
| TGGCAGG | 7.17 |
| TCAGGGC | 7.17 |
| ATGCCAC | 7.16 |
| GTGCTCC | 7.16 |
| CGGCTTG | 7.16 |
| GACGCCC | 7.15 |
| CGAGCCG | 7.15 |
| GGTGTCC | 7.14 |
| GGACACC | 7.14 |
| CAGGAGG | 7.14 |
| CACCCAC | 7.14 |
| ACACCTT | 7.13 |
| GCTCCCG | 7.13 |
| ACTGGGG | 7.13 |
| GTGGGTG | 7.12 |
| CAGGTCA | 7.12 |
| CCTGCCA | 7.12 |
| GTACGTG | 7.12 |
| GGGCGTC | 7.11 |
| CTACCTA | 7.11 |
| TACCCTG | 7.11 |
| AGAGGGC | 7.11 |
| GAGCTCG | 7.11 |
| TCTGTAC | 7.11 |
| GCGGGGT | 7.10 |
| ATGGTGG | 7.10 |
| CCGGTGT | 7.10 |
| CGGGAGC | 7.10 |
| TAGGGTG | 7.10 |
| AAGGTTG | 7.09 |
| CCGCCTA | 7.09 |
| CGGGGAA | 7.09 |
| ATGCCAG | 7.09 |
| AGCCCAT | 7.08 |
| CAACCTT | 7.08 |
| GTACTGC | 7.07 |
| CAGGGAT | 7.07 |
| TCCCCGC | 7.07 |
| GGAGCTT | 7.07 |
| AAGCTCC | 7.07 |
| CCCGTCC | 7.06 |
| ATCCCTG | 7.06 |
| AAGGCAA | 7.05 |
| GGAGCAC | 7.05 |
| AGGGACT | 7.05 |
| TACCCTC | 7.04 |
| CGTCCCA | 7.04 |
| TGACCTT | 7.03 |
| CACCCTA | 7.03 |
| TCAGGGG | 7.03 |
| AAGGTCA | 7.03 |
| CAGGGTA | 7.03 |
| GCCCTCT | 7.02 |
| CTGGCAT | 7.02 |
| ATCCCTC | 7.02 |
| AATGCCG | 7.01 |
| GTAGTCC | 7.01 |
| TAGGCAA | 7.00 |
| GTACAAC | 7.00 |
| TTGCCTT | 6.99 |
| TTGCATA | 6.99 |
| TAGGTGT | 6.98 |
| GAGGGTA | 6.98 |
| GTGCCAT | 6.97 |
| GGGCAAA | 6.97 |
| GACCCAT | 6.97 |
| ATGGGCT | 6.96 |
| CAGGCGA | 6.96 |
| CGGGACA | 6.96 |
| CCCCTGA | 6.96 |
| GCGCTCC | 6.96 |
| GAGGGAT | 6.96 |
| TTAGGCC | 6.95 |
| GTCCCAA | 6.95 |
| GCCGCCC | 6.95 |
| GATGTTC | 6.95 |
| ATGGGCA | 6.95 |
| TAGGTAG | 6.94 |
| GAGGCGT | 6.94 |
| TTTGCCC | 6.94 |
| GTGGCAT | 6.94 |
| CCTCCTT | 6.94 |
| CACGGGG | 6.94 |
| GAGCTGG | 6.94 |
| GCCCATC | 6.94 |
| TTCCCCA | 6.93 |
| ACACCTA | 6.91 |
| TGCCCAT | 6.91 |
| ATGGCAC | 6.91 |
| CAGCCGT | 6.91 |
| TATGCAA | 6.91 |
| TTGGGAC | 6.90 |
| GCACCAC | 6.90 |
| GATGGGC | 6.90 |
| CAAGCCG | 6.90 |
| GGAGGCA | 6.90 |
| CTACCTC | 6.89 |
| CCGGTCG | 6.89 |
| TTGCCTA | 6.89 |
| TCGCCTG | 6.89 |
| GTGCATG | 6.89 |
| GGGCGGC | 6.88 |
| ACACCTC | 6.88 |
| CGCGACG | 6.88 |
| CACGTAC | 6.87 |
| GAGGTGT | 6.87 |
| TTGGTAC | 6.87 |
| GCACCAT | 6.87 |
| TGGGGAA | 6.87 |
| GGTCCAC | 6.86 |
| TTCCCTC | 6.86 |
| TGCCTCC | 6.86 |
| CTAGCCA | 6.86 |
| TGGGACG | 6.85 |
| AGACCTC | 6.85 |
| TGTGGAG | 6.85 |
| TGTCCCG | 6.84 |
| ATGGTGC | 6.84 |
| CCGGACC | 6.83 |
| ATGGGTC | 6.83 |
| CGGGTTA | 6.83 |
| TGGCTAG | 6.83 |
| GGCCTAA | 6.83 |
| CATGTGC | 6.82 |
| GAGGTCT | 6.82 |
| GTACCAA | 6.81 |
| CCACTCC | 6.81 |
| GAGGTAG | 6.81 |
| TGTGGGC | 6.81 |
| GAGGGAA | 6.81 |
| AGGCACT | 6.80 |
| CCGCTCC | 6.80 |
| CCCCGTG | 6.80 |
| CATGCAC | 6.79 |
| CACGTCC | 6.79 |
| CTCCACA | 6.79 |
| CCCCACT | 6.79 |
| CATGTCG | 6.78 |
| TAACCCG | 6.78 |
| GCGGCTG | 6.77 |
| TAGCCAC | 6.77 |
| GCCCTTC | 6.77 |
| AAACCCT | 6.75 |
| GGCCAGA | 6.75 |
| AAGGAGG | 6.75 |
| GTGGACC | 6.75 |
| GCACATG | 6.74 |
| AGTGCCT | 6.74 |
| GCACTCC | 6.73 |
| GGTCCGG | 6.73 |
| GCCCACA | 6.72 |
| CCAGCTC | 6.72 |
| AGGGTTT | 6.72 |
| AGACCTA | 6.70 |
| GTACCGT | 6.70 |
| GCCCAAG | 6.70 |
| AAGGCGT | 6.70 |
| ACCCTGG | 6.70 |
| TGGCTTA | 6.70 |
| TCTGGCC | 6.70 |
| TAAGGGG | 6.70 |
| TAGGTCT | 6.69 |
| CCAGGGT | 6.69 |
| GTGGTGC | 6.69 |
| ATGCTCC | 6.68 |
| AAGCTCG | 6.67 |
| GGAGCGG | 6.67 |
| CTTGGGC | 6.66 |
| GGAGTGG | 6.66 |
| GGTCCAT | 6.66 |
| GAAGGGC | 6.65 |
| GGAGCAT | 6.65 |
| ACGGTAC | 6.65 |
| TCCCCAA | 6.65 |
| ATCGTCG | 6.64 |
| GATGTTG | 6.64 |
| GTGCCGG | 6.64 |
| TAACCCA | 6.64 |
| TTGGGGA | 6.63 |
| GTGGCTA | 6.63 |
| TAAGCCA | 6.63 |
| AAGGTGA | 6.62 |
| GCCCTTA | 6.62 |
| CCCCTTA | 6.61 |
| CCGGCAC | 6.61 |
| GTACAGT | 6.61 |
| CAACATC | 6.61 |
| TCACCTT | 6.61 |
| AACCCCT | 6.60 |
| AAGCCGT | 6.60 |
| AGTGGGG | 6.60 |
| GTTGTAC | 6.60 |
| TTGGCTG | 6.60 |
| TAAGGGC | 6.59 |
| CCCCAAC | 6.59 |
| ATACCTT | 6.59 |
| CCACGGG | 6.59 |
| GACCCGA | 6.58 |
| GTCCCGC | 6.58 |
| GGACAAG | 6.57 |
| CTTGTCC | 6.57 |
| TGGGTTA | 6.57 |
| CAGCTGG | 6.56 |
| TTCCCTG | 6.55 |
| ATGGACC | 6.55 |
| CAACCTA | 6.55 |
| AGGGGTT | 6.55 |
| AAGCCAA | 6.55 |
| TCACCTA | 6.54 |
| CAGCCGC | 6.54 |
| CGGCAGG | 6.54 |
| GTAGCCA | 6.54 |
| CAGCTAC | 6.53 |
| TAGGTGA | 6.52 |
| CCAGCTG | 6.52 |
| GAGCTTA | 6.52 |
| GAGGTGA | 6.52 |
| ACTGTAC | 6.51 |
| TCGCCTC | 6.51 |
| CCAGGGA | 6.50 |
| ATGGCTA | 6.49 |
| TTCCCTT | 6.49 |
| CCAGTAC | 6.48 |
| CGGGACT | 6.48 |
| TAGCCAT | 6.47 |
| CAGGGAA | 6.47 |
| TAGGTCA | 6.47 |
| GGAGTGC | 6.47 |
| TGACATG | 6.47 |
| TTGGCTT | 6.47 |
| GAGGCGA | 6.46 |
| CTGCCAG | 6.45 |
| TGACCTA | 6.45 |
| TTCCATA | 6.45 |
| CAGGTAT | 6.44 |
| TGGCTAC | 6.44 |
| CAGCCAA | 6.44 |
| ACGGCTT | 6.44 |
| AAGGGAA | 6.44 |
| GGACGTA | 6.44 |
| CGGCTGC | 6.44 |
| ATACCTG | 6.44 |
| CTGGCAG | 6.43 |
| TCACCTC | 6.43 |
| AGGCAGC | 6.43 |
| GTGCGTG | 6.43 |
| CTGCCGG | 6.43 |
| AAGGTAT | 6.43 |
| ACCCCAA | 6.43 |
| GCTGCCT | 6.43 |
| TCCCTGG | 6.43 |
| GCGGGAC | 6.43 |
| GCAGCCA | 6.43 |
| CTCCAGT | 6.41 |
| TAAGCTC | 6.40 |
| GTACTGG | 6.40 |
| TGACCGG | 6.40 |
| GCTCCCA | 6.40 |
| TTGGGGT | 6.40 |
| TAGGTTG | 6.40 |
| TATGGAA | 6.39 |
| GAACCTG | 6.38 |
| CCACCGC | 6.37 |
| GCACCGT | 6.37 |
| TCGCCGG | 6.37 |
| TGGGAGC | 6.37 |
| GTAGCTG | 6.37 |
| CTTCCCT | 6.37 |
| CGTCCTG | 6.36 |
| TGGCTGC | 6.36 |
| GGGCGTT | 6.36 |
| GGAGTCT | 6.36 |
| ACTGCCT | 6.36 |
| GCACCAA | 6.36 |
| GCCCAGT | 6.36 |
| TCCCGGG | 6.36 |
| TCGCTCC | 6.35 |
| CTGGGTG | 6.35 |
| ACGGCTG | 6.35 |
| CACCCAG | 6.34 |
| CTCCCAT | 6.34 |
| CGACATG | 6.34 |
| GTTGGGG | 6.33 |
| ATGCCGG | 6.33 |
| TACGTCC | 6.33 |
| ACTGGAG | 6.32 |
| CCGGCAT | 6.32 |
| CAGGTAA | 6.32 |
| CGAGCTC | 6.32 |
| TGTCCTG | 6.32 |
| AACGCCC | 6.32 |
| AGACTCC | 6.31 |
| CTGGCGC | 6.31 |
| GGCCAAC | 6.31 |
| GGTCTCC | 6.31 |
| TCGGCTT | 6.31 |
| TCGGTAC | 6.31 |
| GCAGCCG | 6.31 |
| AGGCAGT | 6.31 |
| TCTCCCT | 6.30 |
| CCCGTGG | 6.30 |
| TTACCTT | 6.30 |
| CATGTCA | 6.30 |
| CGCCGGC | 6.30 |
| AAGGCGA | 6.30 |
| AAGGTAA | 6.30 |
| ATGGGAG | 6.29 |
| AGGGAAG | 6.29 |
| CAGGTTC | 6.29 |
| GCGCGTG | 6.28 |
| TTGGTGC | 6.28 |
| CCGCCGA | 6.28 |
| CAGGACA | 6.27 |
| AAGGTTA | 6.26 |
| CCAGCTT | 6.26 |
| CGAGCTT | 6.26 |
| AGTCCCG | 6.25 |
| CAACCTG | 6.25 |
| CAGGTTG | 6.25 |
| ATGCCAT | 6.25 |
| ACGGTGC | 6.25 |
| TAGCCAA | 6.24 |
| GATCGCC | 6.23 |
| AACCCCG | 6.23 |
| GGGCGGT | 6.23 |
| AAGCTGG | 6.23 |
| TTACCTG | 6.22 |
| CAAGCTA | 6.22 |
| TGACCTC | 6.21 |
| CTGCTCC | 6.21 |
| ACTGGGC | 6.20 |
| TGCCCGG | 6.20 |
| AACGTCC | 6.20 |
| AGGCAGA | 6.20 |
| GCGGTGG | 6.20 |
| CGGGGTT | 6.20 |
| CCCGGGA | 6.20 |
| CCCCGGA | 6.19 |
| GGCCAAT | 6.19 |
| CTAGCCG | 6.19 |
| TAGCTTG | 6.18 |
| AGGGAGA | 6.18 |
| GAGGTCA | 6.18 |
| CCGGTCA | 6.18 |
| ACGGTCT | 6.17 |
| TTGGCTA | 6.17 |
| CAGGACG | 6.17 |
| GGCCGCC | 6.16 |
| GCGCCAG | 6.16 |
| TCCCCGT | 6.16 |
| CTCCAGG | 6.15 |
| TGCCCAA | 6.15 |
| GTACGGA | 6.14 |
| GTGCCAA | 6.14 |
| ATGGCAT | 6.14 |
| GAGCTGC | 6.14 |
| CCGGCAG | 6.14 |
| CCTGCCG | 6.13 |
| AAGGGAT | 6.13 |
| CCTCTCC | 6.13 |
| GGAGCAG | 6.13 |
| AAGCCGA | 6.13 |
| CCCCGAG | 6.13 |
| GTCCATG | 6.12 |
| GGCGGCC | 6.12 |
| GGCCGGC | 6.12 |
| CATGGAC | 6.12 |
| GGAGCGA | 6.12 |
| GGTGCCA | 6.12 |
| GTTGGCC | 6.11 |
| TCTGCCT | 6.11 |
| GGAGACC | 6.11 |
| TAACCTT | 6.11 |
| AAGCCGC | 6.10 |
| AAGCTGC | 6.10 |
| ACAGCCA | 6.09 |
| CTGCCAC | 6.09 |
| TAGGCGA | 6.09 |
| ATCCCTT | 6.08 |
| CTACCGG | 6.08 |
| TCCGGGG | 6.08 |
| CGTGCCG | 6.07 |
| GTACGGG | 6.07 |
| GCACGGG | 6.07 |
| ACCGCCG | 6.07 |
| TACCCTT | 6.07 |
| GGCCGTG | 6.07 |
| CACGCAC | 6.06 |
| TCGCCCA | 6.06 |
| CCTGGAG | 6.06 |
| TGGCTGA | 6.06 |
| TGGCACC | 6.06 |
| CGCCCAT | 6.06 |
| TACCCTA | 6.06 |
| TCGCCTA | 6.06 |
| TCTGGGG | 6.05 |
| CCGGGCA | 6.05 |
| CTGGTGT | 6.04 |
| ACACCAG | 6.04 |
| TCAGCCA | 6.04 |
| TGTCCTT | 6.04 |
| GGAGAGG | 6.04 |
| GGACGGC | 6.03 |
| ATTGGCC | 6.03 |
| TTGGGCA | 6.03 |
| AACCCCA | 6.03 |
| GCCCTAA | 6.02 |
| GTGGAGG | 6.02 |
| TTGGCAC | 6.02 |
| GCTGTCC | 6.02 |
| CGACCGC | 6.01 |
| GTACCGC | 6.01 |
| GTGGCAG | 6.01 |
| GCACATT | 6.01 |
| CGGCTAG | 6.01 |
| AGACCGT | 6.00 |
| GGACGTC | 5.99 |
| GGACGGA | 5.99 |
| TGGCTGT | 5.99 |
| AATGTGC | 5.98 |
| TAGGGTA | 5.98 |
| CGAGCTG | 5.98 |
| AGACGGG | 5.98 |
| ATGGTGT | 5.98 |
| CACCCAT | 5.97 |
| GCAGCTC | 5.97 |
| TTGGTGG | 5.97 |
| CCGCCAG | 5.97 |
| GAAGCCA | 5.97 |
| CCTCCAG | 5.97 |
| CCCGTAC | 5.97 |
| CTCGGGG | 5.97 |
| GACGTCC | 5.97 |
| TGGGGTT | 5.96 |
| ACGGGGA | 5.96 |
| ATACCTC | 5.96 |
| GGACAGA | 5.96 |
| ACGGTGG | 5.96 |
| GCCCTAT | 5.95 |
| ATCCCTA | 5.95 |
| CTGGTAG | 5.95 |
| CACCAGG | 5.95 |
| CTACCAT | 5.95 |
| GCAGCTG | 5.95 |
| CGACCGG | 5.95 |
| TAGGGAT | 5.95 |
| ATGGGCG | 5.95 |
| ACACCAT | 5.95 |
| GGACAGC | 5.95 |
| GCAGCTT | 5.94 |
| CCTCCAC | 5.94 |
| TCGCCTT | 5.93 |
| GGACTGC | 5.93 |
| CTAGTCC | 5.93 |
| CCCCAGA | 5.93 |
| AAGGGTA | 5.93 |
| GCTCCTC | 5.93 |
| GACCCGC | 5.93 |
| TGGCTTC | 5.93 |
| AAGGACG | 5.92 |
| CCTGGTG | 5.92 |
| TCTGTCC | 5.92 |
| TCGCGCG | 5.92 |
| AGCCTGG | 5.91 |
| CTACCAG | 5.91 |
| CTGGAGG | 5.91 |
| CACGGCC | 5.91 |
| AACGGCC | 5.91 |
| ACCCGGG | 5.91 |
| GAGGAGC | 5.91 |
| TGGGCGA | 5.90 |
| ATAGGGG | 5.90 |
| TTACCTA | 5.90 |
| GTCCCGT | 5.90 |
| TAGCTCC | 5.90 |
| CCCCTAT | 5.90 |
| CCCGTGC | 5.89 |
| CCGGCGT | 5.89 |
| TGGCACG | 5.89 |
| GAGGACA | 5.88 |
| CCGGTAG | 5.88 |
| GCAGTCC | 5.88 |
| CGTCCTC | 5.88 |
| CTAGCTC | 5.88 |
| ATACCTA | 5.88 |
| CTGGCGG | 5.88 |
| TGTCCTC | 5.88 |
| GAGGTTG | 5.88 |
| GGCCGTT | 5.87 |
| TGTGCCA | 5.87 |
| TACCATG | 5.87 |
| CGCGGGA | 5.87 |
| GCGGCTT | 5.87 |
| TAGCCGA | 5.86 |
| TGGCACA | 5.86 |
| CCACCAA | 5.86 |
| ATGGTAG | 5.86 |
| AGACCAG | 5.86 |
| GAGGTTC | 5.86 |
| GGACTAG | 5.85 |
| GGAGCTA | 5.85 |
| GAGCATC | 5.85 |
| TAGGTAT | 5.85 |
| CGGCGGT | 5.85 |
| CTCCCAA | 5.84 |
| TCCCGCG | 5.84 |
| AAGGACA | 5.84 |
| CCCGCGA | 5.84 |
| CCCGGCC | 5.83 |
| CCACCGT | 5.83 |
| CCTCCTA | 5.83 |
| GTAGCCG | 5.83 |
| CCAGGCT | 5.83 |
| CAGCTGC | 5.83 |
| GTTGCCT | 5.82 |
| GATGCTC | 5.81 |
| TAGGTAA | 5.81 |
| TAGGAGG | 5.81 |
| TAGCCGC | 5.80 |
| CCCCGGT | 5.80 |
| GAGCTAG | 5.80 |
| CATGCAT | 5.80 |
| CGTGCCA | 5.80 |
| GTACCGA | 5.80 |
| AGCCCAA | 5.79 |
| AGGCAAC | 5.79 |
| GCGGTAC | 5.79 |
| CAGCTTA | 5.79 |
| GGAGGTG | 5.79 |
| ATGGGTG | 5.78 |
| CATGGTA | 5.78 |
| TTAGGGC | 5.78 |
| ACGGCTA | 5.78 |
| GGCGTCC | 5.78 |
| GAACCTC | 5.78 |
| GCGCCGA | 5.77 |
| GTGGAGC | 5.77 |
| GGGCGTG | 5.77 |
| ATAGGGC | 5.77 |
| ACGCCGG | 5.77 |
| GCGGGCT | 5.77 |
| CACGCGC | 5.76 |
| CAACCTC | 5.76 |
| TATCCCC | 5.76 |
| GCTCCAG | 5.76 |
| TAGCGTG | 5.76 |
| ACCGGGG | 5.76 |
| GCCCGTG | 5.76 |
| GAGGTAT | 5.76 |
| ATGCATG | 5.75 |
| CATCCCT | 5.75 |
| CCCGTCT | 5.75 |
| CACCTCC | 5.75 |
| TTGGGCT | 5.75 |
| AATGGCT | 5.75 |
| CGCGTCG | 5.75 |
| CCACATG | 5.75 |
| AGACCGA | 5.75 |
| CAGCTCG | 5.74 |
| TTAGCCG | 5.74 |
| GCCCAGC | 5.74 |
| ACGCCAG | 5.74 |
| GCTCCAC | 5.74 |
| TCCGCGC | 5.73 |
| AAAGGGC | 5.73 |
| GAGGTAA | 5.72 |
| CTGGTCT | 5.71 |
| CAGCCGA | 5.71 |
| AGGGATG | 5.71 |
| CATGTGG | 5.71 |
| GCTGGGC | 5.71 |
| AGGCGCC | 5.70 |
| CGACGAT | 5.70 |
| ACTCCCT | 5.70 |
| GGCCGGG | 5.70 |
| TAAGCTG | 5.70 |
| AGGGAGT | 5.70 |
| CGGCGTC | 5.70 |
| CGGCTAA | 5.70 |
| CACCGGG | 5.69 |
| GAGGACG | 5.69 |
| TTGGGAG | 5.69 |
| CCGCCAC | 5.69 |
| ATGCCAA | 5.69 |
| TCGGGAC | 5.69 |
| GGGCGCG | 5.69 |
| ACGGGAC | 5.69 |
| ATGGTCG | 5.68 |
| TCGGCTA | 5.68 |
| TCACCAC | 5.67 |
| AGCCCGC | 5.67 |
| GAACCTA | 5.67 |
| GCACCGC | 5.67 |
| GCCCTTT | 5.67 |
| AGCCATT | 5.67 |
| CGTCCTT | 5.67 |
| GGACGTT | 5.67 |
| GCCCACT | 5.66 |
| CACGCCC | 5.66 |
| TTACCTC | 5.66 |
| TACGCCC | 5.66 |
| AATGTAT | 5.65 |
| AGCCATG | 5.65 |
| CTAGCTG | 5.65 |
| AACCATG | 5.65 |
| GACGGCC | 5.65 |
| AGCCTCC | 5.65 |
| TGGCTAA | 5.65 |
| CGGCTTA | 5.65 |
| CAGGGTT | 5.64 |
| AAAGGGG | 5.64 |
| CATGGTT | 5.64 |
| CTGGTCG | 5.64 |
| TGGCATC | 5.64 |
| ACGGCGC | 5.64 |
| ACGCTCC | 5.64 |
| GGACAGT | 5.64 |
| GGGCGTA | 5.64 |
| GTCCTGG | 5.63 |
| GCCGGCC | 5.63 |
| TTGGCAT | 5.63 |
| CATGGCT | 5.63 |
| CCGGGTA | 5.63 |
| CAGCTAG | 5.63 |
| CGGCTTC | 5.62 |
| ACTGTCC | 5.62 |
| CTCCCGG | 5.62 |
| TTAGCCA | 5.62 |
| ACAGCCG | 5.62 |
| GGACGCG | 5.61 |
| AAGCTGA | 5.61 |
| ACCCATG | 5.61 |
| CCGGGAG | 5.61 |
| GCTCCTG | 5.61 |
| CGGCACG | 5.61 |
| GTGCATT | 5.60 |
| GGAGCGC | 5.60 |
| TAGGTTC | 5.60 |
| AAACCCG | 5.60 |
| CAGGAGC | 5.60 |
| GACCAGG | 5.60 |
| CCACTGG | 5.59 |
| ATGGTGA | 5.59 |
| CATGGGA | 5.58 |
| CGGCTAC | 5.58 |
| TCGGCTG | 5.58 |
| ATACATT | 5.57 |
| AACCCTG | 5.57 |
| CCTGCTC | 5.57 |
| GAAGCCG | 5.57 |
| AGGCAAT | 5.57 |
| AAGCTGT | 5.57 |
| GAGCAGG | 5.57 |
| CCCCTTT | 5.57 |
| GACCCAA | 5.56 |
| GGAGCGT | 5.56 |
| CTGGAGC | 5.56 |
| CTAGCTT | 5.56 |
| AGTGGGC | 5.56 |
| CCAGTGG | 5.56 |
| CACGGGC | 5.56 |
| GGACTCG | 5.56 |
| CCAGGAC | 5.56 |
| TCACCAT | 5.56 |
| CCCGGTG | 5.56 |
| ACAGCTC | 5.55 |
| GTCCATT | 5.55 |
| TTGCTCC | 5.55 |
| GTGGTGA | 5.55 |
| AGACCAT | 5.54 |
| ACAGCTT | 5.54 |
| TGAGCTT | 5.54 |
| CCCGTCG | 5.54 |
| AAGCTCA | 5.54 |
| TCAGCTT | 5.53 |
| GCCCGGC | 5.53 |
| ATGGTCT | 5.53 |
| GCGGTCG | 5.53 |
| TTCCTCC | 5.53 |
| CGTCCGA | 5.53 |
| ACACCAC | 5.52 |
| ATTGCCT | 5.52 |
| TTGCCAG | 5.52 |
| GATGCCA | 5.52 |
| CGACCAG | 5.51 |
| GGTGGGT | 5.51 |
| AAACCTT | 5.51 |
| TACGTAC | 5.51 |
| GTTCCCT | 5.51 |
| TTGGGTC | 5.51 |
| CCACAGG | 5.50 |
| CGCGCCC | 5.50 |
| GGAGGCT | 5.50 |
| GGAGGAA | 5.50 |
| GAGGGTT | 5.50 |
| TGAGCTC | 5.50 |
| GTACGTA | 5.50 |
| CTTGCCA | 5.50 |
| ACGGCAC | 5.49 |
| GTCCGGG | 5.49 |
| GAGCTGT | 5.49 |
| CTGGCAA | 5.49 |
| CGACCAT | 5.49 |
| ACCCACC | 5.49 |
| CGGCTGT | 5.48 |
| AACCCTC | 5.48 |
| CATGGGT | 5.48 |
| GTGGCGG | 5.48 |
| TCGGGGT | 5.48 |
| CGGCACC | 5.48 |
| AAGCTAG | 5.48 |
| ACCGCGG | 5.48 |
| GGACGCC | 5.47 |
| TCCCACC | 5.47 |
| AATGCAC | 5.47 |
| CCTGGTC | 5.47 |
| AAGGTTT | 5.47 |
| GAGCTCA | 5.47 |
| CCGCCGT | 5.47 |
| TCTCCCG | 5.47 |
| CCCGGAC | 5.47 |
| TCCCAGG | 5.46 |
| CATGTAG | 5.46 |
| TGGCAAG | 5.46 |
| TCCGTCC | 5.45 |
| CGGGTTT | 5.45 |
| CCTGTGC | 5.45 |
| TCCCATG | 5.45 |
| TTAGGGG | 5.45 |
| GTGGGTA | 5.45 |
| TAGCTGG | 5.45 |
| GGCGCCT | 5.45 |
| TGCCCGT | 5.45 |
| CCCGGAG | 5.45 |
| TGGCGGG | 5.44 |
| ACGGTCG | 5.44 |
| TAAGCCG | 5.44 |
| TCGGTCT | 5.44 |
| TACCCAC | 5.43 |
| GACCTCC | 5.43 |
| ACCGCCC | 5.43 |
| TACCCGG | 5.43 |
| AATGGAC | 5.43 |
| GGGCGAA | 5.43 |
| GGTCCAA | 5.43 |
| GTGGTGT | 5.42 |
| CCTGGGA | 5.42 |
| GTACTAC | 5.42 |
| GGAGGTC | 5.41 |
| TCACCGG | 5.41 |
| GCACAGG | 5.41 |
| CTCCGGG | 5.41 |
| GCGCCAC | 5.41 |
| GGGGATA | 5.40 |
| CTACATG | 5.40 |
| CGGGAGA | 5.40 |
| GTCCCGA | 5.40 |
| CCTGTGG | 5.40 |
| GTACGCG | 5.40 |
| CGTCCTA | 5.39 |
| CGTCCGG | 5.39 |
| CCAGCTA | 5.39 |
| TCGCGGG | 5.38 |
| GGAGCAA | 5.38 |
| CTACCAA | 5.38 |
| GCCCGCC | 5.38 |
| GGACACT | 5.38 |
| AGGGAAC | 5.38 |
| GAGCTGA | 5.38 |
| CAGCATC | 5.37 |
| GCGGCTA | 5.37 |
| TCCCCGA | 5.37 |
| GGGCGAG | 5.37 |
| CGCCCGT | 5.37 |
| CCCGGGT | 5.37 |
| TCGCGGC | 5.37 |
| CATCCCA | 5.36 |
| GCGGGTC | 5.36 |
| GAGCACG | 5.36 |
| GGACTGT | 5.36 |
| CGAGTCC | 5.36 |
| TACCCAG | 5.36 |
| GTACTCG | 5.36 |
| GGAGACG | 5.36 |
| CGAGTAC | 5.35 |
| GGCCAAA | 5.35 |
| GAAGCTC | 5.35 |
| CTCCTTC | 5.35 |
| CTACGGG | 5.35 |
| CTCCAGC | 5.35 |
| ACGGGCA | 5.35 |
| GTACGTC | 5.34 |
| TCGGCGC | 5.33 |
| GCTGGAG | 5.33 |
| AATGTAG | 5.33 |
| TTTGGCC | 5.33 |
| TTGGACC | 5.33 |
| TAGGGAA | 5.33 |
| GCGGGCG | 5.32 |
| TTGGTAG | 5.32 |
| GTCGGGG | 5.32 |
| TCTCCCA | 5.32 |
| GATGCTG | 5.32 |
| TCAGCTC | 5.32 |
| GCGCGCT | 5.31 |
| GCTCCTT | 5.31 |
| TCGGTGC | 5.31 |
| TAACATC | 5.31 |
| TTCCCTA | 5.31 |
| CTGGGTA | 5.30 |
| TAGCCGT | 5.30 |
| CAGCTGT | 5.30 |
| CCAGTGC | 5.30 |
| CCGGTGA | 5.30 |
| TGCCATT | 5.30 |
| GTGGCGC | 5.29 |
| ACGGCGG | 5.29 |
| TCGGGGA | 5.29 |
| GAGCTTC | 5.29 |
| GGAGTCG | 5.29 |
| GGTGGGA | 5.28 |
| TTGGGTG | 5.28 |
| TCGGGCT | 5.28 |
| TCTGGGC | 5.28 |
| GAAGGAG | 5.28 |
| CACCCAA | 5.28 |
| CTCGCCC | 5.28 |
| TGGGAGA | 5.28 |
| CTACCAC | 5.28 |
| ACAGCTG | 5.27 |
| CCTGCAC | 5.27 |
| GGCGGGC | 5.27 |
| CAGCAGG | 5.27 |
| AGTGTCC | 5.27 |
| GATGCTT | 5.26 |
| CTACATT | 5.26 |
| CGGGAAG | 5.26 |
| ACTCCCA | 5.26 |
| ACCCCGT | 5.25 |
| ATTGTAC | 5.25 |
| ATAGCCA | 5.25 |
| ACGCGGG | 5.25 |
| AGAGCCA | 5.25 |
| AATGGCA | 5.25 |
| GGCGATC | 5.25 |
| AAGCATC | 5.25 |
| ACGGGCT | 5.25 |
| AGTCCTG | 5.25 |
| GTAGCTT | 5.24 |
| TCTCCTC | 5.24 |
| AACGTAC | 5.24 |
| CGCCCAG | 5.24 |
| CAGGACT | 5.24 |
| ACCCCGA | 5.24 |
| GGGCGCT | 5.24 |
| GGTGCCG | 5.23 |
| TGGGAGT | 5.23 |
| TCCCTTC | 5.23 |
| GATCCCT | 5.23 |
| GTACAAT | 5.23 |
| TGGGATG | 5.23 |
| GGTCGGG | 5.22 |
| CGCGTCC | 5.22 |
| GCCCAGA | 5.22 |
| CGGGAAC | 5.22 |
| GTAGTAC | 5.22 |
| ACAGTCC | 5.22 |
| ATTCCCC | 5.22 |
| TCGGCGG | 5.21 |
| TCACCAG | 5.21 |
| AACCCTT | 5.21 |
| CGTCTCC | 5.21 |
| CCTGGGT | 5.21 |
| CTGGTGA | 5.21 |
| ACCCAGG | 5.20 |
| TGTCCTA | 5.20 |
| AGACGGA | 5.20 |
| CTTCCCG | 5.20 |
| GTGCCGT | 5.20 |
| GCACCGA | 5.20 |
| ATGGCAG | 5.20 |
| TGGCTAT | 5.19 |
| GCTCCGG | 5.19 |
| TGGCTCT | 5.19 |
| CTGGGCG | 5.19 |
| GTGCCGA | 5.19 |
| GTTCCCG | 5.19 |
| CACCCGG | 5.19 |
| TAGCGCG | 5.18 |
| GTGCAGG | 5.18 |
| CCGGAGG | 5.18 |
| CGCCATT | 5.18 |
| GCGGCAC | 5.18 |
| AAGGAGC | 5.18 |
| AACGGGC | 5.18 |
| TAGGACA | 5.18 |
| GCACGTG | 5.18 |
| AGCCCGT | 5.18 |
| CCGGCGA | 5.17 |
| GATGTTA | 5.17 |
| GTCCAGG | 5.17 |
| AAGCTAC | 5.17 |
| CCTGCTG | 5.17 |
| GGCCGTC | 5.16 |
| TCAGCCG | 5.16 |
| GAACCGG | 5.16 |
| GAAGGGA | 5.16 |
| CTAGTAC | 5.15 |
| TTTCCCC | 5.15 |
| GCCCAAC | 5.15 |
| TCGGGCG | 5.15 |
| CCTGGAC | 5.15 |
| CCTCCGG | 5.15 |
| GCACTGG | 5.15 |
| AGGCAAA | 5.14 |
| AGGGATC | 5.14 |
| TCGCCGC | 5.14 |
| GAGGAGA | 5.14 |
| AGCGCCC | 5.13 |
| CCGGCAA | 5.13 |
| TGTCCAG | 5.13 |
| CAGGTTA | 5.13 |
| CTGGTCA | 5.13 |
| CCGGTTC | 5.13 |
| TGCCTGG | 5.12 |
| GGAGGCG | 5.12 |
| GAGGACT | 5.12 |
| TTGCCGG | 5.12 |
| AGTCCTC | 5.12 |
| AATGGCG | 5.12 |
| GCGGTGC | 5.12 |
| CGGCTGA | 5.11 |
| AAGGGTT | 5.11 |
| GTACTAG | 5.11 |
| CCCCTAA | 5.11 |
| CACGGAG | 5.11 |
| GGCCGGT | 5.11 |
| GAACGGA | 5.10 |
| AGCGCGC | 5.10 |
| GACGTAC | 5.10 |
| CTGGCGT | 5.10 |
| CTGCCAT | 5.10 |
| CCGGGTG | 5.10 |
| TGTCTCC | 5.10 |
| CCAGGCA | 5.10 |
| TAGCTTA | 5.09 |
| TGACCAG | 5.09 |
| ATGCCGT | 5.09 |
| CTGGAGT | 5.09 |
| TAGGACG | 5.09 |
| GAAGGAA | 5.09 |
| GCAGGGT | 5.09 |
| TCAGCTG | 5.09 |
| CCCGCCA | 5.08 |
| CGACCGT | 5.08 |
| CGCCCGC | 5.08 |
| TTTGCCT | 5.08 |
| ATGGCGC | 5.08 |
| ACTCCAG | 5.08 |
| CGTGCTC | 5.07 |
| ACGCCAC | 5.07 |
| ACGGGGT | 5.07 |
| CGGCTCT | 5.07 |
| TTTCTTT | 5.07 |
| GCGCGGA | 5.07 |
| ATACCAT | 5.07 |
| GAGCAAG | 5.06 |
| TGAGTCC | 5.06 |
| TGCCAGG | 5.06 |
| CGACCAA | 5.06 |
| GTAGCTC | 5.06 |
| CCTGGCA | 5.06 |
| ACCCTGC | 5.06 |
| TAAGCTA | 5.06 |
| TTGGGCG | 5.05 |
| TAACCTA | 5.05 |
| GTGGTAG | 5.05 |
| AGCCCGA | 5.05 |
| TAACCTC | 5.05 |
| CTGGACA | 5.05 |
| AGGCGGG | 5.04 |
| GAGCTAC | 5.04 |
| CGCGTAC | 5.04 |
| GAAGCTG | 5.04 |
| GGGGAAA | 5.04 |
| CTTGCTC | 5.04 |
| TAACCTG | 5.03 |
| CCTCGGG | 5.03 |
| TGTCCAC | 5.03 |
| GCAGGGA | 5.03 |
| GTTGGGC | 5.03 |
| CAGCTTC | 5.03 |
| GGGCGGA | 5.03 |
| CCGGACG | 5.02 |
| GGGGAAT | 5.02 |
| ATGGTAT | 5.02 |
| TCGGGTC | 5.02 |
| CGGGATG | 5.02 |
| CGCCTCC | 5.02 |
| CCGCGGT | 5.01 |
| CTGCGCG | 5.01 |
| TCGGTGG | 5.00 |
| CTACCGT | 5.00 |
| TAGCTCG | 5.00 |
| CAGCTGA | 5.00 |
| ATGGTCA | 4.99 |
| GGACTCA | 4.99 |
| ACTCCCG | 4.99 |
| GAGGTTA | 4.99 |
| CTTCCTC | 4.99 |
| GTGCGGG | 4.99 |
| TTGGTCG | 4.99 |
| GCCCGAG | 4.99 |
| ACACCAA | 4.98 |
| GCCCGGA | 4.98 |
| TTGGCGC | 4.98 |
| TGACCAT | 4.98 |
| AAAGCCG | 4.98 |
| ACACCGT | 4.98 |
| ACCGGCC | 4.98 |
| CATCCCG | 4.98 |
| AGACCAA | 4.97 |
| TCCCTGC | 4.97 |
| ACGGGTC | 4.97 |
| GCGGCGA | 4.97 |
| ACCGGGC | 4.97 |
| GACCCGT | 4.97 |
| TCCGCGG | 4.96 |
| GTACGTT | 4.96 |
| TGGCTTT | 4.96 |
| CGAGCTA | 4.96 |
| TTCCTTC | 4.96 |
| TAGGTTA | 4.96 |
| CTGGTAA | 4.96 |
| CAGCACG | 4.96 |
| GTGCCGC | 4.96 |
| AAACCCA | 4.96 |
| TGACCGT | 4.95 |
| AATGTGT | 4.95 |
| ACGCCAT | 4.95 |
| CGGCAAG | 4.95 |
| GCCGGGC | 4.95 |
| GCCCGGT | 4.94 |
| GTGGCAA | 4.94 |
| CTCGGGC | 4.94 |
| TGGGTTT | 4.94 |
| CCGCGCA | 4.94 |
| CTCCGTG | 4.94 |
| TGCCCGC | 4.94 |
| CGACGGG | 4.93 |
| GGACTGA | 4.93 |
| TACGGGC | 4.93 |
| TCTCCTG | 4.93 |
| TTACCAG | 4.93 |
| TGCCATG | 4.92 |
| GAGGAAG | 4.92 |
| CTCCCGC | 4.92 |
| AGACCAC | 4.92 |
| GCGCCAT | 4.91 |
| CGCGTTG | 4.91 |
| GGAGACA | 4.91 |
| CCCCGTC | 4.91 |
| ATGCCGA | 4.91 |
| TCCGGGC | 4.91 |
| AGTCCTT | 4.91 |
| CGACTCC | 4.91 |
| CATGGCA | 4.90 |
| CAGGAGA | 4.90 |
| CTCCTCT | 4.90 |
| TTGGTGT | 4.90 |
| ATAGCTT | 4.90 |
| ACGGCAT | 4.90 |
| CCCGCCT | 4.90 |
| GTACAAA | 4.89 |
| GGTCCGT | 4.89 |
| TTGCCAC | 4.89 |
| ATGCATT | 4.88 |
| AATGCAT | 4.88 |
| CGTCCAG | 4.88 |
| CGACCAC | 4.88 |
| GACGCCG | 4.88 |
| GCGGGAG | 4.88 |
| CCCCGAC | 4.87 |
| GCCCGTA | 4.87 |
| AGTGCCA | 4.87 |
| CGCCCAA | 4.87 |
| GTTCCCA | 4.87 |
| CCCGAGG | 4.87 |
| AGCGCGG | 4.87 |
| TTGGTCT | 4.86 |
| GGACGGT | 4.86 |
| TCAGTCC | 4.86 |
| ACGGTGT | 4.86 |
| GCGCTCG | 4.86 |
| CTCGCGG | 4.86 |
| TGTCCAT | 4.86 |
| GTGGGAT | 4.86 |
| GTGGACA | 4.85 |
| ATCCCAC | 4.85 |
| ACCCTCG | 4.85 |
| ACCCGGC | 4.85 |
| CTGCCAA | 4.85 |
| GTGCACG | 4.85 |
| AATCCCC | 4.85 |
| CGGCTTT | 4.84 |
| CCACGTG | 4.84 |
| GGCCGGA | 4.84 |
| GAGGAGT | 4.83 |
| AGAGGAG | 4.83 |
| ATCCCAG | 4.82 |
| CAGCAAG | 4.82 |
| GTGGCGT | 4.82 |
| ACCCATT | 4.82 |
| GCGGGCA | 4.82 |
| GCGCCGT | 4.82 |
| CTGGGAT | 4.82 |
| GGCCGAG | 4.82 |
| ACGGTCA | 4.82 |
| ACACGGG | 4.82 |
| GTGGTCT | 4.82 |
| ATGGTTG | 4.81 |
| CCCGCAC | 4.81 |
| TGAGTAC | 4.81 |
| CTTCCCA | 4.81 |
| CTGCATG | 4.81 |
| AAGCTAT | 4.81 |
| ATAGCCG | 4.81 |
| AGCCGGG | 4.81 |
| ATACATG | 4.81 |
| CCGGAGC | 4.81 |
| CAACCAT | 4.80 |
| TGGGAAC | 4.80 |
| TGGGAAG | 4.80 |
| CACGTGC | 4.80 |
| ACACATT | 4.79 |
| CACGCTA | 4.79 |
| CTTGCCG | 4.79 |
| AAAGCCA | 4.79 |
| TGGCAGC | 4.79 |
| CGTGCTG | 4.79 |
| ACCCGCG | 4.79 |
| ACGGGCG | 4.79 |
| GGACGAG | 4.79 |
| GGACAAC | 4.78 |
| CGACCGA | 4.78 |
| CGTCGGG | 4.78 |
| GTGGTCG | 4.78 |
| ATGGACA | 4.78 |
| GTCGTCG | 4.78 |
| ACTCCTC | 4.78 |
| GCTCCTA | 4.78 |
| ATGGAGC | 4.78 |
| TGGCACT | 4.78 |
| GACCTGG | 4.77 |
| TCACCAA | 4.77 |
| TAGGAGC | 4.77 |
| CGGCGGA | 4.77 |
| ATGGCGT | 4.77 |
| TTTGTAC | 4.77 |
| ATGCTTG | 4.77 |
| CATGCAG | 4.77 |
| GGTGCAC | 4.76 |
| TCTCCTT | 4.76 |
| GAGCTAT | 4.76 |
| AGACCGC | 4.76 |
| TCCGCCG | 4.76 |
| CAACCAG | 4.75 |
| GCGGCAG | 4.75 |
| CATGTAT | 4.75 |
| CGAGCGG | 4.75 |
| GTACTCA | 4.75 |
| GCGGTCT | 4.75 |
| TGACCAA | 4.75 |
| CCTCCAT | 4.75 |
| CCAGGTC | 4.75 |
| TCCCGAG | 4.75 |
| CGCCGGA | 4.74 |
| GACGGGG | 4.74 |
| CAGCTCA | 4.74 |
| TAGCTAG | 4.74 |
| CCACCGA | 4.74 |
| CTAGCTA | 4.74 |
| GCAGCTA | 4.74 |
| GAACCAT | 4.74 |
| TAGCATC | 4.74 |
| CAAGTCC | 4.74 |
| TCCGTAC | 4.74 |
| GCTGCCA | 4.74 |
| GCTCCAT | 4.74 |
| ACAGTAC | 4.74 |
| ACGGACC | 4.73 |
| ACGGGAG | 4.73 |
| AATGGGT | 4.73 |
| CACGTGG | 4.73 |
| ACGGTAG | 4.73 |
| GTGCACC | 4.73 |
| GCGCGCA | 4.72 |
| TACCTCC | 4.72 |
| AAGGACT | 4.72 |
| CGTGCAC | 4.72 |
| ATACCAG | 4.72 |
| TTGGCAG | 4.72 |
| GCACACG | 4.71 |
| GATGCCG | 4.71 |
| CGGGAGT | 4.71 |
| TACCCAT | 4.71 |
| CAAGCAT | 4.71 |
| AGCCAGG | 4.71 |
| CGTGCTT | 4.70 |
| CTTGCTG | 4.70 |
| TAGGACT | 4.70 |
| GTCGGGC | 4.70 |
| GCCGTCC | 4.70 |
| ATGCCGC | 4.70 |
| GGACTAT | 4.70 |
| TTGGTGA | 4.69 |
| GGAGGTA | 4.69 |
| CCCGACC | 4.69 |
| TGCCCGA | 4.69 |
| GATGGTG | 4.69 |
| CCGCGCT | 4.69 |
| CCCCGCA | 4.69 |
| CTCGCCG | 4.69 |
| CACCATC | 4.68 |
| CGGCATC | 4.68 |
| GATGTTT | 4.68 |
| CTGGTAT | 4.68 |
| CCGCGGA | 4.68 |
| CCCGTGT | 4.68 |
| AGTCCTA | 4.68 |
| TGGCGAT | 4.68 |
| TCGGCAC | 4.67 |
| AGAGCCG | 4.67 |
| TGAGCCG | 4.67 |
| CGGCTCA | 4.67 |
| ATGGTTC | 4.67 |
| CTGGACG | 4.67 |
| ACGGGTG | 4.67 |
| TGACGGG | 4.66 |
| GTACTGA | 4.66 |
| GGACTTG | 4.66 |
| ACCCGCC | 4.66 |
| CCCGCAG | 4.66 |
| TGAGCTG | 4.66 |
| CTGCCGC | 4.65 |
| GAGCGCG | 4.65 |
| CGCGGGT | 4.65 |
| TCCGCCC | 4.65 |
| CAACCGG | 4.65 |
| CGGCTAT | 4.65 |
| CTGGTTG | 4.65 |
| GACCATC | 4.65 |
| CAAGGGT | 4.65 |
| CCCGCGT | 4.64 |
| ACTCCTT | 4.64 |
| CCGCCAT | 4.64 |
| CTCCGTC | 4.64 |
| CCCGGCT | 4.64 |
| GTTGTCC | 4.64 |
| GGCCGTA | 4.64 |
| ACCGTCC | 4.63 |
| CAAGCAC | 4.63 |
| TTGGTCA | 4.63 |
| GATGGTC | 4.63 |
| ACCCTTG | 4.63 |
| CCCGGTC | 4.63 |
| AAGGAGT | 4.63 |
| AGGCGTC | 4.63 |
| CGGCGTT | 4.63 |
| GTACTGT | 4.63 |
| ATACCGG | 4.62 |
| CTGCCGT | 4.62 |
| ATTGGGC | 4.62 |
| ATGGCGG | 4.62 |
| GGGGATT | 4.62 |
| CTCCCGT | 4.62 |
| GCGGACC | 4.62 |
| CATCCTG | 4.62 |
| GCGGCGT | 4.62 |
| ATCCATT | 4.62 |
| GCGCCAA | 4.61 |
| CACGTCG | 4.61 |
| CGTGTGC | 4.60 |
| ATGCTGG | 4.60 |
| GGACGCA | 4.60 |
| AATGGAT | 4.60 |
| CAGGATG | 4.60 |
| ATAGCTC | 4.60 |
| CTCGTCC | 4.60 |
| TCGGCAT | 4.60 |
| AAAGAAA | 4.59 |
| CGCGGAG | 4.59 |
| GGAGTGT | 4.59 |
| GTCCACC | 4.59 |
| GATGCTA | 4.59 |
| GCAGTGC | 4.59 |
| CATGTGT | 4.59 |
| ATGGAGG | 4.59 |
| ATAGTCC | 4.59 |
| CGACGCC | 4.58 |
| CGCCCGA | 4.58 |
| TCGGGCA | 4.58 |
| CGAGGGT | 4.58 |
| AAGGAGA | 4.58 |
| ATCCCGG | 4.58 |
| GCCCGAC | 4.57 |
| ACACTCC | 4.57 |
| CCTGGCT | 4.57 |
| CCACGCC | 4.57 |
| TTCCCAG | 4.57 |
| ATGGGTA | 4.57 |
| CTACTCC | 4.57 |
| CTGCGGG | 4.57 |
| AAACATC | 4.57 |
| CTCGGCC | 4.57 |
| CGACGTG | 4.57 |
| GCCCAAT | 4.57 |
| GCACTGC | 4.57 |
| TCCCGCC | 4.57 |
| CCACACC | 4.57 |
| GAGCACA | 4.56 |
| CCGCCAA | 4.56 |
| GGAGTCA | 4.56 |
| CCGGTTG | 4.56 |
| TAGCTGC | 4.55 |
| TAGGGTT | 4.55 |
| GAGCACC | 4.55 |
| ATCCTCC | 4.55 |
| GTGCTTG | 4.55 |
| ACACATG | 4.55 |
| CGCGGTC | 4.55 |
| GATCCTC | 4.54 |
| TTACCAT | 4.54 |
| CCACTGC | 4.54 |
| TGCCACC | 4.54 |
| CTAGGGA | 4.54 |
| TAGCTAC | 4.54 |
| AGTCCAG | 4.54 |
| TACCGGT | 4.53 |
| CCCGCAT | 4.53 |
| TGCGGGG | 4.53 |
| TTCGCCC | 4.53 |
| GGTCCGC | 4.53 |
| ACGCCGC | 4.52 |
| TGCCTGT | 4.52 |
| GGAGTAG | 4.52 |
| TCCGGCG | 4.52 |
| ACGGCAG | 4.52 |
| GGTGGAC | 4.52 |
| CGAGTGG | 4.52 |
| AACCCTA | 4.52 |
| ATGCGGG | 4.51 |
| GCGCGTC | 4.51 |
| ATGGTAA | 4.51 |
| GTACGGC | 4.51 |
| ACTGCCA | 4.50 |
| CAAGGGA | 4.50 |
| TGTGCTC | 4.50 |
| TCAGTAC | 4.50 |
| GCCCGTT | 4.50 |
| TGCGCGC | 4.49 |
| ACAGGCA | 4.49 |
| GAACCAG | 4.49 |
| GGGCGAC | 4.49 |
| TACCGGG | 4.49 |
| TCCCTTG | 4.49 |
| GCCGGGT | 4.49 |
| TTGGCGG | 4.48 |
| CAACCGT | 4.48 |
| CCGGGAT | 4.48 |
| AATGGGA | 4.48 |
| CTGGTTC | 4.48 |
| TCTCTCC | 4.48 |
| GTGGACG | 4.48 |
| TCGGTCA | 4.48 |
| GTAGCTA | 4.47 |
| TTGCCAT | 4.47 |
| TGACCAC | 4.47 |
| TCCCATT | 4.47 |
| ATGGCAA | 4.47 |
| CCCGGCA | 4.46 |
| CGCCAGG | 4.46 |
| CTCCTTG | 4.46 |
| CTGGACT | 4.46 |
| AGACTGG | 4.46 |
| CAACGTA | 4.45 |
| CAAGGAC | 4.45 |
| ACCCTGT | 4.45 |
| CCGCGAG | 4.45 |
| GCAGTGG | 4.45 |
| TGGCAGT | 4.45 |
| CAGGAAG | 4.45 |
| GGAGGAT | 4.45 |
| GACCGGG | 4.45 |
| TTCCCGG | 4.45 |
| GTGGTCA | 4.45 |
| TTAGCTT | 4.44 |
| AAGCACG | 4.44 |
| CTGGGAA | 4.44 |
| CATCCTC | 4.43 |
| CCAGCAT | 4.43 |
| GTACTTG | 4.43 |
| TCCGTCT | 4.42 |
| GACGGGC | 4.42 |
| GGTGTGG | 4.42 |
| CGAGGGA | 4.42 |
| GTCCTGC | 4.42 |
| TAGCTGA | 4.41 |
| CGGCAGC | 4.41 |
| CTTCCTG | 4.41 |
| TCCCTAG | 4.41 |
| CCCCAAT | 4.40 |
| GTCCTTG | 4.40 |
| CAAGGAG | 4.40 |
| AACGGGG | 4.39 |
| GCAGGAC | 4.39 |
| GATCCCA | 4.39 |
| AGTCCGG | 4.39 |
| TGACTCC | 4.38 |
| CCTGCAT | 4.38 |
| CCGGACT | 4.38 |
| CTACAGG | 4.38 |
| CCGGTAA | 4.38 |
| GAGGATG | 4.38 |
| TATCCCT | 4.38 |
| CTCCCGA | 4.38 |
| CCTGCTT | 4.38 |
| GAGCGGG | 4.38 |
| GCCGCGA | 4.38 |
| ATTGGGG | 4.37 |
| ACACTGG | 4.37 |
| TGCGCGG | 4.37 |
| GGAGAGA | 4.37 |
| GGCCGCA | 4.37 |
| TGTGCCG | 4.37 |
| CCAGACC | 4.37 |
| CTCGGGA | 4.37 |
| CAACGGG | 4.37 |
| GAGGATC | 4.37 |
| GATCCCG | 4.37 |
| CCCGTAG | 4.36 |
| GGTGCTC | 4.36 |
| ACAGGGT | 4.36 |
| CGGCACA | 4.36 |
| TCACCGT | 4.36 |
| GGTGGCA | 4.36 |
| CGTCCAC | 4.36 |
| CTAGGGT | 4.36 |
| CGTCGCG | 4.36 |
| AAGCAGG | 4.36 |
| CGCCCGG | 4.35 |
| TCAGCTA | 4.35 |
| CACGCCG | 4.35 |
| AGACATC | 4.35 |
| CAACCAA | 4.34 |
| TGCCGGG | 4.34 |
| GAACTGC | 4.34 |
| TTACCGG | 4.34 |
| GGTCTGG | 4.34 |
| CGCCGCT | 4.34 |
| CCCGTAT | 4.34 |
| GTCGCCC | 4.34 |
| GATGGAC | 4.33 |
| CGCGCTC | 4.33 |
| CCGGTAT | 4.33 |
| GCACGCG | 4.33 |
| AAGCTAA | 4.33 |
| CTCCGCG | 4.33 |
| GTCCTAG | 4.33 |
| GATGTCT | 4.32 |
| GCGGGTG | 4.32 |
| AAGCAAG | 4.32 |
| CGAGCGC | 4.32 |
| CCTGCAG | 4.32 |
| CCAGTCT | 4.32 |
| ACGGTGA | 4.32 |
| TGGCGTG | 4.31 |
| GAACCGT | 4.31 |
| ACGGTTG | 4.31 |
| ATGCACC | 4.31 |
| CACCCGC | 4.31 |
| CGGCGCA | 4.31 |
| GCGGCAT | 4.31 |
| TTGGGTA | 4.30 |
| TACGGCC | 4.30 |
| CGCGGAC | 4.30 |
| CGCGCGT | 4.30 |
| TCCGGCC | 4.30 |
| CGTGGAC | 4.30 |
| GCGCGAG | 4.30 |
| GGTCCGA | 4.30 |
| AGAGCTC | 4.29 |
| AGGCGGC | 4.29 |
| ATGCTCG | 4.29 |
| GTCCACG | 4.29 |
| GTTCCTT | 4.28 |
| CCGCTCG | 4.28 |
| AATGTGG | 4.28 |
| ACCGTAC | 4.28 |
| CCTCCAA | 4.28 |
| CTGCAGG | 4.28 |
| CGGCGCT | 4.28 |
| TACGTTG | 4.28 |
| TCCCAAG | 4.28 |
| CCGGGCG | 4.28 |
| CGTCCAT | 4.27 |
| AGCCGCG | 4.27 |
| TACCCAA | 4.27 |
| ATGCAGG | 4.27 |
| CCCCGTT | 4.27 |
| AGGCGTT | 4.27 |
| TCCCTAC | 4.27 |
| CGCCGTC | 4.27 |
| TTGCCAA | 4.27 |
| ACCGTGC | 4.27 |
| TAGCAAG | 4.27 |
| TACGGGG | 4.26 |
| GTTCCTC | 4.26 |
| AACCCAG | 4.26 |
| CTCGCGC | 4.26 |
| GCGCGAC | 4.26 |
| CCACATT | 4.26 |
| TGGGATC | 4.26 |
| GTCCGCG | 4.26 |
| CTAGGAC | 4.26 |
| TGCGTCC | 4.26 |
| TTCCCAC | 4.26 |
| CAAGTAC | 4.26 |
| GCAGTTC | 4.25 |
| CTCCTCG | 4.25 |
| TCGGACC | 4.25 |
| TAGCTGT | 4.25 |
| ATAGCTG | 4.25 |
| TTGGTTG | 4.25 |
| GTGGGAA | 4.25 |
| CTCCTGG | 4.25 |
| AGTCTCC | 4.25 |
| CTGGGTT | 4.25 |
| CAGCTAT | 4.25 |
| CGAGGAG | 4.25 |
| ATGGACG | 4.25 |
| CCTGTAG | 4.24 |
| GCTGCCG | 4.24 |
| ATACCAC | 4.24 |
| CTTCTCC | 4.24 |
| AGACAGG | 4.24 |
| ACTCCTA | 4.24 |
| TGAGCTA | 4.24 |
| TTAGCTG | 4.24 |
| CCTGGCG | 4.24 |
| CTTGCTT | 4.24 |
| TCGGGAG | 4.24 |
| CGGCGAC | 4.24 |
| GGGCGCA | 4.23 |
| CCCGTCA | 4.23 |
| AGCGGAC | 4.23 |
| ACAGCTA | 4.23 |
| CCGGGAA | 4.23 |
| GTGGAGT | 4.23 |
| GCCGTCG | 4.23 |
| ACAGGGA | 4.23 |
| GTAGGGT | 4.23 |
| ACTCCTG | 4.23 |
| GTCCTCG | 4.22 |
| GTCCATC | 4.22 |
| CAGCTAA | 4.22 |
| GGTGCAT | 4.22 |
| CACCCGT | 4.22 |
| ACCCTTC | 4.22 |
| TAGCTCA | 4.22 |
| CAACGAG | 4.22 |
| ACGCCGA | 4.22 |
| AGGGATA | 4.21 |
| TGAGGGT | 4.21 |
| CGCCGAC | 4.21 |
| CAGGAGT | 4.21 |
| GGCGGGA | 4.21 |
| CCAGGAG | 4.21 |
| CTTGCTA | 4.21 |
| GGCGGGT | 4.21 |
| CATCCTT | 4.21 |
| CGTGGGT | 4.21 |
| TTGGTAT | 4.21 |
| ACCCTAG | 4.21 |
| TTGGCAA | 4.21 |
| TCCCGGC | 4.20 |
| CGCCGGT | 4.20 |
| ACGCGCC | 4.20 |
| CCTGCGG | 4.20 |
| AAGCTCT | 4.20 |
| CTGCTGG | 4.20 |
| TAGCAGG | 4.20 |
| TGGCGTT | 4.20 |
| CCAGTGT | 4.20 |
| GAGCTAA | 4.20 |
| GTCGCCG | 4.20 |
| ATCCCAT | 4.19 |
| ACCCTCA | 4.19 |
| AGACGGT | 4.19 |
| CCCGCTG | 4.19 |
| CGTGTTG | 4.19 |
| CCTGGTA | 4.19 |
| CCTGCTA | 4.19 |
| CACCTGG | 4.19 |
| GTGGTAT | 4.19 |
| TCCCTCT | 4.19 |
| CTTGGGA | 4.19 |
| ACGGTTC | 4.19 |
| ACCCTAC | 4.19 |
| AAGGAAC | 4.19 |
| CTCCGCC | 4.19 |
| TTAGCTC | 4.18 |
| GTAGGGA | 4.18 |
| TCCCTGT | 4.18 |
| TCCGTTC | 4.18 |
| CGGGATC | 4.18 |
| ATGGGAT | 4.18 |
| TGCCTGC | 4.18 |
| CTGCATT | 4.18 |
| ACCCTCT | 4.18 |
| GAGCTTT | 4.18 |
| CCCCGCT | 4.18 |
| CCAGCAG | 4.17 |
| CCGGTTA | 4.17 |
| GTCCTTA | 4.17 |
| ACCCATC | 4.17 |
| TTGGAGG | 4.17 |
| GAGGAAC | 4.17 |
| GGTCAGG | 4.17 |
| GTACGCA | 4.17 |
| CGTCCGC | 4.17 |
| GCGGTGT | 4.17 |
| TACCAGG | 4.17 |
| GAAGGGT | 4.17 |
| CAGGAAC | 4.16 |
| GGTCTAG | 4.16 |
| AGTCCAC | 4.16 |
| TGGCAGA | 4.16 |
| GCCGCGT | 4.16 |
| TTTCCTT | 4.16 |
| GCGCATA | 4.16 |
| TCTGCCA | 4.16 |
| GAGCTCT | 4.16 |
| CGCGGCT | 4.15 |
| GGAGACT | 4.15 |
| GGAGAAG | 4.15 |
| CTAGGCT | 4.15 |
| TTGCGTC | 4.15 |
| GCACGGT | 4.15 |
| GGCGTAC | 4.15 |
| TATGCGG | 4.14 |
| CCCGACG | 4.14 |
| ATACCAA | 4.14 |
| AGCCTAG | 4.14 |
| TCGCCAC | 4.14 |
| GCCGCCT | 4.14 |
| CCTGTCT | 4.14 |
| GCACACC | 4.14 |
| ACTCCAC | 4.13 |
| CGGCGTG | 4.13 |
| TCCCACG | 4.13 |
| GGGCGAT | 4.13 |
| AGAGGGT | 4.13 |
| TAACCAT | 4.13 |
| CGACGGC | 4.13 |
| CCCGTTG | 4.13 |
| TCCCTCG | 4.13 |
| GTTCCTG | 4.13 |
| GGTCACC | 4.13 |
| CCCCGTA | 4.13 |
| ATACGGG | 4.13 |
| AGAGCTT | 4.12 |
| TCACTCC | 4.12 |
| TTGGTTC | 4.12 |
| TGACCGA | 4.12 |
| CCGGGTT | 4.12 |
| GTGGCGA | 4.12 |
| TAGCTTC | 4.12 |
| TAGGAGT | 4.12 |
| CTCCGTT | 4.11 |
| AGCGGCG | 4.11 |
| TACGCGG | 4.11 |
| AGAGGGA | 4.11 |
| GTGGTAA | 4.11 |
| TTCCATT | 4.11 |
| CAACCAC | 4.11 |
| AATGCAG | 4.11 |
| TTCCATG | 4.11 |
| GCCCGCA | 4.11 |
| AATGTAA | 4.11 |
| TATGCGT | 4.11 |
| GGACAAT | 4.10 |
| GCAGGCA | 4.10 |
| TGCGCCC | 4.10 |
| CCAGCGC | 4.10 |
| GCCGGGA | 4.10 |
| GAACCAA | 4.10 |
| ACGCCAA | 4.10 |
| CCGCGTG | 4.10 |
| CCGCTGG | 4.10 |
| ATGCACG | 4.09 |
| GGAGTGA | 4.09 |
| CGTGTGG | 4.09 |
| CGCGTGC | 4.09 |
| GCTCTCC | 4.09 |
| AACCCGG | 4.09 |
| GGACGAC | 4.08 |
| CACCACC | 4.08 |
| GCCCGTC | 4.08 |
| TTACCAA | 4.08 |
| CCAGGTG | 4.08 |
| ATACCGT | 4.08 |
| CCTGACC | 4.08 |
| AGAGTCC | 4.08 |
| CATGGAA | 4.08 |
| GAAGCTA | 4.08 |
| CACGGGT | 4.08 |
| GACGCCT | 4.08 |
| CGGCACT | 4.08 |
| AAGGAAA | 4.08 |
| GTACGAG | 4.07 |
| AAAGCTC | 4.07 |
| GTGGACT | 4.07 |
| ATGGTTA | 4.07 |
| ACCGCTA | 4.07 |
| AAGGATG | 4.07 |
| CAGCACA | 4.07 |
| CAACACG | 4.07 |
| CCACGGA | 4.06 |
| GACCAAG | 4.06 |
| TGCGGGC | 4.06 |
| CCTCTGG | 4.06 |
| TCTCCTA | 4.06 |
| CTGGCGA | 4.06 |
| GCCGTAC | 4.06 |
| ATTGTCC | 4.06 |
| TTGGTAA | 4.06 |
| GACGGCG | 4.06 |
| GTGCACA | 4.05 |
| TGGCGGC | 4.05 |
| CCACACG | 4.05 |
| TGGCAAC | 4.05 |
| TGTGCAC | 4.05 |
| CCGCATA | 4.05 |
| GTCCTTC | 4.05 |
| AGCCTGC | 4.05 |
| GAAGGAC | 4.04 |
| GATGGGT | 4.03 |
| GGCGTGG | 4.03 |
| CACCACG | 4.03 |
| GGAGAGC | 4.03 |
| CTCGGCG | 4.03 |
| CGTGCAT | 4.03 |
| TGTCCGG | 4.03 |
| ACCCACG | 4.03 |
| TTACATT | 4.03 |
| GTGCATC | 4.02 |
| CTAGACC | 4.02 |
| ACGGTAT | 4.02 |
| ACCGTGG | 4.02 |
| GTGGTTG | 4.02 |
| GTGCGCC | 4.02 |
| TGTGCTT | 4.02 |
| CCAGAGG | 4.02 |
| TGTGCTG | 4.01 |
| GGACTCT | 4.01 |
| ACACCGC | 4.01 |
| GATGCAC | 4.01 |
| TAAGGAC | 4.01 |
| GTGCTGC | 4.01 |
| GCACGCC | 4.01 |
| ACCCACA | 4.01 |
| AAACCTC | 4.01 |
| TAGGAGA | 4.01 |
| GGACGCT | 4.00 |
| ACACCGA | 4.00 |
| CTTGGTC | 4.00 |
| CCGCGTC | 4.00 |
| CTAGCAT | 3.99 |
| GGTGGTG | 3.99 |
| GTAGTGG | 3.99 |
| TGTGGGT | 3.99 |
| TTTGGGG | 3.99 |
| CCCCAAA | 3.99 |
| GGTGTGC | 3.98 |
| AACGCCT | 3.98 |
| CCAGCGG | 3.98 |
| GAGGTTT | 3.98 |
| CGACGCA | 3.98 |
| AGCCTTC | 3.98 |
| AACGCCG | 3.98 |
| GTACTAT | 3.98 |
| CGTGGGA | 3.97 |
| AATGGAA | 3.97 |
| CCGGACA | 3.97 |
| TATCCCG | 3.97 |
| TCGGTGT | 3.97 |
| ACCCGTG | 3.97 |
| ACCCTTA | 3.97 |
| CGAGCAT | 3.97 |
| GAACCGA | 3.97 |
| TAACCGG | 3.96 |
| GCACTAC | 3.96 |
| GGCCGCT | 3.96 |
| AAGCACA | 3.96 |
| GCAGGCT | 3.96 |
| TGGCGCG | 3.96 |
| GGACTAA | 3.96 |
| TTACATG | 3.96 |
| GCGGTAG | 3.96 |
| CACGGGA | 3.96 |
| ATAGTAC | 3.95 |
| CGTCGCC | 3.95 |
| CTCCTGC | 3.95 |
| CACCAAG | 3.94 |
| GCGCAGG | 3.94 |
| GTAGGAC | 3.94 |
| TTACCAC | 3.94 |
| CCCGCTC | 3.94 |
| AGCCTTG | 3.94 |
| AACGGAG | 3.94 |
| TAGCACG | 3.94 |
| ATGCTAG | 3.94 |
| GACGGAG | 3.94 |
| CACGCCA | 3.94 |
| CAAGGCT | 3.94 |
| CTACCGC | 3.93 |
| GACGCGG | 3.93 |
| GAACCAC | 3.93 |
| AGTGCCG | 3.93 |
| CCTGCGC | 3.93 |
| CGCGAGG | 3.93 |
| CACCCGA | 3.93 |
| CTACGTG | 3.93 |
| GTGCTCG | 3.93 |
| CGAGGCT | 3.93 |
| CGAGGAC | 3.93 |
| CGTGCTA | 3.93 |
| TCACGGG | 3.92 |
| GTACGCC | 3.92 |
| GTGCAAG | 3.92 |
| CTACTGG | 3.92 |
| CCCGGTA | 3.92 |
| CCCGTGA | 3.92 |
| GGCGCGT | 3.92 |
| CCGCAGG | 3.92 |
| CAACACA | 3.92 |
| TCGCCAG | 3.92 |
| CGGCGAG | 3.92 |
| ACGCATA | 3.92 |
| ACCGTCT | 3.91 |
| GGTCGCC | 3.91 |
| CCACGTC | 3.91 |
| GAAGGCT | 3.91 |
| AACCTCC | 3.91 |
| TCCCAGC | 3.91 |
| GTGGTTC | 3.91 |
| ACTCGGG | 3.91 |
| TCGGTCG | 3.91 |
| CAAGGCA | 3.91 |
| CGCGCTA | 3.90 |
| GTCCGCC | 3.90 |
| AGCCACC | 3.90 |
| GAACGTA | 3.90 |
| GTGCTAC | 3.90 |
| GTAGCAC | 3.90 |
| TAAGGGT | 3.90 |
| TGCCTTG | 3.90 |
| TACCCGC | 3.90 |
| GTACGGT | 3.89 |
| ACCGTCG | 3.89 |
| TGTCCAA | 3.89 |
| ACCCGAC | 3.89 |
| TGTGTTG | 3.89 |
| CCAGCAC | 3.89 |
| AGCCTAC | 3.89 |
| GATGGCT | 3.88 |
| GGAGGTT | 3.88 |
| GCAGGAG | 3.88 |
| TAAGCAT | 3.88 |
| CCACTAC | 3.88 |
| ATCCGCC | 3.88 |
| ATCCGGG | 3.88 |
| TGACGGT | 3.88 |
| GGTGGCT | 3.88 |
| GAACCGC | 3.88 |
| TGCGTAC | 3.88 |
| CATGTAA | 3.88 |
| CTACGGT | 3.87 |
| GCCGTGC | 3.87 |
| AGCGCCG | 3.87 |
| ACGCGGC | 3.87 |
| TGCCTCA | 3.87 |
| CGGCGAA | 3.87 |
| AGTGGGT | 3.87 |
| AAACCTG | 3.87 |
| CATCATA | 3.87 |
| AGAGCTG | 3.87 |
| GTCCTAC | 3.87 |
| TGCGGCC | 3.86 |
| TACGCGC | 3.86 |
| CTGGTTA | 3.86 |
| GACCACA | 3.86 |
| GGCGGAG | 3.86 |
| CCCGGAT | 3.86 |
| GACCACC | 3.86 |
| TAACCAG | 3.86 |
| AGACGTA | 3.86 |
| ATGCTTA | 3.86 |
| CAACGTG | 3.85 |
| GCTGGGA | 3.85 |
| ACCCGGT | 3.85 |
| GTTGCCA | 3.85 |
| CTTGGTG | 3.85 |
| GACGCGA | 3.85 |
| TTAGTCC | 3.85 |
| GTGCTAG | 3.85 |
| TCCCGTG | 3.85 |
| ATCCATG | 3.85 |
| GAACGGG | 3.85 |
| ATGCTGC | 3.85 |
| CTTCTTC | 3.85 |
| AGCCATC | 3.85 |
| ATGGGAA | 3.85 |
| CATGACC | 3.85 |
| ACTCCAT | 3.85 |
| GTACTAA | 3.85 |
| GGTGACC | 3.85 |
| CGTGGTG | 3.85 |
| TTCCCAT | 3.85 |
| CCTCGCG | 3.84 |
| GCAGACC | 3.84 |
| CTCCGTA | 3.84 |
| GCCGTGG | 3.84 |
| CAGCTCT | 3.84 |
| GGACTTA | 3.84 |
| GTGCTGG | 3.84 |
| TGGCGCC | 3.84 |
| ACCGGGT | 3.84 |
| AACCCAC | 3.84 |
| AGCGTCC | 3.84 |
| ATGGAGT | 3.83 |
| ATGCGTG | 3.83 |
| CTAGTGG | 3.83 |
| GATCCTT | 3.83 |
| CTAGCAC | 3.83 |
| ACACAGG | 3.83 |
| CTTGCAC | 3.83 |
| CCTCCGT | 3.83 |
| CAGGTTT | 3.83 |
| ACGGTAA | 3.83 |
| GGTGCTG | 3.83 |
| ATGCGCC | 3.83 |
| CTCCTTT | 3.83 |
| TCGGACG | 3.83 |
| CGTGTCT | 3.83 |
| GCTCCAA | 3.82 |
| TCGGCAG | 3.82 |
| TTGGCGT | 3.82 |
| GATCCTG | 3.82 |
| TCGGTTC | 3.82 |
| ACCCACT | 3.82 |
| GTGGGTT | 3.82 |
| GCCGGAG | 3.82 |
| AGACACG | 3.82 |
| GCCCGCT | 3.82 |
| TACGTCT | 3.82 |
| CAGCACC | 3.82 |
| ACCCAGC | 3.82 |
| GGTCGTG | 3.81 |
| TGCGGCG | 3.81 |
| GCGCTGG | 3.81 |
| ACCCAAG | 3.81 |
| GTCCAGT | 3.81 |
| AGACGTG | 3.81 |
| CCACTAG | 3.81 |
| TCCCATC | 3.81 |
| AGTCCAT | 3.81 |
| TGCCGCG | 3.81 |
| CCAGTAG | 3.81 |
| TCGGGTG | 3.81 |
| GCTCGGG | 3.81 |
| CCTGTGT | 3.81 |
| GGCGCAC | 3.81 |
| TGACCGC | 3.81 |
| TTGGACA | 3.80 |
| CTGCCGA | 3.80 |
| CTTGGAC | 3.80 |
| TTAGTAC | 3.80 |
| ACCGGTA | 3.80 |
| CCCGTTA | 3.80 |
| CAGCGGG | 3.80 |
| CATGGAT | 3.80 |
| TTGGACG | 3.80 |
| TAACCAA | 3.80 |
| CACGTTG | 3.80 |
| ACACGGT | 3.80 |
| GTCGCGC | 3.80 |
| GGTCTGC | 3.80 |
| CATCCAG | 3.80 |
| CCCGCAA | 3.80 |
| TTTGGGC | 3.80 |
| CTGGAGA | 3.80 |
| AGACGGC | 3.80 |
| AGCGGCC | 3.79 |
| CTAGTGC | 3.79 |
| CGGGATA | 3.79 |
| ACTGGAC | 3.79 |
| ATCGCCC | 3.79 |
| CCACTCG | 3.79 |
| TCTCCAG | 3.79 |
| GCCCAAA | 3.79 |
| GACCGCG | 3.78 |
| GTCCTGT | 3.78 |
| AGGCGAG | 3.78 |
| TCGGTAG | 3.78 |
| GATGTCG | 3.78 |
| TGAGGGA | 3.78 |
| ATCGCCA | 3.78 |
| TCGGTGA | 3.78 |
| TGCCTAG | 3.78 |
| GTTCCAC | 3.78 |
| TCTCTCT | 3.78 |
| CGGCAGT | 3.78 |
| TCACATG | 3.78 |
| GGTCATG | 3.78 |
| AAACCTA | 3.78 |
| GTCGTCC | 3.77 |
| GCAGCAC | 3.77 |
| TACGTTC | 3.77 |
| CTAGGCA | 3.77 |
| CCACGGC | 3.77 |
| AACGGCG | 3.77 |
| GCACACA | 3.77 |
| ATCCCAA | 3.77 |
| TTGCGTA | 3.77 |
| AGACGCC | 3.77 |
| TATGATG | 3.77 |
| CCGCGAC | 3.77 |
| GTCCAAG | 3.76 |
| TCCCTCA | 3.76 |
| TGTGTGC | 3.76 |
| TAAGTCC | 3.76 |
| TAGGTTT | 3.76 |
| CGCCACC | 3.76 |
| TGAGGCA | 3.76 |
| CGCCGAG | 3.76 |
| CGACAGG | 3.76 |
| CGCCGAT | 3.76 |
| TGACGTG | 3.76 |
| GCCGCCA | 3.76 |
| AGCCTCG | 3.76 |
| GGACGAT | 3.76 |
| GTAGTGC | 3.76 |
| GCACGGC | 3.76 |
| AAAGGAG | 3.76 |
| GGCCGAC | 3.76 |
| GTCGGCC | 3.76 |
| CGTGGTC | 3.76 |
| CCACGGT | 3.76 |
| GGAGTAA | 3.75 |
| CTTGGGT | 3.75 |
| AATCCCA | 3.75 |
| TTGGGAT | 3.75 |
| GTAGGCT | 3.75 |
| CATGTGA | 3.75 |
| TCTCCAC | 3.75 |
| GGCGCCA | 3.75 |
| TATGCGC | 3.75 |
| ATGCTAC | 3.74 |
| GTAGGCA | 3.74 |
| GACCGTG | 3.74 |
| CGTCCAA | 3.74 |
| GCACGTC | 3.74 |
| TACGGAG | 3.74 |
| CTACCGA | 3.74 |
| TCACCGA | 3.74 |
| CTCGCCT | 3.74 |
| ATTCCCT | 3.74 |
| CCCGAGC | 3.74 |
| TTGGTTA | 3.74 |
| CTACGCC | 3.73 |
| ACAGGAC | 3.73 |
| GTGGAAC | 3.73 |
| CGCCATG | 3.73 |
| GATGGGA | 3.73 |
| GTCCACT | 3.73 |
| AGCGGGG | 3.73 |
| TGTGGTC | 3.73 |
| ACTGCCG | 3.73 |
| GAACGTT | 3.73 |
| TCGGGTA | 3.73 |
| GTCCACA | 3.73 |
| GCACAGT | 3.72 |
| ACACGTG | 3.72 |
| GCTGGGT | 3.72 |
| TCGCCAA | 3.72 |
| ACCCGAG | 3.72 |
| GTCGGGT | 3.72 |
| CAGCTTT | 3.72 |
| GCACTAG | 3.72 |
| TCCGTGG | 3.72 |
| TGTGGAC | 3.72 |
| TGCCTAC | 3.71 |
| CCACATC | 3.71 |
| CGAGCAC | 3.71 |
| TATCCCA | 3.71 |
| TACGCCG | 3.71 |
| CACGTAG | 3.71 |
| CAACAAG | 3.71 |
| CGTGGCA | 3.71 |
| CTCGTAC | 3.71 |
| GTAGCAT | 3.71 |
| CCCGTTC | 3.71 |
| CATCTCC | 3.70 |
| TGGCAAT | 3.70 |
| TTCGTCC | 3.70 |
| TTACCGT | 3.70 |
| ACTGTGC | 3.70 |
| TAAGTAC | 3.70 |
| TCCCAGT | 3.69 |
| GACCACG | 3.69 |
| CAGCGGC | 3.69 |
| CACGACC | 3.69 |
| GTCCTCA | 3.69 |
| TTACTCC | 3.69 |
| ATGCATC | 3.69 |
| TAAGGGA | 3.69 |
| GGAGTAT | 3.69 |
| ATTGCCA | 3.69 |
| AACGCGG | 3.69 |
| CGAGTCT | 3.69 |
| TCCCGGA | 3.69 |
| CAGGATC | 3.68 |
| GTTCCAT | 3.68 |
| CCTCAGG | 3.68 |
| GTCGGCG | 3.68 |
| GATGTGC | 3.68 |
| GCAGCAT | 3.68 |
| AGCCTCA | 3.68 |
| ACTCTCC | 3.68 |
| TGAGGAC | 3.68 |
| CATGCAA | 3.68 |
| CTGGATG | 3.68 |
| GCACATC | 3.68 |
| ACCGGCG | 3.68 |
| GTTGCCG | 3.67 |
| GTTCCTA | 3.67 |
| CTGCACC | 3.67 |
| GTACGAA | 3.67 |
| AACGCCA | 3.67 |
| TTGGAGC | 3.67 |
| GCACAAG | 3.67 |
| TTCGTAC | 3.67 |
| ACCGCCT | 3.66 |
| CCAGTCG | 3.66 |
| GTGGAGA | 3.66 |
| TGAGGCT | 3.66 |
| TGACGCG | 3.66 |
| CATCCTA | 3.66 |
| GACGCAA | 3.66 |
| TTGCCGT | 3.65 |
| GCACGGA | 3.65 |
| TAGGATG | 3.65 |
| CTTCGCC | 3.65 |
| GATGTGG | 3.65 |
| CTTGTTC | 3.65 |
| GAGCGCC | 3.65 |
| GGTGGTC | 3.65 |
| ACAGTGG | 3.65 |
| TGGGATT | 3.65 |
| GTGCTGT | 3.65 |
| TGGCGGT | 3.65 |
| ACGGCAA | 3.65 |
| CTACGCA | 3.65 |
| ATGGAAC | 3.64 |
| CGCCGCA | 3.64 |
| ATGCTCA | 3.64 |
| TGCCACT | 3.64 |
| TTTCCCT | 3.64 |
| AGGCGGT | 3.64 |
| ATACTCC | 3.64 |
| CTTCCTA | 3.64 |
| GGCGCTC | 3.64 |
| GATGGCA | 3.64 |
| AAGGATC | 3.64 |
| TAACCGT | 3.64 |
| CTTGTGC | 3.64 |
| AGTGGCA | 3.64 |
| AAAGCTG | 3.64 |
| ACGGAGG | 3.64 |
| CTCGTTG | 3.64 |
| AGCCAAG | 3.63 |
| ACTGGGA | 3.63 |
| ATGGACT | 3.63 |
| TGGCGTC | 3.63 |
| GACGCAT | 3.63 |
| ACCGTCA | 3.63 |
| CGCGCAG | 3.63 |
| ACAGGCT | 3.63 |
| TAGGAAG | 3.63 |
| ATCGTCC | 3.63 |
| TACCCGA | 3.63 |
| CACCACA | 3.63 |
| CCTGTCG | 3.63 |
| ACAGCAC | 3.63 |
| GAGCAGC | 3.62 |
| ACGCGCG | 3.62 |
| TGCCACG | 3.62 |
| CTTGGCT | 3.62 |
| ACAGGAG | 3.62 |
| TTGCATG | 3.62 |
| GTACTTA | 3.62 |
| TGCGCCG | 3.62 |
| AGCCTTA | 3.62 |
| TGCCATC | 3.62 |
| TGAGCAT | 3.62 |
| GGAGAGT | 3.62 |
| GAGCACT | 3.62 |
| GTCCGTG | 3.62 |
| CCTGAGG | 3.62 |
| AGGGAAT | 3.62 |
| GAACACA | 3.62 |
| CCACTGT | 3.61 |
| TGTCGGG | 3.61 |
| GCAGAGG | 3.61 |
| ACGGCGT | 3.61 |
| CCCGAGT | 3.61 |
| AGGCGCG | 3.61 |
| TCCGGGA | 3.61 |
| CGACATC | 3.61 |
| TAGGAAC | 3.61 |
| TCGGCGT | 3.61 |
| TTTGTCC | 3.61 |
| TCCCGGT | 3.60 |
| CTCCAGA | 3.60 |
| ATGCGGT | 3.60 |
| CATGGCG | 3.60 |
| CCTGTCA | 3.60 |
| CGACGAG | 3.60 |
| TGACGTA | 3.60 |
| TATGACC | 3.60 |
| ATCCCGC | 3.60 |
| GTCCGGC | 3.60 |
| GCTGGAC | 3.60 |
| CGGCAAC | 3.60 |
| GGACAAA | 3.60 |
| TCCCTTA | 3.60 |
| GGAGATG | 3.60 |
| GACGCGC | 3.60 |
| GTCCTCT | 3.60 |
| CGTGCGG | 3.60 |
| GAACAAG | 3.60 |
| GATGCAT | 3.60 |
| AGAGGAC | 3.60 |
| TGTGTTC | 3.60 |
| GTCCAGC | 3.59 |
| GGCGACG | 3.59 |
| CCACGTA | 3.59 |
| CGCGCTG | 3.59 |
| GGCGAAG | 3.59 |
| AGACTCG | 3.59 |
| AGGGAAA | 3.59 |
| GAAGAAG | 3.59 |
| TGACAGG | 3.59 |
| CGGCAGA | 3.59 |
| TTGGCGA | 3.58 |
| GGTGCAG | 3.58 |
| GCTGCTC | 3.58 |
| GCCGCTG | 3.58 |
| CATCCAC | 3.58 |
| GACCTGC | 3.58 |
| ACACACA | 3.58 |
| ACGGTTA | 3.58 |
| TGTGGGA | 3.58 |
| TCTGGAG | 3.58 |
| TAGCGGT | 3.58 |
| TCACCGC | 3.58 |
| AGTGCTC | 3.58 |
| CGCCTGG | 3.58 |
| GGTCATA | 3.58 |
| CCAGGCG | 3.57 |
| AGGCGTG | 3.57 |
| ACCCAGT | 3.57 |
| AGTGGAC | 3.57 |
| ATAGCTA | 3.57 |
| AGCCACG | 3.57 |
| TCAGGGT | 3.57 |
| GCAGCGC | 3.57 |
| TCCCTTT | 3.57 |
| ATGCTTC | 3.57 |
| CTCCAAG | 3.57 |
| GCGGACG | 3.57 |
| CTGCACT | 3.56 |
| TGTGTGT | 3.56 |
| TAAGGAG | 3.56 |
| TCCCACA | 3.56 |
| TAGCTAT | 3.56 |
| CCCGACA | 3.56 |
| TGTGGTG | 3.56 |
| CTCCTGT | 3.56 |
| CGTGGCT | 3.56 |
| GTCGCCA | 3.56 |
| CATCCAT | 3.56 |
| GGCGGAT | 3.56 |
| CCGCGTT | 3.55 |
| GTGCTTC | 3.55 |
| GAACACG | 3.55 |
| TGGCGAC | 3.55 |
| CGAGGCA | 3.55 |
| CCGCGCC | 3.55 |
| GCGGTCA | 3.55 |
| AGCCGGC | 3.55 |
| TAGCTAA | 3.55 |
| CCGCATG | 3.55 |
| AGCGTCG | 3.55 |
| TAGCACA | 3.55 |
| AGCCTGT | 3.55 |
| AGCCGCC | 3.54 |
| GTTCCAG | 3.54 |
| GGTCGCG | 3.54 |
| TGGCGTA | 3.54 |
| GGTGGCG | 3.54 |
| TGGGATA | 3.54 |
| TGACACG | 3.54 |
| GCACGTA | 3.54 |
| CCTCTGC | 3.54 |
| ACCGGGA | 3.54 |
| AACGTTC | 3.54 |
| CTTGGAG | 3.54 |
| GTGCTCA | 3.54 |
| ATGCGTC | 3.53 |
| AACCCAT | 3.53 |
| TTGCGAT | 3.53 |
| ATTCCCG | 3.53 |
| CAACGGT | 3.53 |
| GTCCGTC | 3.53 |
| TAAGGCT | 3.53 |
| CGCCTGT | 3.53 |
| CCGCGTA | 3.53 |
| AGCCTCT | 3.53 |
| ACTGGGT | 3.53 |
| CAGCGTG | 3.52 |
| GTTCTCC | 3.52 |
| ATGCAAG | 3.52 |
| CCCGTAA | 3.52 |
| AGAGAGA | 3.52 |
| TCCCTGA | 3.52 |
| TAAGCAC | 3.52 |
| TGCCGGC | 3.52 |
| AGACACA | 3.52 |
| TGCCTCG | 3.52 |
| CACGTCT | 3.52 |
| GAAGCAC | 3.52 |
| CGAGTGC | 3.51 |
| ATGGGTT | 3.51 |
| GCCGGCA | 3.51 |
| GCACGTT | 3.51 |
| GACGTGC | 3.51 |
| TAACGGG | 3.51 |
| ACAGGCG | 3.51 |
| CAACAGG | 3.51 |
| AAGCACC | 3.50 |
| CTTGTTG | 3.50 |
| GGCGTCT | 3.50 |
| TCAGGGA | 3.50 |
| GCCGTCT | 3.50 |
| CAGCACT | 3.50 |
| TGTGCTA | 3.50 |
| GGAGTTG | 3.50 |
| CTCGGGT | 3.50 |
| ATACGGC | 3.50 |
| TATCCTT | 3.50 |
| CTCCTTA | 3.49 |
| CGTCGGA | 3.49 |
| CCTCGCC | 3.49 |
| GCCGGAC | 3.49 |
| GCGGCAA | 3.49 |
| TCCCGTC | 3.49 |
| TCGGCAA | 3.49 |
| ACCGCTC | 3.49 |
| TTAGCTA | 3.49 |
| TCACGTG | 3.49 |
| GGCGCGG | 3.49 |
| GGCGGAC | 3.49 |
| GCGCTGC | 3.49 |
| GGCGGCA | 3.49 |
| CTACACG | 3.48 |
| GAAGGCA | 3.48 |
| AGAGGCT | 3.48 |
| TTCGGCC | 3.48 |
| ACGCGAC | 3.48 |
| GATGTCA | 3.48 |
| CGCCTTG | 3.48 |
| CCTGTTG | 3.48 |
| ACCGTGT | 3.48 |
| TTGCCGC | 3.48 |
| GCAGGTC | 3.48 |
| GTCCTTT | 3.47 |
| AAAGGGA | 3.47 |
| TGAGCAC | 3.47 |
| AATGTGA | 3.47 |
| CACGGAC | 3.47 |
| CATGCGC | 3.47 |
| GTGCGGC | 3.47 |
| TGACTGG | 3.47 |
| GGTGCTT | 3.47 |
| ACCGCCA | 3.47 |
| CAACGTT | 3.46 |
| GAAGCAT | 3.46 |
| GCCGACC | 3.46 |
| GCCGTCA | 3.46 |
| GCGCATG | 3.46 |
| ACGGGTA | 3.46 |
| CTTGCAT | 3.45 |
| TTTCCTC | 3.45 |
| CACCACT | 3.45 |
| TTGCATT | 3.45 |
| TGTGTGG | 3.45 |
| CTCCTAG | 3.45 |
| TATCCTC | 3.45 |
| CCCGCTT | 3.45 |
| CGCGACC | 3.45 |
| TGCCTTC | 3.45 |
| TGTGCAT | 3.45 |
| CTAGAGG | 3.45 |
| TTGCCGA | 3.45 |
| ATGCTGT | 3.45 |
| CTTGTGG | 3.44 |
| GCGCACC | 3.44 |
| CTGCTCG | 3.44 |
| CTAGGAG | 3.44 |
| TACGTGG | 3.44 |
| ACCCTGA | 3.44 |
| AGTGCAG | 3.44 |
| GTGGATG | 3.44 |
| GCAGCGG | 3.44 |
| CAACTCC | 3.44 |
| ACAGCAT | 3.44 |
| CCACACA | 3.43 |
| TCACATT | 3.43 |
| AGTGCTG | 3.43 |
| AGGCGAC | 3.43 |
| TGCCGAG | 3.43 |
| ATGGATG | 3.43 |
| TACGGGT | 3.43 |
| TGGCAAA | 3.43 |
| ACGCTGG | 3.43 |
| TGTGTCT | 3.43 |
| AGCGTAC | 3.43 |
| GACCGCC | 3.42 |
| CGTGTAG | 3.42 |
| ATCGCCG | 3.42 |
| CCAGTCA | 3.42 |
| CTCCTCA | 3.42 |
| CGACGCT | 3.42 |
| GACGTGG | 3.42 |
| GCCGACG | 3.42 |
| TGACATC | 3.42 |
| GTCCGCT | 3.42 |
| GTACTCT | 3.42 |
| ACAGTGC | 3.41 |
| CCACAAG | 3.41 |
| GGACGAA | 3.41 |
| ACGCCGT | 3.41 |
| CTCCGGC | 3.41 |
| CTACTCG | 3.41 |
| CCTCGGC | 3.41 |
| AGAGTAC | 3.41 |
| CGACGGT | 3.41 |
| AAGCGGG | 3.41 |
| GCGGTTC | 3.41 |
| GTCGGGA | 3.41 |
| ATGCACA | 3.41 |
| AGGCGGA | 3.41 |
| GTGCTTA | 3.41 |
| TAACCAC | 3.40 |
| ACCGGAG | 3.40 |
| CTGGAAC | 3.40 |
| AGGCGTA | 3.40 |
| GAAGTCC | 3.40 |
| CCCCGAA | 3.40 |
| GGTCGTC | 3.40 |
| TGCCTAT | 3.39 |
| CCTCTAG | 3.39 |
| GTGCGTT | 3.39 |
| GATCATA | 3.39 |
| GCACTGT | 3.39 |
| AATGCAA | 3.39 |
| GGTGCGC | 3.39 |
| TCGGCGA | 3.39 |
| CCCGGAA | 3.39 |
| CGTGTTC | 3.38 |
| CGCGCCA | 3.38 |
| CACCGCC | 3.38 |
| ATAGGCA | 3.38 |
| GAGGAAA | 3.38 |
| GTGGTTA | 3.38 |
| TGCCAAG | 3.38 |
| CAAGGCG | 3.38 |
| CGCCTGC | 3.38 |
| TGAGGAG | 3.38 |
| GCCGGCT | 3.38 |
| TTTGCCA | 3.38 |
| CACCTGT | 3.38 |
| AAGGATA | 3.38 |
| TCGCCGT | 3.37 |
| CAACGGC | 3.37 |
| ACGGACG | 3.37 |
| CTCCTAC | 3.37 |
| TTCCCAA | 3.37 |
| CGTGACC | 3.37 |
| GGACTTC | 3.37 |
| CACGCAT | 3.36 |
| AGACGTT | 3.36 |
| GCAGGCG | 3.36 |
| TTGCGGG | 3.36 |
| GGCGTGC | 3.36 |
| TCTCCGG | 3.36 |
| ATAGGGT | 3.36 |
| GCGCACG | 3.36 |
| TCTGCCG | 3.36 |
| GAGCGGT | 3.36 |
| TTCCCGC | 3.36 |
| CGACTGG | 3.36 |
| GGCGGCT | 3.36 |
| GACGCCA | 3.36 |
| TCGCCAT | 3.36 |
| AGTGGTG | 3.35 |
| TATGATC | 3.35 |
| CCTCGTG | 3.35 |
| CACCTGC | 3.35 |
| ACAGGTG | 3.35 |
| GTCCTGA | 3.35 |
| GTGCAGC | 3.35 |
| CCAGCGT | 3.35 |
| GGCGTCG | 3.34 |
| CCGCTGC | 3.34 |
| ATACGTG | 3.34 |
| ACCGTAG | 3.34 |
| GTAGGAG | 3.34 |
| AGACGTC | 3.34 |
| CTTCCAG | 3.34 |
| GCGGGTA | 3.33 |
| TACCCGT | 3.33 |
| CTTGGCA | 3.33 |
| CCACAGC | 3.33 |
| GATCCTA | 3.33 |
| AGACGCA | 3.33 |
| GTGCGCA | 3.33 |
| GCGGTTG | 3.33 |
| CATGCGG | 3.33 |
| ATTCCTT | 3.33 |
| TACGTCA | 3.33 |
| TACCATC | 3.33 |
| ACCGTTA | 3.33 |
| CGTGCAG | 3.32 |
| GCGGTGA | 3.32 |
| TCCGCGT | 3.32 |
| GGTCTAC | 3.32 |
| TCAGGAC | 3.32 |
| AGAGGCA | 3.32 |
| GAACGGC | 3.32 |
| AGCCTTT | 3.32 |
| TTGGGAA | 3.32 |
| GCCGTTG | 3.32 |
| ACCCTAT | 3.32 |
| CACGCCT | 3.32 |
| CACGCGG | 3.32 |
| CTAGGTC | 3.31 |
| GGTCACG | 3.31 |
| ACGGAGC | 3.31 |
| AGTCGGG | 3.31 |
| CCACGTT | 3.31 |
| AACGTCT | 3.31 |
| TCCGTTG | 3.31 |
| CGGGAAA | 3.31 |
| TGCCGTG | 3.31 |
| GCAGGTG | 3.31 |
| TGCCAGC | 3.31 |
| CCACGAG | 3.31 |
| ATGCTGA | 3.31 |
| AGCCACT | 3.30 |
| CGCGTGG | 3.30 |
| GTAGGTC | 3.30 |
| GCCGCAC | 3.30 |
| GAGCAAC | 3.30 |
| GGAGAAC | 3.30 |
| CTGCTTG | 3.30 |
| GACGTCG | 3.30 |
| ACTCCGG | 3.30 |
| CTGCACG | 3.30 |
| ACGCGTC | 3.30 |
| AGCCGGA | 3.30 |
| GCGGAGC | 3.30 |
| CGAGTAG | 3.30 |
| AGTGCAC | 3.30 |
| CGCCACT | 3.29 |
| AACCGGT | 3.29 |
| AACGTCG | 3.29 |
| GAGCGGC | 3.29 |
| CACGTGA | 3.29 |
| CTGGAAG | 3.29 |
| CACCGCA | 3.29 |
| CTGCGTG | 3.29 |
| GACGGAC | 3.29 |
| CCGGAGT | 3.29 |
| TGGCGCA | 3.29 |
| GAACAGG | 3.29 |
| AAAGGGT | 3.29 |
| GACGTAG | 3.29 |
| AGACGAC | 3.29 |
| TAGGATC | 3.29 |
| CGACGTT | 3.28 |
| ACCCTTT | 3.28 |
| GAGCGTA | 3.28 |
| AGCGGGC | 3.28 |
| ACCCGTA | 3.28 |
| CACGCTG | 3.28 |
| GCGCGAT | 3.28 |
| TGACGTT | 3.28 |
| TACGTGC | 3.28 |
| TCCCGAC | 3.28 |
| CAACGGA | 3.28 |
| GACCAGC | 3.28 |
| AGTCCAA | 3.28 |
| TACGCCT | 3.27 |
| GGAGAAA | 3.27 |
| TCAGCAT | 3.27 |
| CAGCGCG | 3.27 |
| GTACGAC | 3.27 |
| AGTGGGA | 3.27 |
| TCCGTCA | 3.27 |
| GACCTAG | 3.27 |
| GTCGCGG | 3.27 |
| TAACGTA | 3.27 |
| TAAGGCA | 3.27 |
| GCTGGCA | 3.27 |
| GCTCACG | 3.27 |
| TCGGTTG | 3.27 |
| CGTCGGT | 3.27 |
| TCGCCGA | 3.27 |
| AGTGGCT | 3.27 |
| CGTGTCA | 3.27 |
| GAGGATA | 3.27 |
| CACCTTG | 3.27 |
| TTTCTCC | 3.27 |
| TGTCCGT | 3.26 |
| GCTGTGG | 3.26 |
| TACGTCG | 3.26 |
| ATCCCGT | 3.26 |
| CAAGGTG | 3.26 |
| TGCCTCT | 3.26 |
| TGTGGCA | 3.26 |
| GGCGCAT | 3.26 |
| AACGCTC | 3.26 |
| GAACGTG | 3.26 |
| CTCGTGC | 3.26 |
| GCTGCAC | 3.25 |
| TCCGGGT | 3.25 |
| CCCGCTA | 3.25 |
| CATGAGC | 3.25 |
| TGCCTTA | 3.25 |
| CCGCACC | 3.25 |
| AACGTCA | 3.25 |
| CAAGCAG | 3.25 |
| AATCCCT | 3.25 |
| GTGCACT | 3.24 |
| GGCCGAA | 3.24 |
| CTTCCAC | 3.24 |
| GCCGTAT | 3.24 |
| TCGCATA | 3.24 |
| TCCGCCA | 3.24 |
| GTAGACC | 3.24 |
| GCAGCAG | 3.24 |
| GTTGCTC | 3.24 |
| GATGGTA | 3.24 |
| TAGCACC | 3.24 |
| AACGTTG | 3.24 |
| GTCCGGA | 3.24 |
| GTGGAAG | 3.24 |
| TCCCACT | 3.24 |
| TGTCGCC | 3.24 |
| CAGCGTA | 3.23 |
| TCGCGCC | 3.23 |
| ACCGGTC | 3.23 |
| CTACGGC | 3.23 |
| TACGCAA | 3.23 |
| TGCGTGC | 3.23 |
| AAAGGAC | 3.23 |
| TGCCACA | 3.23 |
| GGTGCTA | 3.23 |
| ATGGCGA | 3.23 |
| TTCCATC | 3.23 |
| CACGTGT | 3.23 |
| GGCGACC | 3.23 |
| GCGCGTT | 3.23 |
| ATTGCCG | 3.23 |
| AGACGAG | 3.23 |
| GGTGTCT | 3.23 |
| ACGCATG | 3.23 |
| GCCGGTG | 3.22 |
| AGCCACA | 3.22 |
| TGCCGCC | 3.22 |
| ACACGTA | 3.22 |
| ACACGGC | 3.22 |
| CCCCGAT | 3.22 |
| TACCTGG | 3.22 |
| TGGCGAA | 3.22 |
| TCGGTAA | 3.22 |
| CAGCGCC | 3.22 |
| CAACCGC | 3.22 |
| CTGCTGC | 3.22 |
| TCCGTGC | 3.21 |
| TCCGGAC | 3.21 |
| GTACGCT | 3.21 |
| CACCGTG | 3.21 |
| GTGCGTA | 3.21 |
| CGCCGTG | 3.21 |
| TTGGACT | 3.21 |
| CCCGAAG | 3.21 |
| GACCTAC | 3.21 |
| TAGCGGG | 3.21 |
| GCTCATG | 3.21 |
| AGGCGCT | 3.21 |
| GGCGGTG | 3.21 |
| AACGTGC | 3.21 |
| GACGTCT | 3.21 |
| ATCCATC | 3.20 |
| AAAGGCT | 3.20 |
| GCCGAGG | 3.20 |
| CAACCGA | 3.20 |
| TACGCTT | 3.20 |
| CTCGCCA | 3.20 |
| ATCGTCA | 3.20 |
| GACCGTA | 3.20 |
| ACCGCAT | 3.20 |
| TACGTAG | 3.20 |
| TTTCCCG | 3.20 |
| GCAGTAG | 3.20 |
| AGACGAA | 3.20 |
| CTCCAAC | 3.20 |
| GTACTTC | 3.19 |
| CGTGGCG | 3.19 |
| CTGGATC | 3.19 |
| TATCCTG | 3.19 |
| TACGGGA | 3.19 |
| CTACATC | 3.19 |
| GATGGAT | 3.19 |
| AGGGATT | 3.19 |
| CTCCGAG | 3.19 |
| GTTGGAG | 3.19 |
| AATCATA | 3.19 |
| ATACGCA | 3.19 |
| GGCGTTC | 3.19 |
| GGCGTTG | 3.19 |
| TAGCTCT | 3.18 |
| CTCGTGG | 3.18 |
| GAACGCC | 3.18 |
| CACGAGG | 3.18 |
| GATGGAA | 3.18 |
| TGTGGCT | 3.18 |
| GCTGGTC | 3.18 |
| GCTGGCT | 3.18 |
| GCCGTTC | 3.18 |
| CCAGGTA | 3.18 |
| AGACGAT | 3.18 |
| GACCTTG | 3.17 |
| ATACCGA | 3.17 |
| CGACGGA | 3.17 |
| CGTGTCG | 3.17 |
| AAGGAAT | 3.17 |
| GTACGAT | 3.17 |
| TGCCGGT | 3.17 |
| GTAGGTG | 3.17 |
| CAAGGTC | 3.17 |
| GGCCGAT | 3.17 |
| CACGGTC | 3.17 |
| ATTCCCA | 3.17 |
| AAGCGTA | 3.17 |
| GTAGGCG | 3.17 |
| CGGCAAT | 3.17 |
| AGCCAGC | 3.17 |
| CTCCGGT | 3.16 |
| GGTCGTT | 3.16 |
| GTGCGAT | 3.16 |
| TGAGTGG | 3.16 |
| GTGCTGA | 3.16 |
| GCACTCG | 3.16 |
| TACGCAC | 3.16 |
| TGCGTAT | 3.16 |
| GAAGGCG | 3.16 |
| GCACGCA | 3.16 |
| ACGGACA | 3.15 |
| AGCCAGT | 3.15 |
| GATCCAT | 3.15 |
| CGAGCAG | 3.15 |
| CAGGATA | 3.15 |
| GGTGGAT | 3.15 |
| GATGTAG | 3.15 |
| GCGGGAT | 3.15 |
| GCGCGAA | 3.15 |
| ACTGGCT | 3.15 |
| GACCACT | 3.15 |
| AGCCGGT | 3.14 |
| GGTGAGG | 3.14 |
| CTACGTC | 3.14 |
| CACGCAG | 3.14 |
| TCAGGCT | 3.14 |
| CTACTGC | 3.14 |
| TCCGCCT | 3.14 |
| GAGCGTG | 3.14 |
| GCTCCGT | 3.14 |
| TGACGGA | 3.14 |
| CACGTCA | 3.14 |
| CACGGCG | 3.14 |
| TATGATT | 3.14 |
| CCACTCA | 3.14 |
| CCGGAGA | 3.14 |
| GTGCGTC | 3.14 |
| AACCGGG | 3.14 |
| GCGGGAA | 3.14 |
| GGTGTGT | 3.13 |
| GATCCAC | 3.13 |
| AGCCTGA | 3.13 |
| GGCGTGT | 3.13 |
| TTACGGG | 3.13 |
| CGTGAGC | 3.13 |
| ATCGCGG | 3.13 |
| CGAGTCG | 3.13 |
| AGTGGTC | 3.13 |
| CGCCTAG | 3.12 |
| AGTGTTG | 3.12 |
| GATCCAG | 3.12 |
| AGAGCTA | 3.12 |
| TCAGCAC | 3.12 |
| CACCGAG | 3.12 |
| CGCGTCA | 3.12 |
| TCGGTAT | 3.12 |
| GGTCACA | 3.12 |
| GCGGACT | 3.12 |
| TTGCAGG | 3.11 |
| CAACGCC | 3.11 |
| TTTGCCG | 3.11 |
| TACGTGT | 3.11 |
| AGACACC | 3.11 |
| AGACTAG | 3.11 |
| AAACCAT | 3.11 |
| TCGCGTG | 3.11 |
| GTGGATC | 3.11 |
| CACGGTG | 3.11 |
| TACGCCA | 3.11 |
| TACGGTC | 3.11 |
| AGGCGCA | 3.11 |
| CCTCACC | 3.10 |
| ATGCTAT | 3.10 |
| ATACCGC | 3.10 |
| GCGCTAC | 3.10 |
| CCTGTTC | 3.10 |
| AAGCGTC | 3.10 |
| GTGCTCT | 3.10 |
| TATGCGA | 3.10 |
| GACCAGT | 3.10 |
| TGGGAAT | 3.10 |
| ATCGTCT | 3.10 |
| CGCCTAC | 3.09 |
| CATGAGG | 3.09 |
| AAGCACT | 3.09 |
| CGCCACG | 3.09 |
| ATGGTTT | 3.09 |
| TACGCTG | 3.09 |
| CTAGTCT | 3.09 |
| GTCGTGG | 3.09 |
| TGTGACC | 3.09 |
| GCGCGTA | 3.09 |
| ATCCACC | 3.09 |
| GAAGTAC | 3.09 |
| ACTGCTC | 3.09 |
| TAACACG | 3.09 |
| GACCGGC | 3.09 |
| CTCCGCT | 3.09 |
| AGTGCTT | 3.09 |
| CAGCAAC | 3.09 |
| GAACGGT | 3.09 |
| ACACACC | 3.08 |
| TCCCGTA | 3.08 |
| CGCGCCT | 3.08 |
| ATGGATC | 3.08 |
| AACCATC | 3.08 |
| AAACGTA | 3.08 |
| GAGCAGT | 3.08 |
| CAGCAGC | 3.08 |
| TTGCGGC | 3.08 |
| AGAGCAC | 3.08 |
| CCAGAGC | 3.07 |
| CATCCGG | 3.07 |
| CCAGCAA | 3.07 |
| GCGGTAA | 3.07 |
| AACGTGG | 3.07 |
| CAACACT | 3.07 |
| CTCGGCA | 3.07 |
| CCTCCGC | 3.07 |
| GGTCGTA | 3.07 |
| CACCTCG | 3.07 |
| ACCGGAC | 3.07 |
| TGACGAG | 3.07 |
| CTACTAG | 3.07 |
| CTTCCAT | 3.07 |
| CACCTAC | 3.07 |
| ACCCGGA | 3.07 |
| CGACACG | 3.06 |
| CCAGTGA | 3.06 |
| ACTGGTC | 3.06 |
| TTGCTGG | 3.06 |
| CACCTAG | 3.06 |
| TGCCAGT | 3.06 |
| TATCTCC | 3.06 |
| GCGGACA | 3.06 |
| AGCGCCT | 3.06 |
| GACCGAG | 3.06 |
| TTGCGTG | 3.06 |
| GACGCGT | 3.06 |
| GCACGAG | 3.06 |
| TGACGCC | 3.06 |
| ATAGGAC | 3.06 |
| TTACCGC | 3.06 |
| ACCGGTT | 3.05 |
| GCTCGCG | 3.05 |
| GTTGCTG | 3.05 |
| TTCGGGG | 3.05 |
| TAACGTG | 3.05 |
| CACGTAT | 3.05 |
| CTCGGTC | 3.05 |
| CTTGTCT | 3.05 |
| GATGGCG | 3.05 |
| ACCCTAA | 3.05 |
| CGCCATC | 3.05 |
| CACGTAA | 3.05 |
| TCTCGCT | 3.05 |
| ACGGTTT | 3.05 |
| CTGCGGC | 3.05 |
| TAGCTTT | 3.05 |
| TCCGTAG | 3.04 |
| TCACTGG | 3.04 |
| TTGCTTG | 3.04 |
| GACGGGA | 3.04 |
| GCTCCGC | 3.04 |
| ATAGCAT | 3.04 |
| TGCGCAC | 3.04 |
| CTACACC | 3.04 |
| CTAGGTG | 3.04 |
| CGTCCGT | 3.04 |
| GACGCAC | 3.04 |
| ATCGCCT | 3.04 |
| CTAGTAG | 3.04 |
| GTCCTAT | 3.03 |
| CCTCATG | 3.03 |
| TACGTTA | 3.03 |
| GCGGTAT | 3.03 |
| ACACACG | 3.03 |
| TACCAAG | 3.03 |
| TCTGGGT | 3.03 |
| CGGGAAT | 3.03 |
| TCGGGAT | 3.03 |
| TTACCGA | 3.03 |
| ATAGGGA | 3.03 |
| CAAGCAA | 3.03 |
| CGAGGCG | 3.03 |
| CTACACA | 3.03 |
| TATCCTA | 3.03 |
| AGACAAG | 3.03 |
| GCTGCTG | 3.03 |
| TGGCGAG | 3.03 |
| GTCGTCT | 3.02 |
| GCACTGA | 3.02 |
| ATTGCTC | 3.02 |
| AAGCGAT | 3.02 |
| GAAGGTC | 3.02 |
| ATGCTAA | 3.02 |
| ATGGAAG | 3.02 |
| CGACGTA | 3.02 |
| CACCGGT | 3.02 |
| AGTGGAA | 3.02 |
| ATGGAGA | 3.02 |
| AGCCTAT | 3.02 |
| GTTGGGT | 3.02 |
| ACCCGTC | 3.02 |
| TCACGCC | 3.01 |
| GATGGTT | 3.01 |
| TGACACA | 3.01 |
| CATGCGT | 3.01 |
| CCTGCAA | 3.01 |
| CTCGTCT | 3.01 |
| AGCCGTG | 3.01 |
| AGACAGA | 3.01 |
| ACCCAAC | 3.00 |
| CGCCAGC | 3.00 |
| CTTGGTA | 3.00 |
| AAAGCTA | 3.00 |
| ACGGCGA | 3.00 |
| GGTGCGG | 3.00 |
| ATCGGGG | 3.00 |
| CTAGGCG | 3.00 |
| TTAGGGT | 3.00 |
| GGCGAGG | 3.00 |
| AGGCGAA | 3.00 |
| GTAGCGG | 3.00 |
| TGCGTCT | 3.00 |
| GCACAGC | 3.00 |
| ACGCTCG | 3.00 |
| TTCCACC | 3.00 |
| GAGCGTT | 3.00 |
| CCGCACG | 3.00 |
| CAGCAGT | 3.00 |
| TGACGAT | 3.00 |
| TGGCGGA | 2.99 |
| ATCCGGC | 2.99 |
| CACGGCA | 2.99 |
| TTGGGTT | 2.99 |
| GCTCTGG | 2.99 |
| GCCGCTC | 2.99 |
| TGTGTCA | 2.99 |
| CGGCAAA | 2.99 |
| AACGCAC | 2.99 |
| GCCGGTC | 2.99 |
| GCGCTTG | 2.99 |
| CCTGCGT | 2.99 |
| CGTGTGT | 2.99 |
| ACTGGCA | 2.99 |
| CTGCATC | 2.99 |
| CTTGCAG | 2.99 |
| CGACACA | 2.99 |
| TACGCTC | 2.99 |
| TCAGTGC | 2.99 |
| GGAGATA | 2.99 |
| GTGCGGT | 2.99 |
| AGTCCGC | 2.99 |
| TCTCCAT | 2.98 |
| CTACGAG | 2.98 |
| GCTCGCC | 2.98 |
| GACCGCT | 2.98 |
| ATTCCTC | 2.98 |
| CTTGGCG | 2.98 |
| TCGGAGG | 2.97 |
| ATACGTC | 2.97 |
| GAGCAAT | 2.97 |
| GACCTTC | 2.97 |
| ACACGCC | 2.97 |
| TCTGCTC | 2.97 |
| CGACGTC | 2.97 |
| CACCTTC | 2.97 |
| GACCTTA | 2.97 |
| CACCGGC | 2.97 |
| GGCGTAG | 2.97 |
| TCTGTGC | 2.97 |
| CGAGGTG | 2.97 |
| CCCGACT | 2.97 |
| CTCGTCA | 2.96 |
| AACCCAA | 2.96 |
| TGCGGGA | 2.96 |
| TCGCGTC | 2.96 |
| GCTGGTG | 2.96 |
| TTCCACT | 2.96 |
| TAGCACT | 2.96 |
| CTTGACC | 2.96 |
| GTGCAGT | 2.96 |
| ATAGGCT | 2.96 |
| AGTGGCG | 2.96 |
| ACCCAGA | 2.96 |
| GTCCGTT | 2.96 |
| ACCGCGA | 2.96 |
| AACCGGC | 2.96 |
| CGCCAAG | 2.96 |
| CCACGCG | 2.96 |
| CGTGTTA | 2.96 |
| CACCGAT | 2.95 |
| GGCGCTG | 2.95 |
| ACCCGTT | 2.95 |
| CTCGGAC | 2.95 |
| GAGCAGA | 2.95 |
| TACCACC | 2.95 |
| GCTCGGC | 2.95 |
| CACGCTC | 2.95 |
| ACGGGAA | 2.95 |
| AACGCGC | 2.94 |
| TTAGCAT | 2.94 |
| TTCCCGT | 2.94 |
| GACGGGT | 2.94 |
| ATCCTGG | 2.94 |
| TCTGTCT | 2.94 |
| ACACGGA | 2.94 |
| CGTGCGC | 2.94 |
| GCGCACA | 2.94 |
| CCACACT | 2.94 |
| GCTGTGC | 2.94 |
| ACGCGTG | 2.94 |
| CCCGGTT | 2.94 |
| CTAGTCG | 2.94 |
| TTACGTG | 2.93 |
| GCCGTGT | 2.93 |
| CGCGGCA | 2.93 |
| CACCGGA | 2.93 |
| ATGCACT | 2.93 |
| GCGGAGG | 2.93 |
| TCAGGCA | 2.93 |
| GGTGGAA | 2.93 |
| GATCTCC | 2.93 |
| AATCCCG | 2.93 |
| GGTCGAC | 2.93 |
| GCCGTAG | 2.92 |
| ACTGTGG | 2.92 |
| TCCGGCT | 2.92 |
| ATACGCC | 2.92 |
| CCACAAC | 2.92 |
| GAACGAT | 2.92 |
| TCCCAAC | 2.92 |
| GTCCGGT | 2.92 |
| TTTCCCA | 2.92 |
| TGGGAAA | 2.92 |
| CACCAGC | 2.91 |
| GCACAGA | 2.91 |
| ACGCTTG | 2.91 |
| CCGCTAC | 2.91 |
| TACGTAT | 2.91 |
| GCCGCAT | 2.91 |
| GATGCAG | 2.91 |
| TTACGGC | 2.91 |
| GTTGGGA | 2.91 |
| TGCGTAG | 2.91 |
| CGCCTTC | 2.91 |
| TGCGCCA | 2.91 |
| CCACTTG | 2.91 |
| CAACGCG | 2.91 |
| GGTCAAG | 2.91 |
| TCTCGGG | 2.91 |
| TGCCTTT | 2.91 |
| TTCGGGC | 2.91 |
| TGTGTAG | 2.90 |
| ACGCAGG | 2.90 |
| GGTGTAG | 2.90 |
| GCTGCGG | 2.90 |
| TGCCTGA | 2.90 |
| ACTGCTG | 2.90 |
| TTGGATG | 2.90 |
| GCTGGCG | 2.90 |
| GACGCTC | 2.89 |
| CTACGTA | 2.89 |
| CTGCAAG | 2.89 |
| GAACGTC | 2.89 |
| GGTCGGC | 2.89 |
| AGTGTCT | 2.89 |
| CCACAGT | 2.89 |
| CATCCAA | 2.89 |
| CTGCTAC | 2.89 |
| CACGTTC | 2.89 |
| TCGGTTA | 2.89 |
| GCCGCAA | 2.89 |
| GCTCAGG | 2.89 |
| TAAGGTG | 2.88 |
| ATACGTA | 2.88 |
| TTAGGCT | 2.88 |
| GTCCGAG | 2.88 |
| GACCTGT | 2.88 |
| TTCCCGA | 2.88 |
| CGTGGAA | 2.88 |
| AAACCGG | 2.88 |
| TGCGGCA | 2.88 |
| CCGCAGC | 2.88 |
| CAGGAAT | 2.88 |
| CGCCTCT | 2.88 |
| TGACGCA | 2.88 |
| AGTGTGG | 2.88 |
| GTCGCCT | 2.87 |
| ATAGCAC | 2.87 |
| ATCCCGA | 2.87 |
| TGCGTCG | 2.87 |
| ATCCGGT | 2.87 |
| ACTGCAC | 2.87 |
| AGCCTAA | 2.87 |
| TGACGGC | 2.87 |
| TTAGCAC | 2.87 |
| AAGCAAC | 2.87 |
| GGTCAGC | 2.87 |
| AGACACT | 2.87 |
| GATCGAA | 2.87 |
| TTCCGGG | 2.87 |
| ACCGGCT | 2.87 |
| TAACAAG | 2.87 |
| AGAGCAT | 2.87 |
| CAAGCGT | 2.87 |
| CACCGTA | 2.87 |
| CCAGGAT | 2.87 |
| ACCCGCA | 2.87 |
| AGACTGC | 2.87 |
| GCCCGAA | 2.87 |
| CAACGTC | 2.86 |
| ATGCTCT | 2.86 |
| TAGCGCT | 2.86 |
| GAACTCC | 2.86 |
| TAGCGGC | 2.86 |
| ACGCGGA | 2.86 |
| CTACTTG | 2.86 |
| TAAGGTC | 2.86 |
| TCCCTAT | 2.86 |
| ATTCCTG | 2.86 |
| ACTCCAA | 2.86 |
| TGTGTCG | 2.86 |
| TGTCAGG | 2.86 |
| GTTCGGG | 2.85 |
| CCGCTTC | 2.85 |
| TCTGGGA | 2.85 |
| GTCCAGA | 2.85 |
| CCTGAGC | 2.85 |
| GTGCTAT | 2.85 |
| AGCGGTC | 2.85 |
| AAGCGTG | 2.85 |
| ATACACA | 2.85 |
| TAACGCC | 2.85 |
| GGAGTTC | 2.85 |
| CCTCCGA | 2.85 |
| TTCCACG | 2.84 |
| AGACGCG | 2.84 |
| TCCCAGA | 2.84 |
| TGCGTTC | 2.84 |
| ACAGGTC | 2.84 |
| CACGGCT | 2.84 |
| GGTCGCA | 2.84 |
| TCTCCAA | 2.84 |
| GTTGCTT | 2.84 |
| TACGTTT | 2.84 |
| CTACGGA | 2.84 |
| CTGCTAG | 2.84 |
| AGTGCAT | 2.84 |
| CACCTTA | 2.84 |
| GTGCTAA | 2.84 |
| ACCGTGA | 2.84 |
| TTCGTCT | 2.84 |
| GGAGATC | 2.83 |
| GAGGAAT | 2.83 |
| TAGCGTA | 2.83 |
| CAAGTAG | 2.83 |
| CTTCGGG | 2.83 |
| TGAGTGC | 2.83 |
| TTGGAGT | 2.83 |
| GCAGTCT | 2.83 |
| CAGGAAA | 2.83 |
| TAGGATA | 2.83 |
| CCGGTTT | 2.83 |
| TCTGGAC | 2.83 |
| GTTGTGG | 2.83 |
| AAAGGCA | 2.83 |
| CAAGTGG | 2.83 |
| GTTGGAC | 2.82 |
| GACCAGA | 2.82 |
| GCTGACC | 2.82 |
| CGCCTCG | 2.82 |
| TGTCGTG | 2.82 |
| GCCGAGC | 2.82 |
| ATTGCTG | 2.82 |
| CCTGTGA | 2.82 |
| CACCTCA | 2.82 |
| TAACCGA | 2.82 |
| GGCGTTA | 2.82 |
| AACGTAG | 2.82 |
| CAGCAAT | 2.82 |
| TGTCCGC | 2.82 |
| TCACAGG | 2.82 |
| AGGCGAT | 2.82 |
| CTAGCAG | 2.82 |
| ATCGGCC | 2.82 |
| AGTGCTA | 2.82 |
| GGAGTTA | 2.82 |
| GACCGTT | 2.81 |
| GAAGGTG | 2.81 |
| TGAGGTG | 2.81 |
| CAGCAGA | 2.81 |
| GCACGAT | 2.81 |
| ACCGTTG | 2.81 |
| TAACTCC | 2.81 |
| GCCGTTA | 2.81 |
| TATGACG | 2.81 |
| AGAGGCG | 2.81 |
| CCACGAC | 2.81 |
| TGTGCGC | 2.80 |
| CCAGACA | 2.80 |
| GGCGACA | 2.80 |
| GTAGCAG | 2.80 |
| CCTCTCT | 2.80 |
| TGTCTGG | 2.80 |
| AGTGTGC | 2.80 |
| TCCGTGT | 2.80 |
| GCGGGTT | 2.80 |
| TTCGCCT | 2.80 |
| CCTGACA | 2.80 |
| TACGGTG | 2.80 |
| GCACACT | 2.80 |
| CCACTGA | 2.79 |
| GACGCTG | 2.79 |
| CCAGACG | 2.79 |
| CCTCAGC | 2.79 |
| TTGCGGT | 2.79 |
| GCACTCA | 2.79 |
| GGCGCGA | 2.79 |
| AATCCTT | 2.78 |
| CGCGAGC | 2.78 |
| CACGCGT | 2.78 |
| CGCGCTT | 2.78 |
| CACGGTA | 2.78 |
| ACCGTAT | 2.78 |
| CACGCTT | 2.78 |
| GTTGTTC | 2.78 |
| TGACGTC | 2.78 |
| GAACAAC | 2.78 |
| TCAGTGG | 2.78 |
| CATCGAT | 2.78 |
| CTGCGGT | 2.77 |
| TTGCACC | 2.77 |
| AGCGCTA | 2.77 |
| TCGGACT | 2.77 |
| GGTCTTC | 2.77 |
| TATCGCG | 2.77 |
| TACCGTA | 2.77 |
| TTGGAGA | 2.77 |
| ACACGTC | 2.77 |
| TCTGGTC | 2.76 |
| GAAGCGG | 2.76 |
| TTTCCTG | 2.76 |
| CACGCGA | 2.76 |
| ACCGTTT | 2.76 |
| CGTCTGG | 2.76 |
| TCACGTC | 2.76 |
| AGTCCGT | 2.76 |
| CAACGAA | 2.76 |
| TCCGTCG | 2.76 |
| CTACTCA | 2.76 |
| CAAGCGC | 2.76 |
| TCGCTGG | 2.75 |
| CTGCACA | 2.75 |
| TCTGCTG | 2.75 |
| GCACGAC | 2.75 |
| GATGTAT | 2.75 |
| ATGCGGC | 2.75 |
| ACCGCAC | 2.75 |
| TGAGTCT | 2.75 |
| TCGCGCA | 2.75 |
| CTCCGGA | 2.75 |
| ACAGTCT | 2.75 |
| CTACAAG | 2.75 |
| AACGGAC | 2.75 |
| TTCCGTT | 2.75 |
| GACCGGT | 2.75 |
| GCCGCAG | 2.75 |
| CAGCGGT | 2.75 |
| ATCGGGC | 2.75 |
| CTTGTTA | 2.75 |
| GTGCGGA | 2.75 |
| CCACGCA | 2.74 |
| ATCGTAC | 2.74 |
| GACGTTG | 2.74 |
| GAGCGAG | 2.74 |
| CAGCGTC | 2.74 |
| TTCCTTT | 2.74 |
| GGTCGAG | 2.74 |
| GGTGTCG | 2.74 |
| ATGCGTT | 2.74 |
| AAGCAGC | 2.74 |
| CACCAGT | 2.74 |
| GACCTCT | 2.74 |
| AAACGGA | 2.74 |
| GGTCTTG | 2.73 |
| CGTGCGT | 2.73 |
| CCTCTTC | 2.73 |
| TAACGGT | 2.73 |
| CTGCTGT | 2.73 |
| AACGCTG | 2.73 |
| GTCCTAA | 2.73 |
| ACGCGCT | 2.73 |
| TCCGGTC | 2.73 |
| AACGACC | 2.73 |
| TAACACA | 2.73 |
| AGCGTGC | 2.73 |
| GCTGAGG | 2.73 |
| TACGGTA | 2.73 |
| TTCCAGG | 2.72 |
| AGACTCA | 2.72 |
| GTAGTAG | 2.72 |
| TGACACC | 2.72 |
| ACAGCAG | 2.72 |
| CTCGTAG | 2.72 |
| TTACGGT | 2.72 |
| AGACTAC | 2.72 |
| CCTCATA | 2.72 |
| GAAGACC | 2.72 |
| GACGCTT | 2.72 |
| GCTGCTT | 2.72 |
| GGTCGGT | 2.72 |
| ATCGCTT | 2.72 |
| CTACTAC | 2.72 |
| ATCGCGC | 2.72 |
| CTCGACC | 2.72 |
| CGTCATA | 2.71 |
| TGCGCCT | 2.71 |
| ATCGCAC | 2.71 |
| AGACTGA | 2.71 |
| GACCAAC | 2.71 |
| TGCGTGG | 2.71 |
| CTCGTCG | 2.71 |
| AATCCTC | 2.71 |
| GTAGTCT | 2.71 |
| TGCGGGT | 2.71 |
| GTCCAAC | 2.71 |
| ACGCGGT | 2.71 |
| TGTGCAG | 2.71 |
| ACACTAG | 2.71 |
| AAGCGCC | 2.71 |
| TAAGGCG | 2.70 |
| TCCCGTT | 2.70 |
| ATTGCTT | 2.70 |
| GAGCGTC | 2.70 |
| GAACGCA | 2.70 |
| CAACGAC | 2.70 |
| AGAGAGG | 2.70 |
| CTCGGAG | 2.70 |
| TTGCACG | 2.70 |
| CACCAAC | 2.70 |
| GAAGAGG | 2.70 |
| TCACGGT | 2.70 |
| GGTGTGA | 2.70 |
| TTAGGAC | 2.70 |
| CACGGAT | 2.70 |
| GTTGGTC | 2.69 |
| AAGCGCG | 2.69 |
| TCAGTCT | 2.69 |
| TGAGTAG | 2.69 |
| GGTGGTA | 2.69 |
| CCTGTAT | 2.69 |
| CCTCGAG | 2.69 |
| TCCGCTG | 2.69 |
| AAACCGT | 2.69 |
| TCACACC | 2.69 |
| CTCGGTG | 2.69 |
| GGCGTGA | 2.69 |
| AAAGGAA | 2.69 |
| GCACTTG | 2.69 |
| TCCGGCA | 2.69 |
| CTACGCG | 2.69 |
| CGACAAG | 2.69 |
| AGCCGAG | 2.69 |
| CTGCTTC | 2.69 |
| ACCGCTG | 2.69 |
| GTCCGCA | 2.69 |
| TGCGTCA | 2.68 |
| ACTGGTG | 2.68 |
| AACCCGA | 2.68 |
| CCACAGA | 2.68 |
| ACGCTAG | 2.68 |
| CAAGAGG | 2.68 |
| ATACACG | 2.68 |
| TTCGCCA | 2.68 |
| TGAGGCG | 2.68 |
| GCCGCTA | 2.68 |
| TCTGTGG | 2.68 |
| GTTCCGG | 2.68 |
| CCTGGAA | 2.68 |
| ATACATC | 2.68 |
| GTGCAGA | 2.67 |
| GAAGCAG | 2.67 |
| TTAGGGA | 2.67 |
| GACCGAC | 2.67 |
| GATCCGG | 2.67 |
| ATACGGT | 2.67 |
| TCGCGAT | 2.67 |
| CAAGTGC | 2.67 |
| GATGCAA | 2.67 |
| AGCGTTC | 2.67 |
| GATGTGT | 2.67 |
| TCCCGCA | 2.67 |
| GGTGTCA | 2.67 |
| ATCCAGG | 2.67 |
| TCTCGGC | 2.66 |
| ACACAAG | 2.66 |
| AAGCGTT | 2.66 |
| GTCCGAC | 2.66 |
| ACGGACT | 2.66 |
| CGCGAAG | 2.66 |
| GCCGGTA | 2.66 |
| CTTGTGT | 2.66 |
| CAGCGTT | 2.66 |
| GGTCTGT | 2.66 |
| TATCATA | 2.66 |
| GTTGGTG | 2.66 |
| CGAGAGG | 2.66 |
| ACAGACC | 2.66 |
| GACGACG | 2.66 |
| CTCGTGT | 2.66 |
| GACGTTC | 2.66 |
| GTCGGAC | 2.65 |
| TGTGTTA | 2.65 |
| AAGGATT | 2.65 |
| TACCGTG | 2.65 |
| ATACAGG | 2.65 |
| TGACGAC | 2.65 |
| AAGCGAG | 2.65 |
| ATGCAGC | 2.65 |
| TAGCAAC | 2.65 |
| TATGACT | 2.65 |
| CGGCGAT | 2.65 |
| CAAGACC | 2.65 |
| CTAGCGG | 2.65 |
| ATCGGCT | 2.65 |
| CCTCGAC | 2.65 |
| CGCCTCA | 2.65 |
| TGTCCGA | 2.65 |
| TACGTGA | 2.65 |
| TAGCGCC | 2.65 |
| GCCGGTT | 2.65 |
| AGACTGT | 2.65 |
| CTCCTGA | 2.64 |
| TGCCGTT | 2.64 |
| CACCTCT | 2.64 |
| AAGCAAT | 2.64 |
| TTCCGAG | 2.64 |
| CGCGTCT | 2.64 |
| CTAGCGT | 2.64 |
| TCTGCAC | 2.64 |
| GCAGAGC | 2.64 |
| CTTGTAG | 2.64 |
| TCAGGCG | 2.64 |
| AACCCGC | 2.64 |
| GGCGGTC | 2.64 |
| AGCCAAC | 2.64 |
| GCAGTCG | 2.64 |
| GACGTCA | 2.64 |
| CTAGTGT | 2.64 |
| AACGGAA | 2.64 |
| AACGGTC | 2.64 |
| AAGCAGT | 2.64 |
| GAGGATT | 2.64 |
| CTCGTTA | 2.64 |
| ACCGCGT | 2.64 |
| TACGGAC | 2.64 |
| TCTGGCA | 2.64 |
| GTGCAAC | 2.64 |
| GACGACC | 2.64 |
| TATCCAT | 2.64 |
| CCGCTAG | 2.63 |
| GGTGCAA | 2.63 |
| GTTGCAC | 2.63 |
| TATGAGG | 2.63 |
| CTGCGAG | 2.63 |
| CTACGTT | 2.63 |
| GTCGCAC | 2.63 |
| AGCCAGA | 2.63 |
| AATGACC | 2.63 |
| GGTCGCT | 2.63 |
| GTCGTAC | 2.63 |
| ACGCGAA | 2.63 |
| TACGCTA | 2.63 |
| TCTGGCT | 2.63 |
| AGCCGAC | 2.63 |
| TTGCATC | 2.63 |
| CAAGTCG | 2.63 |
| CTTGTCA | 2.63 |
| AGCGCCA | 2.63 |
| AGCCGTT | 2.63 |
| CCGGATG | 2.63 |
| AGAGGTG | 2.63 |
| GCTCTGC | 2.63 |
| GCCGTGA | 2.63 |
| CTCGAGG | 2.63 |
| TGCCAGA | 2.63 |
| CCTCTCG | 2.62 |
| TCGCGCT | 2.62 |
| GTAGTTC | 2.62 |
| GATCTAC | 2.62 |
| TGAGTCG | 2.62 |
| GGTCATT | 2.62 |
| GTAGCGC | 2.62 |
| ACGCACC | 2.62 |
| ACCGCAG | 2.62 |
| TTGCGCC | 2.62 |
| GCGCAGC | 2.62 |
| TGCGACC | 2.62 |
| AAACGGT | 2.62 |
| TTCGCGC | 2.62 |
| CCTGGAT | 2.62 |
| TCAGGAG | 2.61 |
| CGTGGTA | 2.61 |
| CGTGCAA | 2.61 |
| TCCGGAG | 2.61 |
| AGTCATA | 2.61 |
| AACGCAT | 2.61 |
| ATGCAAC | 2.61 |
| TAACGAA | 2.61 |
| GCGCTAG | 2.61 |
| GGCGTAT | 2.61 |
| CCTGGTT | 2.61 |
| GCTGCAT | 2.61 |
| AACGGCA | 2.61 |
| ACCCGCT | 2.61 |
| CTCCAAT | 2.61 |
| AAACGTT | 2.61 |
| TTACGTA | 2.61 |
| CACGACA | 2.61 |
| TCCGGTG | 2.61 |
| GGACTTT | 2.61 |
| GCCGTAA | 2.61 |
| GGTCACT | 2.61 |
| AGAGGTC | 2.61 |
| TGACAAG | 2.60 |
| GTTGGCT | 2.60 |
| CGACTAG | 2.60 |
| AGCGGAG | 2.60 |
| GAACTAC | 2.60 |
| ATCCGTG | 2.60 |
| TGCCGTC | 2.60 |
| CGCGTAT | 2.60 |
| TCGCTCG | 2.60 |
| TGTGGCG | 2.60 |
| AGCGCAC | 2.60 |
| CGCCTTA | 2.60 |
| GGTGCGT | 2.60 |
| AGCGTCT | 2.60 |
| AACGCTT | 2.60 |
| TAACGTT | 2.60 |
| TCCCGAA | 2.60 |
| CGCCTGA | 2.60 |
| ACCGACC | 2.60 |
| GACGTGA | 2.60 |
| CTGCGTC | 2.60 |
| TCACGTA | 2.60 |
| TCCGCTC | 2.60 |
| GTGCGAG | 2.60 |
| TCCCTAA | 2.60 |
| GACGTAT | 2.59 |
| ACCCGAA | 2.59 |
| CCTCTTG | 2.59 |
| CCAGACT | 2.59 |
| ATCGTGC | 2.59 |
| ACACATC | 2.59 |
| TCACGGC | 2.59 |
| AGAGTGG | 2.59 |
| GCACTAT | 2.59 |
| CACGGAA | 2.59 |
| ACCCAAT | 2.59 |
| ATAGTGG | 2.59 |
| ACTGCTT | 2.59 |
| ACGCGAG | 2.59 |
| CGTGTAT | 2.59 |
| CTAGACA | 2.59 |
| AACGTTT | 2.59 |
| AGCGTGG | 2.59 |
| ACTGTCT | 2.59 |
| TAACCGC | 2.59 |
| CCGGAAC | 2.59 |
| TTCGTGC | 2.59 |
| TACCGCG | 2.59 |
| TGCGTAA | 2.58 |
| GATGACC | 2.58 |
| TGCGCTC | 2.58 |
| TGTCGAG | 2.58 |
| GCAGCGT | 2.58 |
| CTAGCGC | 2.58 |
| TGTGTAT | 2.58 |
| AGCGTGA | 2.58 |
| CTAGTCA | 2.58 |
| AGTCCGA | 2.58 |
| CGACACC | 2.58 |
| TCCCGCT | 2.58 |
| CAGCGAG | 2.58 |
| TTGCGAA | 2.58 |
| CTGCTCA | 2.57 |
| TATGATA | 2.57 |
| CTTCCGG | 2.57 |
| CCCGAGA | 2.57 |
| AAGCAGA | 2.57 |
| AAACCAA | 2.57 |
| TGCGGTG | 2.57 |
| GCGGTTA | 2.57 |
| GACGGCA | 2.57 |
| TGCCGGA | 2.57 |
| TGACGCT | 2.57 |
| AAAGTCC | 2.57 |
| AACCCGT | 2.57 |
| ATTGGAG | 2.57 |
| GAACACT | 2.57 |
| AGTCTGG | 2.57 |
| ATGCTTT | 2.57 |
| GTTGCAT | 2.56 |
| CAACGAT | 2.56 |
| TGACTAG | 2.56 |
| ATTCGGC | 2.56 |
| TGCCGCA | 2.56 |
| AACGTTA | 2.56 |
| ACCGGCA | 2.56 |
| ACCGTTC | 2.56 |
| GGTCTCT | 2.56 |
| CGGCGTA | 2.56 |
| CCGCTTG | 2.56 |
| GCTGCGC | 2.55 |
| GAGCAAA | 2.55 |
| CTCGCTT | 2.55 |
| GACGTGT | 2.55 |
| TCGGGTT | 2.55 |
| CTCCGAT | 2.55 |
| CTGCTTA | 2.55 |
| CCAGCGA | 2.55 |
| GGCGCTA | 2.55 |
| CCTGACT | 2.55 |
| CCTCTAC | 2.55 |
| CACCGTT | 2.55 |
| CACGACG | 2.55 |
| GGTCATC | 2.55 |
| ATGGATA | 2.55 |
| ACACTGC | 2.55 |
| GTAGAGG | 2.55 |
| TGCCAAC | 2.55 |
| AACGTAT | 2.55 |
| CCTCGTC | 2.55 |
| CTCCTAT | 2.55 |
| CGACTCG | 2.55 |
| CAAGTCT | 2.55 |
| ATCGGCG | 2.55 |
| TCTGCTT | 2.54 |
| TGTCTAG | 2.54 |
| ATAGGAG | 2.54 |
| CACGTTA | 2.54 |
| GCCCGAT | 2.54 |
| AGTGACC | 2.54 |
| AGCGACC | 2.54 |
| TCGGGAA | 2.54 |
| CGTCGAC | 2.54 |
| AAACCAG | 2.54 |
| CTGCAGC | 2.54 |
| ATCCGCT | 2.54 |
| TACCACG | 2.54 |
| GTCCGTA | 2.54 |
| CAGCGGA | 2.54 |
| AGACAGT | 2.54 |
| CGCGACA | 2.54 |
| TTGGTTT | 2.54 |
| AGACTTG | 2.54 |
| ATAGTGC | 2.54 |
| TACGGCA | 2.54 |
| CTCGCAG | 2.54 |
| AGCGAGA | 2.54 |
| CTCGTAT | 2.54 |
| CAGCGAT | 2.54 |
| GCCGGAT | 2.54 |
| AGTGTTC | 2.54 |
| GCGCGGT | 2.54 |
| AGCGTCA | 2.54 |
| ATACGCG | 2.53 |
| GTTGGCA | 2.53 |
| GCTCTAG | 2.53 |
| TCTCGAA | 2.53 |
| TACCGGC | 2.53 |
| GGAGATT | 2.53 |
| TGCCTAA | 2.53 |
| ATCCGCG | 2.53 |
| CCACTCT | 2.53 |
| ATCCGTC | 2.53 |
| TCTCGAG | 2.53 |
| AACGCTA | 2.52 |
| AAAGCAT | 2.52 |
| AACCAGG | 2.52 |
| GTTGTGC | 2.52 |
| TGCGTTG | 2.52 |
| TACGGCT | 2.52 |
| CACGCAA | 2.52 |
| ACGCGAT | 2.52 |
| ATCGCAA | 2.52 |
| GTTGCTA | 2.52 |
| TCGGAGC | 2.52 |
| CCACGCT | 2.52 |
| GTAGATC | 2.52 |
| GGCGTCA | 2.52 |
| TTAGGCA | 2.52 |
| ACGCATT | 2.52 |
| GTCGACC | 2.52 |
| TTACGCC | 2.52 |
| CGCCTAT | 2.52 |
| ATCGGTG | 2.51 |
| TGCCGAA | 2.51 |
| ATTCGAT | 2.51 |
| ACGGGAT | 2.51 |
| CTCGACG | 2.51 |
| AGTCAGG | 2.51 |
| TAGGAAA | 2.51 |
| ATTGGGT | 2.51 |
| CACCAGA | 2.51 |
| GCTGCAG | 2.51 |
| CTTGTCG | 2.51 |
| ACGCTTA | 2.51 |
| TCTGACC | 2.51 |
| CTCGTGA | 2.50 |
| ACACGAT | 2.50 |
| TGCGCGT | 2.50 |
| TGAGCAG | 2.50 |
| TCCGGAT | 2.50 |
| TTGCGCG | 2.50 |
| GCTGCTA | 2.50 |
| TAACGAG | 2.50 |
| TCGCGAC | 2.50 |
| ATTCCTA | 2.50 |
| CGAGCGT | 2.50 |
| CTGCAGT | 2.50 |
| AGTGCGC | 2.50 |
| ATCGTGG | 2.50 |
| GCAGTGT | 2.50 |
| GCACGAA | 2.49 |
| TTTGCTC | 2.49 |
| CGCGGTA | 2.49 |
| TAGCAGC | 2.49 |
| GGCGCTT | 2.49 |
| CATCGGG | 2.49 |
| TGACTCG | 2.49 |
| CTGCGGA | 2.49 |
| ACGCTGC | 2.49 |
| CGAGCAA | 2.49 |
| GTAGTCG | 2.49 |
| CGCCAGT | 2.49 |
| CTAGAGC | 2.49 |
| ACCGGAT | 2.49 |
| ACACGAC | 2.49 |
| TAGCAGA | 2.49 |
| GAACTGG | 2.49 |
| TACGAGG | 2.49 |
| ACTGGCG | 2.49 |
| CTGGTTT | 2.49 |
| GCGCAAG | 2.49 |
| TATCCAG | 2.49 |
| TGCGTGT | 2.49 |
| TAGCAAT | 2.48 |
| ATAGGCG | 2.48 |
| ATCCTTC | 2.48 |
| GGCGATG | 2.48 |
| CGACTGC | 2.48 |
| CGTCGTC | 2.48 |
| ATCCTTG | 2.48 |
| GAAGGAT | 2.48 |
| GCAGCAA | 2.48 |
| AAGCGGC | 2.48 |
| GTAGTGT | 2.48 |
| TACCGGA | 2.48 |
| TGCGGAC | 2.47 |
| GCGCTGT | 2.47 |
| CTCGCGT | 2.47 |
| GCTCATA | 2.47 |
| CGCCGAA | 2.47 |
| CGCGAAA | 2.47 |
| CTAGACG | 2.47 |
| CCACTAT | 2.47 |
| CGAGCGA | 2.47 |
| CAACGCA | 2.47 |
| GCACTCT | 2.47 |
| TTACGGA | 2.47 |
| TTAGTCT | 2.47 |
| GAACGAC | 2.47 |
| AACCTGG | 2.47 |
| TCACGCG | 2.47 |
| CCAGGAA | 2.47 |
| CGTCTAG | 2.47 |
| CCCGATG | 2.47 |
| CCCGTTT | 2.47 |
| CCGGAAG | 2.47 |
| CACCGTC | 2.47 |
| AACGGCT | 2.47 |
| TTGCTCG | 2.47 |
| AGCCGAT | 2.47 |
| TTTCGAG | 2.47 |
| AACCGCC | 2.47 |
| TCACGCT | 2.46 |
| ACACGCG | 2.46 |
| TACGTAA | 2.46 |
| AGTCTAG | 2.46 |
| TAGCAGT | 2.46 |
| GAACAAT | 2.46 |
| AAGCGCT | 2.46 |
| GTGCGCT | 2.46 |
| ACTGCAG | 2.46 |
| AGAGTCG | 2.46 |
| TCGCAGG | 2.46 |
| ACACGAG | 2.46 |
| TAGGAAT | 2.46 |
| TCGCATG | 2.46 |
| AGACGCT | 2.46 |
| GATCCAA | 2.46 |
| TCTGGTG | 2.46 |
| GACCGGA | 2.46 |
| TAAGCAG | 2.46 |
| GAAGCGC | 2.46 |
| TTGGATC | 2.46 |
| GTGCTTT | 2.45 |
| GGTCAGA | 2.45 |
| TAAGACC | 2.45 |
| ACGGAGT | 2.45 |
| TAACGTC | 2.45 |
| TTTCGCA | 2.45 |
| TGGCGCT | 2.45 |
| GATCGCG | 2.45 |
| GCCGAGA | 2.45 |
| TCACGAG | 2.45 |
| CTGCGCC | 2.45 |
| GAAGCAA | 2.45 |
| CGTCGGC | 2.45 |
| TAGCGAG | 2.45 |
| ATGCGGA | 2.45 |
| TATGAGC | 2.45 |
| CTACAGC | 2.44 |
| GACCTCA | 2.44 |
| AACGTAA | 2.44 |
| AGAGTCT | 2.44 |
| ATTGCTA | 2.44 |
| TATCCAC | 2.44 |
| TGTCACC | 2.44 |
| CTCGCTA | 2.44 |
| TTGGAAC | 2.44 |
| AGACTCT | 2.44 |
| TTTCCTA | 2.44 |
| TCTGCTA | 2.44 |
| GTCGAGG | 2.43 |
| TACGACC | 2.43 |
| CTCCGCA | 2.43 |
| TTCCTGG | 2.43 |
| CTTCCAA | 2.43 |
| AGCGGGA | 2.43 |
| GCTCCGA | 2.43 |
| AGCCAAT | 2.43 |
| TAACGGC | 2.43 |
| TTGCTGC | 2.43 |
| ATACAAG | 2.43 |
| AATCTCC | 2.43 |
| CGACGAA | 2.43 |
| CAACACC | 2.43 |
| ACTGCAT | 2.43 |
| CTACGAC | 2.43 |
| GGTGTTG | 2.43 |
| AATCCTA | 2.43 |
| ACGGGTT | 2.43 |
| CTTGCAA | 2.43 |
| TATCCGG | 2.43 |
| TCCGCAC | 2.42 |
| CTAGACT | 2.42 |
| ACACGCA | 2.42 |
| TACCGCT | 2.42 |
| GCTCGTG | 2.42 |
| TCAGGTG | 2.42 |
| GCAGTCA | 2.42 |
| ACTCCGT | 2.42 |
| ACACTAC | 2.42 |
| CAAGGAT | 2.42 |
| ACACGTT | 2.42 |
| TTGCTAG | 2.42 |
| GTCGTCA | 2.42 |
| TGAGACC | 2.42 |
| TCAGCAG | 2.42 |
| TCGCGAG | 2.42 |
| AAACCAC | 2.42 |
| AGCGGAT | 2.42 |
| AGCCGCT | 2.42 |
| TCTCTTT | 2.42 |
| ATCGTGA | 2.42 |
| ACGCTAC | 2.42 |
| ACAGTCG | 2.42 |
| ATTGGCT | 2.42 |
| TTGCTAC | 2.41 |
| TCTCGCC | 2.41 |
| ACGCGCA | 2.41 |
| CACCTAT | 2.41 |
| GGCGCAG | 2.41 |
| ATGCAGT | 2.41 |
| GCACGCT | 2.41 |
| ACACTGT | 2.41 |
| ATGCAGA | 2.41 |
| CGTCAGG | 2.41 |
| TTCGTAG | 2.41 |
| ATACTGG | 2.41 |
| GCCGAAT | 2.41 |
| GGAGTTT | 2.41 |
| CTAGCAA | 2.41 |
| GCACAAC | 2.41 |
| TGACTGC | 2.41 |
| CAAGCGG | 2.41 |
| AATCGAC | 2.41 |
| AATGCGC | 2.41 |
| AATCCTG | 2.41 |
| CCTCTGT | 2.41 |
| ACAGAGG | 2.41 |
| ACCGAGG | 2.41 |
| TGACAGA | 2.41 |
| CTCGGCT | 2.41 |
| CTCGCTG | 2.41 |
| AGAGTGC | 2.40 |
| GGCGGAA | 2.40 |
| TCGGACA | 2.40 |
| TACCACA | 2.40 |
| TCCGTGA | 2.40 |
| GTCGTGC | 2.40 |
| GTAGCAA | 2.40 |
| CTGGATA | 2.40 |
| TGACGAA | 2.40 |
| TTGCTTC | 2.40 |
| GTTGTTG | 2.40 |
| TTCCGCC | 2.40 |
| GTCGTTG | 2.40 |
| CCTGACG | 2.40 |
| GTGGTTT | 2.40 |
| CACCTGA | 2.40 |
| GGCGAGC | 2.40 |
| GGTCTTA | 2.40 |
| GCGCTTC | 2.40 |
| TACCAGC | 2.40 |
| TCGCGGA | 2.40 |
| GGTCTCA | 2.40 |
| GACGGAA | 2.40 |
| GTTCGGT | 2.40 |
| TAACAGG | 2.40 |
| ATTGTTC | 2.40 |
| GTTCCAA | 2.40 |
| GTTCATA | 2.39 |
| TTCGCCG | 2.39 |
| ATGCGCG | 2.39 |
| CGTGAGG | 2.39 |
| CGTGCGA | 2.39 |
| TGTGGTA | 2.39 |
| TCACACG | 2.39 |
| ATCGCGA | 2.39 |
| GACCTCG | 2.39 |
| GTCGTAT | 2.39 |
| CAACTGG | 2.39 |
| TCTGTCA | 2.39 |
| TTAGTCG | 2.39 |
| ATGCGCA | 2.39 |
| GAGCGCA | 2.39 |
| GCACTAA | 2.39 |
| TGTCATG | 2.39 |
| GCGCATC | 2.39 |
| CGAGTCA | 2.39 |
| AAAGCAC | 2.39 |
| CTTCTTT | 2.38 |
| CCTCGGT | 2.38 |
| TCAGACC | 2.38 |
| CCAGTTC | 2.38 |
| AGAGGAA | 2.38 |
| ACTGGAT | 2.38 |
| CCGCAAG | 2.38 |
| AGACAGC | 2.38 |
| AGAGACC | 2.38 |
| GAAGTGG | 2.38 |
| CTGCTGA | 2.38 |
| CGCCACA | 2.38 |
| CGAGTGA | 2.38 |
| ACAGCGC | 2.38 |
| GTCGGCT | 2.38 |
| AGTCGGC | 2.38 |
| TTCCGGT | 2.38 |
| CGCGTTA | 2.37 |
| GGCGTAA | 2.37 |
| ACCGCAA | 2.37 |
| CCAGGTT | 2.37 |
| AAACTCC | 2.37 |
| CAACAAC | 2.37 |
| TCTCCGT | 2.37 |
| CACGAGC | 2.37 |
| TGAGTCA | 2.37 |
| CCGGATC | 2.37 |
| TTGGAAG | 2.37 |
| GCCGACT | 2.37 |
| AACGCAG | 2.37 |
| AAGCGGT | 2.37 |
| CCACGAT | 2.37 |
| ACTGCGC | 2.37 |
| TTCCTCT | 2.37 |
| ATCCAGT | 2.37 |
| ACTGTAG | 2.37 |
| GGCGAGA | 2.37 |
| CGAGGTC | 2.37 |
| ACCGCTT | 2.36 |
| CTGCGTT | 2.36 |
| TCACGAT | 2.36 |
| CCTGCGA | 2.36 |
| TGTCATA | 2.36 |
| CATCGCC | 2.36 |
| GACGAGG | 2.36 |
| ATTCCAT | 2.36 |
| TCTGCAT | 2.36 |
| CCAGAGT | 2.36 |
| TGTCGGC | 2.36 |
| ATCCACG | 2.36 |
| GTCGCTG | 2.36 |
| AGTCGGA | 2.36 |
| CTCGGAT | 2.36 |
| GGTCGGA | 2.36 |
| TGAGCGC | 2.36 |
| ATCCGAG | 2.36 |
| CATGACA | 2.36 |
| GACCTGA | 2.36 |
| AGTGTGT | 2.36 |
| TTGCGAC | 2.36 |
| ACAGTCA | 2.36 |
| TCTCCGC | 2.36 |
| TTCGCGG | 2.36 |
| GCCGACA | 2.36 |
| TCCGTAT | 2.36 |
| ATACACC | 2.35 |
| GGTGACA | 2.35 |
| CCAGTAT | 2.35 |
| GTCCGAT | 2.35 |
| GGTCTGA | 2.35 |
| TGTGCGG | 2.35 |
| TCCGAGG | 2.35 |
| AGCGGTT | 2.35 |
| AGCCGTA | 2.35 |
| TACCAGT | 2.35 |
| GATCTTC | 2.35 |
| ATCGAAT | 2.35 |
| TTACGAA | 2.35 |
| AACGGGT | 2.35 |
| CTTGCGC | 2.35 |
| TCAGGTC | 2.35 |
| TCTCGAC | 2.35 |
| CTTGTAT | 2.35 |
| TATGACA | 2.35 |
| TACGAAC | 2.35 |
| CAGGATT | 2.35 |
| CCGCGAA | 2.35 |
| ATAGGTG | 2.35 |
| GCTGTAG | 2.35 |
| ACTGCTA | 2.34 |
| CTTGCGT | 2.34 |
| ATCGTTG | 2.34 |
| CTCGAGA | 2.34 |
| ACAGTGT | 2.34 |
| TAGGATT | 2.34 |
| TATGAAT | 2.34 |
| ACCCAAA | 2.34 |
| ACCGTAA | 2.34 |
| CCCGAAC | 2.34 |
| GGTCAGT | 2.34 |
| ATCCACT | 2.34 |
| CCACTTC | 2.34 |
| GTCCAAT | 2.34 |
| GCTGTCT | 2.34 |
| CGCCAAC | 2.34 |
| CTCGCAC | 2.33 |
| CTACACT | 2.33 |
| TCTGGCG | 2.33 |
| TTTGCTG | 2.33 |
| CGCCGTT | 2.33 |
| GAACGAA | 2.33 |
| GCTGGTA | 2.33 |
| TTTGGGT | 2.33 |
| CTTGCGG | 2.33 |
| TTCGTAT | 2.33 |
| GCAGCGA | 2.33 |
| CTCGGTA | 2.33 |
| GGCGGTT | 2.33 |
| GCGCTCA | 2.33 |
| GAACGAG | 2.33 |
| TTGCAAG | 2.33 |
| ATTCTCC | 2.33 |
| AGCGGGT | 2.33 |
| CGCGGAA | 2.33 |
| GGCGGTA | 2.33 |
| TCGCGGT | 2.33 |
| CGTCGTG | 2.33 |
| CGGGATT | 2.33 |
| ACCGCGC | 2.33 |
| ATTCCAG | 2.33 |
| ACTCTGG | 2.33 |
| GGAGAAT | 2.33 |
| CCCGAAT | 2.33 |
| GCAGGTA | 2.32 |
| CTCGACA | 2.32 |
| CTACTGT | 2.32 |
| TACCTTC | 2.32 |
| CCTCGTA | 2.32 |
| CAAGGAA | 2.32 |
| TCACTAG | 2.32 |
| TAGCGAT | 2.32 |
| TTACGAG | 2.32 |
| TGAGGTC | 2.32 |
| GACGCTA | 2.32 |
| CCTGTTA | 2.32 |
| ACCGGTG | 2.32 |
| CGTGTGA | 2.32 |
| GATGCGG | 2.32 |
| CAAGGTA | 2.32 |
| TAGCGTC | 2.31 |
| ACGGAGA | 2.31 |
| GGTGTAT | 2.31 |
| CCGCATC | 2.31 |
| GAACGCT | 2.31 |
| GACGGTC | 2.31 |
| CCTCACG | 2.31 |
| ATACGGA | 2.31 |
| CTACAGT | 2.31 |
| TTACTGC | 2.31 |
| CAGCAAA | 2.31 |
| AGACTAA | 2.31 |
| ACACACT | 2.31 |
| AGTGTAG | 2.31 |
| GTCGTAG | 2.31 |
| CGTCGAT | 2.31 |
| ACTGACC | 2.31 |
| ATGCGAG | 2.31 |
| AACGTGA | 2.31 |
| ATGCAAT | 2.31 |
| CGCGTAG | 2.31 |
| TTCCGTG | 2.31 |
| TCCGCTT | 2.31 |
| AAGCGGA | 2.31 |
| GTAGTCA | 2.30 |
| CTAGTGA | 2.30 |
| CGCCGTA | 2.30 |
| GCAGTAA | 2.30 |
| TTCCGTC | 2.30 |
| TGACTAC | 2.30 |
| CAGCGAC | 2.30 |
| CGCCTTT | 2.30 |
| GACGGTG | 2.30 |
| TAACGAT | 2.30 |
| TACGCAT | 2.30 |
| AAAGGCG | 2.30 |
| TTCCTTG | 2.30 |
| ATTGGAC | 2.30 |
| ATTCATA | 2.30 |
| AAACGGC | 2.30 |
| ATTGGTC | 2.30 |
| TACCTAG | 2.30 |
| CTGCGTA | 2.30 |
| ACAGTAG | 2.30 |
| GTAGCGT | 2.30 |
| TGCCGAC | 2.29 |
| TTGCTTA | 2.29 |
| AGCGTAG | 2.29 |
| TTCGTGT | 2.29 |
| TAACGCT | 2.29 |
| TTCGGCA | 2.29 |
| TACCTTG | 2.29 |
| ATACGAA | 2.29 |
| TATGAAG | 2.29 |
| TTGCAGT | 2.29 |
| TATGAAC | 2.29 |
| AACGGTG | 2.29 |
| GCGCACT | 2.29 |
| ACGCTGA | 2.29 |
| GCTCGGT | 2.29 |
| ATGCGTA | 2.29 |
| CCAGTAA | 2.29 |
| CTCGCTC | 2.29 |
| CGTCTCG | 2.29 |
| GAGCGAA | 2.28 |
| ACTGGTA | 2.28 |
| TCCGTTA | 2.28 |
| AGCGGCA | 2.28 |
| CCAGTTG | 2.28 |
| TCTCGTT | 2.28 |
| TTAGTGC | 2.28 |
| TTACTGG | 2.28 |
| GTCGCAT | 2.28 |
| TACCACT | 2.28 |
| TACGGCG | 2.28 |
| TGCGGAG | 2.28 |
| GACCAAT | 2.28 |
| GAAGGTA | 2.28 |
| TTCCACA | 2.28 |
| TCCGCAT | 2.28 |
| GACGTTA | 2.28 |
| TGTGGAA | 2.28 |
| ATCCTTT | 2.28 |
| ACCGGAA | 2.28 |
| TGTGGAT | 2.28 |
| CTTCATA | 2.28 |
| CTCGAAA | 2.28 |
| AGTGCAA | 2.27 |
| CGCGGAT | 2.27 |
| AACGTGT | 2.27 |
| TGTGAGG | 2.27 |
| ATAGGTC | 2.27 |
| AGTGGTA | 2.27 |
| GCGCTGA | 2.27 |
| GCGCAGT | 2.27 |
| ACAGCGT | 2.27 |
| TCCGACC | 2.27 |
| TTTCGCT | 2.27 |
| ATACGAG | 2.27 |
| TTCCGCG | 2.27 |
| GTAGGTA | 2.27 |
| TACGCAG | 2.27 |
| ATCGCGT | 2.27 |
| ATACGTT | 2.27 |
| GACCGCA | 2.27 |
| AAACACA | 2.27 |
| GACCTAT | 2.27 |
| GCCGGAA | 2.27 |
| AATGCGT | 2.27 |
| TGACTGT | 2.26 |
| ACACTCG | 2.26 |
| ATCCACA | 2.26 |
| GGCGCAA | 2.26 |
| TACCTGC | 2.26 |
| TACCTAC | 2.26 |
| TCACGGA | 2.26 |
| AGTGCGG | 2.26 |
| TTTCGCC | 2.26 |
| ATCGATG | 2.26 |
| ACTCATA | 2.26 |
| CTGGAAT | 2.26 |
| TTCGGGT | 2.26 |
| GGCGAAA | 2.26 |
| CCACTAA | 2.26 |
| TTGCGCA | 2.26 |
| CGACTTG | 2.26 |
| CCTCACA | 2.26 |
| ACACAGT | 2.26 |
| GAGCGAC | 2.26 |
| ACCCGAT | 2.26 |
| TCCGACT | 2.25 |
| GCTCACC | 2.25 |
| GTACTTT | 2.25 |
| TTCGCAA | 2.25 |
| CCTCAAG | 2.25 |
| AAGCAAA | 2.25 |
| TTCGGGA | 2.25 |
| GACGCAG | 2.25 |
| GAAGATC | 2.25 |
| TTCCAGT | 2.25 |
| CGTGGAT | 2.25 |
| AGTCGCG | 2.25 |
| GCTCACA | 2.24 |
| TTTGCTT | 2.24 |
| ACGCACA | 2.24 |
| TCAGCGC | 2.24 |
| TAACGGA | 2.24 |
| ACTGTGT | 2.24 |
| TAAGTCG | 2.24 |
| GAGCGGA | 2.24 |
| GATCGGG | 2.24 |
| TCCGAGC | 2.24 |
| TGAGCGT | 2.24 |
| TACCGCC | 2.24 |
| TATGAGT | 2.24 |
| CAACGCT | 2.24 |
| ATCGTAG | 2.24 |
| ATCCGTT | 2.23 |
| GAACACC | 2.23 |
| TCAGTCG | 2.23 |
| GCTCGAC | 2.23 |
| GTGCGAC | 2.23 |
| ATGGAAT | 2.23 |
| TTCCGGC | 2.23 |
| CCGCACT | 2.23 |
| GACCGTC | 2.23 |
| TTGCACT | 2.23 |
| GTTGGCG | 2.23 |
| GCGGAGT | 2.23 |
| TTTCGTT | 2.23 |
| TAAGCGT | 2.23 |
| CTTGAGG | 2.23 |
| CAACAAT | 2.23 |
| TCACACA | 2.23 |
| TGTGTGA | 2.23 |
| TCTCGAT | 2.23 |
| AAAGAGA | 2.23 |
| TGACACT | 2.23 |
| CTCGGAA | 2.23 |
| TTACGTC | 2.23 |
| CTAGAGT | 2.23 |
| ACGCAAG | 2.22 |
| AAACGTC | 2.22 |
| TGTGTTT | 2.22 |
| TTCCGCT | 2.22 |
| TTGCTGT | 2.22 |
| GCGGAGA | 2.22 |
| CAACAGT | 2.22 |
| AATGCGG | 2.22 |
| ATCCTGC | 2.22 |
| TTGCACA | 2.22 |
| CAGCGCA | 2.22 |
| CGTCGTT | 2.22 |
| CATGCGA | 2.22 |
| ATTGTTG | 2.22 |
| TTACGTT | 2.22 |
| CTGCGAC | 2.22 |
| AGTGTCG | 2.22 |
| TAAGTGG | 2.22 |
| CGTCGAG | 2.21 |
| ACACTGA | 2.21 |
| CGCGCAT | 2.21 |
| GAAGTCG | 2.21 |
| ACTGGAA | 2.21 |
| TCCCAAA | 2.21 |
| TACGCGT | 2.21 |
| CCGCTGT | 2.21 |
| AATCGCT | 2.21 |
| CTCGCAT | 2.21 |
| CTCGAGC | 2.21 |
| GTCGCTT | 2.21 |
| AACCGAG | 2.21 |
| ATCCTTA | 2.21 |
| GTCGTGT | 2.21 |
| CACGGTT | 2.21 |
| AGCCGAA | 2.21 |
| TAAGCAA | 2.21 |
| AGTGGAT | 2.21 |
| TTAGTGG | 2.21 |
| CTAGGTA | 2.20 |
| ATGCGAC | 2.20 |
| CGCCAGA | 2.20 |
| CAGCGCT | 2.20 |
| GCGCTCT | 2.20 |
| AAAGGAT | 2.20 |
| CACCGAC | 2.20 |
| GTCGCAA | 2.20 |
| AACCGCT | 2.20 |
| TGACTCA | 2.20 |
| CTACGAA | 2.20 |
| TGTGCGT | 2.20 |
| AACGAAC | 2.20 |
| TCGCGTT | 2.20 |
| GTCGACG | 2.20 |
| TGTGAGC | 2.20 |
| TCGGATG | 2.20 |
| ACAGCAA | 2.20 |
| CGAGTGT | 2.19 |
| TCACAAG | 2.19 |
| TCCGTTT | 2.19 |
| GTAGTGA | 2.19 |
| TCGCGTA | 2.19 |
| CCGCTCA | 2.19 |
| CCGCACA | 2.19 |
| TGAGAGG | 2.19 |
| CTGCTCT | 2.19 |
| CCGCATT | 2.19 |
| GATCGGT | 2.19 |
| GTGGATA | 2.19 |
| TTGCTCA | 2.19 |
| TGCGGTC | 2.19 |
| ATTGGGA | 2.19 |
| AGCCGTC | 2.19 |
| AGCGTAT | 2.19 |
| ATACGAC | 2.19 |
| TACCTTA | 2.19 |
| GTGCAAA | 2.19 |
| AAAGAAG | 2.19 |
| TACCGTC | 2.18 |
| CAACTGC | 2.18 |
| AGTCGGT | 2.18 |
| ACTGTCG | 2.18 |
| CCACGAA | 2.18 |
| AACGAAA | 2.18 |
| ACTCAGG | 2.18 |
| AGCGGTG | 2.18 |
| GATGTAA | 2.18 |
| GGTGAGC | 2.18 |
| TCACGAC | 2.18 |
| TCACTAC | 2.18 |
| ATAGTCG | 2.18 |
| GACCTTT | 2.18 |
| TTACGCA | 2.18 |
| ATCGGAC | 2.18 |
| TAACGAC | 2.18 |
| AGTGTCA | 2.18 |
| ACTGTTG | 2.18 |
| GAAGCGT | 2.18 |
| GGTGTTC | 2.18 |
| GATGTGA | 2.17 |
| AACCAAG | 2.17 |
| TAACGCG | 2.17 |
| ATCCTCT | 2.17 |
| ATTGCAT | 2.17 |
| GCGCATT | 2.17 |
| TTACATC | 2.17 |
| ATCCGCA | 2.17 |
| ATTCCAC | 2.17 |
| TTTGCAC | 2.17 |
| AGCCAAA | 2.17 |
| TAAGGAT | 2.17 |
| TTCGGAC | 2.17 |
| GTCGTTC | 2.17 |
| TAAGGTA | 2.17 |
| ACACAGA | 2.16 |
| AACGGGA | 2.16 |
| GTCGGAG | 2.16 |
| CTACGCT | 2.16 |
| AGTCATG | 2.16 |
| TGCGCTA | 2.16 |
| ATTGGCA | 2.16 |
| TGCGAAA | 2.16 |
| TCCGCGA | 2.16 |
| TGCGCTG | 2.16 |
| TCTGGTA | 2.16 |
| TAGCAAA | 2.16 |
| GCAGGAT | 2.16 |
| TGTCGGT | 2.16 |
| GAACAGT | 2.16 |
| TTTGGGA | 2.16 |
| GTCGATT | 2.16 |
| CCGCGAT | 2.16 |
| CTCCGAA | 2.16 |
| CGCGTAA | 2.16 |
| TTACACG | 2.16 |
| TCAGCGG | 2.16 |
| CACGACT | 2.16 |
| TAAGTGC | 2.16 |
| TACGGAT | 2.16 |
| GTGGAAT | 2.16 |
| ATCGTAT | 2.16 |
| TCCCAAT | 2.16 |
| TGTGCAA | 2.16 |
| TTCGTTA | 2.15 |
| CAAGTCA | 2.15 |
| CGTCGCT | 2.15 |
| CGCGTGA | 2.15 |
| CGTCATG | 2.15 |
| ATGCGAT | 2.15 |
| CTCCTAA | 2.15 |
| TTAGTAG | 2.15 |
| ACGCTTC | 2.15 |
| AAAGGTC | 2.15 |
| CTTGTGA | 2.15 |
| AGACAAC | 2.15 |
| TGAGCAA | 2.15 |
| AGCCGCA | 2.15 |
| CAACTAC | 2.15 |
| CGCGCAA | 2.15 |
| TGCCAAT | 2.15 |
| TTCGCTC | 2.15 |
| CTCGTAA | 2.15 |
| TGCGCAT | 2.14 |
| TTCGTGG | 2.14 |
| GAGCGAT | 2.14 |
| TACCGAG | 2.14 |
| CTAGCGA | 2.14 |
| TCCGGAA | 2.14 |
| AAAGTAC | 2.14 |
| TTCGTCA | 2.14 |
| TCAGTGT | 2.14 |
| CACCGCT | 2.14 |
| GCACTTA | 2.14 |
| ACTGCGG | 2.14 |
| AGAGGAT | 2.14 |
| TCTGCAG | 2.14 |
| CATGACT | 2.14 |
| CCTCGCT | 2.14 |
| TGTCTGC | 2.14 |
| GACGGAT | 2.14 |
| GTCGGTG | 2.14 |
| TCTCGTG | 2.14 |
| ACCGAGC | 2.14 |
| GTCGGAT | 2.14 |
| TTAGGAG | 2.14 |
| GATCGCA | 2.13 |
| CTTGGTT | 2.13 |
| AAACGGG | 2.13 |
| GCCGAGT | 2.13 |
| ATCCGGA | 2.13 |
| ACTCTAG | 2.13 |
| CTGCTAA | 2.13 |
| TTCGTTC | 2.13 |
| CACCAAT | 2.13 |
| GTGGAAA | 2.13 |
| ACTCGAG | 2.13 |
| ACCGATC | 2.13 |
| TCACGTT | 2.13 |
| CACGTTT | 2.13 |
| ACGCGTT | 2.13 |
| TACCTGT | 2.13 |
| CTTGTTT | 2.13 |
| GCTCGGA | 2.12 |
| GATGCGC | 2.12 |
| ATCCGTA | 2.12 |
| ATTGCAC | 2.12 |
| TACCAGA | 2.12 |
| CATCGTG | 2.12 |
| CTCGTTC | 2.12 |
| ACACAGC | 2.12 |
| AAACAAG | 2.12 |
| TAAGTCT | 2.12 |
| CAGCGAA | 2.12 |
| TGACAGT | 2.12 |
| TCTGTCG | 2.12 |
| GTTCGTT | 2.12 |
| AGACTAT | 2.12 |
| TTTGCTA | 2.12 |
| AGAGCAG | 2.12 |
| TCAGTCA | 2.12 |
| TTACAGG | 2.12 |
| GCAGACG | 2.11 |
| TAGCGTT | 2.11 |
| CATCGGC | 2.11 |
| CGTCTAC | 2.11 |
| ATTGGTG | 2.11 |
| TCCGCAG | 2.11 |
| TGAGTGT | 2.11 |
| AAACGCG | 2.11 |
| AACGGTA | 2.11 |
| ACGCTCA | 2.11 |
| CTGCTAT | 2.11 |
| AGTCGTG | 2.11 |
| GACCGAA | 2.11 |
| GAGCGCT | 2.11 |
| TTAGCAG | 2.11 |
| CTGCAAC | 2.11 |
| CGACTAC | 2.11 |
| GTCGGCA | 2.11 |
| GCAGACA | 2.11 |
| ACCGACG | 2.11 |
| GACGGCT | 2.11 |
| TGTCGTC | 2.11 |
| GAAGTGC | 2.11 |
| TCTCGCG | 2.10 |
| TGCCGCT | 2.10 |
| TGCCGTA | 2.10 |
| CCACTTA | 2.10 |
| GCAGTTG | 2.10 |
| CTGCAGA | 2.10 |
| CGTCTGC | 2.10 |
| CTAGGAT | 2.10 |
| TTCGTCG | 2.10 |
| CTACTGA | 2.10 |
| CTTCGCT | 2.10 |
| AGCGCGT | 2.10 |
| CCTGAGT | 2.10 |
| ACACTCA | 2.10 |
| GAAGAAA | 2.10 |
| TAGCGAC | 2.10 |
| GTGCGAA | 2.10 |
| TTTGGCT | 2.10 |
| GGTCAAC | 2.10 |
| GTTCCGC | 2.10 |
| GTCGAGA | 2.09 |
| ATACTAG | 2.09 |
| CCTCTCA | 2.09 |
| TTCGTTG | 2.09 |
| ATCCTAG | 2.09 |
| CTGCGCA | 2.09 |
| ACAGGTA | 2.09 |
| TCTCTGG | 2.09 |
| TCTGTGT | 2.09 |
| ATCGTTA | 2.09 |
| GTAGGAT | 2.09 |
| CGCGAAC | 2.09 |
| GTTGCAG | 2.09 |
| CTACTAA | 2.09 |
| CTTGGAT | 2.09 |
| ATAGTCT | 2.09 |
| ACCGAAA | 2.09 |
| TCACTGT | 2.09 |
| ACTCGCC | 2.09 |
| ACTGTTC | 2.09 |
| CGCGTGT | 2.09 |
| CGTCTCA | 2.09 |
| TCCGTAA | 2.09 |
| AGCGGCT | 2.08 |
| ACTCCGC | 2.08 |
| TACCGTT | 2.08 |
| ACAGTGA | 2.08 |
| TAGCGGA | 2.08 |
| GTTCTTC | 2.08 |
| ATACGAT | 2.08 |
| TCACATC | 2.08 |
| GCAGGAA | 2.08 |
| AACCGGA | 2.08 |
| GACCTAA | 2.08 |
| GACGACA | 2.08 |
| CAAGTGT | 2.08 |
| ACTGTCA | 2.08 |
| AAGCGCA | 2.08 |
| CTGCGCT | 2.08 |
| AGCGCTT | 2.08 |
| CTACAGA | 2.08 |
| ACTGCAA | 2.08 |
| GTCGCTC | 2.08 |
| CCTCGGA | 2.08 |
| GCCGCTT | 2.07 |
| AGCGTGT | 2.07 |
| TATCCAA | 2.07 |
| GAACTAG | 2.07 |
| GTGCAAT | 2.07 |
| GTCGAAT | 2.07 |
| ACAGGAT | 2.07 |
| ATAGCAG | 2.07 |
| TTTCTTC | 2.07 |
| TTGCTGA | 2.07 |
| AAAGGTG | 2.07 |
| ATGCGCT | 2.07 |
| AAACCGA | 2.07 |
| AACGCGT | 2.07 |
| AAACGTG | 2.07 |
| ACACGAA | 2.07 |
| GCAGTAT | 2.07 |
| CGACTGT | 2.07 |
| AACCGTA | 2.07 |
| GCTGTGT | 2.07 |
| TACGAAA | 2.07 |
| CACCGCG | 2.07 |
| TGCGCGA | 2.07 |
| CCTCTTT | 2.07 |
| TTTCCAC | 2.07 |
| CGTGTAA | 2.07 |
| GTTGACC | 2.07 |
| AGACTTA | 2.07 |
| TGTCGAT | 2.07 |
| ACGGATG | 2.06 |
| ATTGTGC | 2.06 |
| GAAGTCT | 2.06 |
| ACACGCT | 2.06 |
| GCTCGAG | 2.06 |
| AACGACG | 2.06 |
| TGACTGA | 2.06 |
| TTGCGGA | 2.06 |
| GCACAAT | 2.06 |
| ACGCTGT | 2.06 |
| AGCGCTC | 2.06 |
| TTTCGTA | 2.06 |
| AACCTTC | 2.06 |
| ATCCTGT | 2.06 |
| CAACAGA | 2.06 |
| TCAGAGG | 2.06 |
| CCGCAGT | 2.06 |
| CTCGACT | 2.05 |
| ATCGGAG | 2.05 |
| CCAGAGA | 2.05 |
| TCAGTAG | 2.05 |
| AAACGAG | 2.05 |
| ATTCGGG | 2.05 |
| GACGTTT | 2.05 |
| GACCGAT | 2.05 |
| TTCGAGA | 2.05 |
| GCCGAAG | 2.05 |
| GATGCGT | 2.05 |
| AAGCGAC | 2.05 |
| ATCCAGC | 2.05 |
| CATCCGT | 2.05 |
| GCAGTGA | 2.05 |
| TCCGCAA | 2.05 |
| TGTCGTT | 2.05 |
| GTTGTCT | 2.05 |
| GCTGGAT | 2.04 |
| CACCTTT | 2.04 |
| ACAGCGG | 2.04 |
| AAACACG | 2.04 |
| AGTCGAG | 2.04 |
| GCCGTTT | 2.04 |
| ATCCTAC | 2.04 |
| CCTGTAA | 2.04 |
| CTGCGAT | 2.04 |
| CGACAGA | 2.04 |
| ATCCAAG | 2.04 |
| ATTGTCT | 2.04 |
| CGAGGAA | 2.04 |
| CCGCTGA | 2.04 |
| GTCCAAA | 2.04 |
| GCACTTC | 2.04 |
| ACCGACA | 2.04 |
| CTAGTTC | 2.03 |
| AACCGCA | 2.03 |
| AGACAAT | 2.03 |
| CTCGCGA | 2.03 |
| CGACACT | 2.03 |
| CGTGTTT | 2.03 |
| TTCGACC | 2.03 |
| GTCGAAA | 2.03 |
| TGACAGC | 2.03 |
| CTTCGTG | 2.03 |
| TGACTTG | 2.03 |
| CACGATG | 2.03 |
| TTGGATA | 2.03 |
| ATGCGAA | 2.03 |
| AGCGAGG | 2.02 |
| TACCTCG | 2.02 |
| GTCGCAG | 2.02 |
| GACGAGC | 2.02 |
| GTAGTTG | 2.02 |
| GACCAAA | 2.02 |
| AGTCACC | 2.02 |
| CGTCACG | 2.02 |
| CTACAAC | 2.02 |
| ACGCATC | 2.02 |
| GCTGTCA | 2.02 |
| TCTGTAG | 2.02 |
| TCAGCAA | 2.02 |
| TGCCGAT | 2.02 |
| TTGCGTT | 2.02 |
| TTAGGTC | 2.02 |
| CACGAAG | 2.02 |
| TGCGGCT | 2.02 |
| ACACTTG | 2.02 |
| TGCGCAA | 2.02 |
| AGCGAAA | 2.01 |
| GTAGACA | 2.01 |
| ATACGCT | 2.01 |
| AACGCGA | 2.01 |
| TTCCGGA | 2.01 |
| CTGCGAA | 2.01 |
| GATCATT | 2.01 |
| TAAGAGG | 2.01 |
| TTCGGCG | 2.01 |
| CTACGAT | 2.01 |
| GGTGACT | 2.01 |
| GACGAAG | 2.01 |
| GACGTAA | 2.01 |
| TCGGTTT | 2.01 |
| TCGCACC | 2.01 |
| TACGCGA | 2.01 |
| TGTCACA | 2.01 |
| TTCCGCA | 2.01 |
| CGACTCA | 2.01 |
| TGTCGGA | 2.00 |
| TTTCCAG | 2.00 |
| CGAGGAT | 2.00 |
| CTGGAAA | 2.00 |
| AGACTTC | 2.00 |
| CTCGGTT | 2.00 |
| CCTCTGA | 2.00 |
| TTCCTGC | 2.00 |
| GGTCTAT | 2.00 |
| TTCGATG | 2.00 |
| TGTCTGT | 2.00 |
| GTCGGTC | 2.00 |
| TTACGAC | 2.00 |
| TCGCTGC | 1.99 |
| ACAGACA | 1.99 |
| AAACGCC | 1.99 |
| ATCGGTC | 1.99 |
| GTCGTTA | 1.99 |
| AAACGCA | 1.99 |
| TCTGTTG | 1.99 |
| ATGGAAA | 1.99 |
| ATCCTCG | 1.99 |
| CTCGCAA | 1.99 |
| TTTGGAC | 1.99 |
| TTACACA | 1.99 |
| ATAGTAG | 1.99 |
| TCGGAGA | 1.99 |
| TTCGGCT | 1.99 |
| GTTCGAG | 1.99 |
| TTACACC | 1.99 |
| CATGACG | 1.99 |
| TAACACT | 1.99 |
| GGTCTTT | 1.99 |
| CAACTAG | 1.99 |
| AAACGAA | 1.99 |
| TAACGCA | 1.99 |
| CTAGTTG | 1.98 |
| CTCCGAC | 1.98 |
| GAAGAAC | 1.98 |
| TAAGGAA | 1.98 |
| TAGCGCA | 1.98 |
| AAGCGAA | 1.98 |
| CGAGACC | 1.98 |
| TGTCACG | 1.98 |
| ATACTGC | 1.98 |
| TCAGCGT | 1.98 |
| TACGGTT | 1.98 |
| AGCGTTG | 1.98 |
| CCGCTCT | 1.98 |
| TCCGGTT | 1.98 |
| AGTGTTA | 1.98 |
| GCGGAAC | 1.98 |
| ATCGCAT | 1.98 |
| TGTCTAC | 1.98 |
| GCCGAAA | 1.98 |
| AGCGTAA | 1.98 |
| CATCTAC | 1.98 |
| TAAGCGG | 1.98 |
| TAAGCGC | 1.98 |
| ACGCACG | 1.98 |
| GTTGTAG | 1.98 |
| CCGCAGA | 1.98 |
| TGCGGAT | 1.97 |
| TGAGTGA | 1.97 |
| TAACAAC | 1.97 |
| CGAGACG | 1.97 |
| GTAGAAG | 1.97 |
| TCACTGC | 1.97 |
| CGCCTAA | 1.97 |
| TTACGCG | 1.97 |
| AGAGCGC | 1.97 |
| TTTGGAG | 1.97 |
| TCCGACA | 1.97 |
| GTTCGGC | 1.97 |
| GTCGCTA | 1.97 |
| CATCGCT | 1.97 |
| TACCAAC | 1.97 |
| AATGATG | 1.97 |
| ATCGACA | 1.97 |
| TTCGGTG | 1.97 |
| TCACGCA | 1.97 |
| GAAGGTT | 1.97 |
| TCTGCGC | 1.97 |
| CCGGATA | 1.96 |
| CATCATT | 1.96 |
| TTTCCAT | 1.96 |
| CTAGAAG | 1.96 |
| CCTCTTA | 1.96 |
| TGCGAGG | 1.96 |
| AAAGAGG | 1.96 |
| TCTCTTC | 1.96 |
| AACCACC | 1.96 |
| AACCGTG | 1.96 |
| AACGAAG | 1.96 |
| TAAGGTT | 1.96 |
| TCACTCA | 1.96 |
| AACGGAT | 1.96 |
| GTCGCGA | 1.96 |
| GAACGCG | 1.96 |
| CCTCATC | 1.96 |
| AACGCAA | 1.96 |
| TTCGCAT | 1.96 |
| TTGCTCT | 1.95 |
| CTCGAAC | 1.95 |
| TCTGCGG | 1.95 |
| CGACTCT | 1.95 |
| TGCCAAA | 1.95 |
| TCACGAA | 1.95 |
| CTCGTTT | 1.95 |
| TTCGTAA | 1.95 |
| CACGAGT | 1.95 |
| AGCGCAG | 1.95 |
| GGTGGTT | 1.95 |
| GCTCAAG | 1.95 |
| GTTGGTA | 1.95 |
| TGCGCAG | 1.95 |
| GCTGTTC | 1.95 |
| AACCACG | 1.94 |
| TTTCGGT | 1.94 |
| GCTCTCG | 1.94 |
| AGCGACG | 1.94 |
| GTAGAGC | 1.94 |
| ATACTAC | 1.94 |
| TCTCCGA | 1.94 |
| TTGCTTT | 1.94 |
| TACCTCA | 1.94 |
| AACCTTG | 1.94 |
| TTGCAGC | 1.94 |
| GCAGACT | 1.94 |
| CACCGAA | 1.94 |
| ATCGCTA | 1.94 |
| CGTCAAG | 1.94 |
| GCTCGTC | 1.94 |
| TTTCGGG | 1.94 |
| AGCGCAT | 1.93 |
| TGAGACG | 1.93 |
| GCTCGCA | 1.93 |
| TGTCTCA | 1.93 |
| AATGATC | 1.93 |
| CTCCAAA | 1.93 |
| TTTCGTG | 1.93 |
| CTAGTAA | 1.93 |
| TTTCGAT | 1.93 |
| GGTGACG | 1.93 |
| GCTCTAC | 1.93 |
| CTTGAGC | 1.93 |
| GATGAGG | 1.93 |
| GCCGAAC | 1.93 |
| CGCGGTG | 1.93 |
| GGTCGAT | 1.93 |
| TTGCGAG | 1.93 |
| GTCCGAA | 1.92 |
| AACCTTA | 1.92 |
| GTAGTAT | 1.92 |
| GTTGTTA | 1.92 |
| TGCGTTA | 1.92 |
| AGCGGTA | 1.92 |
| TGCGAGC | 1.92 |
| TGCGCTT | 1.92 |
| TGAGCGG | 1.92 |
| GGTGTAA | 1.92 |
| ACGGAAG | 1.92 |
| GAAGAGA | 1.92 |
| GGTCTCG | 1.92 |
| GATCGTG | 1.92 |
| TATCGGG | 1.92 |
| GCTGTCG | 1.92 |
| ACTCCGA | 1.92 |
| ATCGGGT | 1.92 |
| TCCGCTA | 1.92 |
| CATCCGA | 1.92 |
| TTACGAT | 1.92 |
| TTACTAG | 1.91 |
| CTACTAT | 1.91 |
| CACCTAA | 1.91 |
| GTAGATG | 1.91 |
| CCACAAT | 1.91 |
| AGTCGAA | 1.91 |
| CATCTTC | 1.91 |
| CACCAAA | 1.91 |
| TGTGACA | 1.91 |
| CGTCACC | 1.91 |
| AAACCGC | 1.91 |
| ATCGCTG | 1.91 |
| GTAGGAA | 1.91 |
| TACCGAT | 1.91 |
| TTAGTCA | 1.91 |
| ATTGTGG | 1.91 |
| TGTCGCG | 1.91 |
| CTTCGTC | 1.91 |
| TGCGATC | 1.91 |
| TGAGGAT | 1.91 |
| TTCCTCG | 1.91 |
| GCGCAGA | 1.91 |
| TCGCTTG | 1.90 |
| ATCCTCA | 1.90 |
| GGCGAGT | 1.90 |
| ACAGGAA | 1.90 |
| ATCCGAC | 1.90 |
| CGTGACG | 1.90 |
| TTTGGTC | 1.90 |
| CTTCTAC | 1.90 |
| AATGCGA | 1.90 |
| GTTCGTA | 1.90 |
| TTCGTTT | 1.90 |
| CATCGAA | 1.90 |
| AGTCGCC | 1.90 |
| CTTCGGC | 1.90 |
| CTTCTAG | 1.90 |
| TTGCTAT | 1.90 |
| ATCGTGT | 1.90 |
| TTAGGTG | 1.90 |
| CATCGTC | 1.90 |
| TACGGAA | 1.90 |
| AATCCAT | 1.90 |
| GTAGACG | 1.90 |
| CAAGTGA | 1.90 |
| TCGCACG | 1.90 |
| AGTCGTC | 1.89 |
| GAACAGC | 1.89 |
| GGTCTAA | 1.89 |
| CAACAGC | 1.89 |
| ATTCCGG | 1.89 |
| CGTGACA | 1.89 |
| CACGAGA | 1.89 |
| ACGGAAC | 1.89 |
| GCTCGAA | 1.89 |
| ATAGACC | 1.89 |
| AGTCTGC | 1.89 |
| AGAGTGT | 1.89 |
| CTAGTAT | 1.89 |
| CTAGAGA | 1.89 |
| AACCGTC | 1.89 |
| TGCGTGA | 1.89 |
| AGCGAAC | 1.89 |
| TGAGGTA | 1.89 |
| ATCGGGA | 1.89 |
| AACGAGG | 1.89 |
| ATAGCAA | 1.89 |
| TTACAAG | 1.89 |
| CGTCTTC | 1.88 |
| ATGCAAA | 1.88 |
| ACAGTTG | 1.88 |
| ATTGGTA | 1.88 |
| CGACAGT | 1.88 |
| TTTGGCA | 1.88 |
| CATCAGG | 1.87 |
| CGTGGTT | 1.87 |
| CTAGATG | 1.87 |
| TCGCGAA | 1.87 |
| CATCGCA | 1.87 |
| TAAGTAG | 1.87 |
| GCAGTTT | 1.87 |
| CGTCTTG | 1.87 |
| CGACTGA | 1.87 |
| CGACTTA | 1.87 |
| ACGGATC | 1.87 |
| CAAGGTT | 1.87 |
| CATCCGC | 1.87 |
| ATACACT | 1.87 |
| GTTCGTG | 1.87 |
| GCGCTTA | 1.87 |
| TGCGGTT | 1.87 |
| ATGGATT | 1.87 |
| CCTCATT | 1.87 |
| AAACTGC | 1.87 |
| ACTCGGT | 1.86 |
| CTTCGGA | 1.86 |
| AGAGTCA | 1.86 |
| TTTGGTG | 1.86 |
| TGAGACA | 1.86 |
| CATGATG | 1.86 |
| GATCATG | 1.86 |
| AGAGCAA | 1.86 |
| TCCGGTA | 1.86 |
| GCTGTTG | 1.86 |
| GGCGTTT | 1.86 |
| CTTCGGT | 1.86 |
| ATCGTTT | 1.86 |
| AATCCAG | 1.86 |
| AGCGCGA | 1.86 |
| AGCGAGC | 1.86 |
| CTTCGTT | 1.86 |
| TTTCGGC | 1.86 |
| TTAGCAA | 1.86 |
| GGCGACT | 1.86 |
| ACTCGGA | 1.86 |
| TCACTCG | 1.86 |
| CATCATG | 1.86 |
| TTGCTAA | 1.85 |
| TTAGACC | 1.85 |
| AGTGAGG | 1.85 |
| TGTCGAC | 1.85 |
| CATCTAG | 1.85 |
| AGTCTCG | 1.85 |
| GAAGATG | 1.85 |
| TTACTAC | 1.85 |
| GCTGCAA | 1.85 |
| CTAGGAA | 1.85 |
| ATACTCG | 1.85 |
| GCCGATG | 1.85 |
| ATCGTTC | 1.85 |
| TTTCGAC | 1.85 |
| CAACTGT | 1.85 |
| ACCGAGA | 1.85 |
| TTTGCAT | 1.85 |
| TGTCTCT | 1.85 |
| AACCACA | 1.85 |
| TGTCTTG | 1.85 |
| ATAGTCA | 1.85 |
| TTCGGAG | 1.85 |
| TTCCTAC | 1.84 |
| GCGGTTT | 1.84 |
| GCTGGAA | 1.84 |
| ACACTCT | 1.84 |
| TCACTTG | 1.84 |
| CCTCAGT | 1.84 |
| AGTGTAT | 1.84 |
| AACCTTT | 1.84 |
| ACACAAC | 1.84 |
| GGTGCGA | 1.84 |
| TACCGCA | 1.84 |
| AGAGACG | 1.84 |
| AAAGCAA | 1.84 |
| GTTGGAT | 1.84 |
| TTCGCAC | 1.84 |
| ACTGAGG | 1.84 |
| GTTGTCA | 1.84 |
| TTCCTTA | 1.84 |
| CCTGATG | 1.84 |
| CGAGGTA | 1.84 |
| TGACAAC | 1.84 |
| TCCCGAT | 1.84 |
| AGTCTTG | 1.84 |
| TCCGAGT | 1.83 |
| CGCGAGT | 1.83 |
| ATCCAAC | 1.83 |
| TGCGATG | 1.83 |
| GCTCTGT | 1.83 |
| CTGCAAT | 1.83 |
| GTAGTAA | 1.83 |
| TGTGTAA | 1.83 |
| GTTCGGA | 1.83 |
| CTGCTTT | 1.83 |
| TCTCGTC | 1.83 |
| TGCGTTT | 1.83 |
| CCTCGCA | 1.83 |
| ACTCGGC | 1.83 |
| TTAGGCG | 1.83 |
| CATCGTA | 1.83 |
| CCTCGTT | 1.83 |
| CTTGACA | 1.83 |
| TCGCTAG | 1.83 |
| TGTCTTC | 1.83 |
| CGACTAT | 1.83 |
| ATCGACC | 1.83 |
| GTAGACT | 1.83 |
| TTCCAGC | 1.82 |
| ATCGAAA | 1.82 |
| TCTCGGT | 1.82 |
| GACGGTA | 1.82 |
| AGACAAA | 1.82 |
| CCAGTTA | 1.82 |
| AACCGTT | 1.82 |
| CGAGAGA | 1.82 |
| TTCGCTA | 1.82 |
| ATTGTAG | 1.82 |
| GCACTTT | 1.82 |
| GCTGCGT | 1.82 |
| ATCGACG | 1.82 |
| TTCCGTA | 1.82 |
| CAAGACA | 1.82 |
| TTCCAAG | 1.82 |
| GTTGTCG | 1.82 |
| GACGGTT | 1.81 |
| TACCAAT | 1.81 |
| CCTCACT | 1.81 |
| AACCGCG | 1.81 |
| AGTGCGT | 1.81 |
| GCGCAAC | 1.81 |
| ACTCGTC | 1.81 |
| GAACTGT | 1.81 |
| ATTGGCG | 1.81 |
| TACGAAG | 1.81 |
| CGCGTTC | 1.81 |
| ATAGCGT | 1.81 |
| AACGACA | 1.81 |
| AATGAGG | 1.81 |
| CATCGAG | 1.81 |
| CCAGAAG | 1.81 |
| AGTCTAC | 1.81 |
| TCTCTCG | 1.81 |
| TAAGTCA | 1.81 |
| TAACAAT | 1.81 |
| TCTCTAG | 1.81 |
| CTACTTA | 1.81 |
| TAACTGG | 1.81 |
| GCTCGTT | 1.81 |
| TTCGGTT | 1.81 |
| ACGCAGC | 1.81 |
| ACCGACT | 1.81 |
| TACCGAA | 1.80 |
| CGACAGC | 1.80 |
| TCCGACG | 1.80 |
| ATTCGAC | 1.80 |
| TTCGAAA | 1.80 |
| GGCGAAC | 1.80 |
| TTTGTGC | 1.80 |
| CGTCTCT | 1.80 |
| AAAGACC | 1.80 |
| TGACTAA | 1.80 |
| CTGGATT | 1.80 |
| GAAGTAG | 1.80 |
| CGCGGTT | 1.80 |
| TGTGGTT | 1.80 |
| CGCGAGA | 1.80 |
| CAAGACT | 1.80 |
| TGTCTCG | 1.80 |
| TCACTGA | 1.80 |
| CTTGGAA | 1.79 |
| GTTCGCC | 1.79 |
| GCGGATG | 1.79 |
| TAACTGC | 1.79 |
| CGCGATC | 1.79 |
| TGCGGTA | 1.79 |
| AGAGACA | 1.79 |
| TTCCTGT | 1.79 |
| CTTCTCT | 1.79 |
| TCGCTTC | 1.79 |
| CGTCACA | 1.79 |
| TTCCTAG | 1.79 |
| ACGCTCT | 1.79 |
| GTTCGTC | 1.79 |
| AGAGCGT | 1.79 |
| AACGAGA | 1.79 |
| CACGAAC | 1.78 |
| AATCCGG | 1.78 |
| AACGGTT | 1.78 |
| ATCGAGA | 1.78 |
| GATCCGT | 1.78 |
| TACCTCT | 1.78 |
| AGCGAAG | 1.78 |
| CGTCGAA | 1.78 |
| ACACTAA | 1.78 |
| GTTGCAA | 1.78 |
| TACGAGA | 1.78 |
| ACAGACG | 1.78 |
| ACAGAGC | 1.78 |
| ATAGTGT | 1.78 |
| TGTCAAG | 1.78 |
| CTTGTAA | 1.78 |
| AGAGCGG | 1.77 |
| CTTCCGT | 1.77 |
| CTTCGAG | 1.77 |
| GGTCAAT | 1.77 |
| TAAGTGT | 1.77 |
| GTAGAGA | 1.77 |
| TGTGCGA | 1.77 |
| AGCGTTA | 1.77 |
| AGAGGTA | 1.77 |
| GTTGTGT | 1.77 |
| ACCGAAT | 1.77 |
| CAAGCGA | 1.77 |
| ATCGCAG | 1.77 |
| GCACAAA | 1.77 |
| ACGCACT | 1.77 |
| CCGCTTA | 1.77 |
| TACCTTT | 1.77 |
| CGTCTTA | 1.77 |
| TGTCGAA | 1.77 |
| CAAGACG | 1.77 |
| CCAGATG | 1.77 |
| TCAGTGA | 1.77 |
| TAAGACG | 1.76 |
| CTTCAGG | 1.76 |
| TTTCGAA | 1.76 |
| GAAGTCA | 1.76 |
| ATTGTTA | 1.76 |
| TTTGTCT | 1.76 |
| AAACAGG | 1.76 |
| CCTCGAT | 1.76 |
| CGAGAGC | 1.76 |
| CTACAAT | 1.76 |
| AGTCACG | 1.76 |
| TTAGTGT | 1.76 |
| ATTGCAG | 1.76 |
| AACCACT | 1.76 |
| CCTCGAA | 1.76 |
| TTGCGCT | 1.76 |
| ACCGATA | 1.76 |
| GTTCTAC | 1.76 |
| AACCGAA | 1.76 |
| AAAGCAG | 1.76 |
| TTAGCGC | 1.76 |
| GCGCTAT | 1.76 |
| GTCGCGT | 1.76 |
| ATTCCAA | 1.76 |
| TGACTAT | 1.75 |
| CCACAAA | 1.75 |
| TCCGAAC | 1.75 |
| TCTCGTA | 1.75 |
| CTTCTGG | 1.75 |
| CCGGAAT | 1.75 |
| ATAGAGG | 1.75 |
| AGCGGAA | 1.75 |
| GCTCGTA | 1.75 |
| AACCTAG | 1.75 |
| GTTCGAA | 1.75 |
| CTAGAAC | 1.75 |
| AACGAGC | 1.75 |
| GAACAAA | 1.75 |
| ACGCAGT | 1.75 |
| CCTCAAC | 1.75 |
| ACAGTTC | 1.75 |
| TATCGGT | 1.75 |
| GCAGAGA | 1.75 |
| GTTCCGT | 1.75 |
| CGAGACT | 1.75 |
| ACGCTAT | 1.75 |
| CTACTCT | 1.75 |
| GACGATG | 1.74 |
| ACCGAAG | 1.74 |
| TCACACT | 1.74 |
| TCTCACC | 1.74 |
| AGCGCTG | 1.74 |
| CGACTAA | 1.74 |
| TCTCGGA | 1.74 |
| CATGATC | 1.74 |
| GCAGTTA | 1.74 |
| TTTGTGG | 1.74 |
| TCAGGTA | 1.74 |
| AGCGCAA | 1.74 |
| GAAGCGA | 1.74 |
| CGCGACT | 1.74 |
| CGAGTAA | 1.73 |
| TTACGCT | 1.73 |
| GAAGACA | 1.73 |
| TATCGCC | 1.73 |
| GCTCTTG | 1.73 |
| TACGACG | 1.73 |
| GTAGAAC | 1.73 |
| GTGGATT | 1.73 |
| CTAGGTT | 1.73 |
| ACACTAT | 1.73 |
| TCTCTGC | 1.73 |
| AAACGAT | 1.73 |
| CTTCGAA | 1.73 |
| GAAGACG | 1.73 |
| TGCGGAA | 1.73 |
| TTGCAAC | 1.73 |
| CCTCAGA | 1.72 |
| GACGATC | 1.72 |
| CGAGTAT | 1.72 |
| GGCGATT | 1.72 |
| GTTGAGG | 1.72 |
| TTTCGTC | 1.72 |
| AAACGCT | 1.72 |
| CCGCTAA | 1.72 |
| TACCTGA | 1.72 |
| AAAGTGC | 1.72 |
| CATCTGG | 1.72 |
| GGTGAGT | 1.72 |
| GTTCTAG | 1.72 |
| ATTCGTG | 1.72 |
| AAAGGTA | 1.72 |
| TCACAGT | 1.72 |
| AATCCAC | 1.72 |
| ACGCTAA | 1.72 |
| TTTGTTC | 1.72 |
| ACTCGAA | 1.72 |
| AGTCATT | 1.72 |
| ATTCGGT | 1.72 |
| TGACTCT | 1.72 |
| ATCCTAT | 1.71 |
| GGTCGAA | 1.71 |
| AGTCAAG | 1.71 |
| CAACTCG | 1.71 |
| CGTGACT | 1.71 |
| CTACTTC | 1.71 |
| CTCGAAG | 1.71 |
| TCTCAGG | 1.71 |
| AGTGGTT | 1.71 |
| TGACTTA | 1.71 |
| GCTCTCT | 1.71 |
| ATACAAC | 1.71 |
| AACCTAC | 1.71 |
| GACGAAA | 1.71 |
| GTAGGTT | 1.70 |
| TTCGACG | 1.70 |
| GTAGCGA | 1.70 |
| CGTCTGT | 1.70 |
| GATCCGC | 1.70 |
| GAACAGA | 1.70 |
| AACCAGT | 1.70 |
| CTCGAGT | 1.70 |
| AGTGTGA | 1.70 |
| CTTCGAT | 1.70 |
| ACCGATG | 1.70 |
| TCGGATC | 1.70 |
| TCGCATT | 1.70 |
| TAGCGAA | 1.70 |
| TTGCAGA | 1.70 |
| GCTGAGC | 1.69 |
| TTTCTCT | 1.69 |
| GTTGTAT | 1.69 |
| TGACAAT | 1.69 |
| TCTCTAC | 1.69 |
| AGAGTAG | 1.69 |
| AATGACT | 1.69 |
| CCTGAAG | 1.69 |
| AGCGATT | 1.69 |
| ACTCGTG | 1.69 |
| AGAGTGA | 1.69 |
| GATCTAG | 1.69 |
| TTGGAAT | 1.69 |
| ACAGTAT | 1.69 |
| CAAGAGC | 1.68 |
| AAACGAC | 1.68 |
| TGAGGAA | 1.68 |
| CATGAGT | 1.68 |
| CAAGAGA | 1.68 |
| TCGCTCT | 1.68 |
| ACACTTA | 1.68 |
| AATCGCA | 1.68 |
| TCCGAGA | 1.68 |
| GCGCTAA | 1.68 |
| GACGAGA | 1.68 |
| CCTGAGA | 1.68 |
| AAAGTCG | 1.68 |
| TCTGTTC | 1.68 |
| CATCGAC | 1.68 |
| ATTGTCA | 1.68 |
| CTTGACT | 1.68 |
| CCTCTAT | 1.68 |
| ACGCAAC | 1.68 |
| ATAGGTA | 1.67 |
| CAACAAA | 1.67 |
| CCGCTAT | 1.67 |
| ATAGCGC | 1.67 |
| TCTGCGT | 1.67 |
| GCTCAGC | 1.67 |
| AGTCGCT | 1.67 |
| TCTGCAA | 1.67 |
| TCGGAGT | 1.67 |
| ATCGAGG | 1.67 |
| CTTGCGA | 1.67 |
| ATACAGT | 1.67 |
| TTCGTGA | 1.67 |
| TGTCGTA | 1.67 |
| GTTCATG | 1.66 |
| TTCGGTA | 1.66 |
| TCTCGCA | 1.66 |
| AGAGAAA | 1.66 |
| AATCGTC | 1.66 |
| AGTCTTC | 1.66 |
| CCTGTTT | 1.66 |
| CATGAAC | 1.66 |
| CCGCAAC | 1.66 |
| TCTCATA | 1.66 |
| GGTGAGA | 1.66 |
| AATGATT | 1.66 |
| TTTCCGG | 1.66 |
| TTCGGAT | 1.66 |
| ATTGACC | 1.66 |
| TTTGTTG | 1.66 |
| ATAGGAT | 1.65 |
| AACCTGC | 1.65 |
| TCACAGC | 1.65 |
| ACTCACC | 1.65 |
| CGACTTC | 1.65 |
| ATCGACT | 1.65 |
| TGAGCGA | 1.65 |
| AATGACG | 1.65 |
| CGAGTTG | 1.65 |
| TCTGAGG | 1.65 |
| GACGAGT | 1.65 |
| TATGAGA | 1.65 |
| ACACAAT | 1.65 |
| ATAGTGA | 1.65 |
| TTCCTCA | 1.65 |
| TCACTCT | 1.65 |
| CGAGTTC | 1.65 |
| CATCACC | 1.65 |
| TACCTAT | 1.65 |
| ATTGTGT | 1.65 |
| GGTGTTA | 1.65 |
| AGCGATG | 1.65 |
| CTTCACC | 1.65 |
| AAAGGTT | 1.65 |
| AATCATT | 1.64 |
| TTCGCTG | 1.64 |
| ACTGTGA | 1.64 |
| GCTCTTC | 1.64 |
| TACCGAC | 1.64 |
| ACACTTC | 1.64 |
| AGAGCGA | 1.64 |
| GTTCGCT | 1.64 |
| TGTCTTA | 1.64 |
| CACGATA | 1.64 |
| TAACACC | 1.64 |
| ATACTGT | 1.64 |
| ATTCGCA | 1.64 |
| TCACAAC | 1.64 |
| ATTCGTC | 1.64 |
| GTCGTAA | 1.64 |
| CAAGTAA | 1.64 |
| AGAGAAG | 1.64 |
| TCGCAAG | 1.64 |
| AGTCACA | 1.63 |
| TGTCATC | 1.63 |
| CTTCCGC | 1.63 |
| CATGAAG | 1.63 |
| TTAGCGG | 1.63 |
| GTTGCGC | 1.63 |
| CTTCGCG | 1.63 |
| TGTGACT | 1.63 |
| AGAGAGC | 1.63 |
| ACTGGTT | 1.63 |
| GGTGATG | 1.63 |
| AAACACT | 1.63 |
| GTTCGAC | 1.63 |
| AGTGTTT | 1.63 |
| TTAGCGT | 1.63 |
| TTCGACT | 1.63 |
| GTTCAGG | 1.63 |
| CGAGACA | 1.62 |
| GATCACC | 1.62 |
| AGACTTT | 1.62 |
| ATCGTAA | 1.62 |
| ATTGCAA | 1.62 |
| ACGCGTA | 1.62 |
| TAAGACA | 1.62 |
| GCTGACA | 1.62 |
| TGTCAGC | 1.62 |
| TCCGAAA | 1.62 |
| GCGGAAG | 1.62 |
| ACGCAGA | 1.62 |
| TCACTAT | 1.62 |
| GAAGACT | 1.61 |
| CTAGATC | 1.61 |
| GCGGATC | 1.61 |
| TACCAAA | 1.61 |
| TAACAGT | 1.61 |
| GTTCACG | 1.61 |
| CTTCGAC | 1.61 |
| ATAGGAA | 1.61 |
| GCAGAGT | 1.61 |
| TTGCAAT | 1.61 |
| GTCGTTT | 1.61 |
| CTTCGCA | 1.61 |
| AAAGTCT | 1.61 |
| CCCGATC | 1.61 |
| TCTCTTG | 1.61 |
| ATCGGCA | 1.61 |
| ATACAGC | 1.61 |
| TTAGTGA | 1.61 |
| TAAGTGA | 1.61 |
| GCTGTAT | 1.61 |
| TCGCACA | 1.60 |
| GTTCTGG | 1.60 |
| GTTGCGG | 1.60 |
| CCCGAAA | 1.60 |
| AATCGGG | 1.60 |
| GAACTGA | 1.60 |
| GTCGGTA | 1.60 |
| TGTCATT | 1.60 |
| TTCGAAC | 1.60 |
| ACTCATG | 1.60 |
| TGAGTAT | 1.60 |
| AACCTCG | 1.60 |
| TCAGGAT | 1.60 |
| CGTCGTA | 1.60 |
| CTTCATG | 1.60 |
| AACCGAC | 1.60 |
| TTCCTAT | 1.60 |
| GATCAGG | 1.59 |
| CGCCAAA | 1.59 |
| ACTGTTA | 1.59 |
| GCTCGCT | 1.59 |
| AAAGCGG | 1.59 |
| ATAGCGG | 1.59 |
| GTCGTGA | 1.59 |
| AGTCGTA | 1.59 |
| ATCCAAT | 1.59 |
| TTTGGTA | 1.59 |
| GCTGTGA | 1.59 |
| ATACTCA | 1.59 |
| TGTCTGA | 1.59 |
| TTCGAGC | 1.59 |
| ATTCGAG | 1.59 |
| ATTGTCG | 1.58 |
| ACCGAGT | 1.58 |
| CAACTAT | 1.58 |
| CAACTAA | 1.58 |
| ATTCGCC | 1.58 |
| GTTGTGA | 1.58 |
| TGCGACG | 1.58 |
| CCTGAAC | 1.58 |
| TACGACA | 1.58 |
| GTTCGCG | 1.58 |
| CTTCGTA | 1.58 |
| GATCGTC | 1.58 |
| TTCGAGT | 1.58 |
| CTCGATG | 1.58 |
| CGACGAC | 1.58 |
| TGTCACT | 1.58 |
| CCGCTTT | 1.58 |
| GCCGATA | 1.58 |
| GTCGACT | 1.58 |
| AATGACA | 1.58 |
| CCACTTT | 1.58 |
| ATACTGA | 1.58 |
| TTTCCAA | 1.58 |
| TTAGTTG | 1.57 |
| AAACAAT | 1.57 |
| GCAGGTT | 1.57 |
| ATACAAT | 1.57 |
| ACTGTAT | 1.57 |
| AACGATG | 1.57 |
| ATTGTTT | 1.57 |
| CAAGTAT | 1.57 |
| CATCGGT | 1.57 |
| TTTGTAG | 1.57 |
| AGTCGAC | 1.57 |
| TGCGAGT | 1.57 |
| TAAGCGA | 1.57 |
| GATCCGA | 1.57 |
| TTCGCGA | 1.57 |
| CTAGTTA | 1.57 |
| ACAGTAA | 1.57 |
| AGTCGTT | 1.57 |
| ACTCGAC | 1.57 |
| ATTCGGA | 1.56 |
| AACCTCT | 1.56 |
| GCGCTTT | 1.56 |
| AAAGCGC | 1.56 |
| GATGACA | 1.56 |
| AATCCAA | 1.56 |
| CACGATC | 1.56 |
| CTACAAA | 1.56 |
| TTGGAAA | 1.56 |
| CCTGATC | 1.56 |
| TCCGAAG | 1.56 |
| CCGGAAA | 1.56 |
| CGTCGCA | 1.56 |
| TTCGCTT | 1.56 |
| AGAGGTT | 1.56 |
| ACTCGCG | 1.56 |
| TGCGAAG | 1.56 |
| CGTCATT | 1.56 |
| TACCTAA | 1.56 |
| GTCGAAG | 1.56 |
| ATCCGAT | 1.56 |
| AGTGACA | 1.56 |
| TCAGTAT | 1.55 |
| TCGCTAA | 1.55 |
| AGCGTTT | 1.55 |
| GAAGTGT | 1.55 |
| ATCGCTC | 1.55 |
| CTTGATG | 1.55 |
| TCGCTGT | 1.55 |
| TTCGCAG | 1.55 |
| GAAGAGC | 1.55 |
| AACGACT | 1.55 |
| ATCCTGA | 1.55 |
| TTTGTCG | 1.55 |
| TGACTTC | 1.55 |
| AATCGCC | 1.55 |
| TCAGACA | 1.55 |
| TATCGAT | 1.55 |
| CTGCAAA | 1.54 |
| ATCGAAC | 1.54 |
| GTTCAAG | 1.54 |
| CATCAAG | 1.54 |
| TTTCGGA | 1.54 |
| ATCCGAA | 1.54 |
| CGCCAAT | 1.54 |
| TTCGAAT | 1.54 |
| TCTCTGT | 1.54 |
| TAACTAG | 1.54 |
| GTAGTTA | 1.54 |
| GCTCTCA | 1.54 |
| TTCGGTC | 1.54 |
| TATCCGT | 1.54 |
| TCGGAAC | 1.54 |
| CGCGATT | 1.54 |
| CAACTGA | 1.54 |
| AAAGTGG | 1.54 |
| ACAGCGA | 1.53 |
| TCTCATG | 1.53 |
| ATAGTTG | 1.53 |
| TGTGACG | 1.53 |
| CGACAAA | 1.53 |
| GTCGACA | 1.53 |
| TGTCTTT | 1.53 |
| TCACTAA | 1.53 |
| CGACAAC | 1.53 |
| GGTGATC | 1.53 |
| TTAGGAT | 1.53 |
| CTCGATC | 1.53 |
| GATGCGA | 1.53 |
| TCAGTTC | 1.53 |
| TTGGATT | 1.53 |
| AGTCTCA | 1.53 |
| CGTGAAC | 1.53 |
| GACGACT | 1.53 |
| CGACAAT | 1.53 |
| TTCCGAC | 1.52 |
| CATGAGA | 1.52 |
| TGAGACT | 1.52 |
| TGAGTAA | 1.52 |
| CACGAAA | 1.52 |
| GGCGATA | 1.52 |
| ACAGAGA | 1.52 |
| TTACTTG | 1.52 |
| ATACAGA | 1.52 |
| TAACTAC | 1.52 |
| CGTCTGA | 1.52 |
| TTCCAGA | 1.52 |
| TCTGGAA | 1.52 |
| TTTGGCG | 1.52 |
| GCCGATT | 1.52 |
| GTTCGCA | 1.52 |
| TCAGTTG | 1.52 |
| CAACTCA | 1.51 |
| AGTCAGA | 1.51 |
| TCTGGAT | 1.51 |
| GAAGTGA | 1.51 |
| TGAGAGC | 1.51 |
| TATCGGC | 1.51 |
| TTACAGC | 1.51 |
| ATACTTG | 1.51 |
| TTACTGT | 1.51 |
| AGTCTCT | 1.51 |
| ATTGTAT | 1.51 |
| GAAGTTC | 1.51 |
| TAACTGT | 1.51 |
| ACTCTGC | 1.51 |
| TACGACT | 1.51 |
| TTCGAGG | 1.51 |
| TTACAGT | 1.51 |
| TCACAGA | 1.51 |
| ACTCGTA | 1.51 |
| TTACTCA | 1.51 |
| CAAGAGT | 1.51 |
| TGACAAA | 1.51 |
| ACTGCGT | 1.50 |
| CCAGAAC | 1.50 |
| CTCGATA | 1.50 |
| TCACTTA | 1.50 |
| CATCGGA | 1.50 |
| GAACTTC | 1.50 |
| ATTGGAT | 1.50 |
| GTTCCGA | 1.50 |
| TCTGTAT | 1.50 |
| GCTCATT | 1.50 |
| CCCGATA | 1.50 |
| TACGATG | 1.50 |
| GTCGGTT | 1.50 |
| GGTGAAG | 1.50 |
| CAAGTTC | 1.50 |
| AACCAAC | 1.50 |
| AACCAGC | 1.49 |
| TTAGAGG | 1.49 |
| GTCGAGT | 1.49 |
| GATCGGA | 1.49 |
| AAACAAC | 1.49 |
| GGTCAAA | 1.49 |
| GTTGTTT | 1.49 |
| TTACTCG | 1.49 |
| TGTCGCT | 1.49 |
| TCGCTCA | 1.49 |
| CTTGACG | 1.49 |
| GTTGGTT | 1.49 |
| TGAGTTG | 1.49 |
| GAACTTG | 1.49 |
| AACCTGT | 1.49 |
| ATCCAGA | 1.49 |
| AACGAAT | 1.49 |
| TCGCATC | 1.49 |
| GGCGAAT | 1.49 |
| TTTCATA | 1.48 |
| TCAGACT | 1.48 |
| GCTGACG | 1.48 |
| TTTGCAG | 1.48 |
| GATCGGC | 1.48 |
| TACGAGT | 1.48 |
| GCAGATC | 1.48 |
| CAAGAAG | 1.48 |
| AGTCTGT | 1.47 |
| CGCGTTT | 1.47 |
| TCAGACG | 1.47 |
| ACTCTTG | 1.47 |
| GCTCAGT | 1.47 |
| ACAGTTA | 1.47 |
| ACGCTTT | 1.47 |
| GACGAAT | 1.47 |
| CCTCTAA | 1.47 |
| TTAGTTC | 1.47 |
| GACGATT | 1.47 |
| TTACACT | 1.47 |
| GATCTGC | 1.47 |
| CATCGCG | 1.47 |
| ATACTAT | 1.47 |
| AGTCATC | 1.47 |
| TTTGTCA | 1.47 |
| GCTGACT | 1.47 |
| TCACAAT | 1.47 |
| TCAGTAA | 1.46 |
| TATGAAA | 1.46 |
| CTTCCGA | 1.46 |
| AACCAAT | 1.46 |
| GAACTAA | 1.46 |
| CATCGTT | 1.46 |
| CGTCAGA | 1.46 |
| CAAGTTG | 1.46 |
| TAAGAGA | 1.46 |
| TGTCAGT | 1.46 |
| CAAGAAA | 1.46 |
| TTACAAC | 1.46 |
| AGTCTGA | 1.46 |
| GCTGTAA | 1.46 |
| CGTCAGC | 1.46 |
| GAACTCG | 1.45 |
| CTTGAAC | 1.45 |
| TATCTAG | 1.45 |
| TCTCTTA | 1.45 |
| TACGAGC | 1.45 |
| CCCGATT | 1.45 |
| GCTCTTA | 1.45 |
| TAAGTTG | 1.45 |
| CCGGATT | 1.45 |
| TGCGAGA | 1.45 |
| ATTCCGT | 1.45 |
| GATCGAG | 1.45 |
| TCCGATC | 1.45 |
| TGCGACT | 1.45 |
| TGCGAAC | 1.45 |
| ATACTTA | 1.45 |
| ATAGACG | 1.45 |
| CTAGATA | 1.45 |
| GAACTAT | 1.44 |
| TTTGACC | 1.44 |
| ATAGTAT | 1.44 |
| TAAGTAT | 1.44 |
| ACTGTAA | 1.44 |
| CAACTTG | 1.44 |
| ACTCTCG | 1.44 |
| GCTCATC | 1.44 |
| ACTCGTT | 1.44 |
| GAAGTAA | 1.44 |
| CGTCTAT | 1.44 |
| CGAGGTT | 1.44 |
| AAAGCGT | 1.44 |
| CGTCACT | 1.44 |
| ATCCTAA | 1.44 |
| TGTCTAT | 1.44 |
| GCTCTGA | 1.44 |
| ATAGTTC | 1.44 |
| TCACTTC | 1.43 |
| AACCTAT | 1.43 |
| GATGACT | 1.43 |
| ATAGACA | 1.43 |
| CACGAAT | 1.43 |
| TGCGAAT | 1.43 |
| ATTGTGA | 1.43 |
| CATCACA | 1.43 |
| GTCGGAA | 1.43 |
| CTCGAAT | 1.43 |
| TGTCGCA | 1.43 |
| CGTCTTT | 1.43 |
| TCGCTAC | 1.43 |
| TTAGTAT | 1.43 |
| ATACTAA | 1.43 |
| TCTGTGA | 1.43 |
| TATCGAG | 1.43 |
| GAACTTA | 1.42 |
| GCTGCGA | 1.42 |
| TCGCTGA | 1.42 |
| AATGAAG | 1.42 |
| ATCCAAA | 1.42 |
| TCTCTCA | 1.42 |
| TCGCTTT | 1.42 |
| AGTGTAA | 1.42 |
| TTCCAAC | 1.42 |
| TGCGACA | 1.42 |
| AATGAGC | 1.42 |
| CTTCTTG | 1.42 |
| ACAGACT | 1.42 |
| TCCGATG | 1.42 |
| AGTCACT | 1.42 |
| TGAGAGA | 1.42 |
| TTAGGTA | 1.42 |
| TAACAAA | 1.42 |
| TCGGATA | 1.42 |
| ATAGCGA | 1.42 |
| AGTGACT | 1.42 |
| ACTGACG | 1.42 |
| ACTCTAC | 1.42 |
| GACGAAC | 1.42 |
| TAAGTAA | 1.42 |
| GATCTGG | 1.41 |
| GCCGATC | 1.41 |
| ATAGGTT | 1.41 |
| TTCGATA | 1.41 |
| TTACTTC | 1.41 |
| CTTCATT | 1.41 |
| AGTCAGC | 1.41 |
| AGCGACT | 1.41 |
| GTTCGAT | 1.41 |
| TAAGAGC | 1.41 |
| ACTCTGT | 1.41 |
| ACTGACA | 1.41 |
| ATAGTAA | 1.41 |
| GCTGGTT | 1.41 |
| GTAGAGT | 1.41 |
| CATCATC | 1.41 |
| TTTGGAT | 1.40 |
| TCAGGAA | 1.40 |
| ACAGGTT | 1.40 |
| GTTCATT | 1.40 |
| ACTCGCA | 1.40 |
| AGTCTTT | 1.40 |
| CGCGATG | 1.40 |
| ATTGGTT | 1.40 |
| TATCATT | 1.40 |
| AAAGTCA | 1.40 |
| TGAGGTT | 1.40 |
| TATCGGA | 1.40 |
| TCAGCGA | 1.40 |
| AACCGAT | 1.40 |
| TTACTTA | 1.39 |
| AGAGACT | 1.39 |
| ATTGCGT | 1.39 |
| ACAGAGT | 1.39 |
| AGTCGAT | 1.39 |
| ACTGAGC | 1.39 |
| AACCAGA | 1.39 |
| TCTGAGC | 1.39 |
| GCTCAGA | 1.39 |
| TTCGATC | 1.39 |
| GCGCAAT | 1.39 |
| TCGGAAG | 1.39 |
| AATCGAA | 1.39 |
| TTCCGAA | 1.39 |
| TTACTGA | 1.39 |
| ATTGCGG | 1.38 |
| TTTGCGC | 1.38 |
| TGTCAGA | 1.38 |
| CGTCATC | 1.38 |
| ATCGGTA | 1.38 |
| ACGGAAT | 1.38 |
| CAACTTA | 1.38 |
| AATCATG | 1.38 |
| TCCGAAT | 1.38 |
| AATGATA | 1.38 |
| CCAGATC | 1.38 |
| ACGCAAT | 1.38 |
| ACGGATA | 1.38 |
| TAACAGA | 1.38 |
| TATCTTC | 1.38 |
| GATGATG | 1.38 |
| GTTGGAA | 1.38 |
| TTACTAA | 1.38 |
| TCTGACT | 1.38 |
| GATGACG | 1.38 |
| GGTGAAC | 1.38 |
| TCTGTTA | 1.38 |
| ATTCTTT | 1.37 |
| TTAGACG | 1.37 |
| CACGATT | 1.37 |
| TCAGAGC | 1.37 |
| TTCGACA | 1.37 |
| TTAGTAA | 1.37 |
| AGTCGCA | 1.37 |
| GATCACG | 1.37 |
| TTTGTTA | 1.37 |
| AGAGTTG | 1.37 |
| AGTCTTA | 1.37 |
| TATCGTT | 1.37 |
| TCTGGTT | 1.37 |
| GTCGAAC | 1.37 |
| ACTCTTT | 1.37 |
| CATGATT | 1.37 |
| AAACTAC | 1.37 |
| TGCGATA | 1.36 |
| CATCTGC | 1.36 |
| ACACAAA | 1.36 |
| AGAGTAT | 1.36 |
| GTTGTAA | 1.36 |
| GCTGTTA | 1.36 |
| GATGAGC | 1.36 |
| CTTGAAG | 1.36 |
| GTTGCGT | 1.36 |
| GCGGATT | 1.36 |
| TCTGACG | 1.36 |
| GCAGATG | 1.36 |
| TTGCAAA | 1.35 |
| TGTCAAC | 1.35 |
| TAAGACT | 1.35 |
| AATGAAC | 1.35 |
| TTTGTGT | 1.35 |
| ACTCGCT | 1.35 |
| TTCGCGT | 1.35 |
| TAACAGC | 1.35 |
| TCTGACA | 1.35 |
| GTTCTTT | 1.35 |
| TATCGCA | 1.35 |
| TCGCTTA | 1.35 |
| AGCGAGT | 1.35 |
| TTACTCT | 1.35 |
| TTACAGA | 1.34 |
| AATCGGT | 1.34 |
| AACCTCA | 1.34 |
| AATCGGC | 1.34 |
| TATCCGC | 1.34 |
| GAAGATA | 1.34 |
| GTAGTTT | 1.34 |
| GATCTTA | 1.34 |
| AGTGACG | 1.34 |
| TAAGTTC | 1.34 |
| TGTGATG | 1.34 |
| TTACTAT | 1.34 |
| CCGCAAT | 1.34 |
| GAAGTAT | 1.33 |
| ATCGGTT | 1.33 |
| TCGCAGA | 1.33 |
| GATCGTA | 1.33 |
| GCTCACT | 1.33 |
| ATACTCT | 1.33 |
| TATCGTG | 1.33 |
| TTCCTGA | 1.33 |
| TTTGCAA | 1.33 |
| CTAGAAA | 1.33 |
| TCGCTAT | 1.33 |
| TATCATG | 1.33 |
| GAACTCA | 1.33 |
| TTCCGAT | 1.33 |
| CTTCAAG | 1.32 |
| TACGATC | 1.32 |
| TTAGACA | 1.32 |
| CGTGATC | 1.32 |
| CGTGATG | 1.32 |
| AGTGAGC | 1.32 |
| CAAGAAC | 1.32 |
| ACGGAAA | 1.32 |
| CGTCAGT | 1.32 |
| GTTGACA | 1.32 |
| ACCGAAC | 1.32 |
| AGAGTAA | 1.32 |
| ATTCGCT | 1.31 |
| TGAGTTC | 1.31 |
| ACTGACT | 1.31 |
| GCGGATA | 1.31 |
| AAACTAG | 1.31 |
| AGCGACA | 1.31 |
| GCTCTAT | 1.30 |
| GTTCACC | 1.30 |
| GATCAAG | 1.30 |
| CCGCAAA | 1.30 |
| TTAGTTA | 1.30 |
| CTTGAGT | 1.30 |
| TCGCACT | 1.30 |
| AAAGACA | 1.30 |
| CTTGAGA | 1.30 |
| ATTGTAA | 1.30 |
| TCTGTAA | 1.30 |
| TGACTTT | 1.30 |
| CTAGAAT | 1.30 |
| ATTCATT | 1.30 |
| TGTGAGT | 1.30 |
| TTACAAT | 1.30 |
| ACTCAAG | 1.29 |
| GCTCTTT | 1.29 |
| TCTCACG | 1.29 |
| CGTGAGA | 1.29 |
| CAACTCT | 1.29 |
| ATCGGAT | 1.29 |
| CATGATA | 1.29 |
| AACGATC | 1.29 |
| ACTCTCT | 1.29 |
| CGACTTT | 1.29 |
| GATCGAT | 1.29 |
| TTTGCGG | 1.29 |
| GTTCTTG | 1.29 |
| TACGATA | 1.29 |
| CGTCAAC | 1.28 |
| CGAGAGT | 1.28 |
| CTAGTTT | 1.28 |
| TTAGGAA | 1.28 |
| ACTCACG | 1.28 |
| GCAGAAG | 1.28 |
| GAACTCT | 1.28 |
| ATCGAGT | 1.28 |
| TGTCTAA | 1.28 |
| TATCGAA | 1.28 |
| TAACTCA | 1.28 |
| ATACTTC | 1.28 |
| ACCGATT | 1.28 |
| ATTCGTT | 1.27 |
| TTAGGTT | 1.27 |
| AGTGCGA | 1.27 |
| AGTCAGT | 1.27 |
| AAAGTAG | 1.27 |
| CAACTTC | 1.27 |
| TCTCAAG | 1.27 |
| ACTCTTC | 1.27 |
| CGTGAGT | 1.27 |
| TCGCAGC | 1.27 |
| CTACTTT | 1.27 |
| GTCGATG | 1.27 |
| TTCGAAG | 1.26 |
| AGTCAAC | 1.26 |
| TCGCAGT | 1.26 |
| TATCAGG | 1.26 |
| AACCTAA | 1.26 |
| ATCGAAG | 1.26 |
| ACACTTT | 1.26 |
| TTCGATT | 1.26 |
| GCGCAAA | 1.26 |
| AATCGAT | 1.26 |
| AGTCTAA | 1.26 |
| CATCACG | 1.26 |
| TCTCACT | 1.26 |
| ATCGATA | 1.26 |
| AATCCGT | 1.26 |
| AAAGACG | 1.25 |
| ACTCTTA | 1.25 |
| CTTCTGC | 1.25 |
| CATCTCT | 1.25 |
| TAAGAAG | 1.25 |
| AAACAGT | 1.25 |
| ACTCACA | 1.25 |
| TAACTAA | 1.25 |
| AGAGTTC | 1.25 |
| AGAGAGT | 1.25 |
| AAACTGG | 1.25 |
| GTCGAGC | 1.25 |
| AATGAGT | 1.25 |
| AGTGAGA | 1.25 |
| GAAGTTG | 1.24 |
| AATGAAT | 1.24 |
| GATCACT | 1.24 |
| GAAGAGT | 1.24 |
| ATTCGAA | 1.24 |
| ATAGAGC | 1.24 |
| TTTGCGT | 1.24 |
| ACGCAAA | 1.24 |
| ATCGATT | 1.24 |
| TAAGAGT | 1.24 |
| TAACTAT | 1.24 |
| ATCGAGC | 1.24 |
| ACTGTTT | 1.24 |
| TATCTGG | 1.24 |
| TAAGATC | 1.24 |
| TATCTAC | 1.24 |
| TGAGAGT | 1.24 |
| GATCACA | 1.24 |
| TGCGATT | 1.24 |
| CCTGATA | 1.24 |
| ACTCATT | 1.24 |
| CCAGTTT | 1.23 |
| AAAGTGT | 1.23 |
| TTTCGCG | 1.23 |
| CTTCATC | 1.23 |
| ATTCTTC | 1.23 |
| TTTCCGC | 1.23 |
| TTTCCGT | 1.23 |
| AACCAAA | 1.22 |
| CTTGATC | 1.22 |
| ATTGCGC | 1.22 |
| TGAGTTA | 1.22 |
| GCTCGAT | 1.22 |
| TAACTGA | 1.22 |
| TTTCCGA | 1.22 |
| ACTCGAT | 1.22 |
| TTCCTAA | 1.22 |
| AAAGACT | 1.22 |
| TTCGGAA | 1.22 |
| ATAGACT | 1.22 |
| CAAGTTA | 1.21 |
| GCTCAAC | 1.21 |
| TGTGAGA | 1.21 |
| AGTCTAT | 1.21 |
| GATGAAG | 1.21 |
| ATAGTTA | 1.21 |
| TCAGGTT | 1.21 |
| ACGGATT | 1.21 |
| TGTGATC | 1.20 |
| GTTGAGC | 1.20 |
| AGTGATC | 1.20 |
| CGTCTAA | 1.20 |
| TCTCACA | 1.20 |
| AAAGCGA | 1.20 |
| TTTGGTT | 1.20 |
| ATCGGAA | 1.20 |
| CTTCTCG | 1.20 |
| AATCGTG | 1.20 |
| TATCCGA | 1.20 |
| CTTCTTA | 1.20 |
| ATACAAA | 1.19 |
| ATTCTGG | 1.19 |
| GTTCATC | 1.19 |
| TTCCAAT | 1.19 |
| TCCGATT | 1.19 |
| TCAGTTA | 1.19 |
| AAACAAA | 1.19 |
| CATCTTG | 1.19 |
| TCGGATT | 1.19 |
| AAACACC | 1.19 |
| GATCGCT | 1.19 |
| AGCGAAT | 1.19 |
| TATCGAC | 1.19 |
| TTAGACT | 1.19 |
| GACGATA | 1.19 |
| CATCTCG | 1.19 |
| ATTCTAG | 1.18 |
| CATCTCA | 1.18 |
| GATCATC | 1.18 |
| AATCGGA | 1.18 |
| GCAGAAC | 1.18 |
| AACCTGA | 1.18 |
| TTTGTAT | 1.18 |
| TATCGTC | 1.18 |
| TATCGTA | 1.18 |
| TTAGCGA | 1.18 |
| GAAGAAT | 1.18 |
| GCGGAAA | 1.18 |
| ATTGGAA | 1.18 |
| CCTCAAT | 1.18 |
| GATCGTT | 1.18 |
| TAACTTG | 1.18 |
| ACTGCGA | 1.18 |
| AACGAGT | 1.18 |
| GTCGATC | 1.17 |
| CATGAAT | 1.17 |
| ACAGTTT | 1.17 |
| GTAGATA | 1.17 |
| AATCGCG | 1.17 |
| CGAGAAG | 1.17 |
| TCTCATT | 1.17 |
| ACAGAAG | 1.17 |
| AGAGATG | 1.17 |
| TTTGTTT | 1.17 |
| GATCGAC | 1.17 |
| ACTCTCA | 1.17 |
| GCTGAGT | 1.16 |
| TCGCAAC | 1.16 |
| AAACTGT | 1.16 |
| CAAGATG | 1.16 |
| TAACTCG | 1.16 |
| GATGAGT | 1.16 |
| TACGAAT | 1.16 |
| TCGGAAA | 1.16 |
| AATCTAG | 1.16 |
| CTTCTCA | 1.16 |
| AATCGTT | 1.16 |
| CCAGATA | 1.15 |
| GTTCTGC | 1.15 |
| GCTGAGA | 1.15 |
| CGAGATG | 1.15 |
| TCTCAGC | 1.15 |
| AATCGTA | 1.15 |
| TAACTTA | 1.15 |
| AAACTCG | 1.15 |
| GTTGACG | 1.15 |
| GCTGAAG | 1.15 |
| ACTCAGC | 1.15 |
| GTTCTTA | 1.15 |
| AACGATA | 1.15 |
| ATTCATG | 1.14 |
| AAAGTGA | 1.14 |
| CGAGTTA | 1.14 |
| TTAGAGC | 1.14 |
| GGTGTTT | 1.14 |
| TGAGAAG | 1.14 |
| GATGATC | 1.14 |
| TCAGAGA | 1.14 |
| AAAGAGT | 1.14 |
| GTTCTCT | 1.14 |
| GTTGACT | 1.13 |
| TGTGAAG | 1.13 |
| CTTCACG | 1.13 |
| TAAGAAC | 1.13 |
| CGCGATA | 1.13 |
| CTAGATT | 1.13 |
| AGCGATC | 1.13 |
| CTTCAGC | 1.13 |
| AATGAGA | 1.13 |
| ATTGAGG | 1.12 |
| TCACAAA | 1.12 |
| TCTCATC | 1.12 |
| TCCGATA | 1.12 |
| TAAGTTA | 1.12 |
| CATCAGC | 1.12 |
| TGAGATG | 1.11 |
| TCAGAGT | 1.11 |
| TAAGAAA | 1.11 |
| TTACTTT | 1.11 |
| TCTCTGA | 1.11 |
| ATAGAGA | 1.11 |
| GCTCTAA | 1.10 |
| AGTGAGT | 1.10 |
| CATCTGT | 1.10 |
| AAAGAGC | 1.10 |
| TCTGCGA | 1.10 |
| GATGAAC | 1.10 |
| GTTCACA | 1.10 |
| CTTCACA | 1.10 |
| TTTCTTG | 1.10 |
| GATCTTG | 1.09 |
| TAAGATG | 1.09 |
| CCAGAAA | 1.09 |
| TCACTTT | 1.09 |
| GTTCAAC | 1.09 |
| AAAGTAA | 1.09 |
| AAAGAAC | 1.09 |
| CGCGAAT | 1.09 |
| ATTCCGC | 1.09 |
| TTTCTAG | 1.09 |
| AATCCGC | 1.09 |
| CATCTTA | 1.09 |
| TCTCTAT | 1.09 |
| CATCTTT | 1.09 |
| TTTGTGA | 1.08 |
| TAACTCT | 1.08 |
| AACGATT | 1.08 |
| CGTGAAG | 1.08 |
| ATTCGTA | 1.08 |
| TAACTTC | 1.08 |
| GATGAGA | 1.08 |
| TGTGAAC | 1.08 |
| AAACTAA | 1.08 |
| ACTCATC | 1.08 |
| CTTCTGT | 1.07 |
| ACTCACT | 1.07 |
| AGAGTTA | 1.07 |
| AAACTAT | 1.07 |
| TCGGAAT | 1.07 |
| AATCAGG | 1.07 |
| AGTGATG | 1.06 |
| GAACTTT | 1.06 |
| TTTGTAA | 1.06 |
| ACAGATG | 1.06 |
| TTACAAA | 1.06 |
| AAAGAAT | 1.06 |
| ATAGAAG | 1.06 |
| AATCCGA | 1.06 |
| TTTCTCG | 1.05 |
| CATCAAC | 1.05 |
| TGTCAAT | 1.05 |
| GTAGAAA | 1.05 |
| ACTCTGA | 1.05 |
| GTTGCGA | 1.05 |
| TTAGTTT | 1.05 |
| AAACTGA | 1.05 |
| TCAGAAG | 1.05 |
| ATTCGCG | 1.05 |
| TATCAAG | 1.05 |
| ATAGTTT | 1.05 |
| CGAGAAA | 1.05 |
| GCTGATG | 1.05 |
| GTTCTCA | 1.05 |
| TTAGAGA | 1.04 |
| GTTGAGT | 1.04 |
| TTAGAGT | 1.04 |
| CATCACT | 1.04 |
| CATCAGT | 1.04 |
| ATCGATC | 1.04 |
| GCGGAAT | 1.04 |
| TATCATC | 1.03 |
| AAACTTC | 1.03 |
| TAACTTT | 1.03 |
| AATGAAA | 1.03 |
| TTTGAGG | 1.03 |
| GTTGATG | 1.03 |
| ATACTTT | 1.03 |
| GAAGTTA | 1.03 |
| ACTGATG | 1.03 |
| AAACAGA | 1.03 |
| AAACTTA | 1.03 |
| ACTCTAT | 1.02 |
| GTTCAGC | 1.02 |
| CATCAGA | 1.02 |
| TATCACC | 1.02 |
| AGAGAAC | 1.02 |
| CTTGATA | 1.02 |
| ATTCAGG | 1.02 |
| TCGCAAT | 1.02 |
| CTCGATT | 1.01 |
| AAACTCT | 1.01 |
| ACTGAGT | 1.01 |
| TGAGAAC | 1.01 |
| TTCCAAA | 1.01 |
| AAAGTTC | 1.01 |
| TCTGAGT | 1.01 |
| ACAGAAA | 1.01 |
| GATCAAC | 1.01 |
| CAAGATC | 1.01 |
| AAAGTAT | 1.01 |
| ATTGACA | 1.00 |
| TCTGATG | 1.00 |
| AATCTTC | 1.00 |
| CCTCAAA | 1.00 |
| TCTGTTT | 1.00 |
| GAAGTTT | 1.00 |
| AGTCAAT | 1.00 |
| TAAGTTT | 1.00 |
| GCTCAAT | 0.99 |
| TCAGATG | 0.99 |
| GTTCTGT | 0.99 |
| CTTCACT | 0.99 |
| CAACTTT | 0.99 |
| ATAGAGT | 0.99 |
| GTTGAGA | 0.99 |
| GATCAGC | 0.99 |
| CTTCTAT | 0.99 |
| TATCTTT | 0.99 |
| AATCTTT | 0.99 |
| GTTCACT | 0.99 |
| AGTGAAG | 0.99 |
| CGTCAAT | 0.99 |
| TCTCTAA | 0.99 |
| TCAGTTT | 0.99 |
| CCTGATT | 0.99 |
| CGAGTTT | 0.98 |
| TTTCATT | 0.98 |
| TTTGACG | 0.98 |
| GTCGATA | 0.98 |
| CATGAAA | 0.98 |
| GTTCTCG | 0.98 |
| GATCTTT | 0.98 |
| CGAGAAC | 0.98 |
| TTTGCGA | 0.98 |
| AGCGATA | 0.98 |
| AAACAGC | 0.98 |
| GGTGATA | 0.98 |
| AAACTTG | 0.98 |
| ATAGAAA | 0.98 |
| ACTCAAC | 0.97 |
| CTTCTAA | 0.97 |
| CATCTGA | 0.97 |
| GCTGAAC | 0.97 |
| TCTGAGA | 0.97 |
| ATTGACT | 0.97 |
| AAAGTTG | 0.97 |
| TCTCAGT | 0.97 |
| CTTCTGA | 0.97 |
| GATGATA | 0.97 |
| ACAGAAC | 0.97 |
| AATCGAG | 0.97 |
| CCAGAAT | 0.97 |
| CCTGAAT | 0.96 |
| GAAGATT | 0.96 |
| GATCTCT | 0.96 |
| CAAGTTT | 0.96 |
| TCTCAAC | 0.96 |
| GTTCTAT | 0.96 |
| ACTCTAA | 0.96 |
| GTAGATT | 0.96 |
| TTTGGAA | 0.96 |
| ACTCAGA | 0.96 |
| ACTGAAG | 0.96 |
| GTTGAAG | 0.96 |
| ACTCAGT | 0.96 |
| AGAGTTT | 0.95 |
| TTAGAAG | 0.95 |
| AATCTAC | 0.95 |
| CAAGAAT | 0.95 |
| GTTGATC | 0.95 |
| TGTCAAA | 0.95 |
| ATTCCGA | 0.95 |
| TCGCAAA | 0.95 |
| TACGATT | 0.95 |
| TCTGAAG | 0.94 |
| CATCTAT | 0.94 |
| ATTGCGA | 0.94 |
| TATCGCT | 0.94 |
| ACTGAGA | 0.94 |
| CTTCAAC | 0.94 |
| GCTGTTT | 0.94 |
| GTTCAGT | 0.94 |
| ATTCAAG | 0.94 |
| AGTGAAC | 0.94 |
| TTTCTGG | 0.93 |
| TCAGAAC | 0.93 |
| AAAGTTA | 0.93 |
| ATAGAAC | 0.93 |
| ATTGAGC | 0.93 |
| TCAGAAA | 0.92 |
| GTTCAGA | 0.92 |
| ATTCATC | 0.92 |
| CATCAAT | 0.92 |
| GATCAGT | 0.92 |
| GTTCTGA | 0.92 |
| TGAGTTT | 0.92 |
| CTTGAAT | 0.92 |
| AATCTGG | 0.92 |
| AAACTCA | 0.92 |
| CTTCAGT | 0.91 |
| GATCTCG | 0.91 |
| TATCTTG | 0.91 |
| CATCTAA | 0.91 |
| TCTCAGA | 0.91 |
| TCTCAAA | 0.91 |
| ATTCTTG | 0.91 |
| GTTGAAC | 0.91 |
| GGTGAAT | 0.91 |
| TTTCTAC | 0.91 |
| ATTCTCT | 0.91 |
| GCTGATC | 0.90 |
| TTTCATG | 0.90 |
| ATAGATG | 0.90 |
| AGAGATC | 0.90 |
| GATCTGT | 0.90 |
| CTTCAGA | 0.90 |
| AGTCAAA | 0.90 |
| AAAGATG | 0.90 |
| GTAGAAT | 0.90 |
| AATCATC | 0.90 |
| TTTGACT | 0.90 |
| TTTCACC | 0.89 |
| TTTCTTA | 0.89 |
| AAACTTT | 0.89 |
| ATTCACC | 0.89 |
| CAAGATA | 0.89 |
| TTAGATG | 0.88 |
| TTTGACA | 0.88 |
| ATTCTCG | 0.88 |
| TGAGAAA | 0.88 |
| TTAGAAA | 0.88 |
| TATCTCT | 0.87 |
| GCTCAAA | 0.87 |
| GTTCTAA | 0.87 |
| ACTGAAC | 0.87 |
| TAAGAAT | 0.87 |
| ACAGATC | 0.87 |
| ATTCACG | 0.87 |
| CCTGAAA | 0.87 |
| ATTGATG | 0.87 |
| CGTGATA | 0.87 |
| ACTGATC | 0.87 |
| TATCTCG | 0.87 |
| GATGAAT | 0.86 |
| GGTGAAA | 0.86 |
| GATCAGA | 0.86 |
| CGAGATC | 0.86 |
| TATCTAT | 0.85 |
| CCAGATT | 0.85 |
| GCAGAAA | 0.85 |
| GATCTAT | 0.85 |
| ATTCTAC | 0.85 |
| TATCTTA | 0.85 |
| ATAGATC | 0.85 |
| TATCTGC | 0.85 |
| GATCTCA | 0.84 |
| TTAGAAC | 0.84 |
| AATCAAG | 0.84 |
| TCTGATC | 0.84 |
| TTTCTGT | 0.84 |
| GCAGATA | 0.84 |
| GTTCAAT | 0.84 |
| TTTGAGA | 0.83 |
| CTTGAAA | 0.83 |
| TCTGAAC | 0.83 |
| AATCACC | 0.83 |
| ATTCTTA | 0.83 |
| TGTGATA | 0.83 |
| TTTCAGG | 0.83 |
| TATCACG | 0.82 |
| TTTGAGC | 0.82 |
| TATCACA | 0.82 |
| ATTGACG | 0.82 |
| GGTGATT | 0.82 |
| CTTGATT | 0.82 |
| GATGATT | 0.82 |
| AAAGTTT | 0.82 |
| CGTCAAA | 0.81 |
| CATCAAA | 0.81 |
| GATGAAA | 0.81 |
| GATCAAT | 0.81 |
| TTTCAAG | 0.80 |
| TATCTCA | 0.80 |
| TGAGATC | 0.80 |
| ATTGAGT | 0.80 |
| GATCTAA | 0.80 |
| TTTCATC | 0.79 |
| ACTCAAT | 0.79 |
| TAAGATA | 0.79 |
| CGAGATA | 0.79 |
| ATAGATA | 0.79 |
| TTAGATC | 0.78 |
| AGAGATA | 0.78 |
| TCAGATC | 0.78 |
| ATTCTAT | 0.78 |
| CTTCAAT | 0.77 |
| ATTGATC | 0.77 |
| GATCTGA | 0.77 |
| GCAGAAT | 0.77 |
| ACAGAAT | 0.77 |
| TATCAGC | 0.77 |
| CGTGAAT | 0.77 |
| ATTGAAG | 0.77 |
| ATTGAAC | 0.77 |
| ATAGAAT | 0.76 |
| TCTCAAT | 0.76 |
| AGAGAAT | 0.76 |
| ATTCAAC | 0.76 |
| AAAGATA | 0.76 |
| ACAGATA | 0.76 |
| TTTCTAT | 0.75 |
| CGTGATT | 0.75 |
| GTTGATA | 0.75 |
| TATCTGT | 0.75 |
| TGAGATA | 0.75 |
| ATTCACA | 0.75 |
| ATTGAGA | 0.75 |
| AATCTTA | 0.75 |
| TTTGATG | 0.74 |
| TTTCTGC | 0.74 |
| CAAGATT | 0.74 |
| AATCTTG | 0.73 |
| TGAGAAT | 0.73 |
| TATCAGA | 0.73 |
| CGAGAAT | 0.73 |
| TTTCTCA | 0.72 |
| ATTCTGT | 0.72 |
| TATCAGT | 0.72 |
| AATCACT | 0.72 |
| TATCAAT | 0.72 |
| GATCAAA | 0.72 |
| ACTCAAA | 0.72 |
| TAAGATT | 0.72 |
| TTTGAAG | 0.71 |
| AAAGATC | 0.71 |
| AATCAAT | 0.71 |
| TATCACT | 0.71 |
| TGTGAAT | 0.71 |
| ATTCAGC | 0.71 |
| TTTCTGA | 0.71 |
| ATTCTGC | 0.71 |
| GTTGAAT | 0.71 |
| AATCAGC | 0.71 |
| AGTGATA | 0.71 |
| AATCTCG | 0.70 |
| AGTGATT | 0.70 |
| TCTGATA | 0.70 |
| TTTGAGT | 0.70 |
| ATTCAAT | 0.70 |
| ATTCTCA | 0.70 |
| TATCAAC | 0.70 |
| CTTCAAA | 0.70 |
| AAAGATT | 0.70 |
| AGTGAAA | 0.69 |
| ATTGATA | 0.69 |
| ACTGATA | 0.69 |
| AATCTCT | 0.69 |
| GCTGATT | 0.69 |
| AATCAAC | 0.68 |
| TTAGAAT | 0.68 |
| TATCTGA | 0.68 |
| AATCTGC | 0.68 |
| AATCACA | 0.68 |
| GTTGATT | 0.67 |
| ATTGATT | 0.67 |
| GTTCAAA | 0.67 |
| ATTGAAA | 0.67 |
| TGTGAAA | 0.67 |
| TTTCTAA | 0.67 |
| ACTGAAA | 0.67 |
| TCAGATA | 0.67 |
| TTTGAAC | 0.67 |
| CGTGAAA | 0.66 |
| TTTGATC | 0.66 |
| GTTGAAA | 0.66 |
| GCTGAAA | 0.66 |
| TATCTAA | 0.66 |
| AATCACG | 0.66 |
| ATTCACT | 0.66 |
| TCAGAAT | 0.66 |
| ATTCAGT | 0.66 |
| TTTCACG | 0.65 |
| TGTGATT | 0.65 |
| ATTGAAT | 0.65 |
| ATTCTAA | 0.65 |
| TTTCACA | 0.65 |
| GCTGATA | 0.65 |
| GCTGAAT | 0.65 |
| TTTCACT | 0.65 |
| AGTGAAT | 0.65 |
| ACTGAAT | 0.64 |
| TTTCAGC | 0.64 |
| TCTGAAA | 0.64 |
| TTTCAAC | 0.63 |
| TTTCAGT | 0.63 |
| ATTCAGA | 0.63 |
| TTTCAGA | 0.63 |
| CGAGATT | 0.63 |
| AATCTGT | 0.62 |
| TTAGATA | 0.62 |
| TCAGATT | 0.62 |
| GCAGATT | 0.62 |
| AATCTGA | 0.62 |
| AGAGATT | 0.62 |
| AATCTCA | 0.61 |
| TATCAAA | 0.61 |
| ATTCTGA | 0.61 |
| TTTCAAT | 0.60 |
| TCTGAAT | 0.60 |
| ACAGATT | 0.59 |
| ATTCAAA | 0.59 |
| TGAGATT | 0.59 |
| TTTGATA | 0.59 |
| TTTGAAT | 0.58 |
| AATCTAT | 0.57 |
| AATCAGA | 0.57 |
| AATCTAA | 0.56 |
| TTTGAAA | 0.56 |
| AATCAGT | 0.56 |
| ATAGATT | 0.54 |
| TCTGATT | 0.54 |
| TTTGATT | 0.53 |
| AATCAAA | 0.52 |
| TTAGATT | 0.52 |
| ACTGATT | 0.52 |
| TTTCAAA | 0.50 |
