# Supplementary material for: Bisulfite probing reveals DNA structural intricacies
Source: Nucleic Acids Res. 2023 Mar 7;51(7):3261–9. doi: 10.1093/nar/gkad115 (PMC10123088; doi:10.1093/nar/gkad115)
Supplement: gkad115_Supplemental_Files [file gkad115_supplemental_files.zip › Table_S1.docx]

**Table S1:** Sequences of degenerate oligonucleotides and corresponding reference sequences for each of the primer pairs used for PCR amplification of bisulfite-modified genomic DNA.

**Primer Reference sequence Degenerate sequence Amplicon**

**name length**

F1 AAACAGTTTTTCCTTCCTGGTTCAGA AAAYARTTTTTYYTTYYTRRTTYARA 1873

R1 GTGAATGTTATGTGCCAGCTTTTCT RTRAATRTTATRTRYYARYTTTTYT

F2 GTGGAAAATTTGCCTCCTAAAATGT RTRRAAAATTTRYYTYYTAAAATRT 1777

R2 AACCCAGAAACCTGCTTGGTATAAAT AAYYYARAAAYYTRYTTRRTATAAAT

F3 TGTTTGTTGCAATAGGAAGAACTTG TRTTTRTTRYAATARRAARAAYTTR 1672

R3 TTTCCTCTCCCATGTCAAGTTTAGA TTTYYTYTYYYATRTYAARTTTARA

F4 CAGAAATATGGAAGCGTTTTGGTAAA YARAAATATRRAARYRTTTTRRTAAA 1771

R4 CAAAATGTCCTCCATTTCAGTCAAT YAAAATRTYYTYYATTTYARTYAAT

F5 TTGAATTTTTCTTCCTTTGAAAACCA TTRAATTTTTYTTYYTTTRAAAAYYA 1897

R5 AAGCATTGTCGTATTTCTTCGCTTAT AARYATTRTYRTATTTYTTYRYTTAT

F6 TGTGATGAAAAATATCCTTTCTGCCT TRTRATRAAAAATATYYTTTYTRYYT 1945

R6 GCAATTCCCAAAATCTGAAGTGAGTA RYAATTYYYAAAATYTRAARTRARTA

F7 ACAACATTGTGGAAAGTAGTCATCTC AYAAYATTRTRRAAARTARTYATYTY 1892

R7 AAATACAGGTAGTTTGTCGAGAGCAAGT AAATAYARRTARTTTRTYRARARYAART

F8 AGGACTCCTTTTAAAACATGGTGCTA ARRAYTYYTTTTAAAAYATRRTRYTA 1894

R8 TGACCTGGCTCTTTGTTTCTTTTT TRAYYTRRYTYTTTRTTTYTTTTT

F9 CTTTGTCCTGTTAATTCAGGAAAGCAT YTTTRTYYTRTTAATTYARRAAARYAT 1884

R9 CACTTCACTTTTATGTGGCATATGAACC YAYTTYAYTTTTATRTRRYATATRAAYY
