# Supplementary material for: Bisulfite probing reveals DNA structural intricacies
Source: Nucleic Acids Res. 2023 Mar 7;51(7):3261–9. doi: 10.1093/nar/gkad115 (PMC10123088; doi:10.1093/nar/gkad115)
Supplement: gkad115_Supplemental_Files [file gkad115_supplemental_files.zip › Table_S4.docx]

| **Sequence** | **Predictor** | **Estimate** | **Std. Error** | **T value** | **P value** |
| --- | --- | --- | --- | --- | --- |
| **DCD** | **A-tract** | **-0.29** | **0.12** | **-2.4** | **0.0132** |
|  | A-T content (± 4 bp) | -0.074 | 0.012 | -6.3 | 2 x 10^-10^ |
|  | T at position minus 1 | -1.06 | 0.04 | -27 | 2 x 10^-16^ |
|  | T at minus 2 | -0.22 | 0.036 | -6 | 1.7 x 10^-9^ |
|  | T at minus 3 | -0.22 | 0.035 | -6.2 | 6.5 x 10^-10^ |
| **DCCD** | **A-tract** | **-0.45** | **0.13** | **-3.37** | **0.00078** |
|  | A-T content (± 4 bp) | -0.094 | 0.015 | -6.41 | 2.3 x 10^-10^ |
|  | T at minus 1 | -0.53 | 0.041 | -13.2 | 2 x 10^-16^ |
|  | T at minus 2 | -0.052 | 0.04 | -1.3 | 0.2 |
|  | T at minus 3 | -0.069 | 0.042 | -1.6 | 0.1 |
| **DCCCD** | **A-tract** | **-0.62** | **0.197** | **-3.14** | **0.0019** |
|  | A-T content (± 4 bp) | -0.065 | 0.019 | -3.5 | 0.00055 |
|  | T at minus 1 | -0.52 | 0.05 | -10.4 | 2 x 10^-16^ |
|  | T at minus 2 | -0.12 | 0.053 | -2.18 | 0.03 |
|  | T at minus 3 | 0.011 | 0.055 | 0.2 | 0.84 |
|  |  |  |  |  |  |

**Table S4**: Statistical models (generalized linear; quasibinomial family in R) showing the effects of 5'-neighbouring A-tracts on reactivity at DCD, DCCD and DCCCD. These were significant when controlling for the possible confounding effects of local AT-content (number of A:T base pairs within 8 bp) and 5' T residues. Only 5’ C’s were considered for DCCD and DCCCD. "Minus" indicates position to the 5' side of the 5' C.
