# Supplementary material for: Bisulfite probing reveals DNA structural intricacies
Source: Nucleic Acids Res. 2023 Mar 7;51(7):3261–9. doi: 10.1093/nar/gkad115 (PMC10123088; doi:10.1093/nar/gkad115)
Supplement: gkad115_Supplemental_Files [file gkad115_supplemental_files.zip › Table_S5.docx]

| **5' Nucleotide** | **Poly-C length** | **3’ Nucleotide** | **N 5’-end mutations** | **N 3’-end mutations** | **5':3' ratio** |
| --- | --- | --- | --- | --- | --- |
| A | 2 | A | 962 | 791 | 1.22 |
| A | 2 | G | 77 | 965 | 0.08 |
| A | 2 | T | 1071 | 888 | 1.21 |
| G | 2 | A | 733 | 529 | 1.39 |
| G | 2 | G | 113 | 1278 | 0.09 |
| G | 2 | T | 984 | 695 | 1.42 |
| T | 2 | A | 757 | 696 | 1.09 |
| T | 2 | G | 87 | 1108 | 0.08 |
| T | 2 | T | 977 | 831 | 1.18 |
| D | 2 | W | 5484 | 4430 | 1.24 |
|  |  |  |  |  |  |
| A | 3 | A | 310 | 203 | 1.53 |
| A | 3 | G | 49 | 341 | 0.14 |
| A | 3 | T | 259 | 173 | 1.50 |
| G | 3 | A | 196 | 175 | 1.12 |
| G | 3 | G | 50 | 488 | 0.10 |
| G | 3 | T | 193 | 162 | 1.19 |
| T | 3 | A | 287 | 282 | 1.02 |
| T | 3 | G | 56 | 492 | 0.11 |
| T | 3 | T | 260 | 256 | 1.02 |
| D | 3 | W | 1505 | 1251 | 1.20 |
|  |  |  |  |  |  |
| A | 4 | A | 107 | 67 | 1.60 |
| A | 4 | G | 18 | 149 | 0.12 |
| A | 4 | T | 111 | 47 | 2.36 |
| G | 4 | A | 49 | 30 | 1.63 |
| G | 4 | G | 22 | 108 | 0.20 |
| G | 4 | T | 58 | 48 | 1.21 |
| T | 4 | A | 93 | 78 | 1.19 |
| T | 4 | G | 17 | 130 | 0.13 |
| T | 4 | T | 54 | 85 | 0.64 |
| D | 3 | W | 472 | 355 | 1.33 |
|  |  |  |  |  |  |
| A | 5 | A | 47 | 21 | 2.24 |
| A | 5 | G | 13 | 20 | 0.65 |
| A | 5 | T | 24 | 8 | 3.00 |
| G | 5 | A | 27 | 21 | 1.29 |
| G | 5 | G | 11 | 28 | 0.39 |
| G | 5 | T | 21 | 9 | 2.33 |
| T | 5 | A | 25 | 39 | 0.64 |
| T | 5 | G | 8 | 34 | 0.24 |
| T | 5 | T | 17 | 21 | 0.81 |
| D | 3 | W | 161 | 119 | 1.35 |

**Table S5:** Number of de novo mutations at the 3’ and 5’ ends of poly-C_n_ genome-wide where n=2–5. The mutation data were from Kessler et al. (2020).
